# Development of a Divergent Synthesis Strategy for 5-Sulfonyl-Substituted Uracil Derivatives

**DOI:** 10.1021/acs.joc.4c01357

**Published:** 2024-10-18

**Authors:** Lukas von Bredow, Alexander Fürll, Maik Tretbar

**Affiliations:** Institute for Drug Discovery, Leipzig University Medical School, Leipzig University, Brüderstraße 34, 04103 Leipzig, Germany

## Abstract

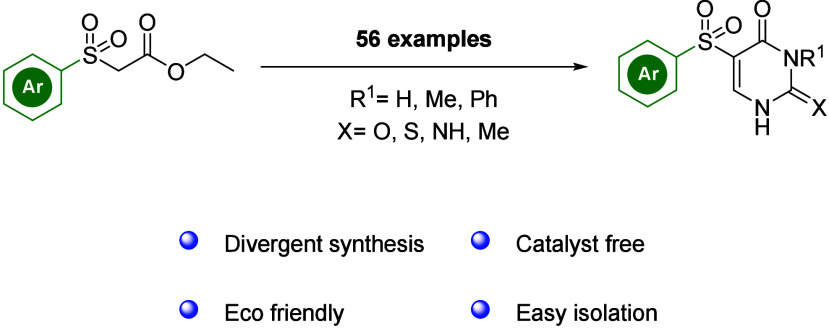

An efficient, diversity-orientated synthesis of 5-sulfone-substituted uracils was established. The use of protecting groups to synthesize sulfones from *N*-heterocycles was avoided. Various heterocycles were synthesized for the first time from favorable, easily accessible starting materials. Diversity-orientated syntheses are important for the medicinal chemistry of virostatics and chemotherapeutics. This approach provides a broad substrate tolerance and excellent yields of up to 98%.

Natural uracil derivatives such as uridine and thymidine are essential structures in biology as they are the building blocks of RNA and DNA, respectively. The pyrimidine core has long been recognized as a privileged structure in medicinal chemistry.^1,2^ A wide range of biologically active compounds containing uracil motifs are known, particularly in the fields of chemotherapy and antiviral treatments.^3−10^ Specifically 5-subsituted uracil bases and nucleosides have proven to be highly effective anticancer drugs.^11^ The best-known drugs are 5-fluorouracil (**I**) and eniluracil (**II**) (Figure 1) with profound activity against solid tumors.^12,13^

**Figure 1:**
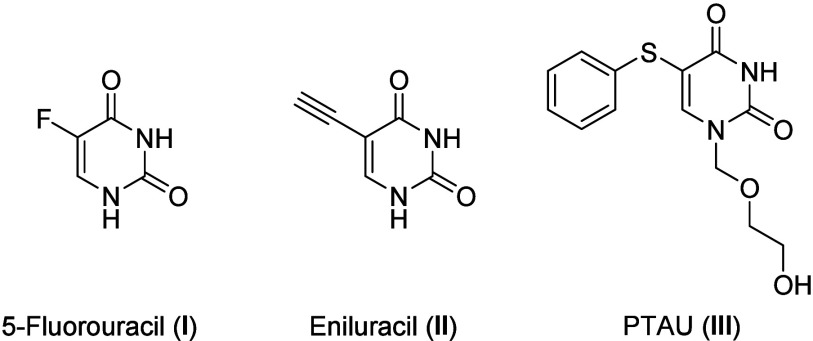
Selected examples of 5-susbstituted uracils.

Despite an intensive search for alternatives due to 5-fluorouracils (**I**) high toxicity, only a few alternatives have been found to date. One example is PTAU (**III**), which offers comparable efficacy with lower toxicity and improved pharmacokinetics.^14^ Due to their intriguing profiles, uracil derivatives have been intensively studied in various fields, including organic synthesis, medicinal chemistry, and materials chemistry.^15−18^ In recent years, enormous efforts have been devoted to the site-selective synthesis and modification of these heterocycles in order to increase their structural diversity and applicability (Scheme 1). In particular, the incorporation of bioactive moieties or functional groups into their scaffold could lead to further applications.^19−21^

**Scheme 1:**
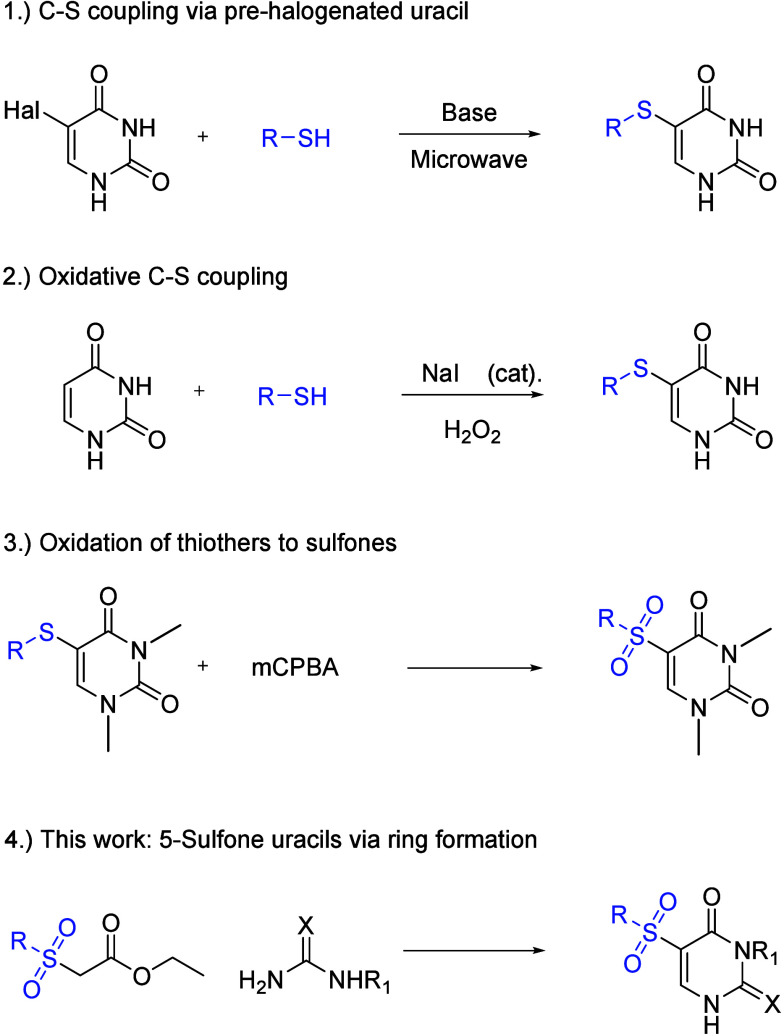
Previous Protocols for the Synthesis of 5-Sulfur/5-Sulfone Uracil Derivatives (**1**, **2**) and Our Approach (**3**)

In general, the introduction of sulfur at the 5-position has been well-studied. Known methods involve either nucleophilic substitution of halogenated uracils (Scheme 1, reaction 1) or oxidative coupling of thiols to uracil (Scheme 1, reaction 2).^21^ However, the introduction of a sulfone at the 5-position remains difficult. Oxidation of thioethers is only possible with methylated or protected nitrogens, as free NH groups form *N*-oxides with oxidizing agents like hydrogen peroxide or *meta*-chlorobenzoic acid (Scheme 1, reaction 1).^22,23^ However, sulfones are playing an increasingly important role in drug development due to their metabolic stability and diverse intermolecular interaction profile. Therefore, they form a valuable bioisosteric group to carbonyls.^24,25^

Because of our interest in the synthesis of diverse chemical libraries for biochemical purposes,^26^ we were determined to develop a protocol for the simple, versatile preparation of 5-sulfone-substituted uracil derivatives. Herein, we report an efficient strategy to synthesize 5-sulfonyl pyrimidines from acyclic precursors (Scheme 1, reaction 4).

In our initial attempt, the oxidation of 5-thio-substituted uracil with hydrogen peroxide or *meta*-chloroperbenzoic acid failed due to *N*-oxide formation. Inspired by the condensation of diethyl malonate with triethyl orthoformate (**3**) and urea (**2a**),^27^ we hypothesized that a 5-sulfone uracil could be obtained by exchanging one ester with a sulfone (**1a**). Interestingly, the first reaction already yielded 20% of the desired condensation product (**4a**) along with a side product (**S4**) that we identified as the corresponding amine (2.3.4, Scheme S8). In the next step we optimized the procedure toward temperature, time, and reagent equivalents (Table 1). We hypothesized that the byproduct (**S4**) formation is promoted by the ethanol formed, as this attacks the carbonyl carbon of the urea, which has a low electron density. To confirm our hypothesis, we stirred the condensation product (**4a**) with ethanol at 100 °C and saw complete conversion to the amine (**S4**). Conversely, opening the reaction system (shifting the equilibrium in the vessel) led to a significant increase in the yield of the desired product (**4a**). This led to a nearly complete suppression of the formation of the byproduct (**S4**, Table 1).

**Table 1:**
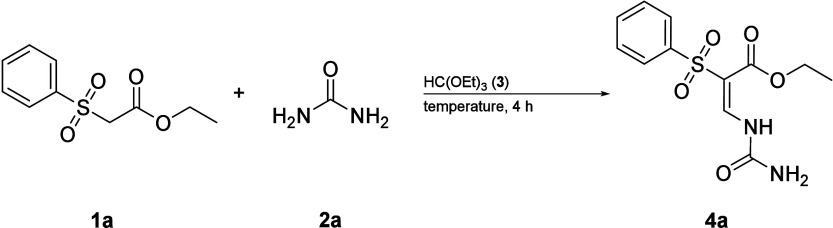
Optimization of the Condensation^a^

Entry	**2a** (equiv)	**3** (equiv)	Temp (°C)	Yield (%)
1	1.5	1.1	110	20
2	1.5	1.1	120	34
3	1.5	1.1	130	55
4	1.5	1.1	140	51
5	2.0	1.1	130	59
6	3.0	1.1	130	58
7	1.0	1.0	130	52
7	2.0	1.5	130	64
8	3.0	2.0	130	84
9	3.0	2.0	130	52^b^
10	3.0	2.0	130	91^c^

a Reaction conditions: **1a** (0.22 mmol, 50 mg, 1 equiv) at the designated temperature in a closed reaction vessel for 4 h, isolated yields given.

b Reaction was carried out in a microwave (150 W max.).

c Reaction vessel was opened after 5 min.

The condensation products (**4**) can be easily isolated by filtration and used in the next step. After optimizing the reaction, we continued with the direct conversion of the intermediates, as they were clean and the conversion was almost complete. For this purpose, the reaction was taken up in ethanol, and sodium ethanolate was added as a base. The reaction worked with *N*-monosubstituted urea (**2b, c**), yielding the *N*3-isomer (**6, 7**) as the single product (Scheme 2). Sterically more demanding urea, such as *N*-phenylurea (**2c**) exhibited comparable yields and regioselectivity compared to *N*-methylurea (**2b**). On the other hand, *N*,*N*′-disubstituted ureas did not lead to the formation of the desired uracils.

**Scheme 2:**
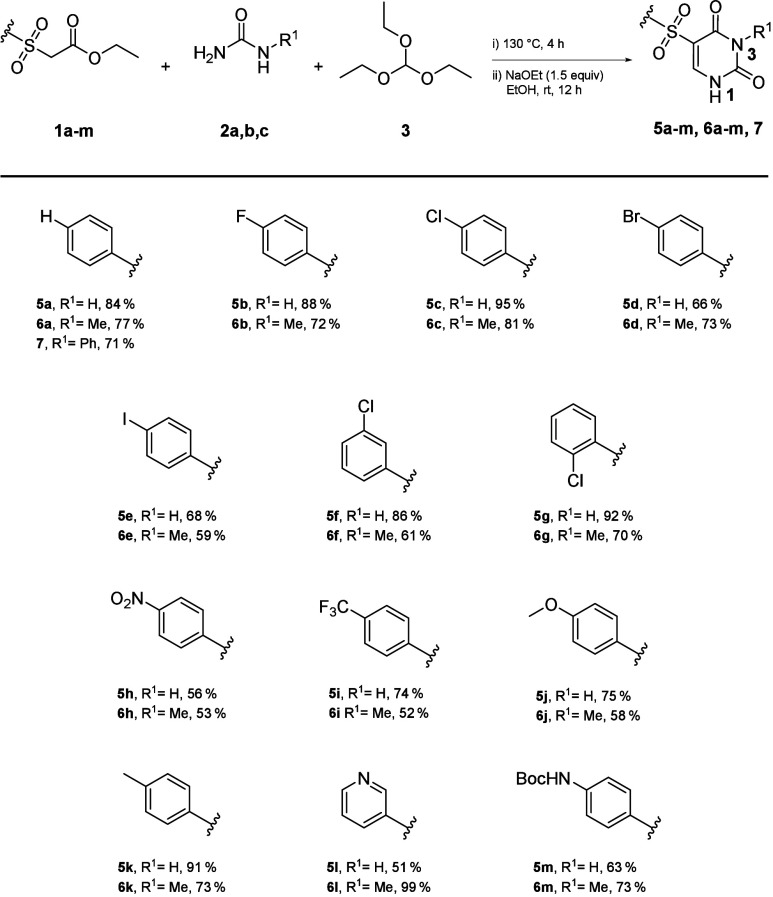
Substrate Scope of One-Pot Uracil Derivatives

Our next attempt was to apply our procedure to the synthesis of thiouracils (**11**–**13**, Scheme 3). This reaction did not proceed under the various conditions. The corresponding enol ethers (**9**) were prepared in a first reaction using triethyl orthoformate (**3**) and acetic anhydride (**8**). This step did not work for the 3-and 4-pyridine derivatives (**9l, o**). The enol ethers (**9**) were then converted to the corresponding thiouracils (**11**–**13**) with *N*-methylthiourea (**9a, b**) in the presence of sodium ethanolate in ethanol. Additionally, *N*-phenylthiourea (**9c**) was investigated in a further example, which demonstrated comparable reactivity. Yields of 30–98% were obtained. *N*,*N′*-substituted thioureas again did not give the desired products.

**Scheme 3:**
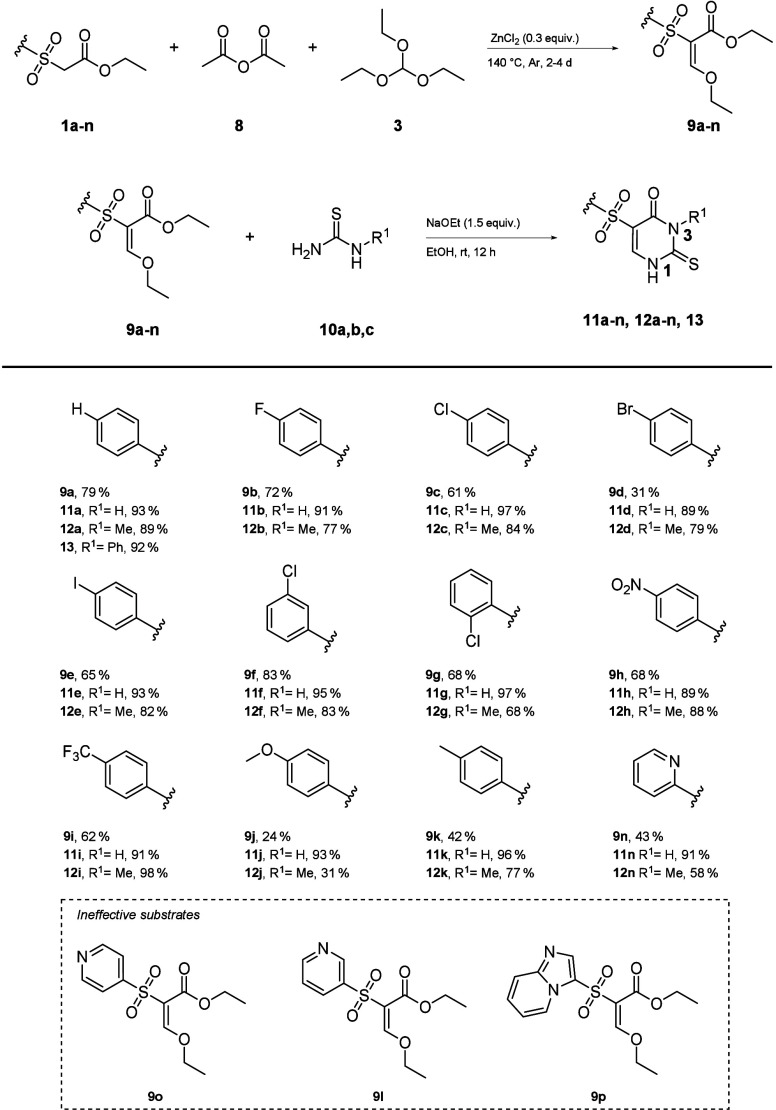
Substrate Scope and Yield of Thiouracil Derivative Synthesis

Interestingly, very small amounts of the *N*1-isomer (<5%) were found in the reactions with *N*-methylthiourea (**10b**). Only the methoxy-substituted substrate (**9j**) led to the formation of two structural isomers (**12j**, **S5**, Supporting Information, 2.4.4) in similar proportions. The products were isolated cleanly by filtration. This makes the workup very simple and efficient.

To further expand the scope of this application, the reactions were conducted with a methyl group in place of an aryl group (Scheme 4). These reactions were found to be equally effective, but due to the increased hydrophilicity, an alternative workup procedure was required (see the Supporting Information for procedures for **5r**, **6r**, **11r**, **12r**, and **s**).

**Scheme 4:**
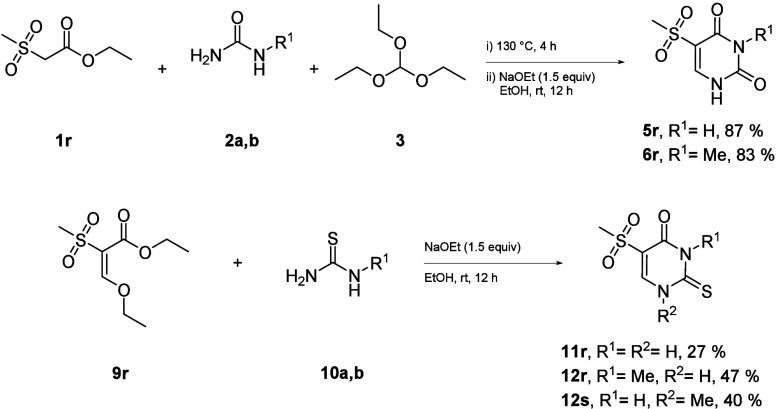
Feasibility with Alkyl Substituents

The high tolerance of functional groups under the specified harsh conditions is notable. The presence of electron-withdrawing and -donating substituents, halogens, and certain heterocycles are tolerated irrespective of their position.

The application of protecting groups is constrained by the formation of enol ethers when acetic anhydride is used at elevated temperature and ring formation under basic conditions. However, the use of a Boc-protecting group demonstrated stability under conditions of the ring closure involving urea (Scheme 2) (**5m**, **6m**).

This was followed by the scale-up experiments (see the Supporting Information, 2.3.2 and 2.3.3). The scale-ups at a scale of 10 mmol also resulted in good yields of 81% for **5a** and 88% for **11a** (compared to 84% and 93%).

The main advantage of synthesizing enol ethers (**9**) first is the possibility of using different nucleophiles for ring closure. The employment of guanidine hydrochloride (**14a**) as a nucleophile broadened the scope to encompass sulfone-isocytosines (**15**). The reaction with substituted guanidines [phenylguanidin (**14b**) and Boc-protected arginine (**14c**)] proceeded with the same regioselectivity as that with urea. Additionally, acetamidine hydrochloride (**16**) proved to be effective, facilitating the synthesis of 5-sulfone-2-methyl-pyrimidinols (**17**). This methodology enables the synthesis of a variety of 5-sulfone heterocycles for the first time (Scheme 5).

**Scheme 5:**
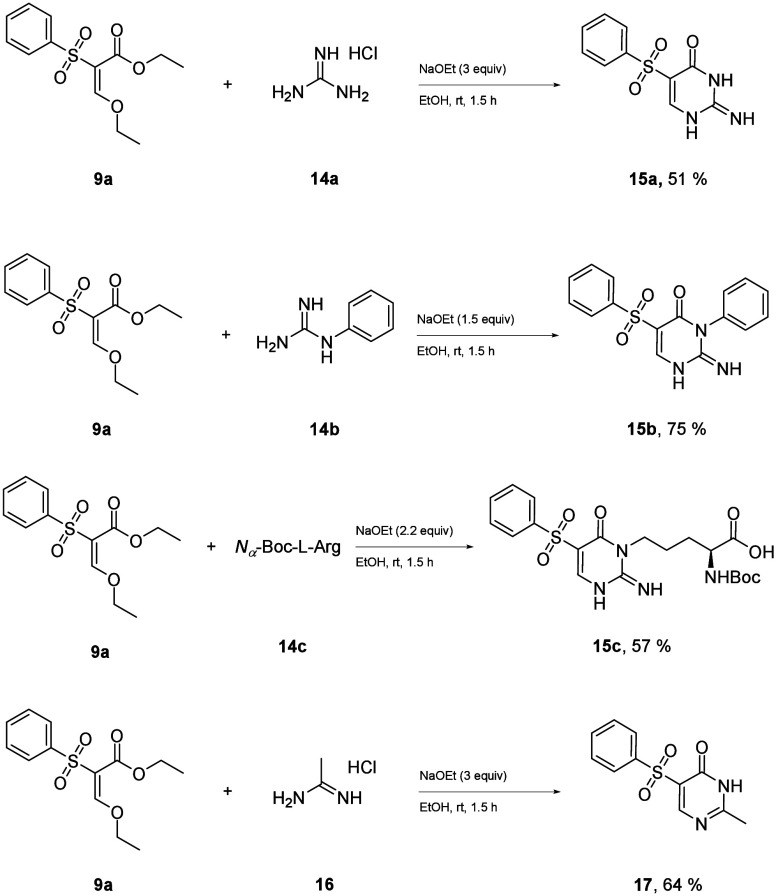
Further Accessible Sulfone Heterocycles

From a follow-up reaction perspective, thiouracils (**11**–**13**) have the advantage of a higher selectivity in nucleophilic substitution reactions. This extends the scope of possible bioactive molecules even more. An example reaction of a derivative (**11a**) with benzyl bromide (**18**) gave a high yield of a single product (**19**, Scheme 6).

**Scheme 6:**
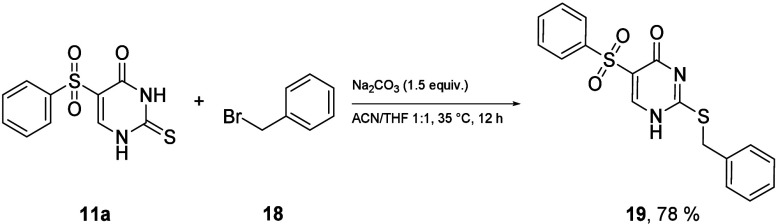
Follow-up Reactions

In summary, we present the first access to different 5-sulfone-substituted uracils. By a divergent approach, different derived heterocycles become accessible for the first time, facilitating the synthesis of chemically diverse libraries. The reagents are nontoxic and inexpensive. The reactions presented are simple to carry out, can be performed, and scaled up with a high substrate tolerance. The final products were readily isolated by filtration. Because the ring itself is built up, the sulfone group can be brought along, and the formation of *N*-oxides is avoided. Overall, these findings illustrate the versatility and potential of these reactions for further derivatization and library synthesis in a medicinal chemistry context.

## Data Availability

The data underlying this study are available in the published article and its Supporting Information.

## Supporting Information Available

Complete experimental details and characterization data for synthesized compounds (PDF)
