# Supplementary material for: Development of a Divergent Synthesis Strategy for 5-Sulfonyl-Substituted Uracil Derivatives
Source: J Org Chem. 2024 Oct 18;89(21):15990–4. doi: 10.1021/acs.joc.4c01357 (PMC11536380; doi:10.1021/acs.joc.4c01357)
Supplement: Supplementary file 1 — jo4c01357_si_001.pdf [file jo4c01357_si_001.pdf]

Development of a divergent synthesis strategy for 5-sulfonyl substituted uracil derivatives

Lukas von Bredow[§], Alexander Fürll[§], Maik Tretbar*

Institute for Drug Discovery, Leipzig University Medical School, Leipzig University, Brüderstraße 34, 04103 Leipzig, Germany

**Contact: maik.tretbar@uni-leipzig.de*

Table of Contents

1. General Information 2
2. Experimental Section 2
 2.1 Preceding Syntheses 2
 2.2 General Reaction Procedures (GP) 4
  2.2.1 Formation of the Uracils (GP1) 4
  2.2.2 Formation of the Enol ethers (GP2) 5
  2.2.3 Formation of the Thiouracils (GP3) 5
 2.3 Optimization of Reaction Conditions and scale-up 7
  2.3.1 Optimization of the Enol ether synthesis 7
  2.3.2 Scale-up of 5a at a 10 mmol scale 8
  2.3.3 Scale-up of 11a at a 10 mmol scale 8
  2.3.4 Synthesis of the condensation intermediate 4a 9
  2.3.5 Synthesis of Guanidine derivatives (15a-c) 10
  2.3.6 Synthesis of 5-(Benzenesulfonyl)-2-methyl-1,4-dihydropyrimidin-4-one (17) 11
  2.3.7 Synthesis of 5-(Benzenesulfonyl)-2-(benzylsulfanyl)-1,4-dihydropyrimidin-4-one (19) ..12
 2.4 Yields and Characterizations 13
  2.4.1 Starting materials 1a-n (sulfonyl acetates) 13
  2.4.2 Uracils 5a-r, 6a-r, 7 18
  2.4.3 Enol ethers 8a-r 28
  2.4.4 Thiouracils 11a-r, 12a-r, 13 34
3. NMR Spectra 45
4. References 147

1. General Information

All reactions were carried out in dried glassware and if necessary, under argon atmosphere. Unless otherwise noted, all reagents and solvents were purchased form commercial suppliers and used without further purification. Dry ethanol and dry sodium ethanolate were purchased from Thermo Scientific in sealed glass bottles. ^{1}H,^{13}C{^{1}H} and ^{19}F NMR spectra were recorded from a *Varian Mercury plus* or a *Bruker Avance III HD 400* (^{1}H: 400 MHz, 300 MHz; ^{19}F: 282 MHz, 376 MHz; ^{13}C{^{1}H}: 101 MHz, 75 MHz). Stated chemical shifts (*δ*, ppm) are relativ to the solvent residual signals. $CDCl_3$ (^{1}H: 7.26 ppm, ^{13}C{^{1}H}: 77.00 ppm), D_2O (^{1}H: 4.79 ppm, ^{13}C{^{1}H} was referenced with the absolute frequency of the ^{1}H solvent signal according to the recommendations of the IUPAC [1,2]) and DMSO-d_6 (^{1}H: 2.50 ppm, ^{13}C{^{1}H}: 39.52 ppm). NMR data are reported as following: chemical shift, multiplicity (s = singlet, d = duplet, br s = broad singlet, t = triplet, q = quartet, p = quintet, m = multiplet, dd = duplet of duplets, dt = duplet of triplets, tt = triplet of triplets), coupling constants (Hz) and integrals. HR-MS (ESI) were recorded using *Bruker Daltonics MicrOTOF*. LR-MS (EI) were recorded using gas chromatograph *Agilent 8890/5977B/MSD*. Reactions were monitored using thin-layer chromatography, with pre-coated plates form *Merck* (silica gel 60 F_{254}). RP-HPLC *Thermo Fisher Scientific UltiMate 3000* was used for additional reaction monitoring and purity determination. For product purifiaction *Teledyne Isco CombiFlash® NexTGen 300+* with *REDISEP® RF SILCIA GEL Disposal Flash Columns* and *KNAUER AZURA®* preparative HPLC system with a *MACHEREY-NAGEL VP 250/32 NUCLEODUR C18 HTec, 5 µm* was used.

2. Experimental Section

2.1 Preceding Syntheses

Scheme S1. Preparation of the starting materials **S1a-l.**

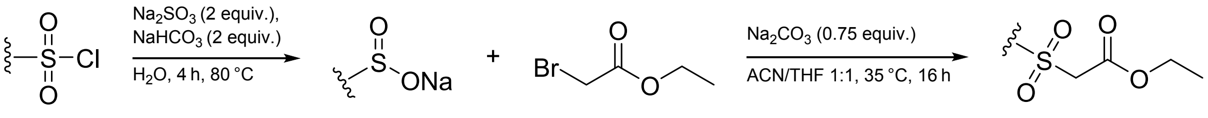

S1a-n **S2a-n** **S3** **1a-n**

The sodium sulfinates **S2a-n** were either purchased or prepared according to [3]. Therefore, a round bottom flask was charged with the corresponding sulfonyl chlorides **S1a-n** (15 mmol, 1 equiv.), sodium sulfite (30 mmol, 2 equiv.) and sodium hydrogencarbonate (30 mmol, 2 equiv.) and solved in 70 mL water. After the reaction was heated at 80 °C in an oil bath for 1 h, the solution was concentrated in vacuo. The crude product was suspended in 100 mL ethanol. The suspension was then filtered, and the filtrate concentrated in vacuo. The sodium sulfinates **S2a-n** were obtained as crystalline powder in sufficient yields and puritys and used without further purification.

The sulfonyl acetates **1a-n** were prepared under standard nucleophilic substitution conditions. Therefore, a round-bottom flask was charged with the corresponding sodium sulfinate **S2a-n** (10 mmol, 1 equiv.), sodium carbonate (7.5 mmol, 0.75 equiv.) and ethyl bromoacetate (**S3**, 2.26 mL, 20 mmol, 2 equiv.) and solved in 50 mL of a 1:1 mixture of acetonitrile and tetrahydrofuran. After the reaction was heated for 16 h at 35 °C in an oil bath, the solution was concentrated in vacuo. The crude products were purified by flash column chromatography (cyclohexane/ethyl acetate).

2.2 General Reaction Procedures (GP)

2.2.1 Formation of the Uracils (GP1)

Scheme S2. Preparation of the uracils **5a-m, 6a-m**

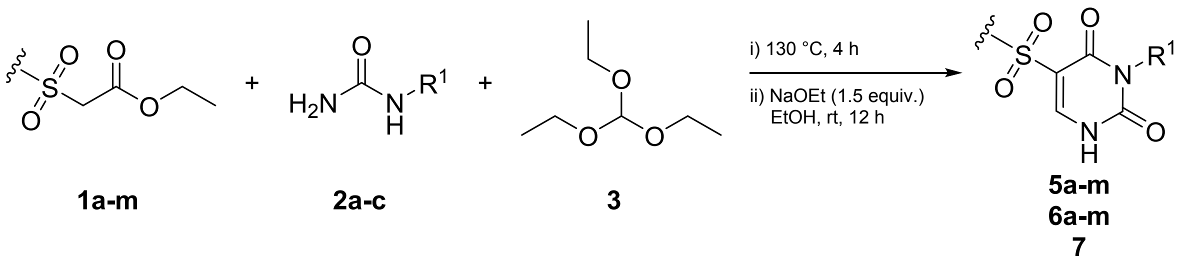

General synthesis procedure 1 (GP1)

A reaction vial was charged with the sulfonyl acetates **1a-m** (0.25 mmol, 1 equiv.), urea (**2a**), *N*-methylurea (**2b**) or *N*-phenylurea (**2c**) (0.75 mmol, 3 equiv.) and triethyl orthoformate (**3**) (0.50 mmol, 2 equiv.). The vial was equipped with a magnetic stirring bar and sealed. The reaction was heated for 5 min at 130 °C in an aluminum heat block. In some cases, the reaction could be stirred, in others reaction became solid. However, tests showed that stirring had no influence on the reaction progress. The vial was then opened, to allow formed ethanol do evaporate. The open reaction was heated for additional 4 h at 130 °C in an aluminum heat block. After cooling to room temperature 1.4 mL dry ethanol and 140 µL (0.38 mmol, 1.5 equiv.) of a dry 21 wt% sodium ethanolate solution was added under argon atmosphere. The vial was closed and stirred for 12 h at room temperature. After completion the reaction was concentrated in vacuo. The crude product was solved in 5 mL water. The solution was then filtered through a glass drip (pore size: 10-16 µM). The filtrate was acidified with concentrated hydrochloric acid (pH = 1) and filtered through a glass drip (pore size: 10-16 µM). The precipitate was washed with 0.1 M hydrochloric acid and dried to afford the desired uracil derivative **5a-m** resp. **6a-m** or **7**.

2.2.2 Formation of the Enol ethers (GP2)

Scheme S3. Preparation of the enol ethers **8a-m**

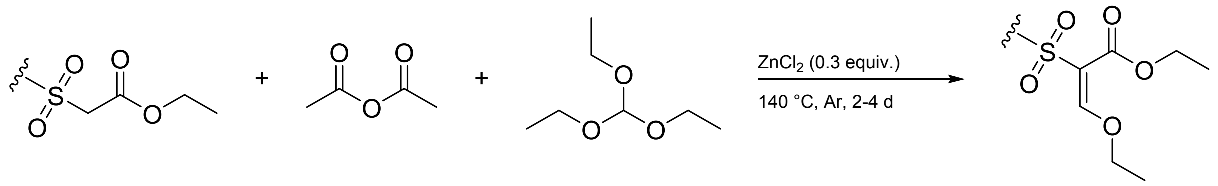

1a-n **8** **3** **9a-n**

General synthesis procedure 2 (GP2)

A reaction vial was charged with the sulfonyl acetates **1a-n** (4.5 mmol, 1 equiv.), acetic anhydride (**8**) (90 mmol, 20 equiv.), triethyl orthoformate (**3**) (90 mmol, 20 equiv.) and (1.35 mmol, 0.3 equiv.) zinc chloride. The reaction was stirred under argon atmosphere for 2-4 d at 140 °C in an aluminum heat block under reflux. After 24 h additional 10 equiv. (45 mmol) acetic anhydride (**8**) and 10 equiv. (45 mmol) triethyl orthoformate (**3**) were added and stirred under the same conditions until complete consumption of the starting material. After cooling to room temperature 50 mL water and 50 mL of saturated sodium carbonate solution were added. The mixture was stirred until no more gas development could be observed. The phases were separated, and the aqueous phase was washed three times with 60 mL of ethyl acetate. The organic phases were combined, dried with sodium sulphate, and filtered. The crude product was concentrated in vacuo and purified by flash column chromatography (cyclohexane/ethyl acetate) to afford the desired product.

2.2.3 Formation of the Thiouracils (GP3)

Scheme S4. Preparation of the thiouracils **10a-m, 11a-m**

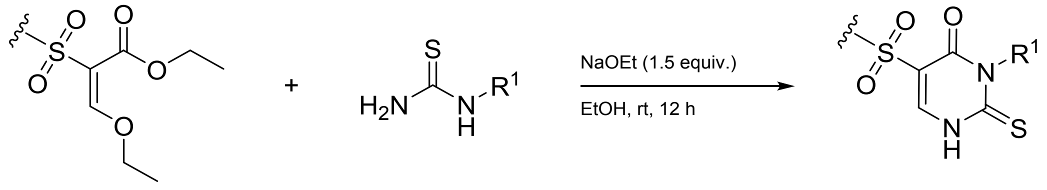

9a-n **10a-c** **11a-n** **12a-n** **13**

A reaction vial was charged with the enol ether **9a-n** (0.25 mmol, 1 equiv.), thiourea (**10a**), *N*-methylthiourea (**10b**) or *N*-phenylthiourea (**10c**) (0.28 mmol, 1.1 equiv.), 1.4 mL dry ethanol and 140 µL (0.38 mmol, 1.5 equiv.) of a dry 21 wt% sodium ethanolate solution. The reaction was stirred at room temperature for 12 h under argon atmosphere. After completion the reaction was concentrated in vacuo. The crude product was solved in 5 mL water. The solution was then filtered

through a glass drip (pore size: 10-16 µM). The filtrate was acidified with concentrated hydrochloric acid (pH = 1) and filtered through a glass drip (pore size: 10-16 µM). The precipitate was washed with 0.1 M hydrochloric acid and dried to afford the desired uracil derivative **11a-n** resp. **12a-n** or **13**.

2.3 Optimization of Reaction Conditions and scale-up

2.3.1 Optimization of the Enol ether synthesis

Table S1. Optimization of the formation of the enol ether **9a**.

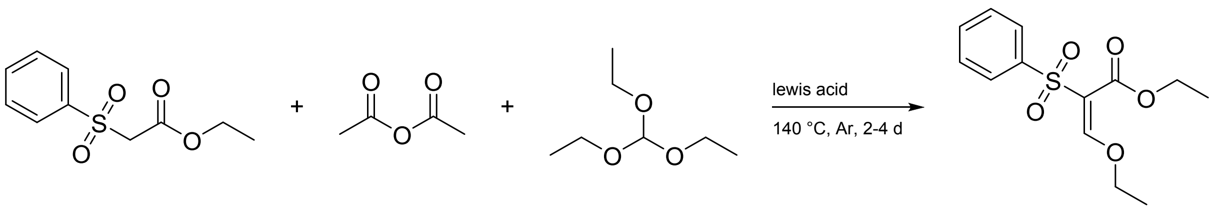

8 (equiv.)	3 (equiv.)	stepwise reagent addition	temp (°C)	lewis acid	yield (%)
**reagent equivalents**					
5	10	-	120	-	40
10	5	-	120	-	38
10	10	-	120	-	43
20	20	-	120	-	46
**30**	**30**	**-**	**120**	**-**	**47**
**reagent addition**					
**30**	**30**	**Yes (20,10)**	**120**	**-**	**61**
**reaction temperature**					
30	30	Yes	100	-	42
30	30	Yes	110	-	55
30	30	Yes	120	-	61
30	30	Yes	130	-	66
**30**	**30**	**Yes**	**140**	**-**	**69**
30	30	Yes	150	-	68
**lewis acid**					
**30**	**30**	**Yes**	**140**	**$ZnCl_2$**	**80**

2.3.2 Scale-up of 5a at a 10 mmol scale

Scheme S5. Scale-up synthesis of **5a**

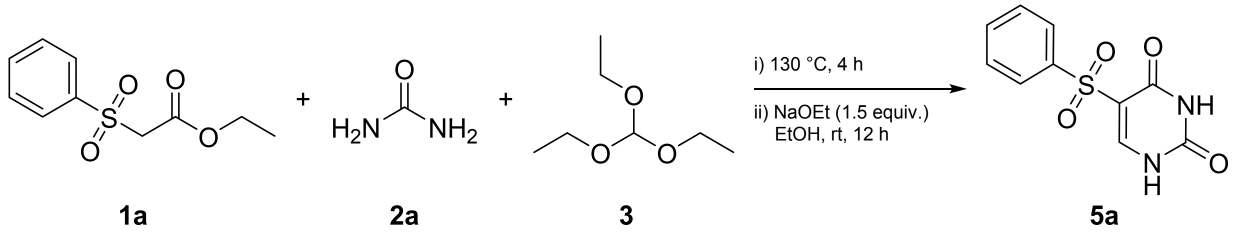

A reaction vial was charged with ethyl 2-(benzenesulfonyl)acetate (**1a**, 2.28 g, 10.0 mmol, 1 equiv.), urea (**2a**, 1.80 g, 30.0 mmol, 3 equiv.) and triethyl orthoformate (**3**, 3.33 mL, 20.0 mmol, 2 equiv.). The reaction was heated for 5 min at 130 °C in an aluminum bead bath. The vial was then opened, to allow formed ethanol do evaporate. The open reaction was heated for additional 4 h at 130 °C. After cooling to room temperature 56 mL dry ethanol and 5.59 mL (15.00 mmol, 1.5 equiv.) of a dry 21 wt% sodium ethanolate solution was added under argon atmosphere. The vial was closed and stirred for 12 h at room temperature. After completion the reaction was concentrated in vacuo. The crude product was solved in 200 mL water. The solution was then filtered through a glass drip (pore size: 10-16 µM). The filtrate was acidified with concentrated hydrochloric acid (pH = 1) and filtered through a glass drip (pore size: 10-16 µM). The precipitate was washed with 0.1 M hydrochloric acid and dried to afford **5a** (2.10 g, 81 %).

2.3.3 Scale-up of 11a at a 10 mmol scale

Scheme S6. Scale-up synthesis of **11a**

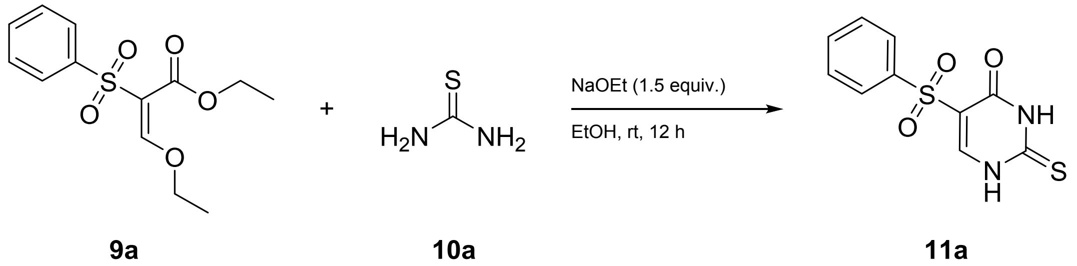

A reaction vial was charged with ethyl-(2*E*)-2-(benzenesulfonyl)-3-ethoxyprop-2-enoate (**9a**, 2.84 g, 10.0 mmol, 1 equiv.), thiourea (**10a**, 838 mg, 11.0 mmol, 1.1 equiv.), 56 mL dry ethanol and 5.59 mL (15.0 mmol, 1.5 equiv.) of a dry 21 wt% sodium ethanolate solution. The reaction was stirred at room temperature for 12 h under argon atmosphere. After completion the reaction was concentrated in vacuo. The crude product was solved in 200 mL water. The solution was then filtered through a glass drip (pore size: 10-16 µM). The filtrate was acidified with concentrated hydrochloric acid (pH = 1) and filtered through a glass drip (pore size: 10-16 µM). The precipitate was washed with 0.1 M hydrochloric acid and dried to afford **11a** (2.36 g, 88 %).

2.3.4 Synthesis of the condensation intermediate 4a

Scheme S7. Synthesis of **4a**

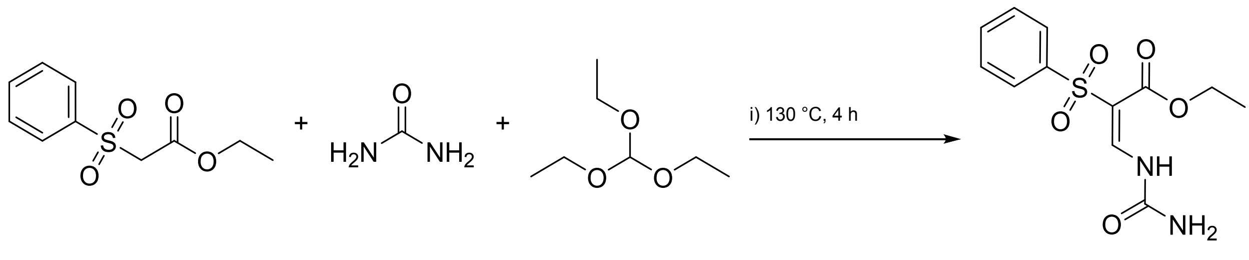

1a **2a** **3** **4a**

A reaction vial was charged with ethyl 2-(benzenesulfonyl)acetate (**1a**, 50 mg, 0.22 mmol, 1 equiv.), urea (**2a**) (39 mg, 0.66 mmol, 3 equiv.) and triethyl orthoformate (**3**) (73 µL, 0.44 mmol, 2 equiv.). The vial was equipped with a magnetic stirring bar and sealed. The reaction was heated for 5 min at 130 °C in an aluminum heat block. The vial was then opened, to allow formed ethanol do evaporate. The open reaction was heated for additional 4 h at 130 °C in an aluminum heat block. The crude product was solved in 2 mL water and 0.5 mL Methanol. The solution was then filtered through a glass drip (pore size: 10-16 µM) to give the title compound **4a** as white solid (59.4 mg, 91 %). **^{1}H-NMR** (400 MHz, DMSO-d$_6$): δ 10.33 (d, J = 13.0 Hz, 1H), 8.69 (d, J = 13.0 Hz, 1H), 7.86 (dd, J = 8.3, 1.4 Hz, 2H), 7.72 – 7.54 (m, 4H), 7.42 (s, 1H), 4.07 (q, J = 7.1 Hz, 2H), 1.02 (t, J = 7.1 Hz, 3H) ppm. **^{13}C{^{1}H}-NMR** (101 MHz, DMSO-d$_6$): δ 163.0, 152.4, 148.87, 141.6, 132.9, 128.9 (2C), 127.6 (2C), 105.5, 60.7, 13.7 ppm. **HR-MS (ESI)** m/z calc. for $C_{12}H_{15}N_2O_5S^+$ [M+H]$^+$: 299.0697, found: 299.0705.

Scheme S8. By-product of the synthesis of **4a**

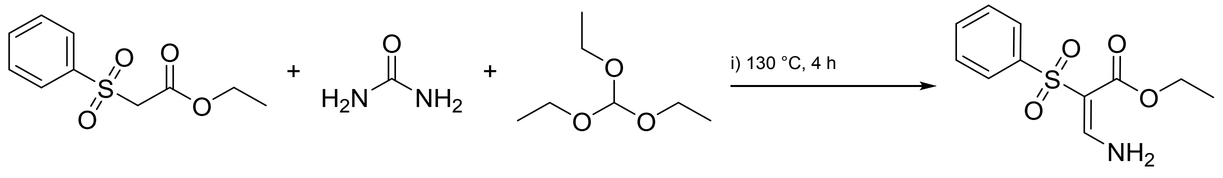

1a **2a** **3** **S4**

^{1}H-NMR (300 MHz, DMSO-d$_6$): δ 8.60 (bs, 1H), 8.36 (bs, d, J = 16.6 Hz, 1H), 8.00 (dd, J = 16.4, 8.8 Hz, 1H), 7.83 – 7.78 (m, 2H), 7.60 – 7.50 (m, 3H), 3.98 (q, J = 7.1 Hz, 2H), 1.00 (t, J = 7.1 Hz, 3H) ppm. **^{13}C{^{1}H}-NMR** (75 MHz, DMSO-d$_6$): δ 163.8, 156.0, 143.2, 132.1, 128.6 (2C), 127.2 (2C), 98.4, 59.3, 13.9 ppm. **HR-MS (ESI)** m/z calc. for $C_{11}H_{14}NO_4S^+$ [M+H]$^+$: 256.0639, found: 256.0638.

2.3.5 Synthesis of Guanidine derivatives (15a-c)

5-(Benzenesulfonyl)-2-imino-1,2,3,4-tetrahydropyrimidin-4-one (15a)

Scheme S9. Synthesis of **15a**

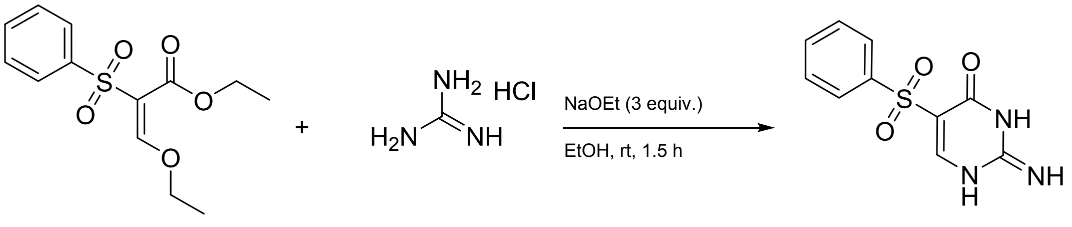

9a **14a** **15a**

A reaction vial was charged with ethyl-(2*E*)-2-(benzenesulfonyl)-3-ethoxyprop-2-enoate (**9a**, 71 mg, 0.25 mmol, 1 equiv.), guanidine hydrochlorid (**14a**, 26 mg, 0.28 mmol, 1.1 equiv.), 1.4 mL dry ethanol and 280 µL (0.75 mmol, 3 equiv.) of a dry 21 wt% sodium ethanolate solution. The reaction was stirred at room temperature for 12 h under argon atmosphere. After completion the reaction was concentrated in vacuo. The crude product was purified by column chromatography (dichloromethane/methanol/formic acid = 89:10:1). The formic acid salt of compound **15a** was obtained as a white solid (38 mg, 51 %). **^{1}H-NMR** (400 MHz, DMSO-d$_6$): δ 8.34 (s, 1H), 8.15 (s, 1H), 7.95 – 7.87 (m, 2H), 7.68 – 7.59 (m, 1H), 7.55 (dd, *J* = 8.3, 6.7 Hz, 2H) ppm. **^{13}C{^{1}H}-NMR** (101 MHz, DMSO-d$_6$): δ 163.3, 160.4, 159.2, 157.5, 141.7, 132.8, 128.8 (2C), 127.39 (2C), 112.80 ppm. **HR-MS (ESI)** m/z calc. for $C_{10}H_{10}N_3O_3S^+$ [M+H]$^+$: 252.0438, found: 252.0435.

5-(Benzenesulfonyl)-2-imino-3-phenyl-1,2,3,4-tetrahydropyrimidin-4-one (15b)

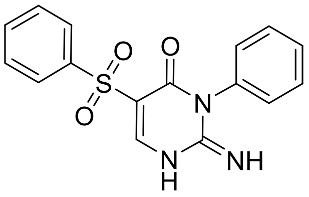

The procedure for **15a** was followed**,** with the exception of the use of 1-phenylguanidine (**14b**, 37 mg, 0.28 mmol, 1.1 equiv.) and a reduced volume of dry 21 wt% sodium ethanolate solution (140 µL, 0.38 mmol, 1.5 equiv.). The crude product was purified by column chromatography (dichloromethane/methanol = 97:3). Compound **15b** was obtained as a white solid (61 mg, 75 %). **^{1}H-NMR** (400 MHz, DMSO-d$_6$): δ 11.44 (br s, 1H), 9.48 (br s, 1H), 8.47 (s, 1H), 7.98 – 7.93 (m, 2H), 7.70 – 7.64 (m, 1H), 7.58 (dd, *J* = 8.3, 6.7 Hz, 2H), 7.55 – 7.50 (m, 2H), 7.36 (dd, *J* = 8.5, 7.3 Hz, 2H), 7.19 – 7.13 (m, 1H) ppm. **^{13}C{^{1}H}-NMR** (101 MHz, DMSO-d$_6$): δ 159.6, 157.4, 155.8, 141.2, 136.9, 133.1, 128.9 (2C), 128.9 (2C), 127.6 (2C), 124.7, 122.0 (2C), 115.6 ppm. **HR-MS (ESI)** m/z calc. for $C_{16}H_{14}N_3O_3S^+$ [M+H]$^+$: 328.0750, found: 328.0764.

(2*S*)-5-[5-(Benzenesulfonyl)-2-imino-6-oxo-1,2,3,6-tetrahydropyrimidin-1-yl]-2-{[(tert-butoxy)-carbonyl]amino}pentanoic acid (15c)

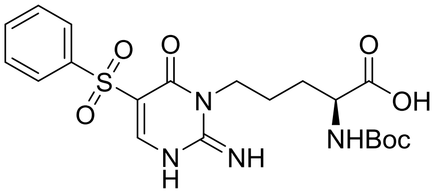

The procedure for **15a** was followed**,** with the exception of the use of (2*S*)-2-{[(tert-butoxy)carbonyl]amino}-5-carbamimidamidopentanoic acid (**14c**, 76 mg, 0.28 mmol, 1.1 equiv.) and a reduced volume of dry 21 wt% sodium ethanolate solution (204 µL, 0.55 mmol, 2.2 equiv.). The crude product was purified by column chromatography (dichloromethane/methanol/formic acid = 94.5:4.5:0.5). Compound **15c** was obtained as a white solid (67 mg, 57 %). ^{1}H-NMR (300 MHz, DMSO-d_6): *δ* = 8.32 (s, 1H), 7.93 – 7.88 (m, 2H), 7.66 – 7.49 (m, 3H), 7.03 (br d, *J* = 7.9 Hz, 1H), 3.82 – 3.79 (m, 1H), 3.72 – 3.66 (m, 2H), 1.70 – 1.41 (m, 4H), 1.35 (s, 9H) ppm. **^{13}C{^{1}H}-NMR** (76 MHz, DMSO-d_6): *δ* 173.9, 158.6, 158.4, 156.8, 155.6, 141.6, 132.9, 128.8 (2C), 127.5 (2C), 111.6, 78.1, 53.3, 40.4, 28.3, 27.9, 23.3 ppm. **HR-MS (ESI)** m/z calc. for $C_{20}H_{25}N_3O_7S^+$ $[M-H]^-$: 465.1449, found: 465.1436.

2.3.6 Synthesis of 5-(Benzenesulfonyl)-2-methyl-1,4-dihydropyrimidin-4-one (17)

Scheme S10. Synthesis of **17**

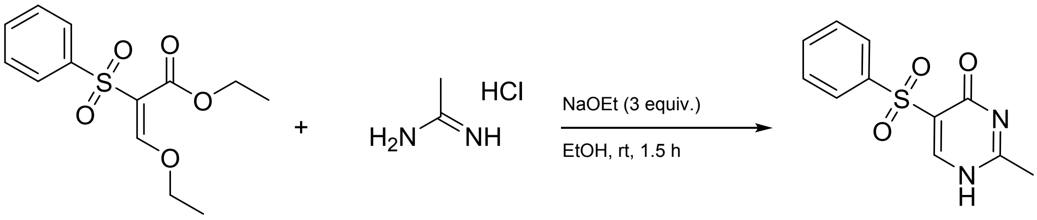

9a **16** **17**

A reaction vial was charged with ethyl-(2*E*)-2-(benzenesulfonyl)-3-ethoxyprop-2-enoate (**9a**, 71 mg, 0.25 mmol, 1 equiv.), acetamidine hydrochlorid (**16**, 26 mg, 0.28 mmol, 1.1 equiv.), 1.4 mL dry ethanol and 280 µL (0.75 mmol, 3 equiv.) of a dry 21 wt% sodium ethanolate solution. The reaction was stirred at room temperature for 12 h under argon atmosphere. After completion the reaction was concentrated in vacuo. The crude products were purified by column chromatography (dichloromethane/methanol = 95:5). The title compound **17** was obtained as a white solid. (40 mg, 64 %). **^{1}H-NMR** (400 MHz, DMSO-d_6): *δ* 8.60 (s, 1H), 8.00 – 7.96 (m, 2H), 7.72 – 7.65 (m, 1H), 7.60 (dd, *J* = 8.4, 7.0 Hz, 2H), 2.35 (s, 3H) ppm. **^{13}C{^{1}H}-NMR** (101 MHz, DMSO-d_6): *δ* 166.3, 157.2, 156.8, 139.9,

133.7, 129.0 (2C), 128.1 (2C), 123.5, 21.7 ppm. **HR-MS (ESI)** m/z calc. for $C_{11}H_{11}N_2O_3S^+$ $[M+H]^+$: 251.0485, found: 251.0488.

2.3.7 Synthesis of 5-(Benzenesulfonyl)-2-(benzylsulfanyl)-1,4-dihydropyrimidin-4-one (19)

Scheme S11. Synthesis of **19**

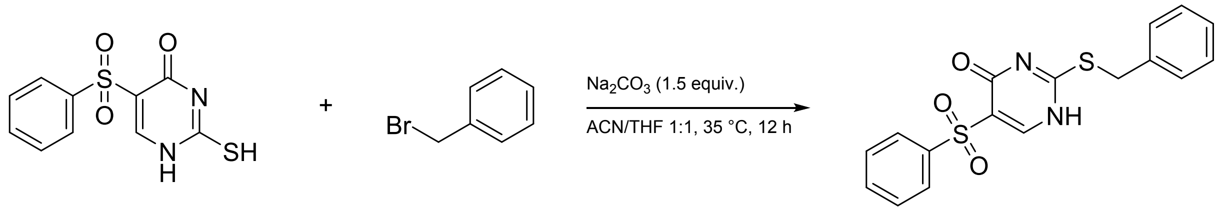

11a **18** **19**

A reaction vial was charged with 5-(benzenesulfonyl)-2-sulfanylidene-1,2,3,4-tetrahydropyrimidin-4-one (**11a**, 29 mg, 0.1 mmol, 1.0 equiv.) and benzyl bromide (**18**, 12,2 µL, 0.1 mmol, 1.0 equiv.) solved in 15 mL of a 1:1 mixture of acetonitrile and tetrahydrofuran. The reaction was stirred for 12 h at 35 °C in an aluminum bead bath. After completion the reaction was concentrated in vacuo. The crude product was purified by column chromatography (dichloromethane/methanol = 95:5). The title compound **19** was obtained as a white solid. (28 mg, 78 %). **^{1}H-NMR** (400 MHz, DMSO-d_6): *δ* 8.55 (s, 1H), 7.99 – 7.95 (m, 2H), 7.71 – 7.66 (m, 1H), 7.62 – 7.57 (m, 2H), 7.43 – 7.39 (m, 2H), 7.34 – 7.29 (m, 2H), 7.29 – 7.24 (m, 1H), 4.44 (s, 2H) ppm. **^{13}C{^{1}H}-NMR** (101 MHz, DMSO-d_6): *δ* 169.1, 158.1, 155.3, 140.3, 136.6, 133.5, 129.1 (2C), 128.9 (2C), 128.6 (2C), 128.0 (2C), 127.5, 121.0, 34.0 ppm. **HR-MS (ESI)** m/z calc. for $C_{17}H_{15}N_2O_3S_2^+$ $[M+H]^+$: 359.0519, found: 359.0514.

2.4 Yields and Characterizations

2.4.1 Starting materials 1a-n (sulfonyl acetates)

Ethyl 2-(benzenesulfonyl)acetate (1a)

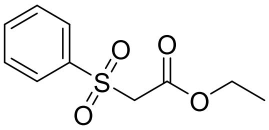

Following the procedure described in Chapter 2 (Preceding Synthesis) the title compound **1a** was obtained and purified by flash column chromatography (cyclohexane/ethyl acetate = 4:1) as a yellow liquid (2.72 g, 95 %). **^{1}H-NMR** (400 MHz, DMSO-d$_6$): δ 7.97 – 7.89 (m, 2H), 7.82 – 7.73 (m, 1H), 7.72 – 7.62 (m, 2H), 4.62 (s, 2H), 4.02 (q, *J* = 7.11 Hz, 2H), 1.04 (t, *J* = 7.10 Hz, 3H) ppm. **^{13}C{^{1}H}-NMR** (101 MHz, DMSO-d$_6$): δ 162.6, 139.0, 134.2, 129.3 (2C), 128.1 (2C), 61.5, 59.8, 13.6 ppm. **HR-MS (ESI)** m/z calc. for $C_{10}H_{13}O_4S^+$ [M+H]$^+$: 229.0530, found: 229.0534.

Ethyl 2-(4-fluorobenzenesulfonyl)acetate (1b)

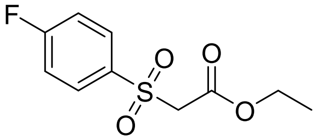

Following the procedure described in Chapter 2 (Preceding Synthesis) the title compound **1b** was obtained and purified by flash column chromatography (cyclohexane/ethyl acetate = 9:1) as a yellow liquid (1.05 g, 43 %). **^{1}H-NMR** (300 MHz, CDCl$_3$): δ 8.08 – 7.90 (m, 2H), 7.31 – 7.19 (m, 2H), 4.15 (q, *J* = 7.2 Hz, 2H), 4.11 (s, 2H), 1.21 (t, *J* = 7.1 Hz, 3H) ppm. **^{13}C{^{1}H}-NMR** (75 MHz, CDCl$_3$): δ 166.3 (d, *J* = 257.3 Hz), 162.4, 134.9 (d, *J* = 3.4 Hz), 131.8 (d, *J* = 9.8 Hz, 2C), 116.7 (d, *J* = 22.8 Hz, 2C), 62.6, 61.1, 14.0 ppm. **^{19}F-NMR** (282 MHz, CDCl$_3$): δ -102.37 (td, J = 1.84, 4.3 Hz) ppm. **LR-MS (EI)** m/z calc. for $C_{10}H_{11}FO_4S^{\bullet+}$ ($M^{+\bullet}$): 246.0, found: 246.1.

Ethyl 2-(4-chlorobenzenesulfonyl)acetate (1c)

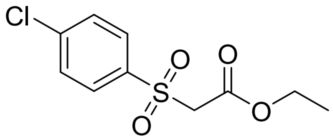

Following the procedure described in Chapter 2 (Preceding Synthesis) the title compound **1c** was obtained and purified by flash column chromatography (cyclohexane/ethyl acetate = 9:1) as a yellow liquid (1.34 g, 51 %). **^{1}H-NMR** (400 MHz, CDCl$_3$): δ 7.89 (d, *J* = 8.6 Hz, 2H), 7.55 (d, *J* = 8.6 Hz, 2H), 4.15 (q, *J* = 7.1 Hz, 2H), 4.11 (s, 2H), 1.21 (t, *J* = 7.1 Hz, 3H) ppm. **^{13}C{^{1}H}-NMR** (101 MHz, CDCl$_3$): δ 162.4,

141.3, 137.3, 130.3 (2C), 129.7 (2C), 62.7, 61.1, 14.0 ppm. **LR-MS (EI)** m/z calc. for $C_{10}H_{11}ClO_4S^{•+}$ ($M^{+•}$): 262.0, found: 262.0.

Ethyl 2-(4-bromobenzenesulfonyl)acetate (1d)

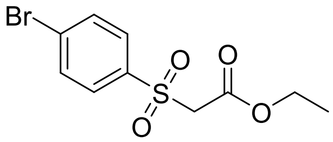

Following the procedure described in Chapter 2 (Preceding Synthesis) the title compound **1d** was obtained and purified by flash column chromatography (cyclohexane/ethyl acetate = 9:1) as a yellow liquid (1.63 g, 53 %). **^{1}H-NMR** (400 MHz, $CDCl_3$): δ 7.82 (d, J = 8.7 Hz, 2H), 7.75 – 7.71 (m, 2H), 4.17 (q, J = 7.1 Hz, 2H), 4.10 (s, 2H), 1.23 (t, J = 7.1 Hz, 3H) ppm. **^{13}C{^{1}H}-NMR** (101 MHz, $CDCl_3$): δ 162.2, 137.7, 132.5 (2C), 130.2 (2C), 129.8, 62.5, 60.9, 13.9 ppm. **LR-MS (EI)** m/z calc. for $C_{10}H_{11}BrO_4S^{•+}$ ($M^{+•}$): 308.0, found: 308.0.

Ethyl 2-(4-iodobenzenesulfonyl)acetate (1e)

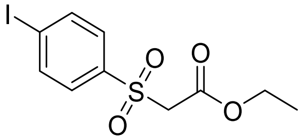

Following the procedure described in Chapter 2 (Preceding Synthesis) the title compound **1e** was obtained and purified by flash column chromatography (cyclohexane/ethyl acetate = 9:1) as a yellow liquid (1.91 g, 54 %). **^{1}H-NMR** (400 MHz, $CDCl_3$): δ 7.97 – 7.93 (m, 2H), 7.67 – 7.63 (m, 2H), 4.17 (q, J = 7.1 Hz, 2H), 4.10 (s, 2H), 1.23 (t, J = 7.1 Hz, 3H) ppm. **^{13}C{^{1}H}-NMR** (101 MHz, $CDCl_3$): δ 162.4, 138.7 (2C), 130.1 (2C), 102.7, 62.7, 61.0, 14.1 ppm. **LR-MS (EI)** m/z calc. for $C_{10}H_{11}IO_4S^{•+}$ ($M^{+•}$): 353.9, found: 354.0.

Ethyl 2-(3-chlorobenzenesulfonyl)acetate (1f)

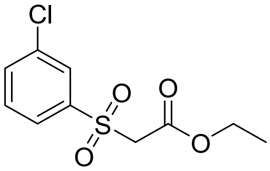

Following the procedure described in Chapter 2 (Preceding Synthesis) the title compound **1f** was obtained and purified by flash column chromatography (cyclohexane/ethyl acetate = 9:1) as a yellow liquid (1.09 g, 41 %). **^{1}H-NMR** (400 MHz, $CDCl_3$): δ 7.94 (t, J = 1.9 Hz, 1H), 7.87 – 7.82 (m, 1H), 7.68 – 7.63 (m, 1H), 7.53 (t, J = 7.9 Hz, 1H), 4.16 (q, J = 7.1 Hz, 2H), 4.12 (s, 2H), 1.21 (t, J = 7.1 Hz, 3H). **^{13}C{^{1}H}-**

NMR (101 MHz, $CDCl_3$): *δ* 162.1, 151.3, 144.3, 130.4 (2C), 124.5 (2C), 62.9, 60.7, 14.0 ppm. **LR-MS (EI)** m/z calc. for $C_{10}H_{11}ClO_4S^{\bullet+}$ ($M^{+\bullet}$): 262.0, found: 262.0.

Ethyl 2-(2-chlorobenzenesulfonyl)acetate (1g)

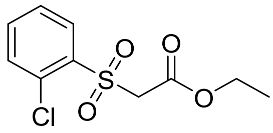

Following the procedure described in Chapter 2 (Preceding Synthesis) the title compound **1g** was obtained and purified by flash column chromatography (cyclohexane/ethyl acetate = 9:1) as a yellow liquid (1.26 g, 48 %). **^{1}H-NMR** (400 MHz, $CDCl_3$): *δ* 8.16 – 8.10 (m, 0H), 7.65 – 7.54 (m, 1H), 7.53 – 7.44 (m, 1H), 4.44 (s, 1H), 4.10 (q, *J* = 7.2 Hz, 1H), 1.13 (td, *J* = 7.1, 0.7 Hz, 2H) ppm.**^{13}C{^{1}H}-NMR** (101 MHz, $CDCl_3$): *δ* 162.2, 136.4, 135.3, 132.8, 132.2, 132.0 127.5, 62.5, 58.9, 13.9 ppm. **LR-MS (EI)** m/z calc. for $C_{10}H_{11}ClO_4S^{\bullet+}$ ($M^{+\bullet}$): 262.0, found: 262.0.

Ethyl 2-(4-nitrobenzenesulfonyl)acetate (1h)

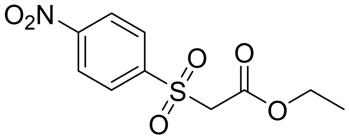

Following the procedure described in Chapter 2 (Preceding Synthesis) the title compound **1h** was obtained and purified by flash column chromatography (cyclohexane/ethyl acetate = 7:3) as a yellow liquid (0.78 g, 28 %). **^{1}H-NMR** (400 MHz, $CDCl_3$): *δ* 8.45 – 8.40 (m, 2H), 8.19 – 8.14 (m, 2H), 4.17 (q, *J* = 6.7 Hz, 4H), 1.23 (t, *J* = 7.2 Hz, 4H) ppm. **^{13}C{^{1}H}-NMR** (101 MHz, $CDCl_3$): *δ* 162.2, 136.4, 135.3, 132.8, 132.2, 132.0, 127.5, 62.5, 58.9, 13.9 ppm. **LR-MS (EI)** m/z calc. for $C_{10}H_{11}NO_6S^{\bullet+}$ ($M^{+\bullet}$): 273.0, found: 273.1.

Ethyl 2-[4-(trifluoromethyl)benzenesulfonyl]acetate (1i)

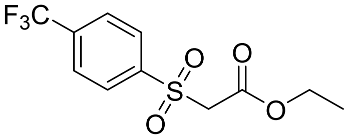

Following the procedure described in Chapter 2 (Preceding Synthesis) the title compound **1i** was obtained and purified by flash column chromatography (cyclohexane/ethyl acetate = 9:1) as a yellow liquid (1.55 g, 52 %). **^{1}H-NMR** (400 MHz, $CDCl_3$): *δ* 8.14 – 8.07 (m, 2H), 7.93 – 7.81 (m, 2H), 4.16 (d, *J* = 7.3 Hz, 4H), 1.21 (t, *J* = 7.2 Hz, 3H) ppm. **^{13}C{^{1}H}-NMR** (101 MHz, $CDCl_3$): *δ* 162.2, 142.3, 136.1 (q, *J* = 33.2 Hz), 129.5, 126.5 (q, *J* = 3.8 Hz), 123.2 (q, *J* = 273.2 Hz), 62.8, 60.9, 14.0 ppm. **^{19}F-NMR** (376 MHz, $CDCl_3$): *δ* -63.32 (s) ppm. **LR-MS (EI)** m/z calc. for $C_{11}H_{11}F_3O_4S^{\bullet+}$ ($M^{+\bullet}$): 297.0, found: 297.0.

Ethyl 2-(4-methoxybenzenesulfonyl)acetate (1j)

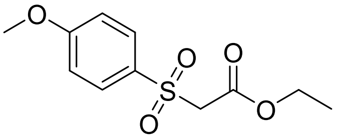

Following the procedure described in Chapter 2 (Preceding Synthesis) the title compound **1j** was obtained and purified by flash column chromatography (cyclohexane/ethyl acetate = 4:1) as a yellow liquid (0.37 g, 14 %). **^{1}H-NMR** (400 MHz, $CDCl_3$): *δ* 7.88 – 7.80 (m, 2H), 7.21 – 7.13 (m, 2H), 4.52 (s, 2H), 4.03 (q, *J* = 7.1 Hz, 2H), 3.86 (s, 3H), 1.07 (t, *J* = 7.1 Hz, 3H) ppm. **^{13}C{^{1}H}-NMR** (101 MHz, $CDCl_3$): *δ* 163.6, 162.8, 130.5 (3C), 114.4 (2C), 61.4, 60.2, 55.8, 13.7 ppm. **LR-MS (EI)** m/z calc. for $C_{11}H_{14}O_5S^{\bullet+}$ ($M^{+\bullet}$): 258.1, found: 258.1.

Ethyl 2-(4-methylbenzenesulfonyl)acetate (1k)

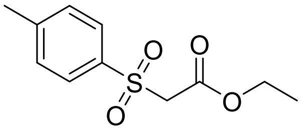

Following the procedure described in Chapter 2 (Preceding Synthesis) the title compound **1k** was obtained and purified by flash column chromatography (cyclohexane/ethyl acetate = 9:1) as a yellow liquid (0.86 g, 36 %). **^{1}H-NMR** (400 MHz, $CDCl_3$): *δ* 7.85 – 7.78 (m, 2H), 7.39 – 7.33 (m, 2H), 4.14 (q, *J* = 7.1 Hz, 2H), 4.08 (s, 2H), 2.45 (s, 3H), 1.19 (t, *J* = 7.1 Hz, 3H) ppm. **^{13}C{^{1}H}-NMR** (101 MHz, $CDCl_3$): *δ* 162.6, 145.5, 135.9, 129.9 (C2), 128.7 (C2), 62.4, 61.2, 21.8, 14.0 ppm. **LR-MS (EI)** m/z calc. for $C_{11}H_{14}O_4S^{\bullet+}$ ($M^{+\bullet}$): 242.1, found: 242.1.

Ethyl 2-(pyridine-3-sulfonyl)acetate (1l)

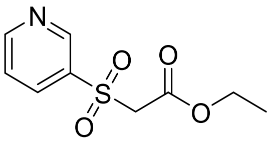

Following the procedure described in Chapter 2 (Preceding Synthesis) the title compound **1l** was obtained and purified by flash column chromatography (cyclohexane/ethyl acetate = 1:1) as a yellow liquid (0.86 g, 36 %). **^{1}H-NMR** (400 MHz, $CDCl_3$): *δ* 9.07 (dd, *J* = 2.4, 0.9 Hz, 1H), 8.93 (dd, *J* = 4.9, 1.6 Hz, 1H), 8.38 – 8.29 (m, 1H), 7.78 – 7.66 (m, 1H), 4.80 (s, 2H), 4.05 (q, *J* = 7.1 Hz, 2H), 1.06 (t, *J* = 7.1 Hz, 3H) ppm. **^{13}C{^{1}H}-NMR** (101 MHz, $CDCl_3$): *δ* 162.6, 154.5, 148.8, 136.5, 135.4, 124.2, 61.7, 59.7, 13.7 ppm. **LR-MS (EI)** m/z calc. for $C_9H_{11}NO_4S^{\bullet+}$ ($M^{+\bullet}$): 229.0, found: 229.1.

Ethyl 2-(4-{[(tert-butoxy)carbonyl]amino}benzenesulfonyl)acetate (1m)

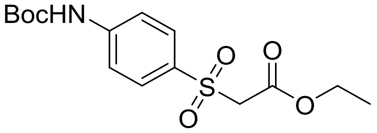

Following the procedure described in Chapter 2 (Preceding Synthesis) the title compound **1m** was obtained and purified by flash column chromatography (cyclohexane/ethyl acetate = 4:1) as a yellow liquid (0.88 g, 30 %). **^{1}H-NMR** (400 MHz, $CDCl_3$): δ 9.93 (br s, 1H), 7.81 – 7.76 (m, 2H), 7.71 – 7.66 (m, 2H), 4.49 (s, 2H), 4.03 (q, *J* = 7.1 Hz, 2H), 1.49 (s, 9H), 1.07 (t, J = 7.1 Hz, 3H) ppm. **^{13}C{^{1}H}-NMR** (101 MHz, $CDCl_3$): δ 162.7, 152.5, 144.8, 131.3, 129.4 (2C), 117.4 (2C), 80.1, 61.4, 60.2, 28.0 (3C), 13.7 ppm. **HR-MS (ESI)** m/z calc. for $C_{15}H_{21}NNaO_6S^+$ [M+Na]$^+$: 366.0982, found: 366.0997.

Ethyl 2-(pyridine-2-sulfonyl)acetate (1n)

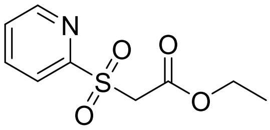

Following the procedure described in Chapter 2 (Preceding Synthesis) the title compound **1n** was obtained and purified by flash column chromatography (cyclohexane/ethyl acetate = 1:1) as a yellow liquid (0.37 g, 16 %). **^{1}H-NMR** (300 MHz, DMSO-d_6): δ 8.82 – 8.79 (m, 1H), 8.18 (td, *J* = 7.7, 1.7 Hz, 1H), 8.06 (dt, *J* = 7.9, 1.1 Hz, 1H), 7.81 – 7.75 (m, 1H), 4.72 (s, 2H), 4.00 (q, *J* = 7.1 Hz, 2H), 1.01 (t, *J* = 7.1 Hz, 3H) ppm. **^{13}C{^{1}H}-NMR** (75 MHz, DMSO-d_6): δ 162.5, 156.2, 150.3, 139.1, 128.3, 122.0, 61.5, 56.1, 13.6 ppm. **LR-MS (EI)** m/z calc. for $C_9H_{11}NO_4S^{\bullet+}$ ($M^{+\bullet}$): 229.0, found: 229.1.

2.4.2 Uracils 5a-r, 6a-r, 7

5-(Benzenesulfonyl)-1,2,3,4-tetrahydropyrimidine-2,4-dione (5a)

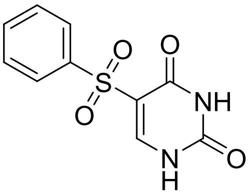

Following **GP1** the title compound **5a** was obtained as a white solid (52.9 mg, 84 %). **^{1}H-NMR** (400 MHz, DMSO-d$_6$): *δ* 11.48 (br s, 1H), 8.26 (s, 1H), 7.99 – 7.91 (m, 2H), 7.72 – 7.63 (m, 1H), 7.59 (dd, *J* = 8.37, 6.93 Hz, 2H) ppm. **^{13}C{^{1}H}-NMR** (101 MHz, DMSO-d$_6$): *δ* 158.6, 151.1, 149.3, 140.6, 133.4, 128.9 (2C), 127.7 (2C), 112.2 ppm. **HR-MS (ESI)** m/z calc. for $C_{10}H_9N_2O_4S^+$ [M+H]$^+$: 253.0278, found: 253.0271.

5-(4-Fluorobenzenesulfonyl)-1,2,3,4-tetrahydropyrimidine-2,4-dione (5b)

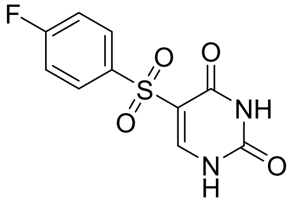

Following **GP1** the title compound **5b** was obtained as a white solid (59.7 mg, 88 %). **^{1}H-NMR** (400 MHz, DMSO-d$_6$): *δ* 11.99 (br s, 1H), 11.59 (br s, 1H), 8.25 (s, 1H), 8.06 – 7.99 (m, 2H), 7.44 (t, *J* = 8.9 Hz, 2H) ppm. **^{13}C{^{1}H}-NMR** (101 MHz, DMSO-d$_6$): *δ* 164.8 (d, *J* = 252.3 Hz), 158.5, 150.5, 148.7, 136.8 (d, *J* = 2.9 Hz), 131.1 (d, *J* = 9.9 Hz, 2C), 116.1 (d, *J* = 23.0 Hz, 2C), 112.4 ppm. **^{19}F-NMR** (282 MHz, DMSO-d$_6$): *δ* -105.33 (tt, *J* = 9.3, 5.4 Hz) ppm. **HR-MS (ESI)** m/z calc. for $C_{10}H_8FN_2O_4S^+$ [M+H]$^+$: 271.0184, found: 271.0197.

5-(4-Chlorobenzenesulfonyl)-1,2,3,4-tetrahydropyrimidine-2,4-dione (5c)

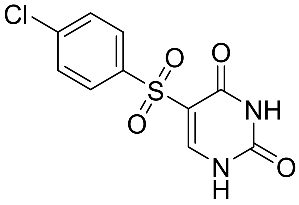

Following **GP1** the title compound **5c** was obtained as a white solid (67.8 mg, 95 %). **^{1}H-NMR** (400 MHz, DMSO-d$_6$): *δ* 8.26 (s, 1H), 7.96 (dt, *J* = 8.7, 2.0 Hz, 2H), 7.68 (dt, *J* = 8.7, 2.0 Hz, 2H)

ppm. **^{13}C{^{1}H}-NMR** (101 MHz, DMSO-d_6): δ 158.5, 150.7, 149.1, 139.3, 138.5, 129.9 (2C), 129.1 (2C), 112.0 ppm. **HR-MS (ESI)** m/z calc. for $C_{10}H_6ClN_2O_4S$ [M-H]$^-$: 284.9742, found: 284.9732.

5-(4-Bromobenzenesulfonyl)-1,2,3,4-tetrahydropyrimidine-2,4-dione (5d)

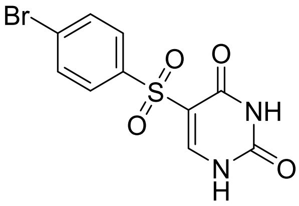

Following **GP1** the title compound **5d** was obtained as a white solid (54.7 mg, 66 %). **^{1}H-NMR** (400 MHz, DMSO-d_6): δ 12.02 (s, 1H), 11.60 (s, 1H), 8.26 (s, 1H), 7.88 (d, J = 8.6 Hz, 2H), 7.82 (d, J = 8.6 Hz, 2H) ppm. **^{13}C{^{1}H}-NMR** (101 MHz, DMSO-d_6): δ 158.5, 150.5, 149.0, 139.7, 132.0 (2C), 129.9 (2C), 127.6, 112.0 ppm. **HR-MS (ESI)** m/z calc. for $C_{10}H_6BrN_2O_4S^-$ [M-H]$^-$: 330.9216, found: 330.9215.

5-(4-Iodobenzenesulfonyl)-1,2,3,4-tetrahydropyrimidine-2,4-dione (5e)

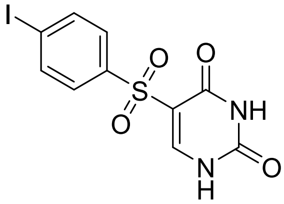

Following **GP1** the title compound **5e** was obtained as a white solid (64.4 mg, 68 %). **^{1}H-NMR** (400 MHz, DMSO-d_6): δ 12.01 (br s, 1H), 11.59 (br s, 1H), 8.25 (s, 1H), 7.99 (dt, J = 8.7, 1.6 Hz, 2H), 7.70 (dt, J = 8.7, 1.6 Hz, 2H) ppm. **^{13}C{^{1}H}-NMR** (101 MHz, DMSO-d_6): δ 158.5, 150.5, 148.9, 140.1, 137.9 (2C), 129.5 (2C), 112.1, 102.1 ppm. **HR-MS (ESI)** m/z calc. for $C_{10}H_6IN_2O_4S^-$ [M-H]$^-$: 376.9098, found: 376.9085.

5-(3-Chlorobenzenesulfonyl)-1,2,3,4-tetrahydropyrimidine-2,4-dione (5f)

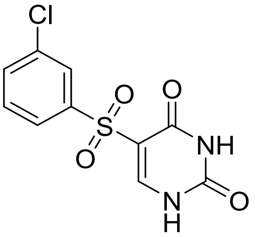

Following **GP1** the title compound **5f** was obtained as a white solid (61.8 mg, 86 %). **^{1}H-NMR** (300 MHz, DMSO-d_6): δ 12.06 (br s, 1H), 11.61 (br s, 1H), 8.27 (s, 1H), 7.98 (t, J = 1.9 Hz, 1H), 7.92 (dt, J = 7.8, 1.4 Hz, 1H), 7.77 (dd, J = 8.2, 2.1 Hz, 1H), 7.64 (t, J = 7.9 Hz, 1H) ppm. **^{13}C{^{1}H}-NMR

(75 MHz, DMSO-d$_6$): δ 158.5, 150.6, 149.4, 142.4, 133.5, 133.4, 131.0, 127.4, 126.5, 111.7 ppm. **HR-MS (ESI)** m/z calc. for $C_{10}H_6ClN_2O_4S^-$ [M-H]$^-$: 284.9742, found: 284.9732.

5-(2-Chlorobenzenesulfonyl)-1,2,3,4-tetrahydropyrimidine-2,4-dione (5g)

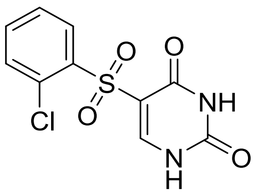

Following **GP1** the title compound **5g** was obtained as a yellow solid (65.7 mg, 92 %). **^{1}H-NMR** (300 MHz, DMSO-d$_6$): δ 12.12 (br s, 1H), 11.64 (br s, 1H), 8.35 (s, 1H), 8.17 (dd, J = 7.9, 1.7 Hz, 1H), 7.75 – 7.49 (m, 3H) ppm. **^{13}C{^{1}H}-NMR** (75 MHz, DMSO-d$_6$): δ 158.2, 150.5, 150.4, 136.7, 135.3, 132.3, 131.7, 130.9, 127.7, 110.8 ppm. **HR-MS (ESI)** m/z calc. for $C_{10}H_6ClN_2O_4S^-$ [M-H]$^-$: 284.9742, found: 284.9737.

5-(4-Nitrobenzenesulfonyl)-1,2,3,4-tetrahydropyrimidine-2,4-dione (5h)

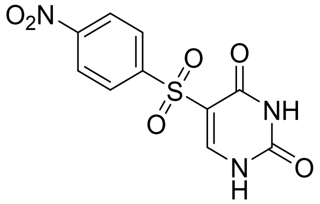

Following **GP1** the title compound **5h** was obtained as a yellow solid (41.4 mg, 56 %). **^{1}H-NMR** (400 MHz, DMSO-d$_6$): δ 11.64 (s, 1H), 8.45 – 8.36 (m, 2H), 8.33 (s, 1H), 8.26 – 8.17 (m, 2H) ppm. **^{13}C{^{1}H}-NMR** (101 MHz, DMSO-d$_6$): δ 158.5, 150.5, 150.2, 149.9, 145.8, 129.5 (2C), 124.2 (2C), 111.2 ppm. **HR-MS (ESI)** m/z calc. for $C_{10}H_7N_3NaO_6S^+$ [M+Na]$^+$: 319.9948, found: 319.9960.

5-[4-(Trifluoromethyl)benzenesulfonyl]-1,2,3,4-tetrahydropyrimidine-2,4-dione (5i)

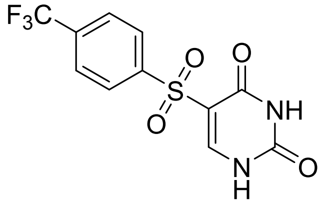

Following **GP1** the title compound **5i** was obtained as a white solid (59.5 mg, 74 %). **^{1}H-NMR** (400 MHz, DMSO-d$_6$): δ 11.60 (br s, 1H), 8.31 (s, 1H), 8.17 (d, J = 8.1 Hz, 2H), 7.99 (d, J = 8.3 Hz, 2H) ppm. **^{13}C{^{1}H}-NMR** (101 MHz, DMSO-d$_6$): δ 158.6, 150.7, 149.7, 144.4, 133.0 (q, J = 32.2 Hz),

128.9 (2H), 126.2 (q, *J* = 3.7 Hz, 2H), 123.4 (q, *J* = 273.1 Hz), 111.4 ppm. **^{19}F-NMR** (376 MHz, DMSO-d$_6$): δ -61.67 (s) ppm. **HR-MS (ESI)** m/z calc. for $C_{11}H_7F_3N_2NaO_4S^+$ [M+Na]$^+$: 342.9971, found:342.9971.

5-(4-Methoxybenzenesulfonyl)-1,2,3,4-tetrahydropyrimidine-2,4-dione (5j)

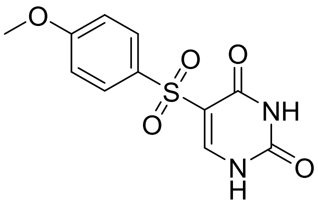

Following **GP1** the title compound **5j** was obtained as a white solid (53.4 mg, 75 %). **^{1}H-NMR** (400 MHz, DMSO-d$_6$): δ 11.92 (br s, 1H), 11.56 (b4 s, 1H), 8.21 (s, 1H), 7.87 (d, *J* = 8.5 Hz, 2H), 7.10 (d, *J* = 7.9 Hz, 2H), 3.83 (s, 3H) ppm. **^{13}C{^{1}H}-NMR** (101 MHz, DMSO-d$_6$): δ 163.1, 158.5, 150.7, 148.0, 131.9, 130.3 (2C), 114.1 (2C), 113.4, 55.8 ppm. **HR-MS (ESI)** m/z calc. for $C_{11}H_{10}N_2NaO_5S^+$ [M+Na]$^+$: 305.0203, found: 305.0212.

5-(4-Methylbenzenesulfonyl)-1,2,3,4-tetrahydropyrimidine-2,4-dione (5k)

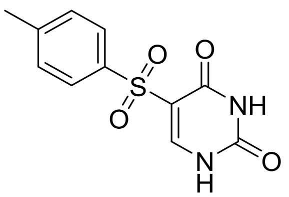

Following **GP1** the title compound **5k** was obtained as a white solid (60.6 mg, 91 %). **^{1}H-NMR** (400 MHz, DMSO-d$_6$): δ 11.94 (br s, 1H), 11.54 (br s, 1H), 8.23 (s, 1H), 7.83 (d, *J* = 8.0 Hz, 2H), 7.39 (d, *J* = 7.9 Hz, 2H), 2.38 (s, 3H) ppm. **^{13}C{^{1}H}-NMR** (101 MHz, DMSO-d$_6$): δ 158.4, 150.6, 148.3, 144.0, 137.6, 129.4 (C2), 127.9 (C2), 112.9, 21.0 ppm. **HR-MS (ESI)** m/z calc. for $C_{11}H_9N_2O_4S^-$ [M-H]$^-$: 265.0288, found: 265.0291.

5-(Pyridine-3-sulfonyl)-1,2,3,4-tetrahydropyrimidine-2,4-dione (5l)

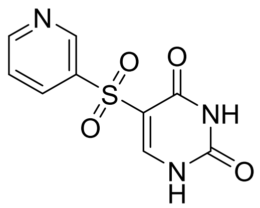

Following **GP1** the title compound **5l** was obtained as a yellow solid (62.0 mg, 99 %). **^{1}H-NMR** (400 MHz, DMSO-d$_6$): δ 12.26 (d, *J* = 6.5 Hz, 1H), 11.63 (br s, 1H), 9.09 (d, *J* = 2.3 Hz, 1H), 8.86 (dd, *J* = 4.9, 1.6 Hz, 1H), 8.37 (dt, *J* = 8.1, 1.9 Hz, 1H), 8.28 (d, *J* = 6.4 Hz, 1H), 7.68 (dd, *J* = 8.1, 4.8 Hz,

1H) ppm. **^{13}C{^{1}H}-NMR** (101 MHz, DMSO-d$_6$): δ 158.6, 153.5, 150.5, 149.4, 148.2, 136.9, 136.5, 124.2, 111.7 ppm. **HR-MS (ESI)** m/z calc. for $C_9H_8N_3O_4S^+$ [M+H]$^+$: 254.0231, found: 254.0242.

***Tert*-butyl-*N*-{4-[(2,4-dioxo-1,2,3,4-tetrahydropyrimidin-5-yl)sulfonyl]phenyl}carbamate (5m)**

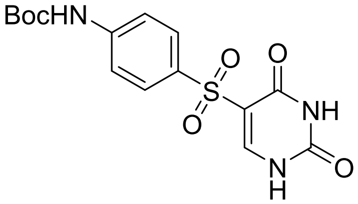

Following **GP1** the title compound **5m** was obtained and purified by flash column chromatography (dichloromethane/methanol = 95:5) as a yellow solid (0.58 g, 63 %). **^{1}H-NMR** (400 MHz, DMSO-d$_6$): δ 11.52 (br s, 1H), 9.85 (br s, 1H), 8.19 (s, 1H), 7.81 (d, J = 8.7 Hz, 2H), 7.61 (d, J = 8.7 Hz, 2H), 1.48 (s, 9H).ppm. **^{13}C{^{1}H}-NMR** (101 MHz, DMSO-d$_6$): δ 158.5, 152.5, 150.9, 148.2, 144.2, 132.9, 129.2 (2C), 117.3 (2C), 113.1, 80.0, 28.0 (3C) ppm. **HR-MS (ESI)** m/z calc. for $C_{15}H_{18}N_3O_6S^+$ [M+H]$^+$: 368.0911, found: 368.0903.

5-Methanesulfonyl-1,2,3,4-tetrahydropyrimidine-2,4-dione (5r)

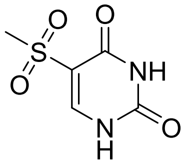

Following **GP1** the title compound **5r** was synthesized, but for the work-up, the crude product was directly filtered to obtain the product **5r** as the disodium salt as a white solid (51 mg, 87 %).

^{1}H-NMR (400 MHz, D_2O): δ 8.31 (s, 1H), 3.25 (s, 3H) ppm. **^{13}C{^{1}H}-NMR** (101 MHz, D_2O): δ 168.9, 166.7, 160.7, 108.6, 42.1 ppm. **HR-MS (ESI)** m/z calc. for $C_5H_7N_2O_4S^+$ [M+H]$^+$: 191.0122, found: 191.0118.

5-(Benzenesulfonyl)-3-methyl-1,2,3,4-tetrahydropyrimidine-2,4-dione (6a)

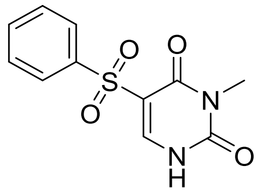

Following **GP1** the title compound **6a** was obtained as a white solid (53 mg, 79 %). **^{1}H-NMR** (400 MHz, DMSO-d$_6$): δ 12.26 (s, 1H), 8.30 (s, 1H), 7.97 (d, *J* = 7.8 Hz, 2H), 7.68 (t, *J* = 7.4 Hz, 1H), 7.60 (t, *J* = 7.6 Hz, 2H), 3.03 (s, 3H) ppm. **^{13}C{^{1}H}-NMR** (101 MHz, DMSO-d$_6$): δ 157.8, 150.5, 146.6, 140.3, 133.5, 128.9 (C2), 127.9 (C2), 112.1, 26.8 ppm. **HR-MS (ESI)** m/z calc. for $C_{11}H_{11}N_2O_4S^+$ [M+H]$^+$: 267.0435, found: 267.0443.

5-(4-Fluorobenzenesulfonyl)-3-methyl-1,2,3,4-tetrahydropyrimidine-2,4-dione (6b)

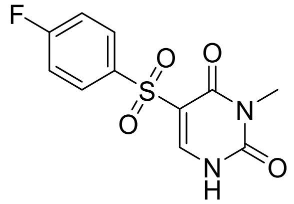

Following **GP1** the title compound **6b** was obtained as a white solid (51 mg, 72 %). **^{1}H-NMR** (400 MHz, DMSO-d$_6$): δ 12.28 (br s, 1H), 8.30 (s, 1H), 8.09 – 7.98 (m, 2H), 7.44 (t, *J* = 8.9 Hz, 2H), 3.04 (s, 3H) ppm. **^{13}C{^{1}H}-NMR** (101 MHz, DMSO-d$_6$): δ 164.9 (d, *J* = 252.4 Hz), 157.8, 150.5, 146.71, 136.6 (d, *J* = 2.9 Hz), 131.3 (d, *J* = 9.8 Hz, 2C), 116.1 (d, *J* = 22.8 Hz, 2C), 112.0, 26.8 ppm. **^{19}F-NMR** (376 MHz, DMSO-d$_6$): δ -105.23 (p, *J* = 8.0, 7.1 Hz) ppm. **HR-MS (ESI)** m/z calc. for $C_{11}H_8FN_2O_4S^-$ [M-H]$^-$: 283.0194, found: 283.0170.

5-(4-Chlorobenzenesulfonyl)-3-methyl-1,2,3,4-tetrahydropyrimidine-2,4-dione (6c)

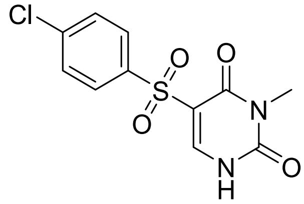

Following **GP1** the title compound **6c** was obtained as a brown solid (61 mg, 81 %). **^{1}H-NMR** (300 MHz, DMSO-d$_6$): δ 12.31 (br s, 1H), 8.31 (s, 1H), 7.98 (d, *J* = 8.6 Hz, 2H), 7.68 (d, *J* = 8.5 Hz, 2H), 3.03 (s, 3H) ppm. **^{13}C{^{1}H}-NMR** (75 MHz, DMSO-d$_6$): δ 157.8, 150.5, 147.0, 139.1, 138.6, 130.0 (C2), 129.1 (C2), 111.7, 26.8 ppm. **HR-MS (ESI)** m/z calc. for $C_{11}H_{10}ClN_2O_4S^+$ [M+H]$^+$: 301.0045, found: 301.0041.

5-(4-Bromobenzenesulfonyl)-3-methyl-1,2,3,4-tetrahydropyrimidine-2,4-dione (6d)

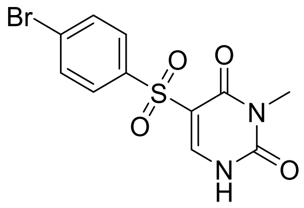

Following **GP1** the title compound **6d** was obtained as a white solid (63 mg, 73 %). **^{1}H-NMR** (400 MHz, DMSO-d$_6$): δ 12.31 (br s, 1H), 8.31 (s, 1H), 7.90 (d, J = 8.7 Hz, 2H), 7.82 (d, J = 8.7 Hz, 2H), 3.03 (s, 3H) ppm. **^{13}C{^{1}H}-NMR** (101 MHz, DMSO-d$_6$): δ 157.8, 150.5, 146.9, 139.6, 132.0 (C2), 130.0 (C2), 127.6, 111.7, 26.8 ppm. **HR-MS (ESI)** m/z calc. for $C_{11}H_{10}BrN_2O_4S^+$ [M+H]$^+$: 344.9540, found: 344.9535.

5-(4-Iodobenzenesulfonyl)-3-methyl-1,2,3,4-tetrahydropyrimidine-2,4-dione (6e)

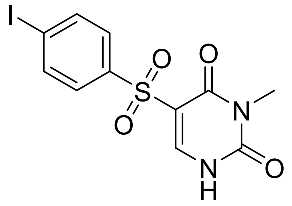

Following **GP1** the title compound **6e** was obtained as a white solid (58 mg, 59 %). **^{1}H-NMR** (400 MHz, DMSO-d$_6$): δ 12.29 (br s, 1H), 8.29 (s, 1H), 8.01 – 7.97 (m, 2H), 7.73 – 7.69 (m, 2H), 3.03 (s, 3H) ppm. **^{13}C{^{1}H}-NMR** (101 MHz, DMSO-d$_6$): δ 157.8, 150.5, 146.9, 139.9, 137.9 (2C), 129.7 (2C), 111.7, 102.2, 26.8 ppm. **HR-MS (ESI)** m/z calc. for $C_{11}H_{10}IN_2O_4S^+$ [M+H]$^+$: 392.9401, found: 392.9384.

5-(3-Chlorobenzenesulfonyl)-3-methyl-1,2,3,4-tetrahydropyrimidine-2,4-dione (6f)

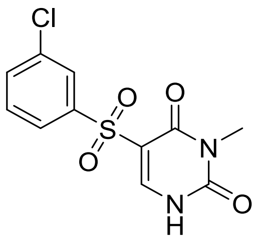

Following **GP1** the title compound **6f** was obtained as a white solid (46 mg, 61 %). **^{1}H-NMR** (400 MHz, DMSO-d$_6$): δ 12.37 (br s, 1H), 8.33 (s, 1H), 7.99 (t, J = 1.9 Hz, 1H), 7.97 – 7.90 (m, 1H), 7.80 – 7.73 (m, 1H), 7.64 (t, J = 8.0 Hz, 1H), 3.04 (s, 3H) ppm. **^{13}C{^{1}H}-NMR** (101 MHz, DMSO-d$_6$): δ

157.9, 150.6, 147.5, 142.2, 133.6, 131.1, 127.6, 126.8, 111.3, 26.9 ppm. **HR-MS (ESI)** m/z calc. for $C_{11}H_{10}ClN_2O_4S^+$ $[M+H]^+$: 301.0045, found: 301.0031.

5-(2-Chlorobenzenesulfonyl)-3-methyl-1,2,3,4-tetrahydropyrimidine-2,4-dione (6g)

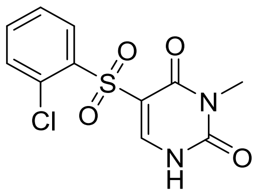

Following **GP1** the title compound **6g** was obtained as a white solid (54 mg, 70 %). **^{1}H-NMR** (400 MHz, DMSO-d$_6$): δ 8.40 (br s, 1H), 8.20 (dd, J = 8.1, 1.7 Hz, 1H), 7.75 – 7.66 (m, 1H), 7.66 – 7.58 (m, 2H), 3.02 (s, 3H) ppm. **^{13}C{^{1}H}-NMR** (101 MHz, DMSO-d$_6$): δ 157.6, 150.5, 148.6, 136.6, 135.4, 132.5, 131.8, 131.0, 127.7, 110.4, 26.8 ppm. **HR-MS (ESI)** m/z calc. for $C_{11}H_{10}ClN_2O_4S^+$ $[M+H]^+$: 301.0045, found: 301.0047.

3-Methyl-5-(4-nitrobenzenesulfonyl)-1,2,3,4-tetrahydropyrimidine-2,4-dione (6h)

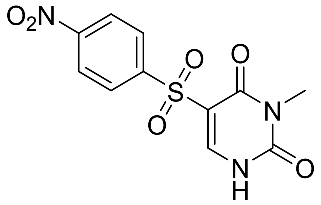

Following **GP1** the title compound **6h** was obtained as a yellow solid (41 mg, 53 %). **^{1}H-NMR** (400 MHz, DMSO-d$_6$): δ 12.43 (br s, NH), 8.41 (d, J = 2.0 Hz, 1H), 8.39 (d, J = 3.0 Hz, 2H), 8.26 – 8.21 (m, 2H), 3.03 (s, 3H) ppm. **^{13}C{^{1}H}-NMR** (101 MHz, DMSO-d$_6$): δ 157.8, 150.5, 150.2, 147.8, 145.6, 129.6 (2C), 124.2 (C2), 110.8, 26.8 ppm. **HR-MS (ESI)** m/z calc. for $C_{11}H_{10}N_3O_6S^+$ $[M+H]^+$:312.0285, found: 312.0279.

3-Methyl-5-[4-(trifluoromethyl)benzenesulfonyl]-1,2,3,4-tetrahydropyrimidine-2,4-dione (6i)

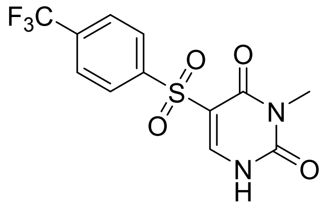

Following **GP1** the title compound **6i** was obtained as a white solid (43 mg, 52 %). **^{1}H-NMR** (400 MHz, DMSO-d$_6$): δ 12.38 (br s, 1H), 8.36 (s, 1H), 8.19 (d, J = 8.2 Hz, 2H), 8.00 (d, J = 8.3 Hz, 2H), 3.03 (s, 3H) ppm. **^{13}C{^{1}H}-NMR** (101 MHz, DMSO-d$_6$): δ 158.3, 151.0, 148.0, 144.7, 133.5 (q, J

= 32.2 Hz), 129.5 (2C), 126.6 (q, *J* = 3.7 Hz, 2C), 123.9 (q, *J* = 273.9 Hz), 111.6, 27.3 ppm. **^{19}F-NMR** (376 MHz, DMSO-d$_6$): δ -61.68 (s) ppm. **HR-MS (ESI)** m/z calc. for $C_{12}H_{10}F_3N_2O_4S^+$ [M+H]$^+$: 335.0308, found: 335.0298.

5-(4-Methoxybenzenesulfonyl)-3-methyl-1,2,3,4-tetrahydropyrimidine-2,4-dione (6j)

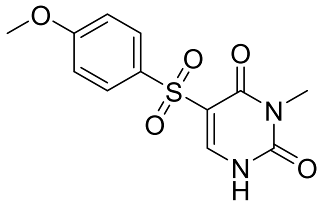

Following **GP1** the title compound **6j** was obtained as a brown solid (43 mg, 58 %). **^{1}H-NMR** (400 MHz, DMSO-d$_6$): δ 12.17 (br s, 1H), 8.25 (s, 1H), 7.94 – 7.84 (m, 2H), 7.14 – 7.06 (m, 2H), 3.84 (s, 3H), 3.04 (s, 3H) ppm. **^{13}C{^{1}H}-NMR** (101 MHz, DMSO-d$_6$): δ 163.1, 157.8, 150.6, 145.9, 131.7, 130.4, 114.1, 112.9, 55.8, 26.7 ppm. **HR-MS (ESI)** m/z calc. for $C_{12}H_{13}N_2O_5S^+$ [M+H]$^+$: 297.0540, found: 297.0539.

3-Methyl-5-(4-methylbenzenesulfonyl)-1,2,3,4-tetrahydropyrimidine-2,4-dione (6k)

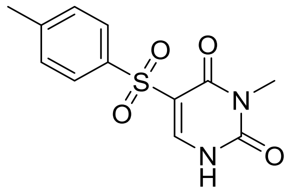

Following **GP1** the title compound **6k** was obtained as a white solid (51 mg, 73 %). **^{1}H-NMR** (400 MHz, DMSO-d$_6$): δ 12.22 (br s, 1H), 8.27 (s, 1H), 7.88 – 7.76 (m, 2H), 7.39 (d, *J* = 8.1 Hz, 2H), 3.03 (s, 3H), 2.38 (s, 3H) ppm. **^{13}C{^{1}H}-NMR** (101 MHz, DMSO-d$_6$): δ 157.7, 150.5, 146.3, 144.0, 137.4, 129.4, 128.0, 112.5, 26.7, 21.0 ppm. **HR-MS (ESI)** m/z calc. for $C_{12}H_{13}N_2O_4S^+$ [M+H]$^+$: 281.0591, found: 281.0586.

3-Methyl-5-(pyridine-3-sulfonyl)-1,2,3,4-tetrahydropyrimidine-2,4-dione (6l)

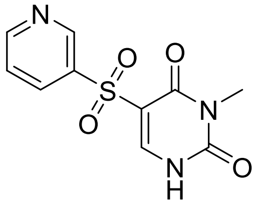

Following **GP1** the title compound **6l** was obtained as a brown solid (34 mg, 51 %). **^{1}H-NMR** (400 MHz, DMSO-d$_6$): δ 9.12 – 9.07 (m, 1H), 8.84 (d, *J* = 4.7 Hz, 1H), 8.40 – 8.31 (m, 2H), 7.65 (dd, *J* = 8.1, 4.7 Hz, 1H), 3.03 (s, 3H) ppm. **^{13}C{^{1}H}-NMR** (101 MHz, DMSO-d$_6$): δ 158.0, 153.8, 150.6,

148.6, 147.4, 136.7, 136.2, 124.0, 111.3, 26.8 ppm. **HR-MS (ESI)** m/z calc. for $C_{10}H_{10}N_3O_4S^+$ [M-H]+: 268.0387, found: 268.0392.

Tert-butyl N-{4-[(3-methyl-2,4-dioxo-1,2,3,4-tetrahydropyrimidin-5-yl)sulfonyl]phenyl}carbamate (6m)

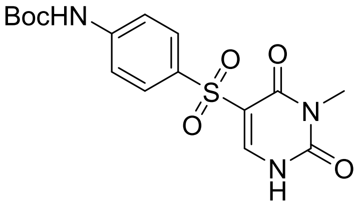

Following **GP1** the title compound **6m** was obtained and purified with flash column chromatography (cyclohexane/ethyl acetate = 3:1) as a yellow solid (70 mg, 73 %). **^{1}H-NMR** (300 MHz, DMSO-d$_6$): δ 12.12 (br s, 1H), 9.83 (br s, 1H), 8.24 (s, 1H), 7.88 – 7.78 (m, 2H), 7.67 – 7.56 (m, 2H), 3.03 (s, 3H), 1.47 (s, 9H). . **^{13}C{^{1}H}-NMR** (76 MHz, DMSO-d$_6$): δ 157.8, 152.5, 150.6, 146.0, 144.3, 132.7, 129.3 (2C), 117.3 (2C), 112.8, 80.0, 28.0 (3C), 26.8 ppm. **HR-MS (ESI)** m/z calc. for $C_{16}H_{20}N_3O_6S^+$ [M+H]+: 382.1067, found: 382.1074.

5-Methanesulfonyl-3-methyl-1,2,3,4-tetrahydropyrimidine-2,4-dione (6r)

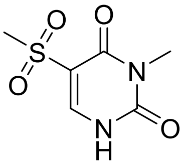

Following **GP1** the title compound **6r** was synthesized, but for the work-up, the crude product was directly filtered to obtain the product **S10** as the sodium salt as a white solid (47 mg, 83 %). **^{1}H-NMR** (400 MHz, DMSO-d$_6$): δ 8.01 (s, 1H), 3.07 (s, 3H), 3.01 (s, 3H) ppm. **^{13}C{^{1}H}-NMR** (101 MHz, DMSO-d$_6$): δ 161.4, 159.8, 157.9, 106.4, 42.3, 26.4 ppm. **HR-MS (ESI)** m/z calc. for $C_6H_9N_2O_4S^+$ [M-H]+: 205.0278, found: 205.0289.

5-(Benzenesulfonyl)-3-phenyl-1,2,3,4-tetrahydropyrimidine-2,4-dione (7)

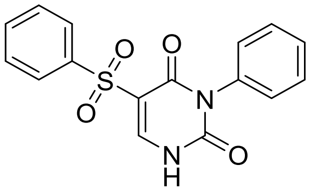

Following **GP1** the title compound **7** was obtained as a white solid (58 mg, 71 %). **^{1}H-NMR** (400 MHz, DMSO-d$_6$): δ 12.40 (br s, 1H), 8.41 (s, 1H), 7.98 – 7.94 (m, 2H), 7.71 – 7.66 (m, 1H), 7.59 (dd, J = 8.4, 7.0 Hz, 2H), 7.46 – 7.36 (m, 3H), 7.21 – 7.17 (m, 2H) ppm. **^{13}C{^{1}H}-NMR** (101 MHz,

DMSO-d_6): δ 157.8, 150.5, 147.6, 140.4, 134.4, 133.5, 129.0, 128.9 (2C), 128.7 (2C), 128.5 (2C), 127.9 (2C), 112.7 ppm. **HR-MS (ESI)** m/z calc. for $C_{16}H_{12}N_2O_4S^+$ [M+H]$^+$: 329.0591, found: 329.0597.

2.4.3 Enol ethers 8a-r

Ethyl (2*E*)-2-(benzenesulfonyl)-3-ethoxyprop-2-enoate (9a)

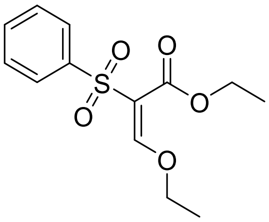

Following **GP2** the title compound **9a** was obtained and purified with flash column chromatography (cyclohexane/ethyl acetate = 4:1) as a yellow solid (1.01 g, 79 %). **^{1}H-NMR** (400 MHz, DMSO-d_6): δ 8.21 (s, 1H), 7.97 – 7.85 (m, 2H), 7.70 – 7.64 (m, 1H), 7.63 – 7.56 (m, 2H), 4.47 (q, *J* = 7.1 Hz, 2H), 4.00 (q, *J* = 7.1 Hz, 2H), 1.32 (t, *J* = 7.1 Hz, 3H), 1.04 (t, *J* = 7.1 Hz, 3H) ppm. **^{13}C{^{1}H}-NMR** (101 MHz, DMSO-d_6): δ 169.5, 160.1, 141.6, 133.0, 128.8, 127.5, 111.8, 74.1, 60.3, 15.1, 13.7 ppm. **HR-MS (ESI)** m/z calc. for $C_{13}H_{17}O_5S^+$ [M+H]$^+$: 285.0792, found: 285.0799.

Ethyl (2*E*)-3-ethoxy-2-(4-fluorobenzenesulfonyl)prop-2-enoate (9b)

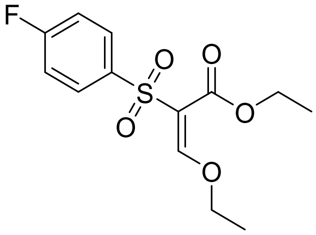

Following **GP2** the title compound **9b** was obtained and purified with flash column chromatography (cyclohexane/ethyl acetate = 4:1) as a yellow solid (0.98 g, 72 %). **^{1}H-NMR** (400 MHz, DMSO-d_6): δ 8.22 (s, 1H), 8.01 – 7.95 (m, 2H), 7.48 – 7.41 (m, 2H), 4.47 (q, *J* = 7.1 Hz, 2H), 4.02 (q, *J* = 7.1 Hz, 2H), 1.32 (t, *J* = 7.1 Hz, 3H), 1.06 (t, *J* = 7.1 Hz, 3H) ppm. **^{13}C{^{1}H}-NMR** (101 MHz, DMSO-d_6): δ 170.3, 165.0 (d, *J* = 251.8 Hz), 160.5, 138.5 (d, *J* = 2.9 Hz), 131.3 (2C), 116.5 (d, *J* = 22.9 Hz, 2C), 112.1, 74.6, 60.9, 15.5, 14.2 ppm. **^{19}F-NMR** (376 MHz, DMSO-d_6): δ -106.05 (tt, *J* = 8.7, 5.1 Hz) ppm. **LR-MS (EI)** m/z calc. for $C_{13}H_{15}FO_5S^{\bullet+}$ ($M^{+\bullet}$): 302.1, found: 302.1.

Ethyl (2*E*)-2-(4-chlorobenzenesulfonyl)-3-ethoxyprop-2-enoate (9c)

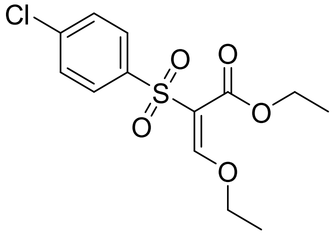

Following **GP2** the title compound **9c** was obtained and purified with flash column chromatography (cyclohexane/ethyl acetate = 4:1) as a yellow solid (0.87 g, 61 %). **^{1}H-NMR** (400 MHz, DMSO-d$_6$): *δ* 8.22 (s, 1H), 7.97 – 7.86 (m, 2H), 7.72 – 7.64 (m, 2H), 4.47 (q, *J* = 7.1 Hz, 2H), 4.02 (q, *J* = 7.1 Hz, 2H), 1.32 (t, *J* = 7.1 Hz, 3H), 1.06 (t, *J* = 7.1 Hz, 3H) ppm. **^{13}C{^{1}H}-NMR** (101 MHz, DMSO-d$_6$): *δ* 170.6, 160.5, 141.0, 138.4, 130.1 (2C), 129.5 (2C), 111.8, 74.8, 61.0, 15.5, 14.2 ppm. **LR-MS (EI)** m/z calc. for $C_{13}H_{15}ClO_5S^{•+}$ ($M^{+•}$): 318.0, found: 318.0.

Ethyl (2E)-2-(4-bromobenzenesulfonyl)-3-ethoxyprop-2-enoate (9d)

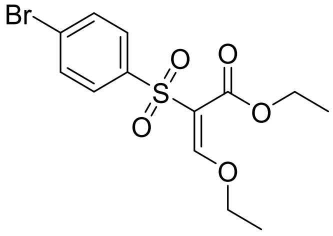

Following **GP2** the title compound **9d** was obtained and purified with flash column chromatography (cyclohexane/ethyl acetate = 4:1) as a yellow solid (0.51 g, 31 %). **^{1}H-NMR** (400 MHz, DMSO-d$_6$): *δ* 8.22 (s, 1H), 7.93 – 7.77 (m, 4H), 4.47 (q, *J* = 7.1 Hz, 2H), 4.02 (q, *J* = 7.1 Hz, 2H), 1.32 (t, *J* = 7.1 Hz, 3H), 1.06 (t, *J* = 7.1 Hz, 3H) ppm. **^{13}C{^{1}H}-NMR** (101 MHz, DMSO-d$_6$): *δ* 170.1, 160.0, 141.0, 132.0 (2C), 129.7 (2C), 127.0, 111.3, 74.3, 60.5, 15.0, 13.7 ppm. **LR-MS (EI)** m/z calc. for $C_{13}H_{15}BrO_5S^{•+}$ ($M^{+•}$): 364.0, found: 364.0.

Ethyl (2*E*)-3-ethoxy-2-(4-iodobenzenesulfonyl)prop-2-enoate (9e)

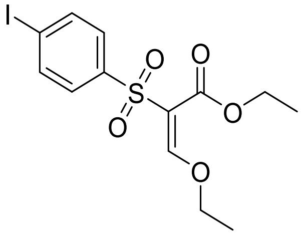

Following **GP2** the title compound **9e** was obtained and purified with flash column chromatography (cyclohexane/ethyl acetate = 4:1) as a yellow solid (1.20 g, 65 %). **^{1}H-NMR** (400 MHz, DMSO-d$_6$): *δ* 8.21

(s, 1H), 8.04 – 7.95 (m, 2H), 7.70 – 7.62 (m, 2H), 4.47 (q, *J* = 7.1 Hz, 2H), 4.02 (q, *J* = 7.1 Hz, 2H), 1.31 (t, *J* = 7.1 Hz, 3H), 1.06 (t, *J* = 7.1 Hz, 3H) ppm. **^{13}C{^{1}H}-NMR** (101 MHz, DMSO-d$_6$): *δ* 170.0, 160.0, 141.3, 137.8 (2C), 129.3 (2C), 111.3, 101.4, 74.2, 60.5, 15.1, 13.7 ppm. **LR-MS (EI)** m/z calc. for $C_{13}H_{15}IO_5S^{•+}$ ($M^{+•}$): 410.0, found: 410.0.

Ethyl (2*E*)-2-(3-chlorobenzenesulfonyl)-3-ethoxyprop-2-enoate (9f)

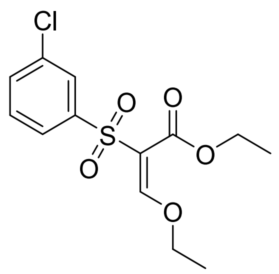

Following **GP2** the title compound **9f** was obtained and purified with flash column chromatography (cyclohexane/ethyl acetate = 4:1) as a yellow solid (1.19 g, 83 %). **^{1}H-NMR** (400 MHz, DMSO-d$_6$): *δ* 8.23 (s, 1H), 7.95 (t, *J* = 1.9 Hz, 1H), 7.90 – 7.87 (m, 1H), 7.78 – 7.74 (m, 1H), 7.64 (t, *J* = 7.9 Hz, 1H), 4.49 (q, *J* = 7.1 Hz, 2H), 4.02 (q, *J* = 7.1 Hz, 2H), 1.32 (t, *J* = 7.1 Hz, 3H), 1.06 (t, *J* = 7.1 Hz, 3H) ppm. **^{13}C{^{1}H}-NMR** (101 MHz, DMSO-d$_6$): *δ* 170.6, 159.9, 143.6, 133.4, 133.0, 130.9, 127.3, 126.4, 110.9, 74.4, 60.5, 15.0, 13.7 ppm. **LR-MS (EI)** m/z calc. for $C_{13}H_{15}ClO_5S^{•+}$ ($M^{+•}$): 318.0, found: 318.0.

Ethyl (2*E*)-2-(2-chlorobenzenesulfonyl)-3-ethoxyprop-2-enoate (9g)

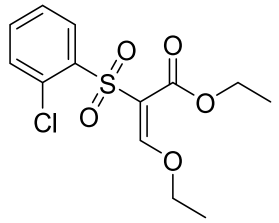

Following **GP2** the title compound **9g** was obtained and purified with flash column chromatography (cyclohexane/ethyl acetate = 4:1) as a yellow solid (0.98 g, 68 %). **^{1}H-NMR** (400 MHz, DMSO-d$_6$): *δ* 8.26 (s, 1H), 8.12 (dd, *J* = 7.9, 1.7 Hz, 1H), 7.72 – 7.58 (m, 3H), 4.53 (q, *J* = 7.1 Hz, 2H), 3.96 (q, *J* = 7.1 Hz, 2H), 1.32 (t, *J* = 7.1 Hz, 3H), 0.98 (t, *J* = 7.1 Hz, 3H) ppm. **^{13}C{^{1}H}-NMR** (101 MHz, DMSO-d$_6$): *δ* 171.9, 159.7, 137.7, 134.9, 132.0, 131.7, 130.6, 127.6, 110.1, 74.4, 60.4, 15.3, 13.6 ppm. **LR-MS (EI)** m/z calc. for $C_{13}H_{15}ClO_5S^{•+}$ ($M^{+•}$): 318.0, found: 318.0.

Ethyl (2*E*)-3-ethoxy-2-(4-nitrobenzenesulfonyl)prop-2-enoate (9h)

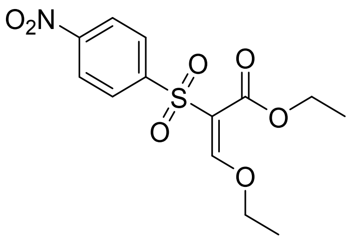

Following **GP2** the title compound **9h** was obtained and purified with flash column chromatography (cyclohexane/ethyl acetate = 4:1) as a yellow solid (1.00 g, 68 %). **^{1}H-NMR** (400 MHz, DMSO-d_6): δ 8.43 – 8.40 (m, 2H), 8.30 (s, 1H), 8.20 – 8.16 (m, 2H), 4.52 (q, *J* = 7.1 Hz, 2H), 4.02 (q, *J* = 7.1 Hz, 2H), 1.33 (t, *J* = 7.1 Hz, 3H), 1.06 (t, *J* = 7.1 Hz, 3H) ppm. **^{13}C{^{1}H}-NMR** (101 MHz, DMSO-d_6): δ 171.1, 159.9, 149.9, 147.1, 129.2 (2C), 124.4 (2C), 110.4, 74.6, 60.6, 15.0, 13.70 ppm. **LR-MS (EI)** m/z calc. for $C_{13}H_{15}NO_7S^{\bullet+}$ ($M^{+\bullet}$): 329.1, found: 329.0.

Ethyl (2*E*)-3-ethoxy-2-[4-(trifluoromethyl)benzenesulfonyl]prop-2-enoate (9i)

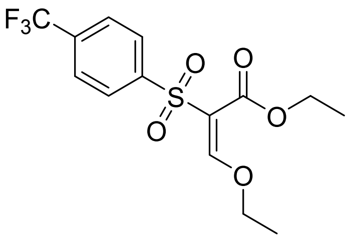

Following **GP2** the title compound **9i** was obtained and purified with flash column chromatography (cyclohexane/ethyl acetate = 4:1) as a yellow solid (0.98 g, 62 %). **^{1}H-NMR** (400 MHz, DMSO-d_6): δ 8.28 (s, 1H), 8.20 – 8.11 (m, 2H), 8.03 – 7.96 (m, 2H), 4.50 (q, *J* = 7.1 Hz, 2H), 4.02 (q, *J* = 7.1 Hz, 2H), 1.33 (t, *J* = 7.1 Hz, 3H), 1.04 (t, *J* = 7.1 Hz, 3H) ppm. **^{13}C{^{1}H}-NMR** (101 MHz, DMSO-d_6): δ 170.7, 159.9, 145.6 (d, *J* = 1.5 Hz), 132.6 (q, *J* = 32.1 Hz), 128.6 (2C), 126.1 (q, *J* = 3.7 Hz), 123.5 (q, *J* = 273.0 Hz, 2C), 110.7, 74.5, 60.5, 15.0, 13.7 ppm. **^{19}F-NMR** (376 MHz, DMSO-d_6): δ -61.63 (s) ppm. **LR-MS (EI)** m/z calc. for $C_{14}H_{15}F_3O_5S^{\bullet+}$ ($M^{+\bullet}$): 352.1, found: 352.1.

Ethyl (2*E*)-3-ethoxy-2-(4-methoxybenzenesulfonyl)prop-2-enoate (9j)

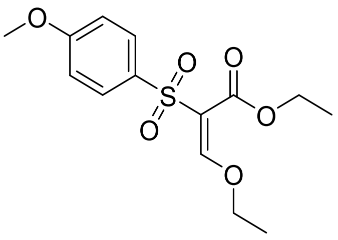

Following **GP2** the title compound **9j** was obtained and purified with flash column chromatography (cyclohexane/ethyl acetate = 3:1) as a yellow solid (0.37 g, 24 %). **^{1}H-NMR** (400 MHz, DMSO-d_6): δ 8.16

(s, 1H), 7.87 – 7.77 (m, 2H), 7.14 – 7.06 (m, 2H), 4.43 (q, *J* = 7.1 Hz, 2H), 4.02 (q, *J* = 7.1 Hz, 2H), 3.84 (s, 3H), 1.30 (t, *J* = 7.1 Hz, 3H), 1.08 (t, *J* = 7.1 Hz, 3H) ppm. **^{13}C{^{1}H}-NMR** (101 MHz, DMSO-d$_6$): *δ* 168.7, 162.6, 160.2, 133.2, 130.0 (2C), 114.0 (2C), 112.6, 73.8, 60.3, 55.7, 15.1, 13.8 ppm. **LR-MS (EI)** m/z calc. for $C_{14}H_{18}O_6S^{\bullet+}$ ($M^{+\bullet}$): 314.1, found: 314.1.

Ethyl (2*E*)-3-ethoxy-2-(4-methylbenzenesulfonyl)prop-2-enoate (9k)

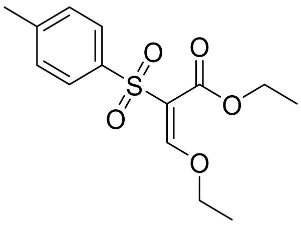

Following **GP2** the title compound **9k** was obtained and purified with flash column chromatography (cyclohexane/ethyl acetate = 4:1) as a yellow solid (0.56 g, 42 %). **^{1}H-NMR** (400 MHz, DMSO-d$_6$): *δ* 8.18 (s, 1H), 7.79 – 7.76 (m, 2H), 7.42 – 7.37 (m, 2H), 4.45 (q, *J* = 7.1 Hz, 2H), 4.00 (q, *J* = 7.2 Hz, 1H), 2.39 (s, 3H), 1.31 (t, *J* = 7.1 Hz, 3H), 1.06 (t, *J* = 7.1 Hz, 3H) ppm. **^{13}C{^{1}H}-NMR** (101 MHz, DMSO-d$_6$): *δ* 169.2, 160.2, 143.4, 138.8, 129.3 (2C), 127.7 (2C), 112.2, 74.0, 60.4, 21.0, 15.1, 13.8 ppm. **LR-MS (EI)** m/z calc. for $C_{14}H_{18}O_5S^{\bullet+}$ ($M^{+\bullet}$): 298.1, found: 298.1.

Ethyl (2*E*)-3-ethoxy-2-(pyridine-2-sulfonyl)prop-2-enoate (9n)

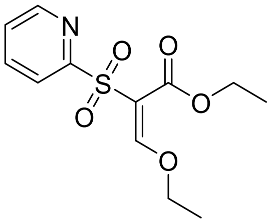

Following **GP2** the title compound **9n** was obtained and purified with flash column chromatography (cyclohexane/ethyl acetate = 3:7) as a yellow solid (0.55 g, 43 %). **^{1}H-NMR** (400 MHz, DMSO-d$_6$): *δ* 8.72 – 8.69 (m, 1H), 8.20 (s, 1H), 8.16 – 8.06 (m, 2H), 7.71 – 7.66 (m, 1H), 4.52 (q, *J* = 7.1 Hz, 2H), 3.94 (q, *J* = 7.1 Hz, 2H), 1.33 (t, *J* = 7.1 Hz, 3H), 0.93 (t, *J* = 7.1 Hz, 3H) ppm. **^{13}C{^{1}H}-NMR** (101 MHz, DMSO-d$_6$): *δ* 170.5, 159.9, 158.1, 149.8, 138.5, 127.3, 122.0, 109.2, 74.4, 60.3, 15.2, 13.6 ppm. **LR-MS (EI)** m/z calc. for $C_{12}H_{15}NO_5S^{\bullet+}$ ($M^{+\bullet}$): 286.1, found: 286.1.

Ethyl (2*E*)-3-ethoxy-2-methanesulfonylprop-2-enoate (9r)

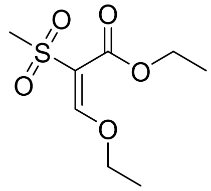

Following **GP2** the title compound **9r** was obtained and purified with flash column chromatography (cyclohexane/ethyl acetate = 3:2) as a yellow oil (0.43 g, 43 %). **^{1}H-NMR** (400 MHz, DMSO-d$_6$): δ 7.89 (s, 1H), 4.39 (q, *J* = 7.1 Hz, 2H), 4.19 (q, *J* = 7.1 Hz, 2H), 3.17 (s, 3H), 1.29 (t, *J* = 7.1 Hz, 3H), 1.23 (t, *J* = 7.1 Hz, 3H) ppm. **^{13}C{^{1}H}-NMR** (101 MHz, DMSO-d$_6$): δ 168.5, 161.0, 112.2, 73.8, 60.7, 43.7, 15.1, 14.0 ppm. **LR-MS (EI)** m/z calc. for $C_8H_{14}O_5S^{\bullet +}$ ($M^{+\bullet}$): 222.1, found: 222.1.

2.4.4 Thiouracils 11a-r, 12a-r, 13

5-(Benzenesulfonyl)-2-sulfanylidene-1,2,3,4-tetrahydropyrimidin-4-one (11a)

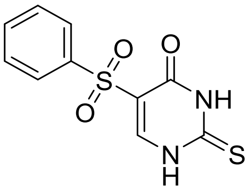

Following **GP3** the title compound **11a** was obtained as a yellow solid (63 mg, 93 %). **^{1}H-NMR** (400 MHz, DMSO-d$_6$): δ 13.14 (br s, 1H), 12.97 (br s, 1H), 8.08 (s, 1H), 7.97 (d, J = 7.4 Hz, 2H), 7.71 (t, J = 7.4 Hz, 1H), 7.61 (t, J = 7.7 Hz, 2H) ppm. **^{13}C{^{1}H}-NMR** (101 MHz, DMSO-d$_6$): δ 177.2, 155.6, 146.6, 139.8, 133.7, 129.00 (2C), 128.0 (2C), 116.4 ppm. **HR-MS (ESI)** m/z calc. for $C_{10}H_9N_2O_3S_2^+$ [M+H]$^+$: 269.0050, found: 269.0066.

5-(4-Fluorobenzenesulfonyl)-2-sulfanylidene-1,2,3,4-tetrahydropyrimidin-4-one (11b)

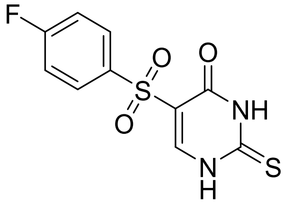

Following **GP3** the title compound **11b** was obtained as a yellow solid (75 mg, 91 %). **^{1}H-NMR** (400 MHz, DMSO-d$_6$): δ 13.16 (br s, 1H), 12.99 (br s, 1H), 8.10 – 8.00 (m, 3H), 7.50 – 7.40 (m, 2H) ppm. **^{13}C{^{1}H}-NMR** (101 MHz, DMSO-d$_6$): δ 177.17, 165.01 (d, J = 252.8 Hz), 155.6, 146.7, 136.1 (d, J = 2.9 Hz), 131.4 (d, J = 9.9 Hz, 2C), 116.3, 116.2 (d, J = 22.8 Hz, 2C) ppm. **^{19}F-NMR** (376 MHz, DMSO-d$_6$): δ -104.78 (tt, J = 8.1, 5.3 Hz) ppm. **HR-MS (ESI)** m/z calc. for $C_{10}H_8FN_2O_3S_2^+$ [M+H]$^+$: 286.9955, found: 286.9961.

5-(4-Chlorobenzenesulfonyl)-2-sulfanylidene-1,2,3,4-tetrahydropyrimidin-4-one (11c)

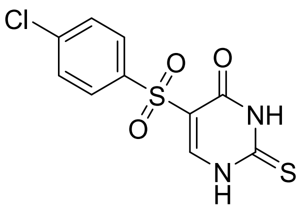

Following **GP3** the title compound **11c** was obtained as a yellow solid (74 mg, 97 %). **^{1}H-NMR** (400 MHz, DMSO-d$_6$): δ 13.17 (br s, 1H), 13.00 (br s, 1H), 8.08 (s, 1H), 8.01 – 7.95 (m, 2H), 7.72 – 7.67 (m, 2H) ppm. **^{13}C{^{1}H}-NMR** (101 MHz, DMSO-d$_6$): δ 177.2, 155.6, 146.9, 138.8, 138.6, 130.1 (2C), 129.1 (2C), 116.0 ppm. **HR-MS (ESI)** m/z calc. for $C_{10}H_8ClN_2O_3S_2^+$ [M+H]$^+$: 302.9660, found: 302.9674.

5-(4-Bromobenzenesulfonyl)-2-sulfanylidene-1,2,3,4-tetrahydropyrimidin-4-one (11d)

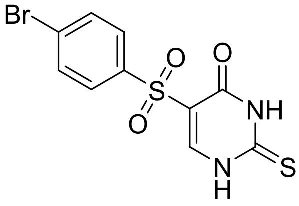

Following **GP3** the title compound **11d** was obtained as a yellow solid (77 mg, 89 %). **^{1}H-NMR** (400 MHz, DMSO-d$_6$): δ 13.17 (br s, 1H), 13.00 (br s, 1H), 8.07 (s, 1H), 7.92 – 7.87 (m, 2H), 7.85 – 7.80 (m, 2H) ppm. **^{13}C{^{1}H}-NMR** (101 MHz, DMSO-d$_6$): δ 177.2, 155.6, 146.9, 139.0, 132.1 (2C), 130.1 (2C), 127.9, 115.9 ppm. **HR-MS (ESI)** m/z calc. for $C_{10}H_7BrN_2NaO_3S_2^+$ [M+Na]$^+$: 368.8974, **found:** 368.8996.

5-(4-Iodobenzenesulfonyl)-2-sulfanylidene-1,2,3,4-tetrahydropyrimidin-4-one (11e)

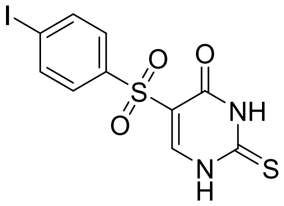

Following **GP3** the title compound **11e** was obtained as a white solid (92 mg, 93 %). **^{1}H-NMR** (400 MHz, DMSO-d$_6$): δ 13.16 (br s, 1H), 12.99 (br s, 1H), 8.06 (s, 1H), 8.02 – 7.98 (m, 2H), 7.75 – 7.69 (m, 2H) ppm. **^{13}C{^{1}H}-NMR** (101 MHz, DMSO-d$_6$): δ 177.2, 155.6, 146.8, 139.4, 137.9 (2C), 129.7 (2C), 116.0, 102.5 ppm. **HR-MS (ESI)** m/z calc. for $C_{10}H_8IN_2O_3S_2^+$ [M+H]$^+$: 394.9016, found: 394.9000.

5-(3-Chlorobenzenesulfonyl)-2-sulfanylidene-1,2,3,4-tetrahydropyrimidin-4-one (11f)

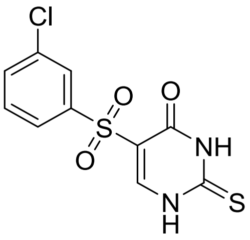

Following **GP3** the title compound **11f** was obtained as a white solid (72 mg, 95 %). **^{1}H-NMR** (400 MHz, DMSO-d$_6$): δ 13.20 (br s, 1H), 13.00 (br s, 1H), 8.09 (s, 1H), 8.01 (t, J = 1.9 Hz, 1H), 7.98 – 7.90 (m, 1H), 7.83 – 7.75 (m, 1H), 7.65 (t, J = 8.0 Hz, 1H) ppm. **^{13}C{^{1}H}-NMR** (101 MHz, DMSO-d$_6$): δ 177.2, 155.7, 147.3, 141.7, 133.7, 133.6, 131.0, 127.6, 126.8, 115.5 ppm. **HR-MS (ESI)** m/z calc. for $C_{10}H_8ClN_2O_3S_2^+$ [M+H]$^+$: 302.9660, found: 302.9671.

5-(2-Chlorobenzenesulfonyl)-2-sulfanylidene-1,2,3,4-tetrahydropyrimidin-4-one (11g)

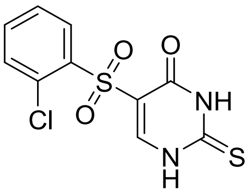

Following **GP3** the title compound **11g** was obtained as a yellow solid (74 mg, 97 %). **^{1}H-NMR** (400 MHz, DMSO-d$_6$): δ 13.31 (br s, 1H), 13.08 (br s, 1H), 8.21 – 8.14 (m, 2H), 7.77 – 7.68 (m, 1H), 7.67 – 7.61 (m, 3H) ppm. **^{13}C{^{1}H}-NMR** (101 MHz, DMSO-d$_6$): δ 177.2, 155.4, 148.2, 136.2, 135.6, 132.4, 131.9, 131.0, 127.9, 114.8 ppm. **HR-MS (ESI)** m/z calc. for $C_{10}H_8ClN_2O_3S_2^+$ [M+H]$^+$: 302.9660, found: 302. 9659.

5-(4-Nitrobenzenesulfonyl)-2-sulfanylidene-1,2,3,4-tetrahydropyrimidin-4-one (11h)

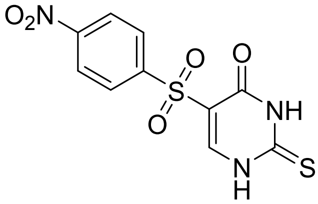

Following **GP3** the title compound **11h** was obtained as a yellow solid (70 mg, 89 %). **^{1}H-NMR** (400 MHz, DMSO-d$_6$): δ 13.26 (br s, 1H), 13.04 (br s, 1H), 8.43 – 8.38 (m, 2H), 8.26 – 8.22 (m, 2H), 8.14 (s, 1H) ppm. **^{13}C{^{1}H}-NMR** (101 MHz, DMSO-d$_6$): δ 177.3, 155.7, 150.4, 147.6, 145.1, 129.7 (2C), 124.2 (2C), 115.1 ppm. **HR-MS (ESI)** m/z calc. for $C_{10}H_7N_3NaO_5S_2^+$ [M+Na]$^+$: 335.9720, found: 335.9727.

2-Sulfanylidene-5-[4-(trifluoromethyl)benzenesulfonyl]-1,2,3,4-tetrahydropyrimidin-4-one (11i)

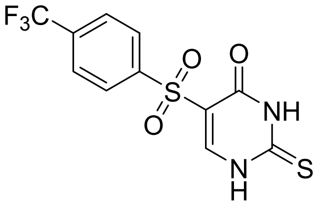

Following **GP3** the title compound **11i** was obtained as a white solid (76 mg, 91 %). **^{1}H-NMR** (400 MHz, DMSO-d$_6$): δ 13.20 (br s, 1H), 13.02 (br s, 1H), 8.22 – 8.18 (m, 2H), 8.12 (s, 1H), 8.00 (d, J = 8.4 Hz, 2H) ppm. **^{13}C{^{1}H}-NMR** (101 MHz, DMSO-d$_6$): δ 177.3, 155.7, 147.4, 143.7, 133.2 (q, J = 32.3 Hz), 129.1 (2C), 126.2 (q, J = 3.8 Hz, 2C), 123.4 (q, J = 273.2 Hz), 115.4 ppm. **^{19}F-NMR** (376 MHz, DMSO-d$_6$): δ -61.70 (s) ppm. **HR-MS (ESI)** m/z calc. for $C_{11}H_8F_3N_2O_3S_2^+$ [M+H]$^+$: 336.9923, found: 336.9915.

5-(4-Methoxybenzenesulfonyl)-2-sulfanylidene-1,2,3,4-tetrahydropyrimidin-4-one (11j)

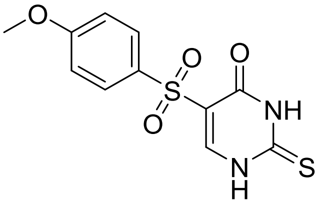

Following **GP3** the title compound **11j** was obtained as a yellow solid (69 mg, 93 %). **^{1}H-NMR** (400 MHz, DMSO-d$_6$): δ 13.08 (br s, 1H), 12.94 (br s, 1H), 8.04 (s, 1H), 7.93 – 7.85 (m, 2H), 7.17 – 7.07 (m, 2H), 3.84 (s, 3H) ppm. **^{13}C{^{1}H}-NMR** (101 MHz, DMSO-d$_6$): δ 177.1, 163.3, 155.6, 145.8, 131.2, 130.5 (2C), 117.3, 114.2 (2C), 55.8 ppm. **HR-MS (ESI)** m/z calc. for $C_{11}H_{11}N_2O_4S_2^+$ [M+H]$^+$: 299.0155, found: 299.0151.

5-(4-Methylbenzenesulfonyl)-2-sulfanylidene-1,2,3,4-tetrahydropyrimidin-4-one (11k)

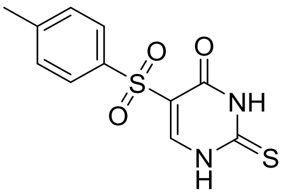

Following **GP3** the title compound **11k** was obtained as a yellow solid (68 mg, 96 %). **^{1}H-NMR** (400 MHz, DMSO-d$_6$): δ 13.14 (br s, 1H), 12.98 (br s, 1H), 8.06 (s, 1H), 7.85 (d, J = 8.4 Hz, 2H), 7.40 (d, J = 8.1 Hz, 2H), 2.38 (s, 3H) ppm. **^{13}C{^{1}H}-NMR** (101 MHz, DMSO-d$_6$): δ 177.2, 155.7, 146.4, 144.4, 136.9, 129.5 (2C), 128.2 (2C), 116.8, 21.1 ppm. **HR-MS (ESI)** m/z calc. for $C_{11}H_{11}N_2O_3S_2^+$ [M+H]$^+$: 283.0206, found: 283.0215.

5-(Pyridine-2-sulfonyl)-2-sulfanylidene-1,2,3,4-tetrahydropyrimidin-4-one (11n)

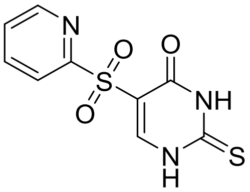

Following **GP3** the title compound **11n** was obtained as a yellow solid (61 mg, 91 %). **^{1}H-NMR** (400 MHz, DMSO-d$_6$): δ 13.24 (br s, 1H), 13.03 (br s, 1H), 8.70 (dt, J = 4.7, 1.3 Hz, 1H), 8.17 – 8.15 (m, 2H), 8.13 (s, 1H), 7.76 – 7.68 (m, 1H) ppm. **^{13}C{^{1}H}-NMR** (101 MHz, DMSO-d$_6$): δ 177.2, 156.7, 155.7, 150.1, 147.6, 138.8, 128.0, 122.6, 114.3 ppm. **HR-MS (ESI)** m/z calc. for $C_9H_8N_3O_3S_2^+$ [M+H]$^+$: 270.0002, found: 270.0003.

5-Methanesulfonyl-2-sulfanylidene-1,2,3,4-tetrahydropyrimidin-4-one (11r)

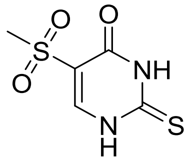

Following **GP3** the title compound **11r** was synthesized, but for the work-up, the crude product was purified with flash column chromatography (dichloromethane/methanol/ formic acid = 89:10:1) as a yellow solid (14 mg, 27 %). **^{1}H-NMR** (400 MHz, DMSO-d_6): 7.82 (s, 1H), 3.17 (s, 3H) ppm. **^{13}C{^{1}H}-NMR** (101 MHz, DMSO-d_6): δ 177.3, 156.8, 145.8, 116.9, 42.0 ppm. **HR-MS (ESI)** m/z calc. for $C_5H_5N_2O_3S_2^+$ $[M-H]^-$: 204.9747, found: 204.9735.

5-(Benzenesulfonyl)-3-methyl-2-sulfanylidene-1,2,3,4-tetrahydropyrimidin-4-one (12a)

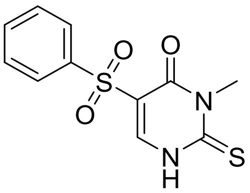

Following **GP3** the title compound **12a** was obtained as a white solid (63 mg, 89 %). ^{1}**H-NMR** (400 MHz, DMSO-d$_6$): δ 13.44 (br s, 1H), 8.15 (s, 1H), 8.04 – 7.97 (m, 2H), 7.76 – 7.67 (m, 1H), 7.62 (dd, *J* = 8.3, 6.9 Hz, 2H), 3.42 (s, 3H) ppm. ^{13}**C{**^{1}**H}-NMR** (101 MHz, DMSO-d$_6$): δ 177.8, 155.9, 145.2, 140.2, 134.2, 129.5 (2C), 128.6 (2C), 115.7, 33.6 ppm. **HR-MS (ESI)** m/z calc. for $C_{11}H_{11}N_2O_3S_2^+$ [M+H]$^+$: 283.0206, found: 283.0205.

5-(4-Fluorobenzenesulfonyl)-3-methyl-2-sulfanylidene-1,2,3,4-tetrahydropyrimidin-4-one (12b)

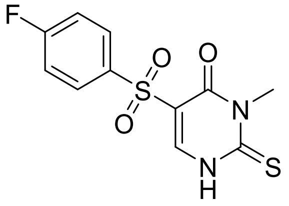

Following **GP3** the title compound **12b** was obtained as a yellow solid (57 mg, 77 %). ^{1}**H-NMR** (400 MHz, DMSO-d$_6$): δ 13.43 (br s, 1H), 8.13 (s, 1H), 8.09 – 8.04 (m, 2H), 7.45 (t, *J* = 8.9 Hz, 2H), 3.41 (s, 3H) ppm. ^{13}**C{**^{1}**H}-NMR** (101 MHz, DMSO-d$_6$): δ 177.3, 165.0 (d, *J* = 252.7 Hz), 155.5, 144.8, 136.0 (d, *J* = 2.9 Hz), 131.5 (d, *J* = 10.0 Hz, 2C), 116.2 (d, *J* = 22.9 Hz, 2C), 115.1, 33.1 ppm. ^{19}**F-NMR** (376 MHz, DMSO-d$_6$): δ -104.76 (tt, *J* = 9.3, 5.5, 4.9 Hz) ppm. **HR-MS (ESI)** m/z calc. for $C_{11}H_{10}FN_2O_3S_2^+$ [M+H]$^+$: 301.0112, found: 301.0111.

5-(4-Chlorobenzenesulfonyl)-3-methyl-2-sulfanylidene-1,2,3,4-tetrahydropyrimidin-4-one (12c)

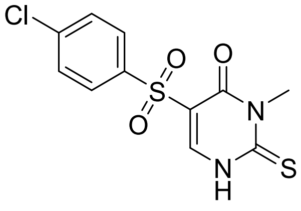

Following **GP3** the title compound **12c** was obtained as a white solid (65 mg, 84 %). ^{1}**H-NMR** (400 MHz, DMSO-d$_6$): δ 13.45 (br s, 1H), 8.13 (s, 1H), 8.04 – 7.96 (m, 2H), 7.73 – 7.65 (m, 2H), 3.41 (s, 3H) ppm. ^{13}**C{**^{1}**H}-NMR** (101 MHz, DMSO-d$_6$): δ 177.8, 155.9, 145.4, 139.3, 139.0, 130.7 (C2), 129.6 (2C), 115.3, 33.6 ppm. **HR-MS (ESI)** m/z calc. for $C_{11}H_{10}ClN_2O_3S_2^+$ [M+H]$^+$: 316.9816, found: 316.9813.

5-(4-Bromobenzenesulfonyl)-3-methyl-2-sulfanylidene-1,2,3,4-tetrahydropyrimidin-4-one (12d)

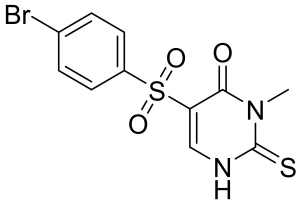

Following **GP3** the title compound **12d** was obtained as a white solid (51 mg, 79 %). **^{1}H-NMR** (400 MHz, DMSO-d$_6$): δ 13.45 (br s, 1H), 8.13 (s, 1H), 7.94 – 7.90 (m, 2H), 7.85 – 7.81 (m, 2H), 3.41 (s, 3H) ppm. **^{13}C{^{1}H}-NMR** (101 MHz, DMSO-d$_6$): δ 177.3, 155.5, 145.0, 138.9, 132.1 (2C), 130.2 (2C), 128.0, 114.8, 33.1 ppm. **HR-MS (ESI)** m/z calc. for $C_{11}H_{10}BrN_2O_3S_2^+$ [M+H]$^+$: 360.9311, found: 360.9309.

5-(4-Iodobenzenesulfonyl)-3-methyl-2-sulfanylidene-1,2,3,4-tetrahydropyrimidin-4-one (12e)

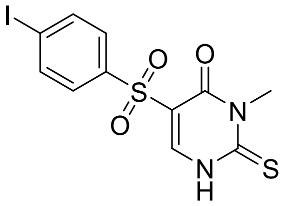

Following **GP3** the title compound **12e** was obtained as a white solid (84 mg, 82 %). **^{1}H-NMR** (400 MHz, DMSO-d$_6$): δ 13.44 (br s, 1H), 8.12 (s, 1H), 8.02 – 7.98 (m, 2H), 7.76 – 7.72 (m, 2H), 3.41 (s, 3H) ppm. **^{13}C{^{1}H}-NMR** (101 MHz, DMSO-d$_6$): δ 177.3, 155.4, 144.9, 139.3, 137.9 (2C), 129.8 (2C), 114.9, 102.6, 33.1 ppm. **HR-MS (ESI)** m/z calc. for $C_{11}H_{10}IN_2O_3S_2^+$ [M+H]$^+$: 408.9173, found: 408.9159.

5-(3-Chlorobenzenesulfonyl)-3-methyl-2-sulfanylidene-1,2,3,4-tetrahydropyrimidin-4-one (12f)

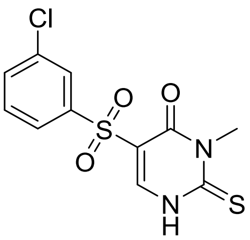

Following **GP3** the title compound **12f** was obtained as a white solid (66 mg, 83 %). **^{1}H-NMR** (400 MHz, DMSO-d$_6$): δ 13.48 (br s, 1H), 8.15 (s, 1H), 8.02 (t, J = 1.9 Hz, 1H), 7.99 – 7.93 (m, 1H), 7.82 – 7.74 (m, 1H), 7.65 (t, J = 8.0 Hz, 1H), 3.42 (s, 3H) ppm. **^{13}C{^{1}H}-NMR** (101 MHz, DMSO-d$_6$): δ

177.8, 156.0, 145.8, 142.1, 134.2, 134.0, 131.5, 128.2, 127.4, 114.9, 33.6 ppm. **HR-MS (ESI)** m/z calc. for $C_{11}H_{10}ClN_2O_3S_2^+$ [M+H]$^+$: 316.9816, found: 316.9814.

5-(2-Chlorobenzenesulfonyl)-3-methyl-2-sulfanylidene-1,2,3,4-tetrahydropyrimidin-4-one (12g)

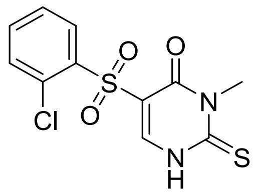

Following **GP3** the title compound **12g** was obtained as a white solid (54 mg, 68 %). **^{1}H-NMR** (400 MHz, DMSO-d$_6$): δ 13.55 (br s, 1H), 8.24 – 8.20 (m, 2H), 7.76 – 7.67 (m, 1H), 7.64 (td, *J* = 8.1, 7.6, 1.3 Hz, 2H), 3.39 (s, 3H) ppm. **^{13}C{^{1}H}-NMR** (101 MHz, DMSO-d$_6$): δ 177.7, 155.7, 146.7, 136.6, 136.0, 133.0, 132.3, 131.6, 128.3, 114.1, 33.6 ppm. **HR-MS (ESI)** m/z calc. for $C_{11}H_{10}ClN_2O_3S_2^+$ [M+H]$^+$: 316.9816, found: 316.9822.

3-Methyl-5-(4-nitrobenzenesulfonyl)-2-sulfanylidene-1,2,3,4-tetrahydropyrimidin-4-one (12h)

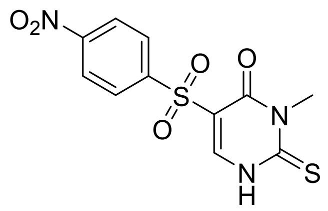

Following **GP3** the title compound **12h** was obtained as a brown solid (72 mg, 88 %). **^{1}H-NMR** (400 MHz, DMSO-d$_6$): δ 13.53 (br s, 1H), 8.41 (d, *J* = 8.7 Hz, 2H), 8.26 (d, *J* = 8.7 Hz, 2H), 8.20 (s, 1H), 3.40 (s, 3H) ppm. **^{13}C{^{1}H}-NMR** (101 MHz, DMSO-d$_6$): δ 177.4, 155.5, 150.4, 145.7, 145.0, 129.9 (2C), 124.2 (2C), 113.9, 33.1 ppm. **HR-MS (ESI)** m/z calc. for $C_{11}H_9N_3NaO_5S_2^+$ [M+Na]$^+$: 349.9876, found: 349.9879.

3-Methyl-2-sulfanylidene-5-[4-(trifluoromethyl)benzenesulfonyl]-1,2,3,4-tetrahydropyrimidin-4-one (12i)

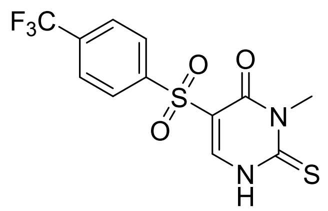

Following **GP3** the title compound **12i** was obtained as a yellow solid (87 mg, 98 %). **^{1}H-NMR** (400 MHz, DMSO-d$_6$): δ 13.50 (br s, 1H), 8.22 (d, *J* = 8.2 Hz, 2H), 8.18 (s, 1H), 8.00 (d, *J* = 8.1 Hz, 2H), 3.41 (s, 3H) ppm. **^{13}C{^{1}H}-NMR** (101 MHz, DMSO-d$_6$): δ 157.8, 150.5, 147.5, 144.2, 133.0 (q, *J* = 32.2 Hz),

129.0 (2C), 126.2 (q, J = 3.7 Hz, 2C), 123.4 (q, J = 273.9 Hz), 111.1, 26.8 ppm. **^{19}F-NMR** (376 MHz, DMSO-d$_6$): δ -61.70 (s) ppm. **HR-MS (ESI)** m/z calc. for $C_{12}H_{10}F_3N_2O_3S_2^+$ [M+H]$^+$: 351.0080, found: 351.0071.

5-(4-Methoxybenzenesulfonyl)-3-methyl-2-sulfanylidene-1,2,3,4-tetrahydropyrimidin-4-one (12j)

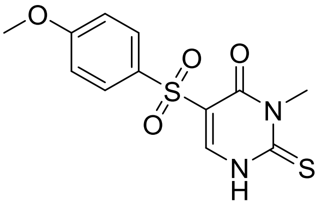

Following **GP3** the title compound **12j** was obtained and purified with preparative HPLC [acetonitrile (0.05 % trifluoroacetic acid)/water (0.05 % trifluoroacetic acid) = 5:95 – 95:5] as a white solid (24 mg, 31 %). **^{1}H-NMR** (400 MHz, DMSO-d$_6$): δ 13.37 (br s, 1H), 8.10 (s, 1H), 7.97 – 7.88 (m, 2H), 7.16 – 7.08 (m, 2H), 3.85 (s, 3H), 3.43 (s, 3H) ppm. **^{13}C{^{1}H}-NMR** (101 MHz, DMSO-d$_6$): δ 177.7, 163.8, 155.9, 144.5, 131.5, 131.2 (2C), 116.6, 114.7 (2C), 56.3, 33.6 ppm. **HR-MS (ESI)** m/z calc. for $C_{12}H_{13}N_2O_4S_2^+$ [M+H]$^+$: 313.0312, found: 313.0309.

5-(4-Methoxybenzenesulfonyl)-1-methyl-2-sulfanylidene-1,2,3,4-tetrahydropyrimidin-4-one (S5)

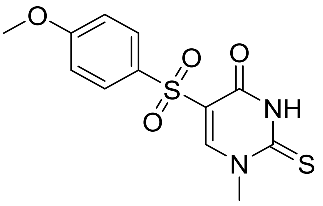

Following **GP3** the title compound **S5** was obtained and purified with preparative HPLC [acetonitrile (0.05 % trifluoroacetic acid)/water (0.05 % trifluoroacetic acid) = 5:95 – 95:5] as a white solid (15 mg, 18 %). **^{1}H-NMR** (400 MHz, DMSO-d$_6$): δ 13.08 (br s, 1H), 8.70 (s, 1H), 7.93 – 7.87 (m, 2H), 7.17 – 7.11 (m, 2H), 3.86 (s, 3H), 3.69 (s, 3H) ppm. **^{13}C{^{1}H}-NMR** (101 MHz, DMSO-d$_6$): δ 178.2, 163.8, 155.6, 151.0, 131.7, 131.0 (2C), 117.8, 114.7 (2C), 56.3, 43.4 ppm. **HR-MS (ESI)** m/z calc. for $C_{12}H_{13}N_2O_4S_2^+$ [M+H]$^+$: 313.0312, found: 313.0309.

3-Methyl-5-(4-methylbenzenesulfonyl)-2-sulfanylidene-1,2,3,4-tetrahydropyrimidin-4-one (12k)

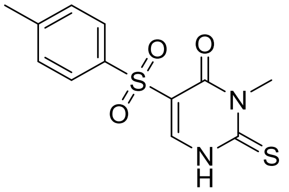

Following **GP3** the title compound **12k** was obtained as a yellow solid (57 mg, 77 %). **^{1}H-NMR** (400 MHz, DMSO-d$_6$): δ 13.39 (br s, 1H), 8.11 (s, 1H), 7.90 – 7.83 (m, 2H), 7.40 (d, J = 8.1 Hz,

2H), 3.41 (s, 3H), 2.38 (s, 3H) ppm. **^{13}C{^{1}H}-NMR** (101 MHz, DMSO-d$_6$): δ 177.3, 155.4, 144.4, 144.4, 136.8, 129.4 (2C), 128.2 (2C), 115.6, 33.1, 21.1 ppm. **HR-MS (ESI)** m/z calc. for $C_{12}H_{13}N_2O_3S_2^+$ [M+H]$^+$: 297.0363, found: 297.0363.

3-Methyl-5-(pyridine-2-sulfonyl)-2-sulfanylidene-1,2,3,4-tetrahydropyrimidin-4-one (12n)

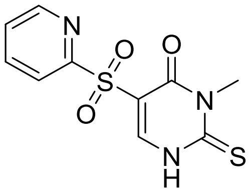

Following **GP3** the title compound **12n** was obtained as a white solid (42 mg, 58 %). **^{1}H-NMR** (400 MHz, DMSO-d$_6$): δ 13.52 (br s, 1H), 8.69 (dt, *J* = 4.7, 1.4 Hz, 1H), 8.19 (s, 2H), 8.23 – 8.11 (m, 1H), 7.76 – 7.68 (m, 1H), 3.40 (s, 3H) ppm. **^{13}C{^{1}H}-NMR** (101 MHz, DMSO-d$_6$): δ 177.3, 156.6, 155.5, 150.1, 145.7, 138.8, 128.0, 122.9, 113.1, 33.1 ppm. **HR-MS (ESI)** m/z calc. for $C_{10}H_{10}N_3O_3S_2^+$ [M+H]$^+$: 284.0159, found: 284.0159.

5-Methanesulfonyl-3-methyl-2-sulfanylidene-1,2,3,4-tetrahydropyrimidin-4-one (12r)

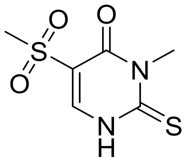

Following **GP3** the title compound **12r** was obtained and purified with preparative HPLC [acetonitrile (0.05 % trifluoroacetic acid)/water (0.05 % trifluoroacetic acid) = 5:95 – 95:5] as a white solid (26 mg, 47 %). **^{1}H-NMR** (400 MHz, DMSO-d$_6$): δ 13.25 (br s, 1H), 8.52 (s, 1H), 3.67 (s, 3H), 3.19 (s, 3H) ppm. **^{13}C{^{1}H}-NMR** (101 MHz, DMSO-d$_6$): δ 178.2, 156.7, 150.7, 117.6, 43.2, 42.6 ppm. **HR-MS (ESI)** m/z calc. for $C_6H_9N_2O_3S_2^+$ [M+H]$^+$: 221.0049, found: 221.0075.

5-Methanesulfonyl-1-methyl-2-sulfanylidene-1,2,3,4-tetrahydropyrimidin-4-one (12s)

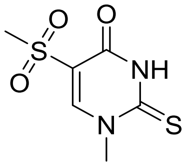

Following **GP3** the title compound **12s** was obtained and purified with preparative HPLC [acetonitrile (0.05 % trifluoroacetic acid)/water (0.05 % trifluoroacetic acid) = 5:95 – 95:5] as a white solid (22 mg, 40 %). **^{1}H-NMR** (400 MHz, DMSO-d$_6$): δ 13.32 (s, 1H), 7.86 (s, 1H), 3.54 (s, 3H), 3.19 (s, 3H) ppm. **^{13}C{^{1}H}-NMR** (101 MHz, DMSO-d$_6$): δ 177.3, 156.4, 143.8, 115.8, 41.9, 33.3 ppm. **HR-MS (ESI)** m/z calc. for $C_6H_9N_2O_3S_2^+$ [M+H]$^+$: 221.0049, found: 221.0071.

5-(Benzenesulfonyl)-3-phenyl-2-sulfanylidene-1,2,3,4-tetrahydropyrimidin-4-one (13)

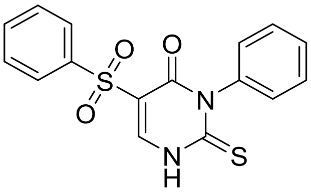

Following **GP3** the title compound **13** was obtained as a white solid (79 mg, 92 %). **^{1}H-NMR** (400 MHz, DMSO-d$_6$): *δ* 13.52 (br s, 1H), 8.22 (s, 1H), 8.00 – 7.96 (m, 2H), 7.72 – 7.68 (m, 1H), 7.60 (t, *J* = 7.7 Hz, 2H), 7.46 – 7.35 (m, 3H), 7.15 (dd, *J* = 7.3, 1.8 Hz, 2H) ppm. **^{13}C{^{1}H}-NMR** (101 MHz, DMSO-d$_6$): *δ* 178.4, 155.7, 145.5, 139.7, 138.1, 133.8, 129.2 (2C), 129.0 (2C), 128.5, 128.3 (2C), 128.1 (2C), 116.3 ppm. **HR-MS (ESI)** m/z calc. for $C_{16}H_{13}N_2O_4S2^+$ [M+H]$^+$: 345.0362, found: 345.0363.

3. NMR Spectra

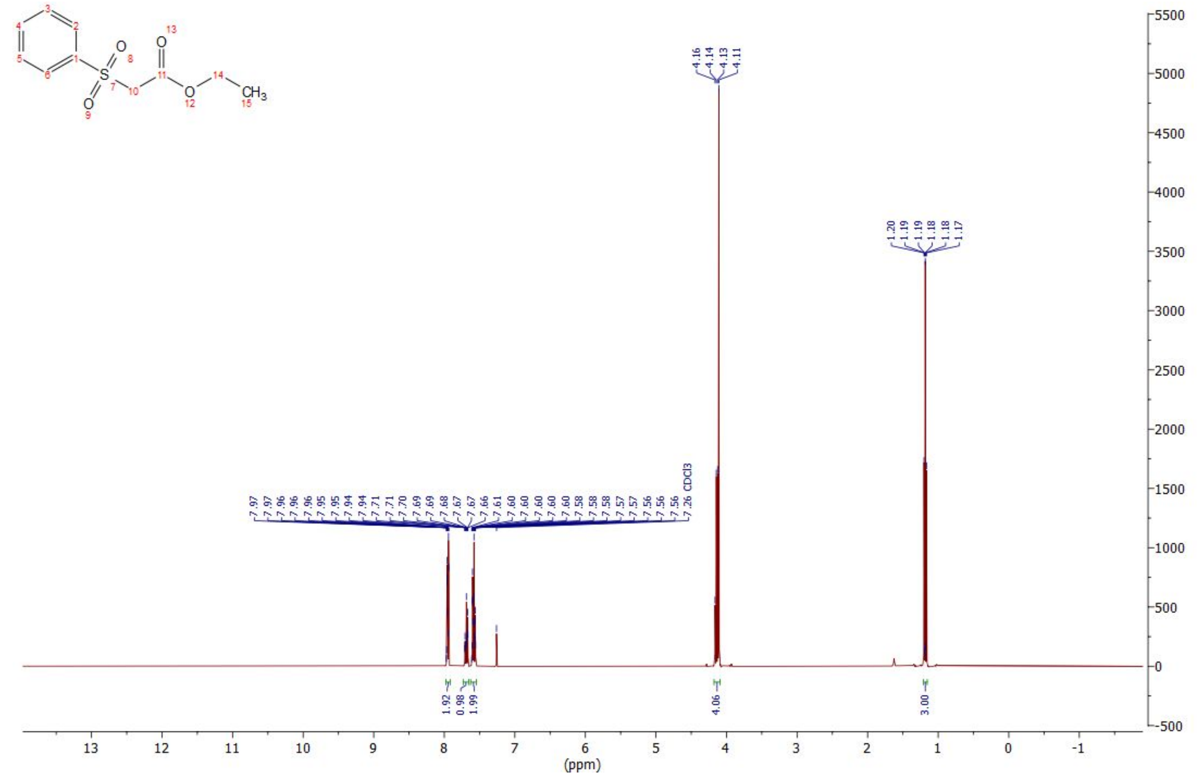

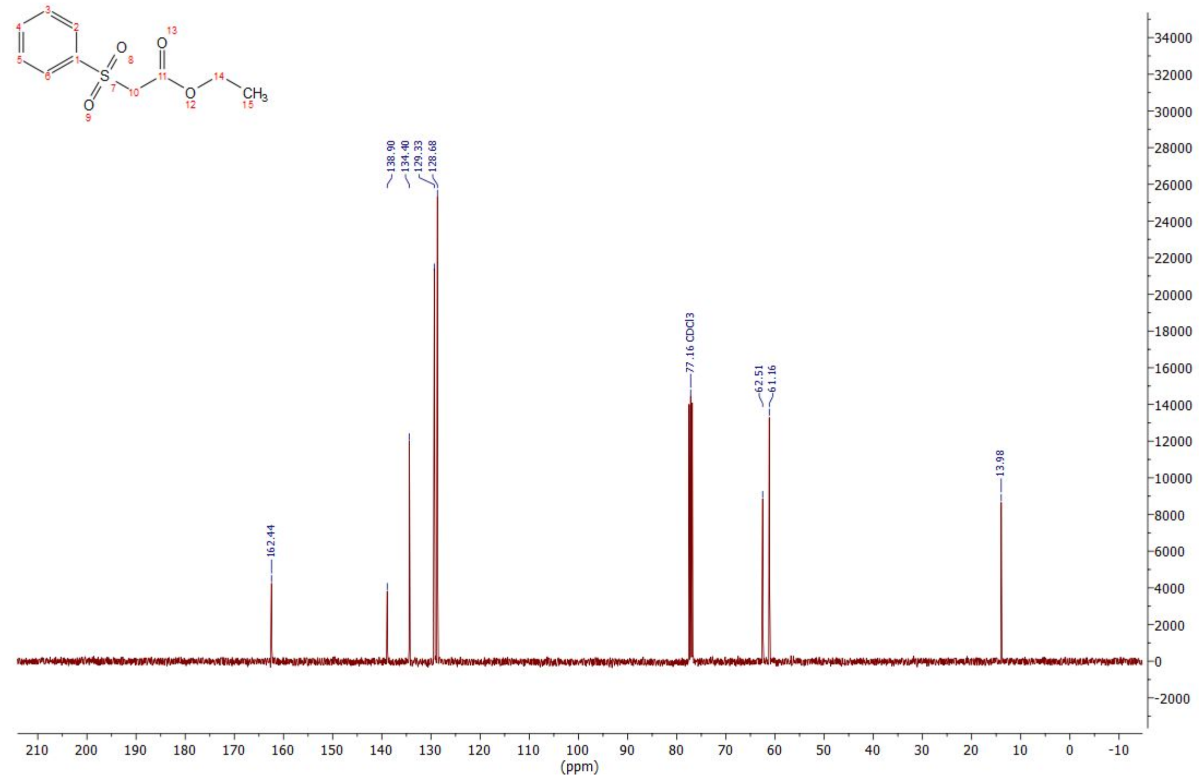

Figure S 1. *^{1}H (400 MHz) and ^{13}C{^{1}H} (101 MHz) NMR-Spectra in $CDCl_3$ of **1a**.*

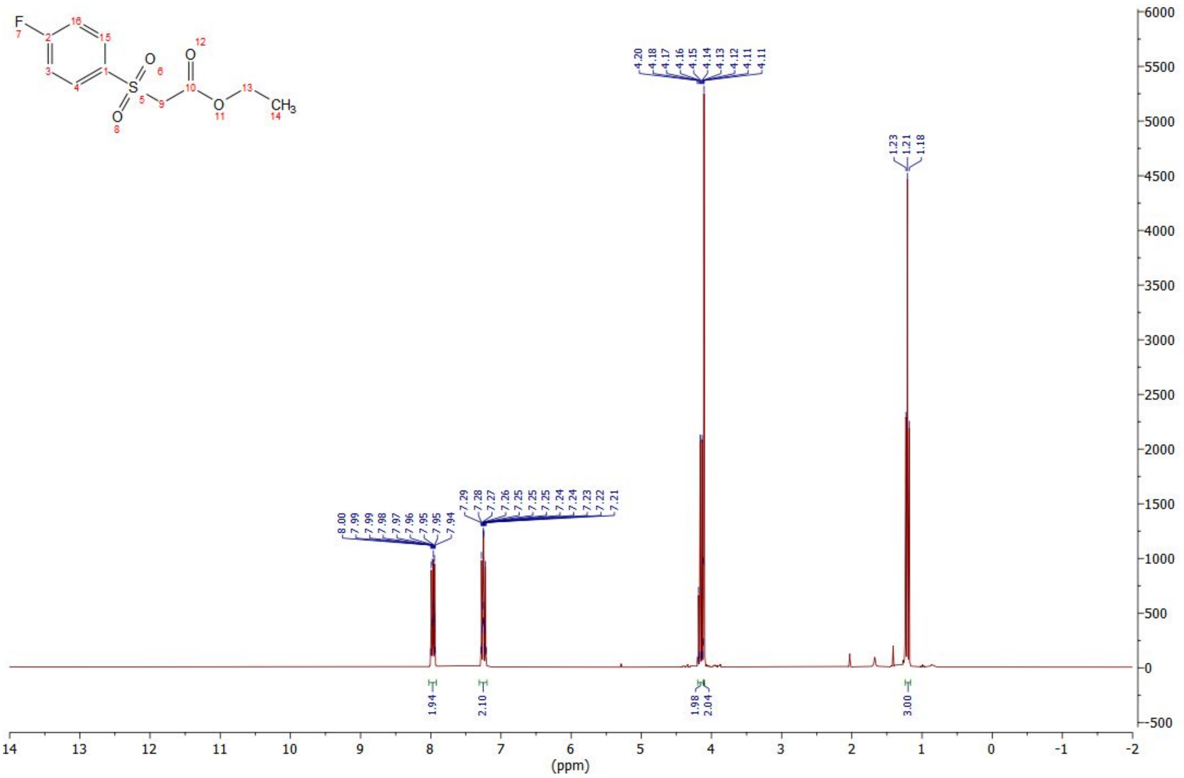

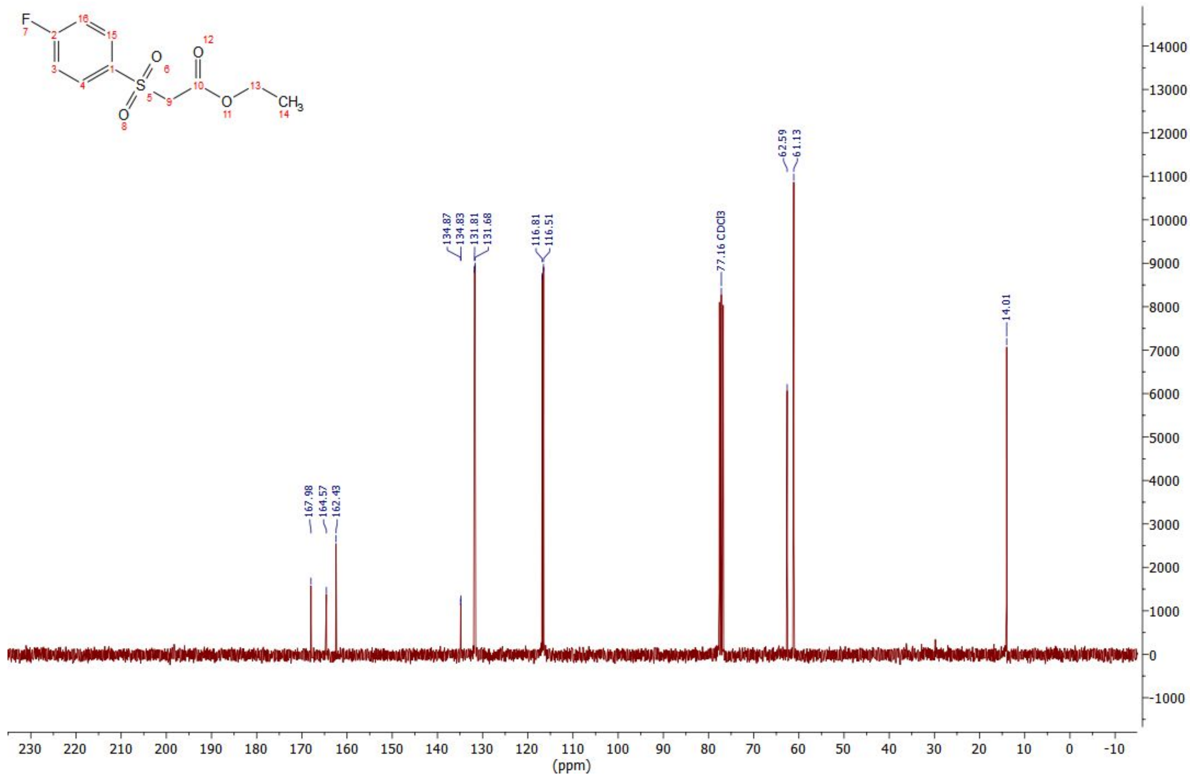

Figure S 2. *^{1}H (300 MHz) and ^{13}C{^{1}H} (75 MHz)NMR-Spectra in $CDCl_3$ of **1b**.*

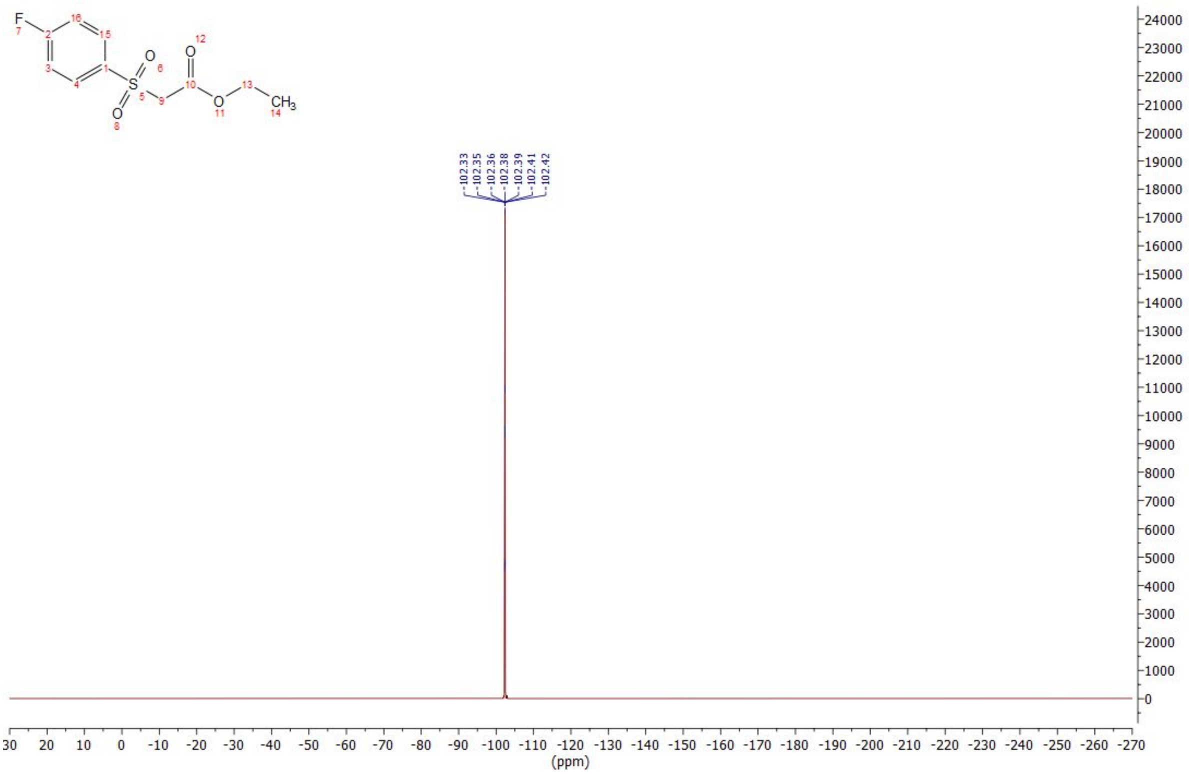

Figure S 3. *^{19}F NMR (282 MHz)-Spectra in $CDCl_3$ of **1b**.*

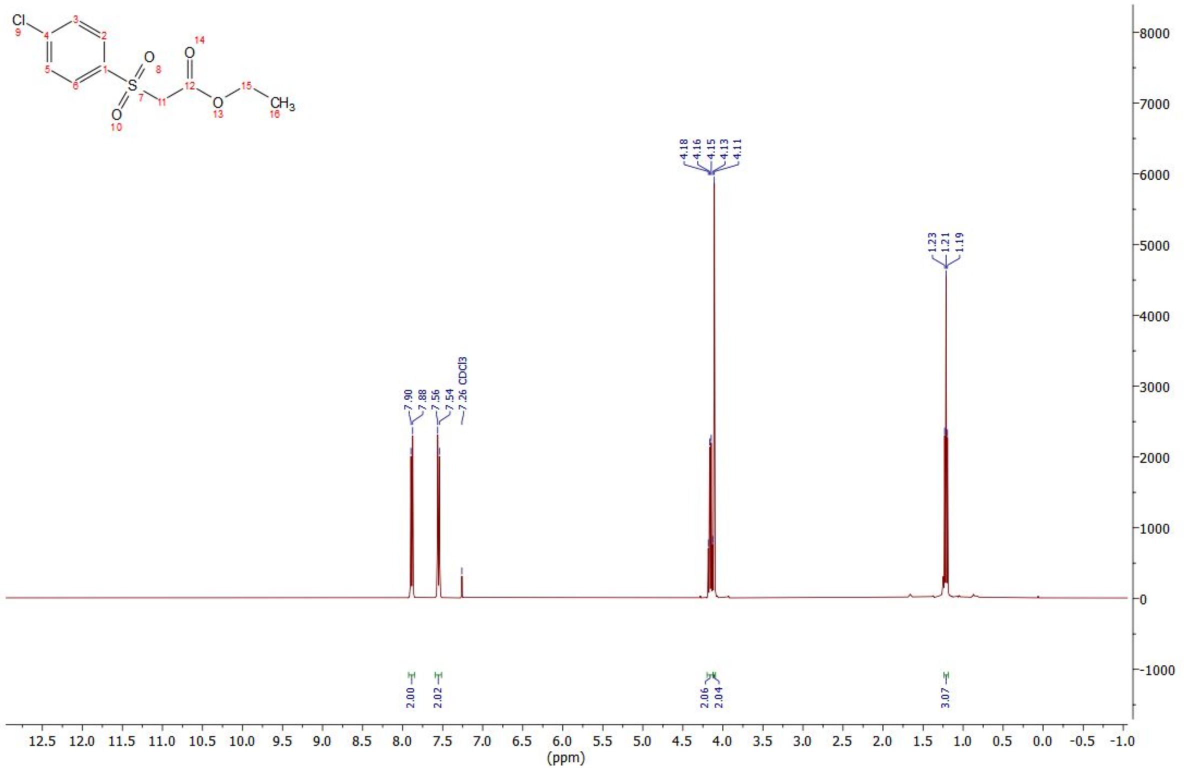

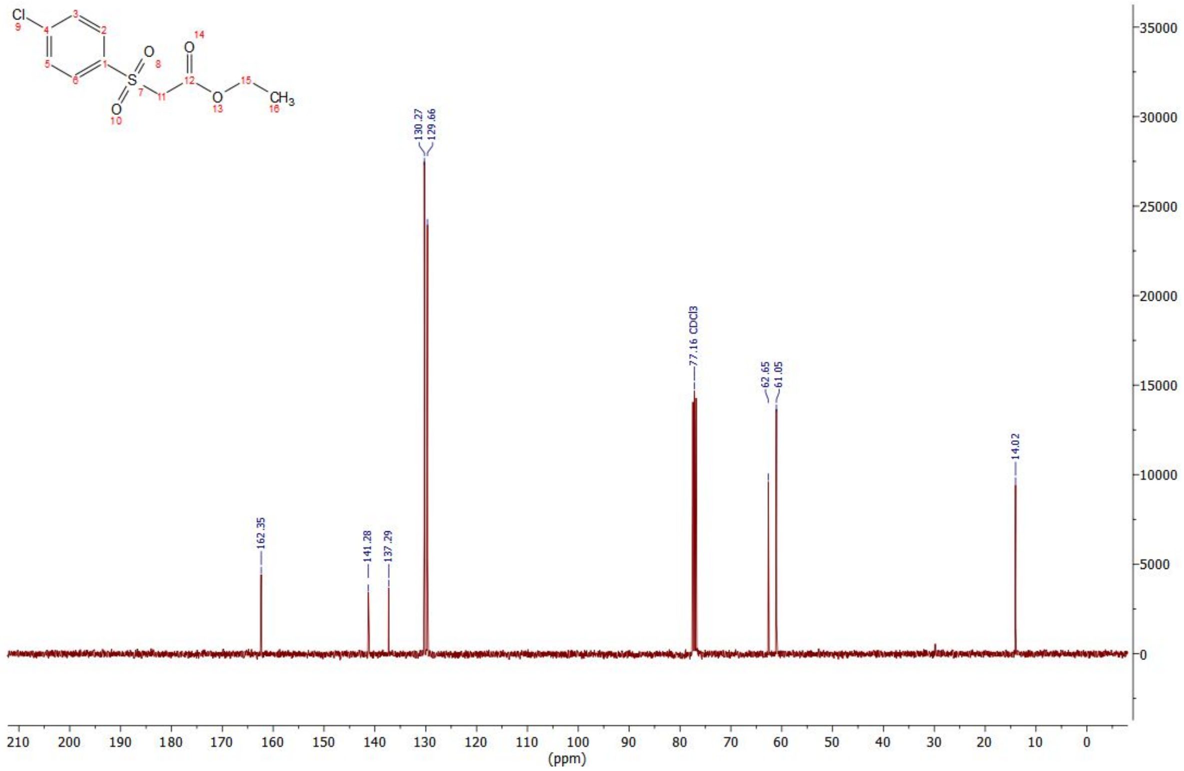

Figure S 4. *^{1}H (400 MHz) and $^{13}C\{^{1}H\}$ (101 MHz)NMR-Spectra in $CDCl_3$ of* ***1c***.

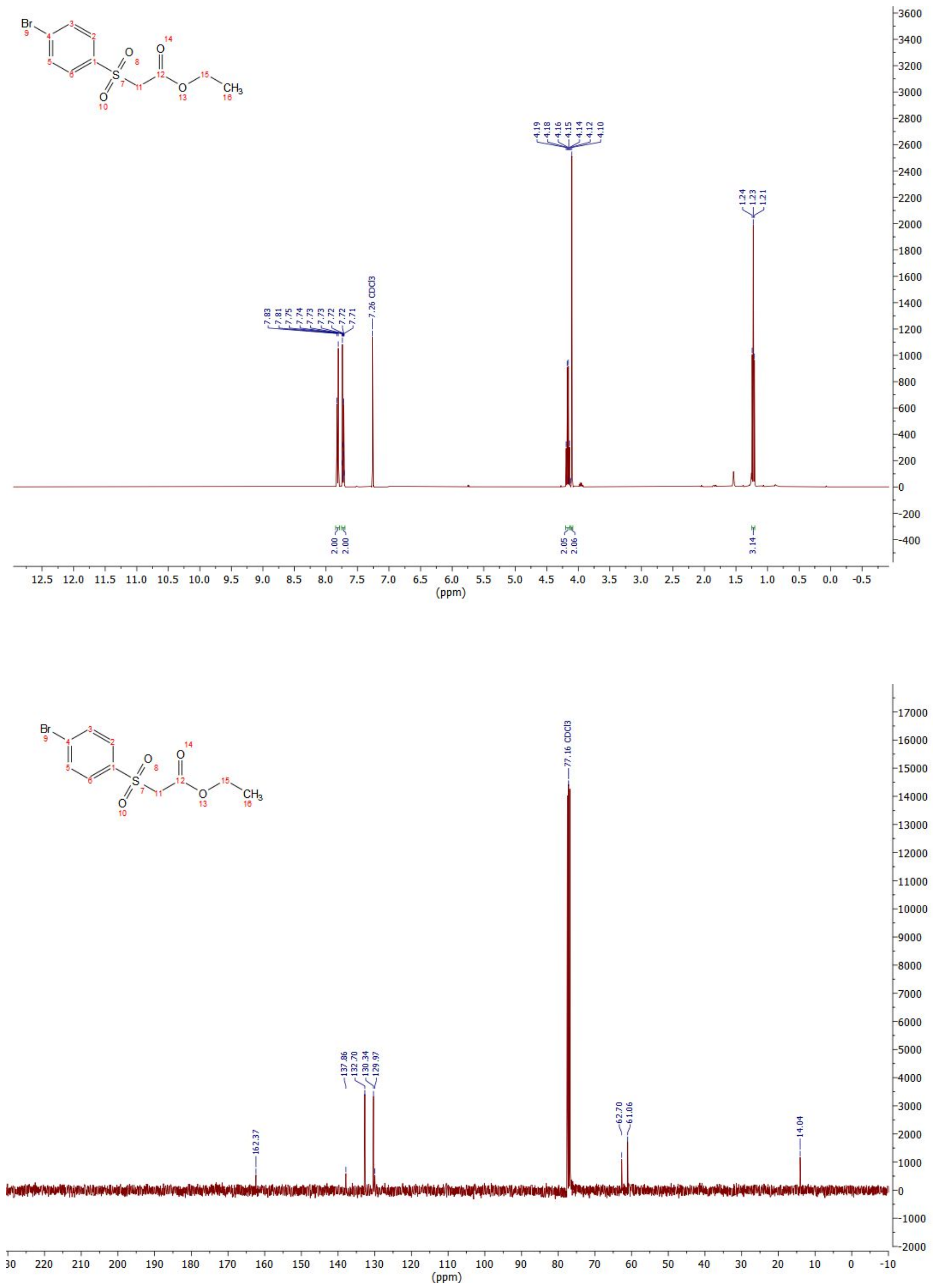

Figure S 5. *^{1}H (400 MHz) and $^{13}C\{^{1}H\}$ (101 MHz)NMR-Spectra in $CDCl_3$ of* ***1d****.*

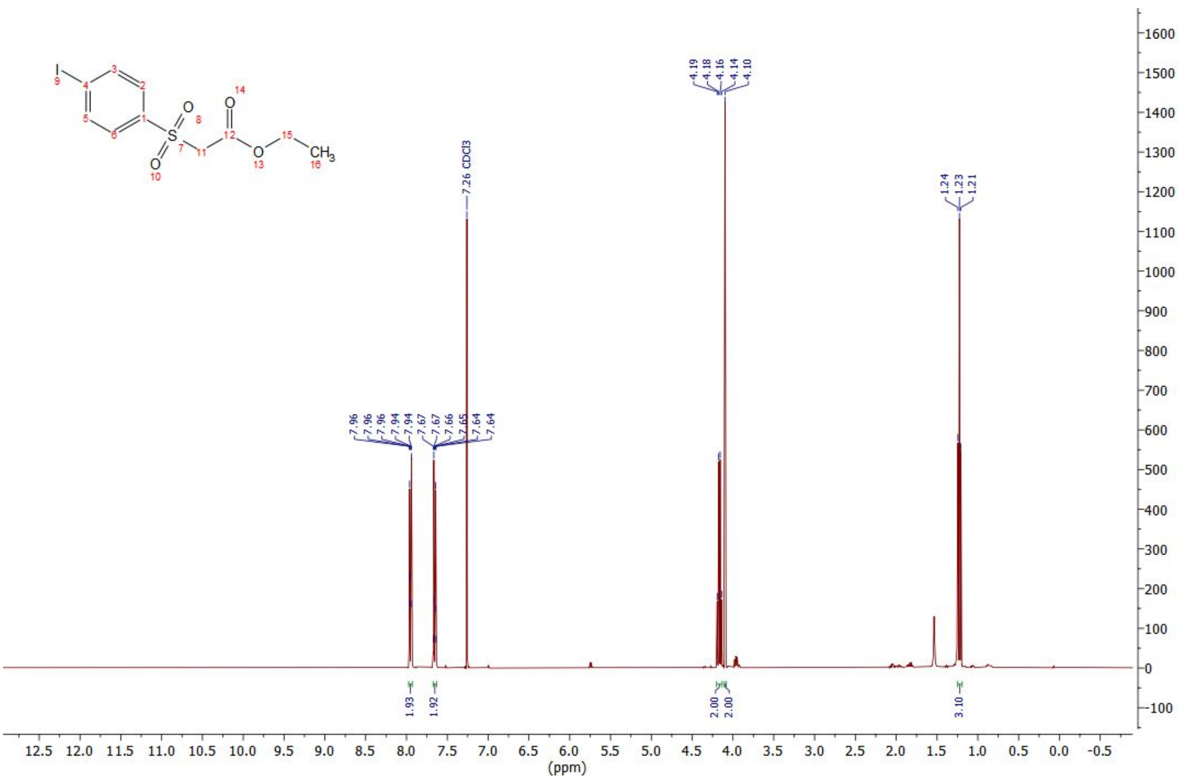

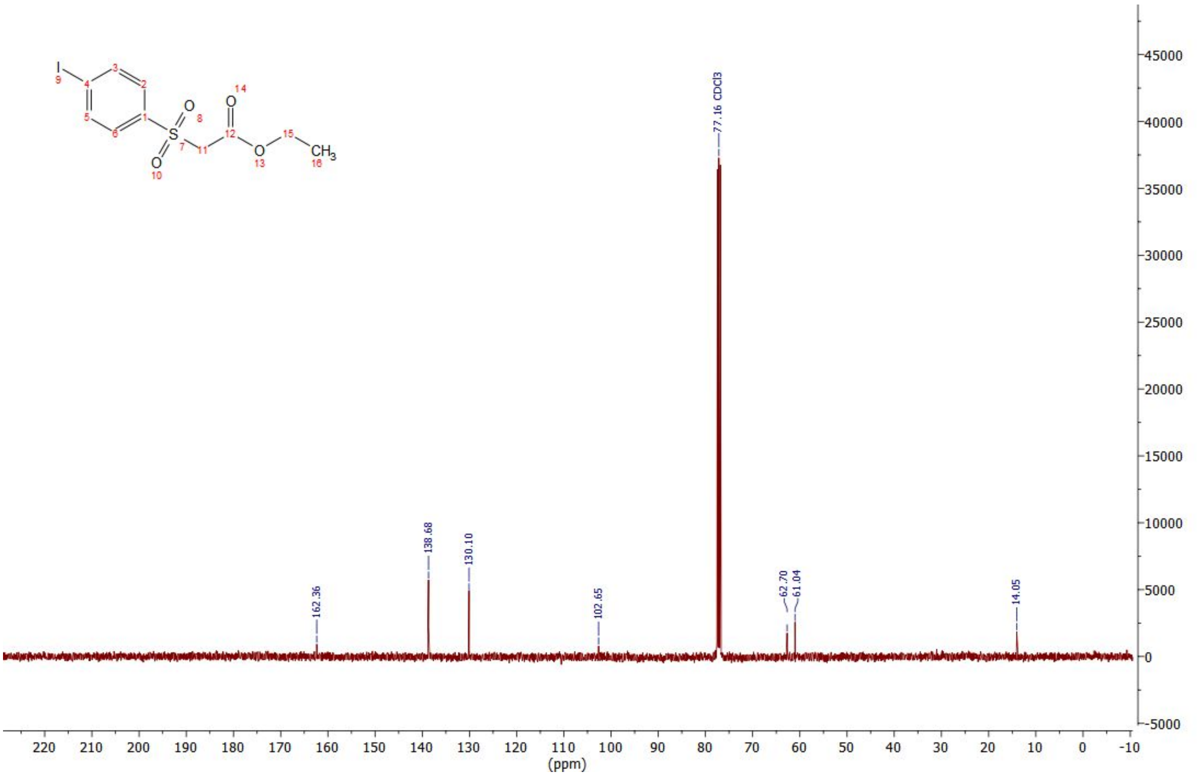

Figure S 6. *^{1}H (400 MHz) and $^{13}C\{^{1}H\}$ (101 MHz) NMR-Spectra in $CDCl_3$ of **1e**.*

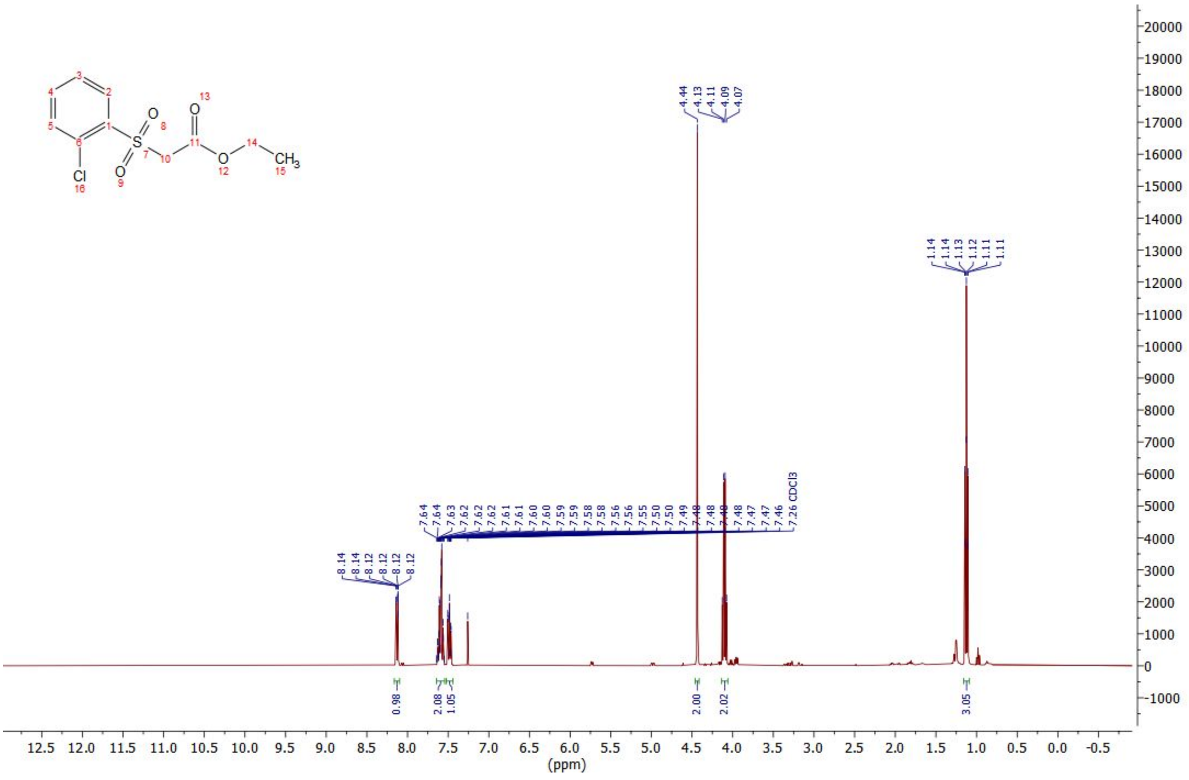

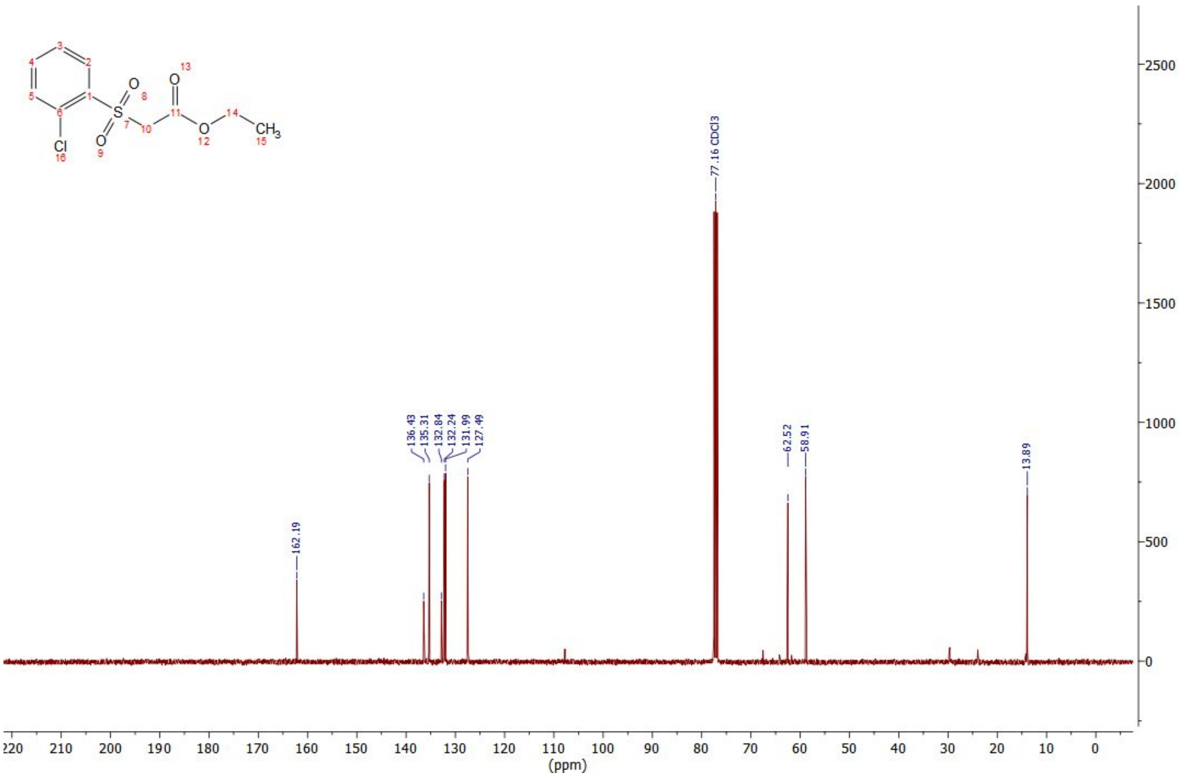

Figure S 7. *^{1}H (400 MHz) and $^{13}C\{^{1}H\}$ (101 MHz) NMR-Spectra in $CDCl_3$ of **1f**.*

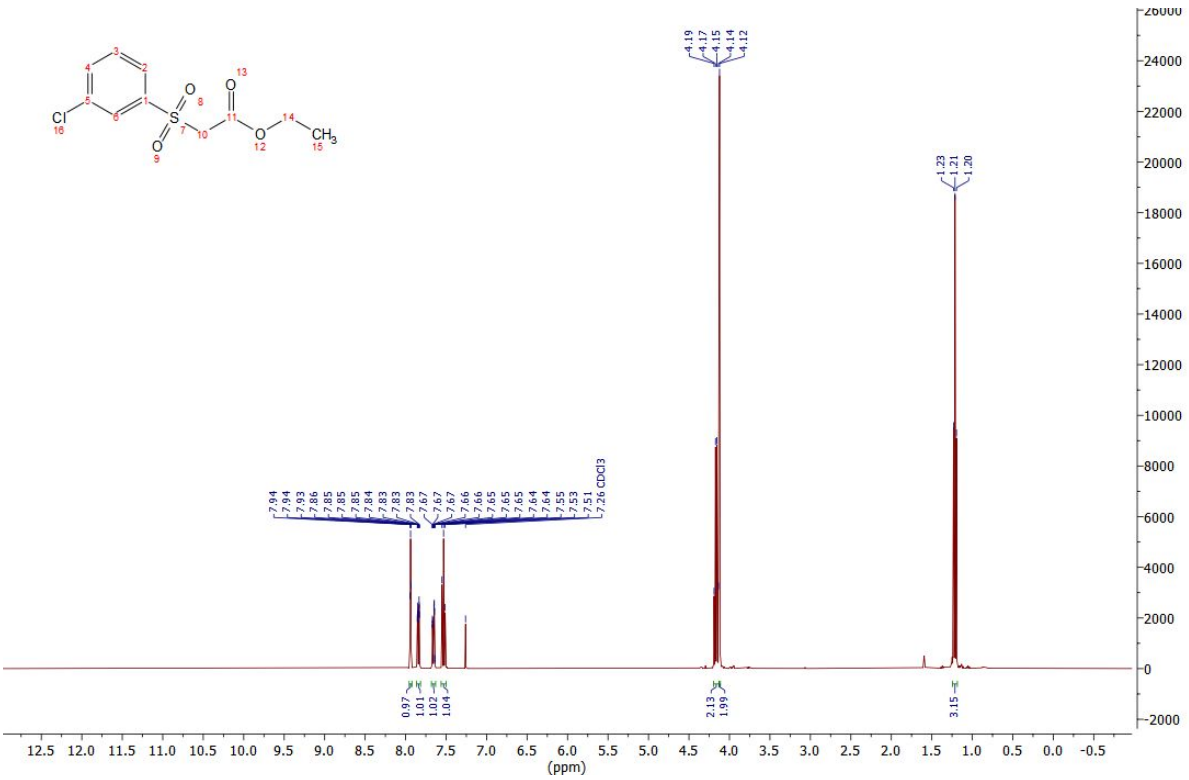

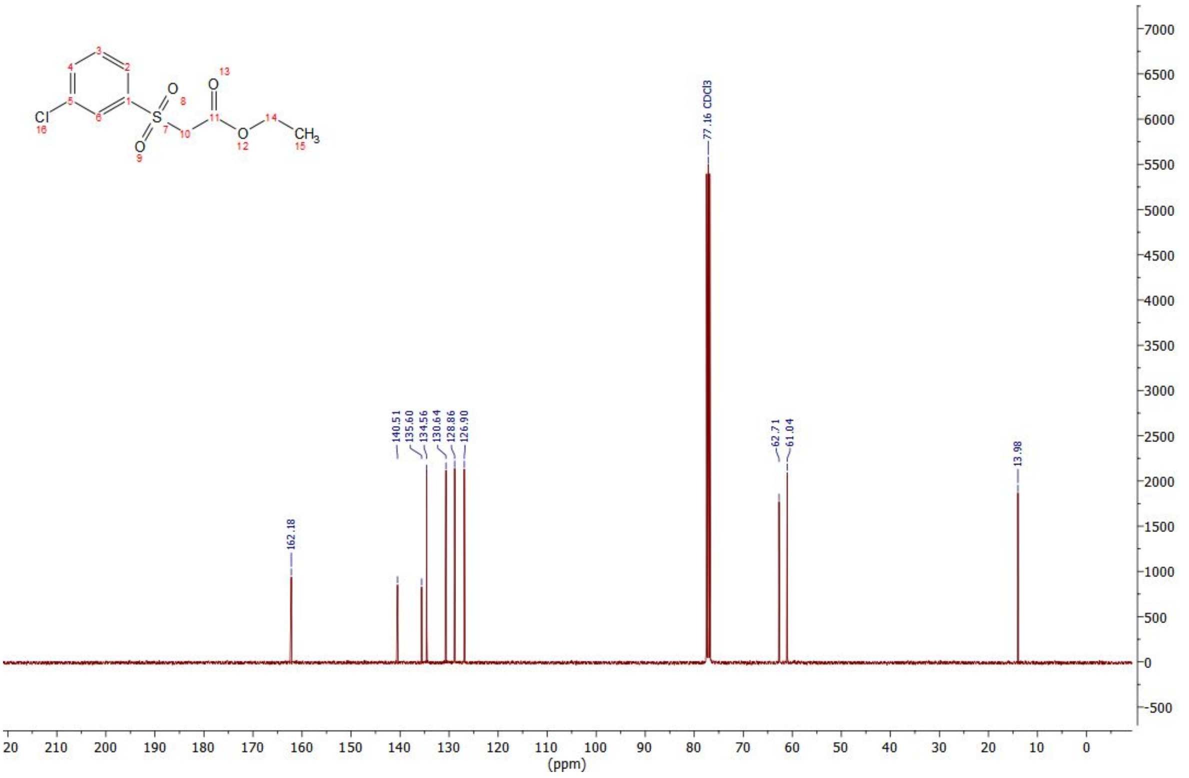

Figure S 8. *^{1}H (400 MHz) and ^{13}C{^{1}H} (101 MHz) NMR-Spectra in $CDCl_3$ of* ***1g****.*

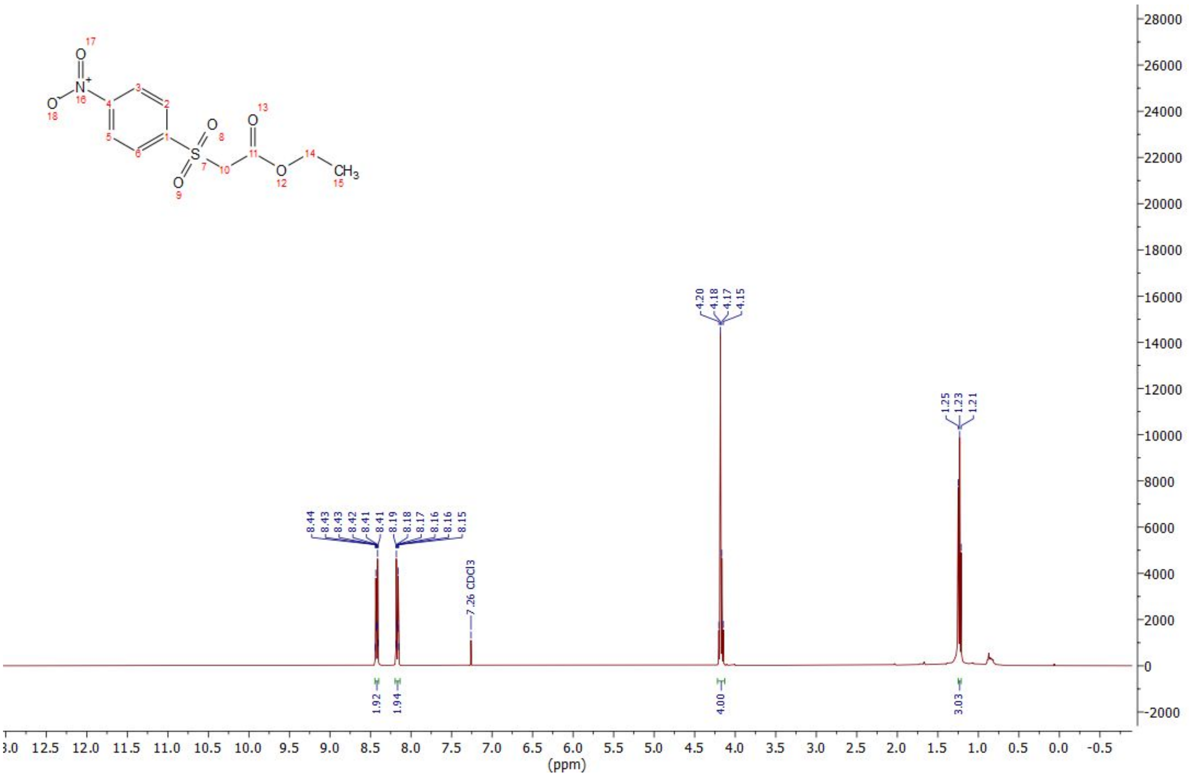

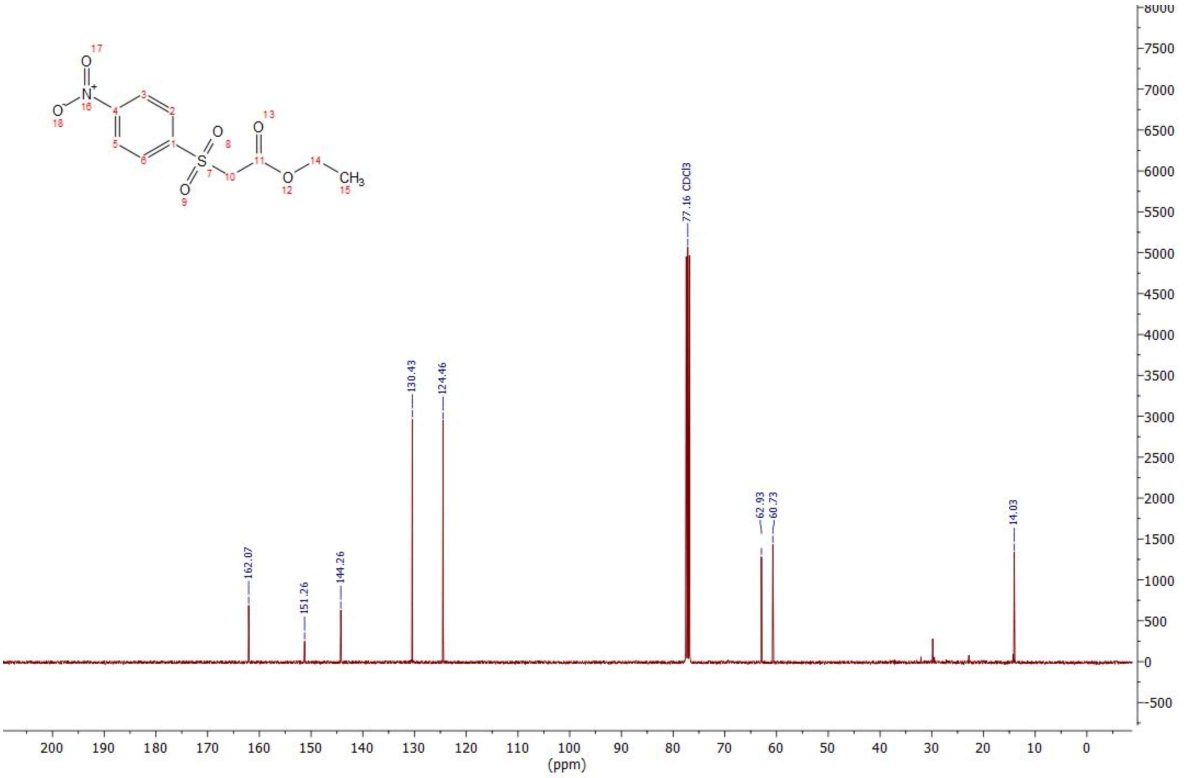

Figure S 9. *^{1}H (400 MHz) and $^{13}C\{^{1}H\}$ (101 MHz) NMR-Spectra in $CDCl_3$ of **1h**.*

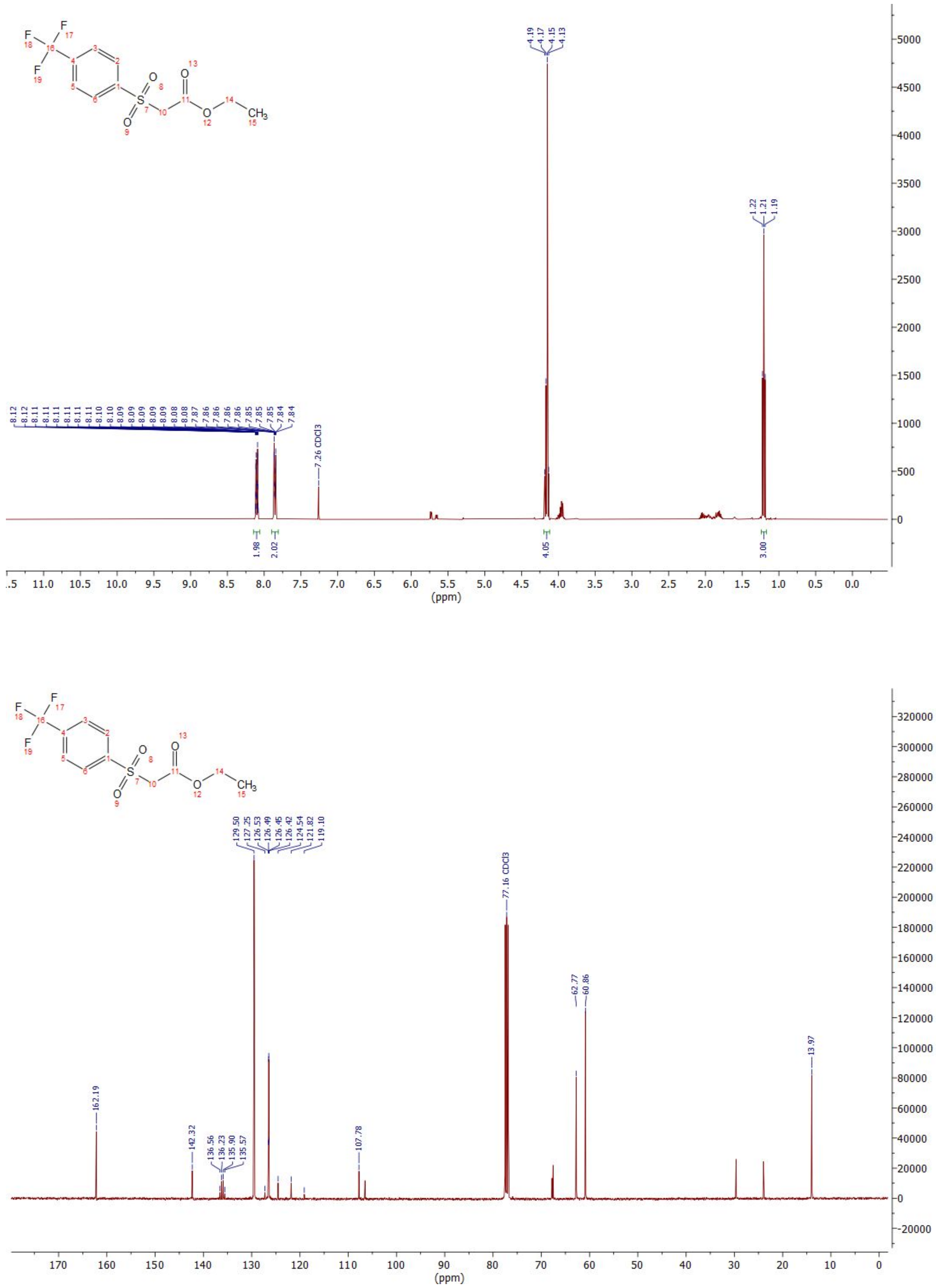

Figure S 10. *^{1}H (400 MHz) and ^{13}C{^{1}H} (101 MHz) NMR-Spectra in $CDCl_3$ of **1i**.*

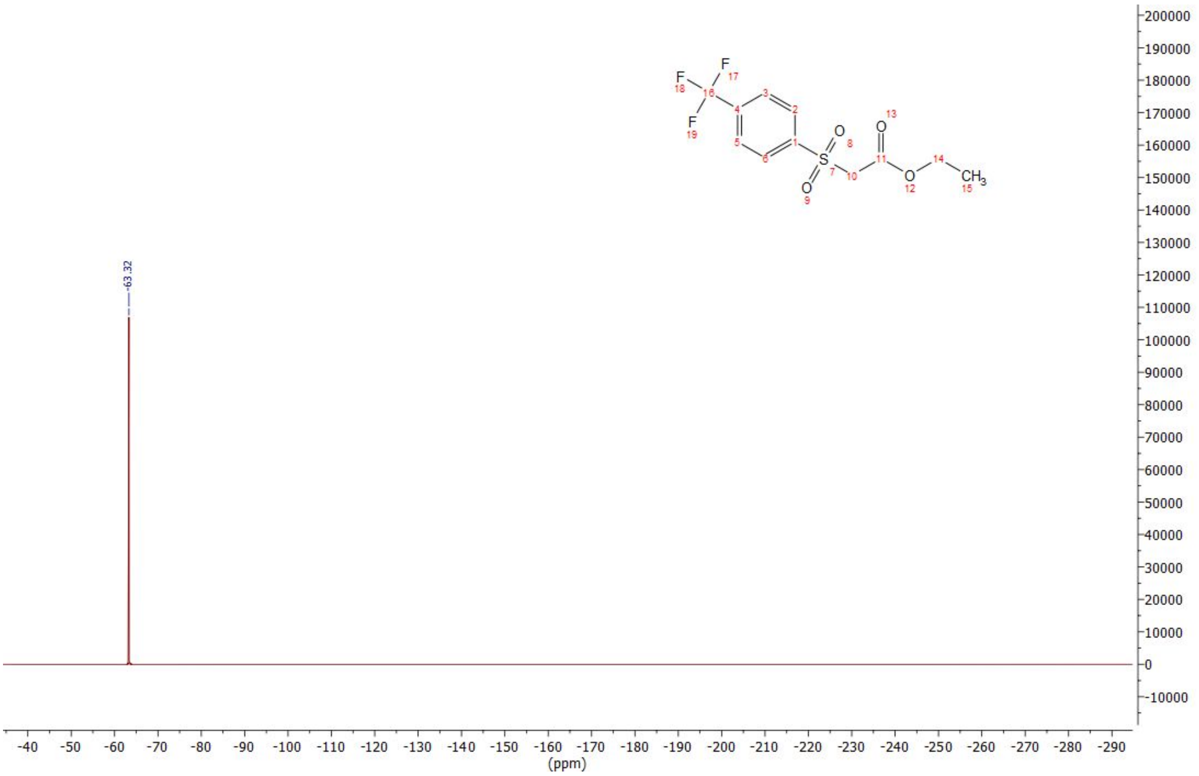

Figure S 11. *^{19}F NMR (376 MHz)-Spectra in $CDCl_3$ of **1i**.*

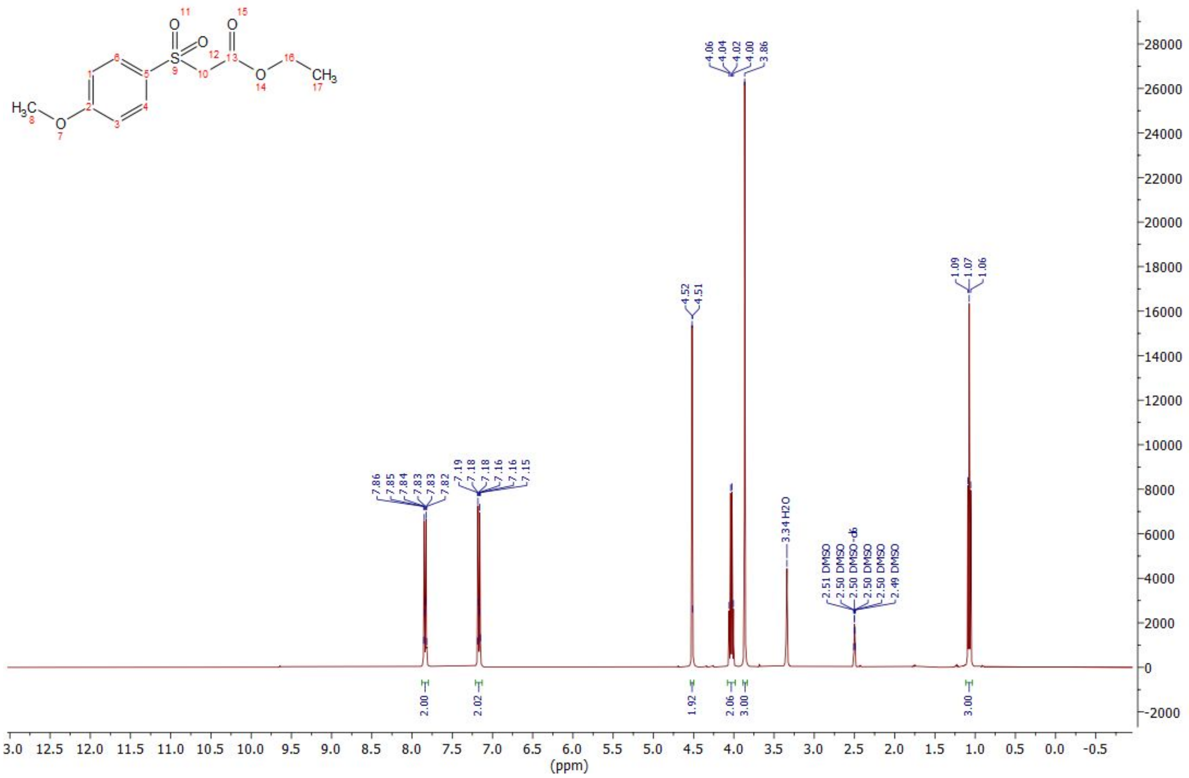

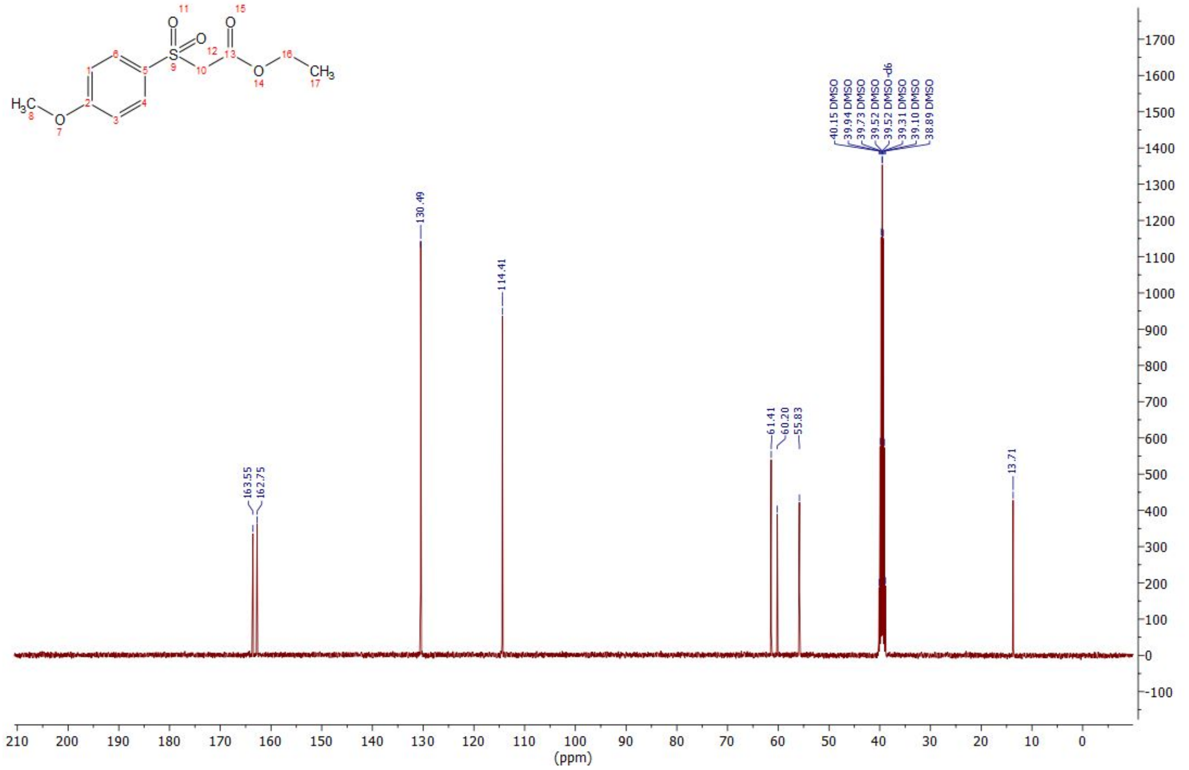

Figure S 12. *^{1}H (400 MHz) and $^{13}C\{^{1}H\}$ (101 MHz) NMR-Spectra in DMSO-d_6 of **1j**.*

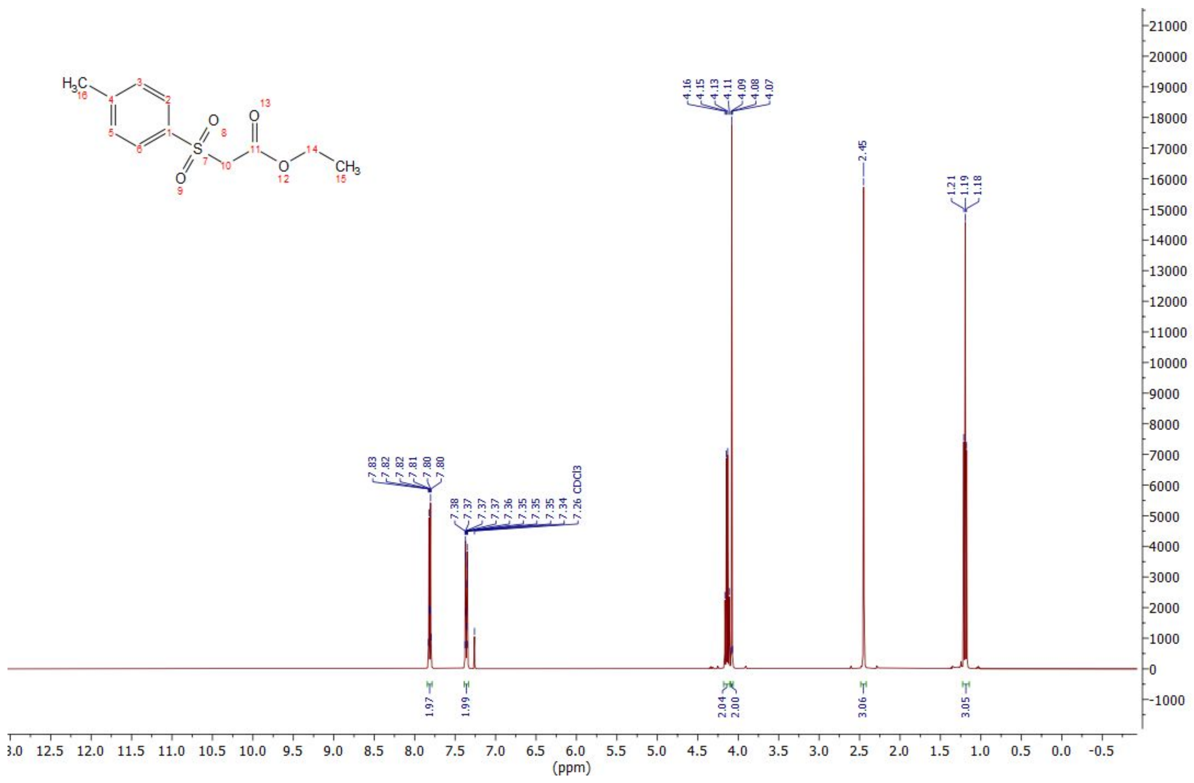

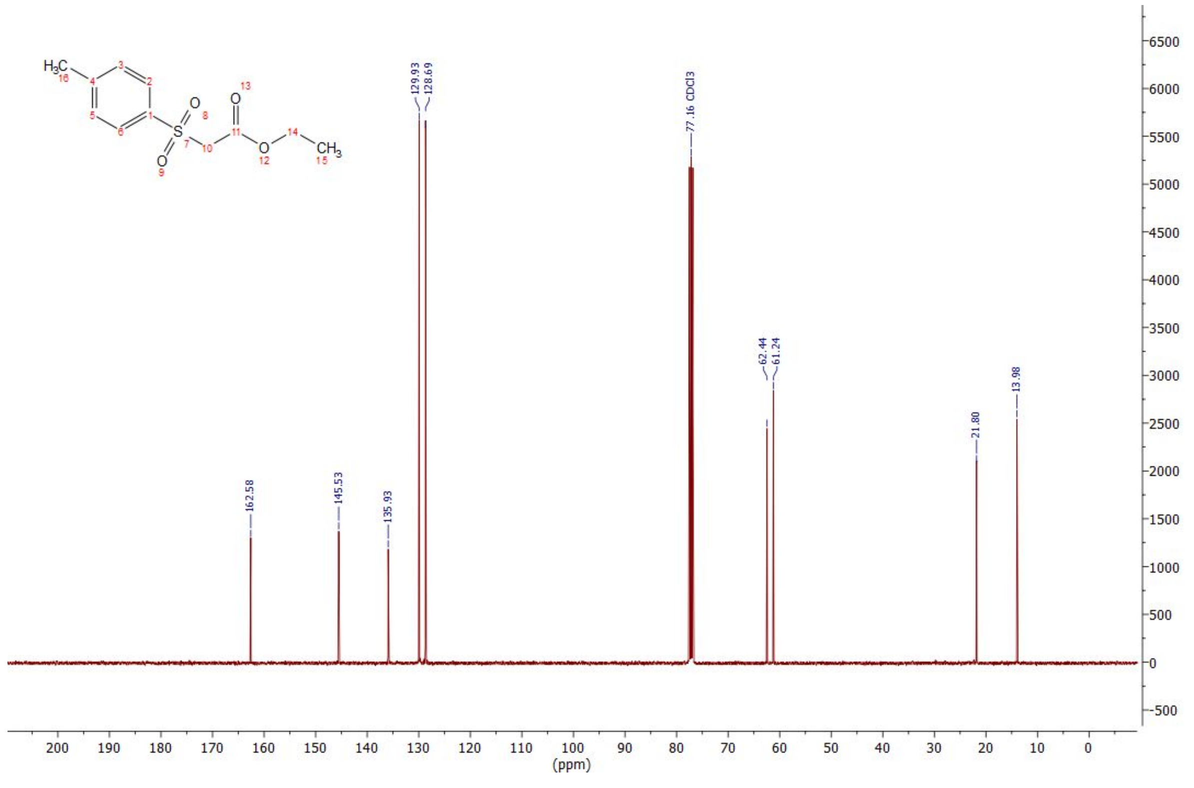

Figure S 13. *^{1}H (400 MHz) and ^{13}C{^{1}H} (101 MHz) NMR-Spectra in $CDCl_3$ of **1k**.*

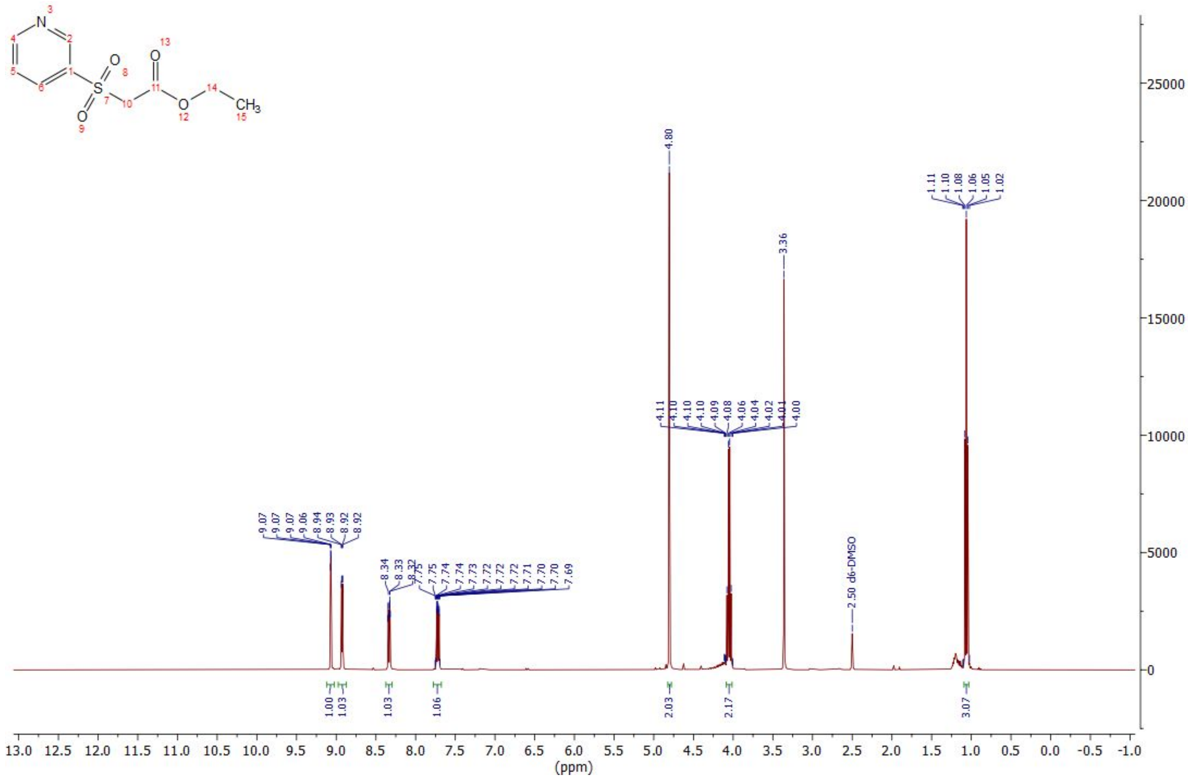

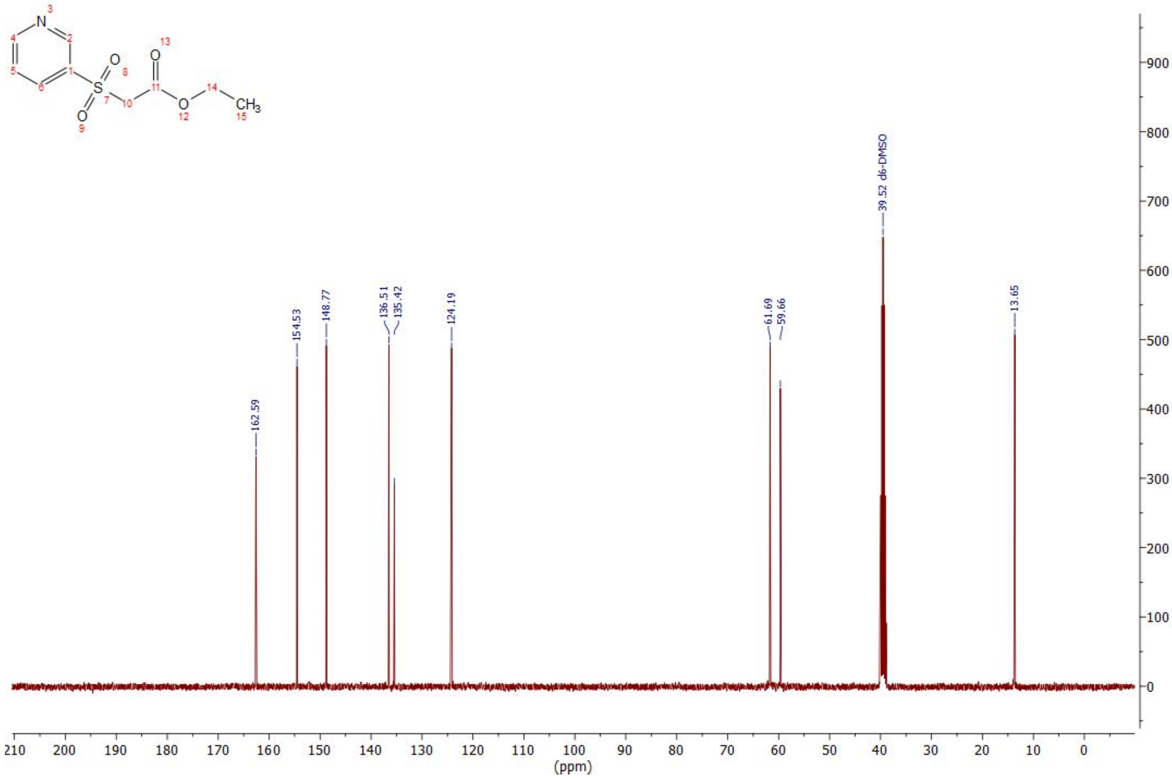

Figure S 14. *^{1}H (400 MHz)and $^{13}C\{^{1}H\}$ (101 MHz) NMR-Spectra in DMSO-d_6 of **1l**.*

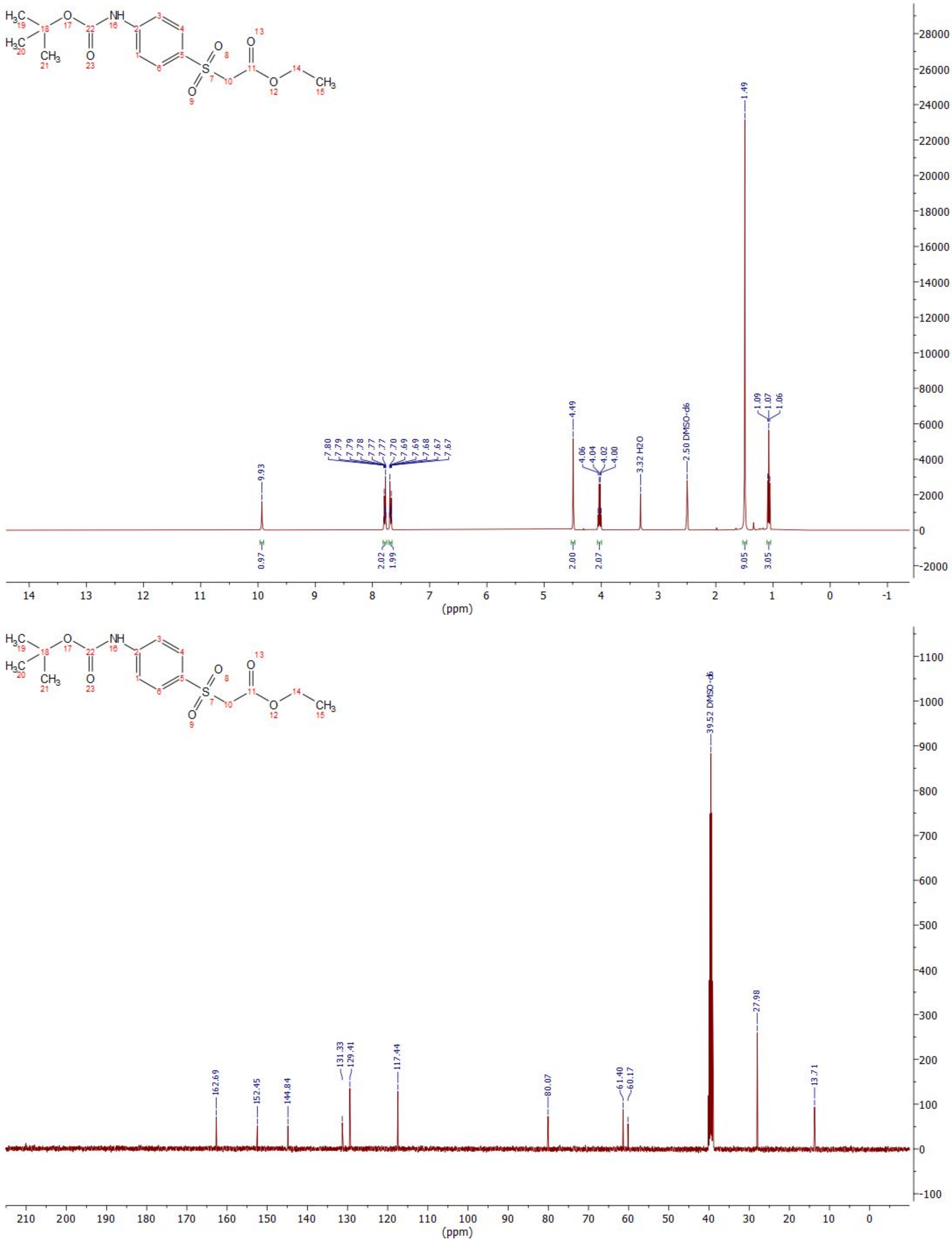

Figure S 15. *^{1}H (400 MHz) and ^{13}C{^{1}H} (101 MHz) NMR-Spectra in DMSO-d$_6$ of* ***1m****.*

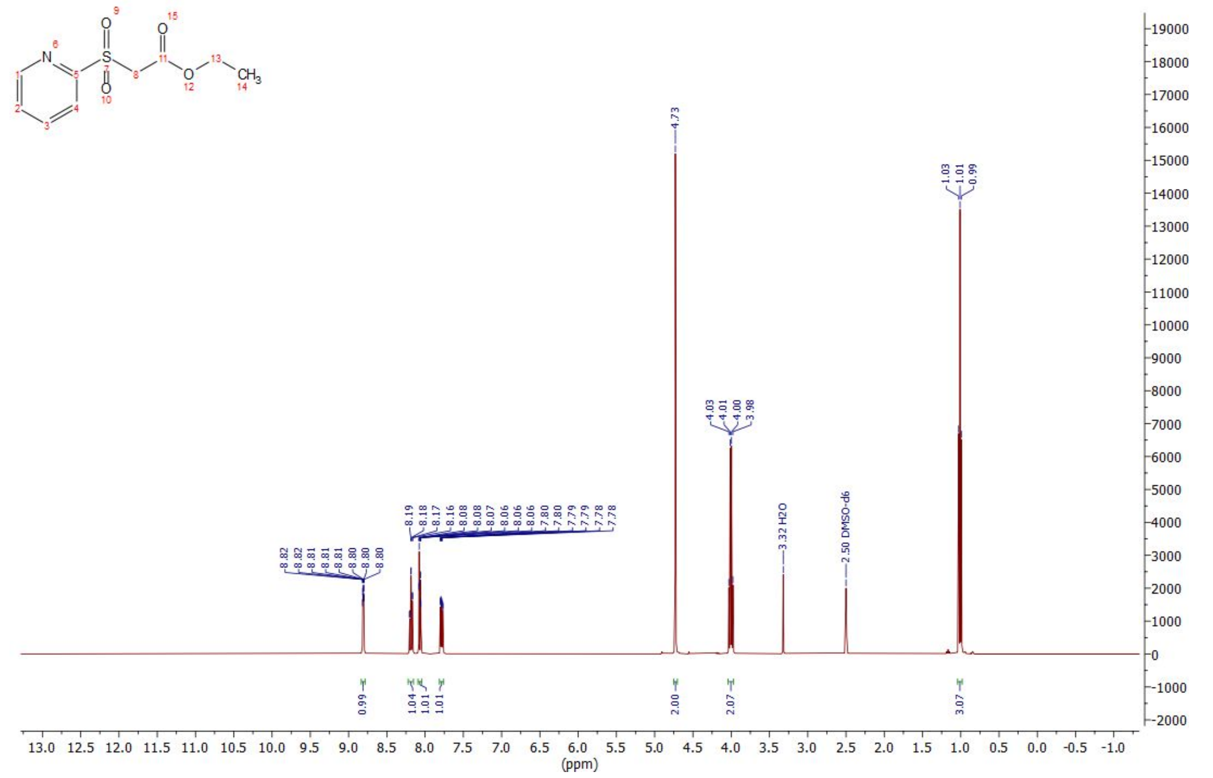

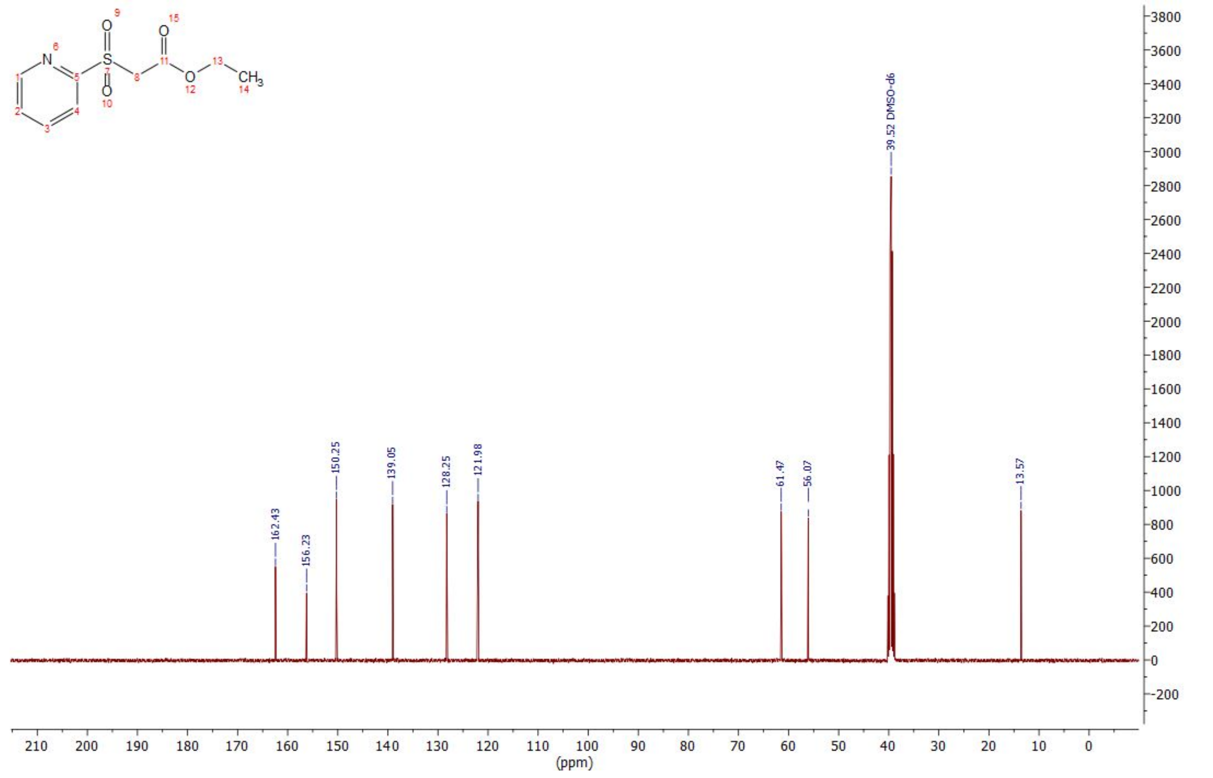

Figure S 16. *^{1}H (300 MHz) and $^{13}C\{^{1}H\}$ (75 MHz) NMR-Spectra in DMSO-d_6 of **1n**.*

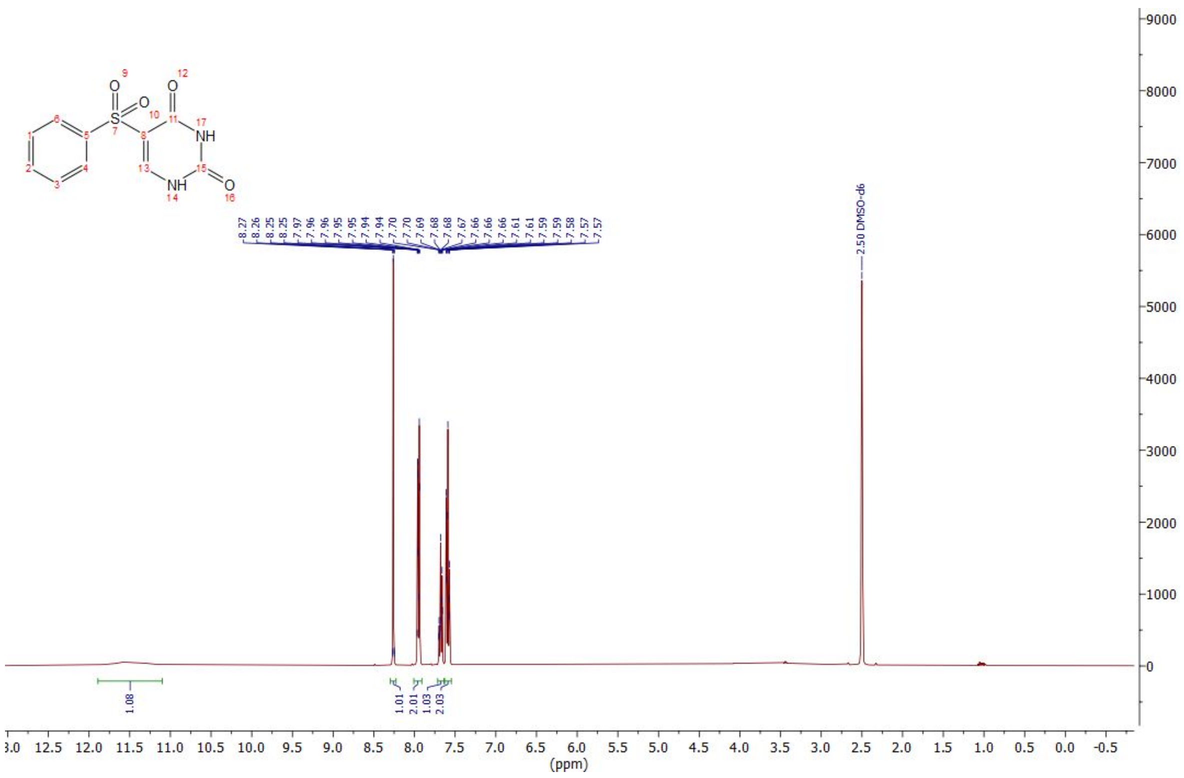

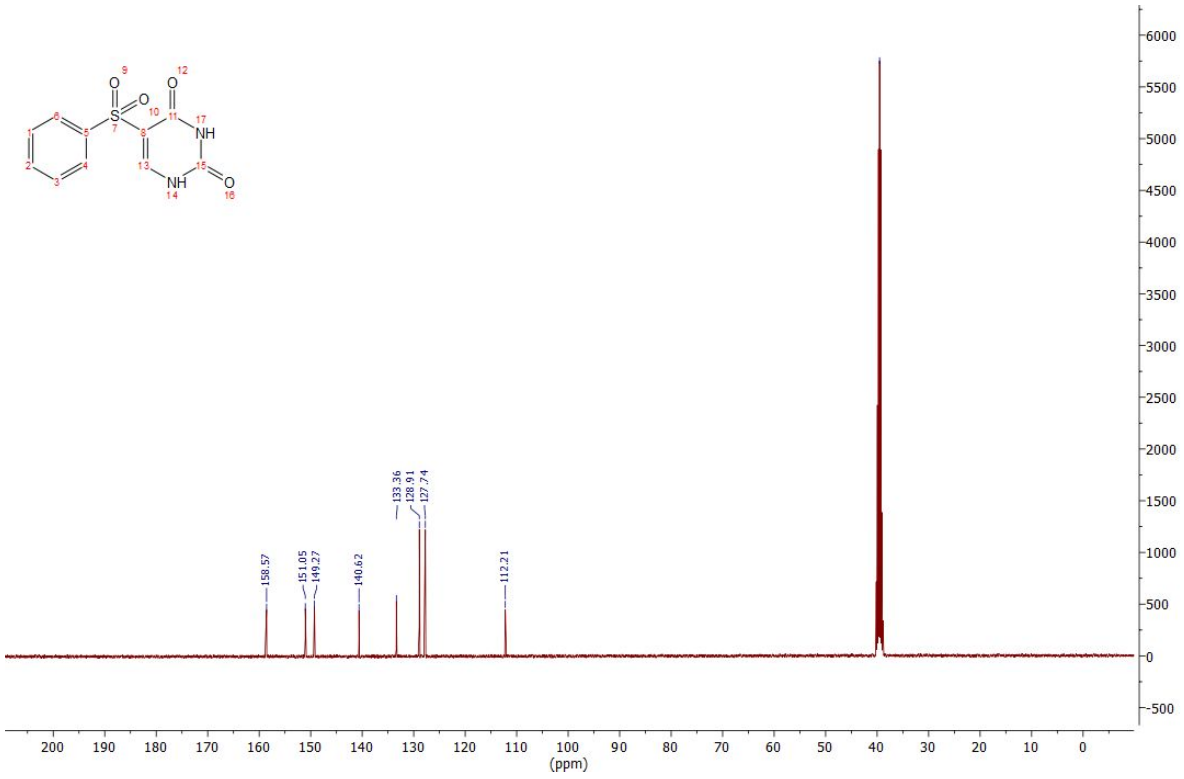

Figure S 17. *^{1}H (400 MHz)and $^{13}C\{^{1}H\}$ (101 MHz) NMR-Spectra in DMSO-d_6 of* ***5a****.*

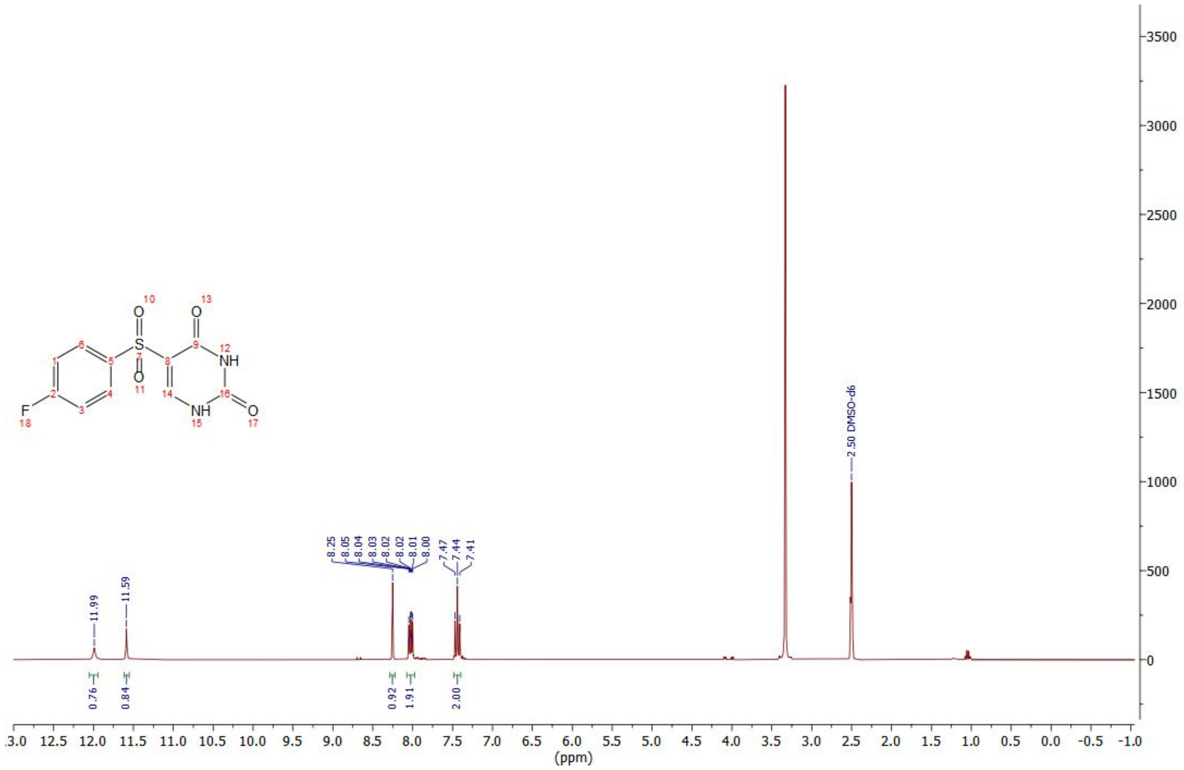

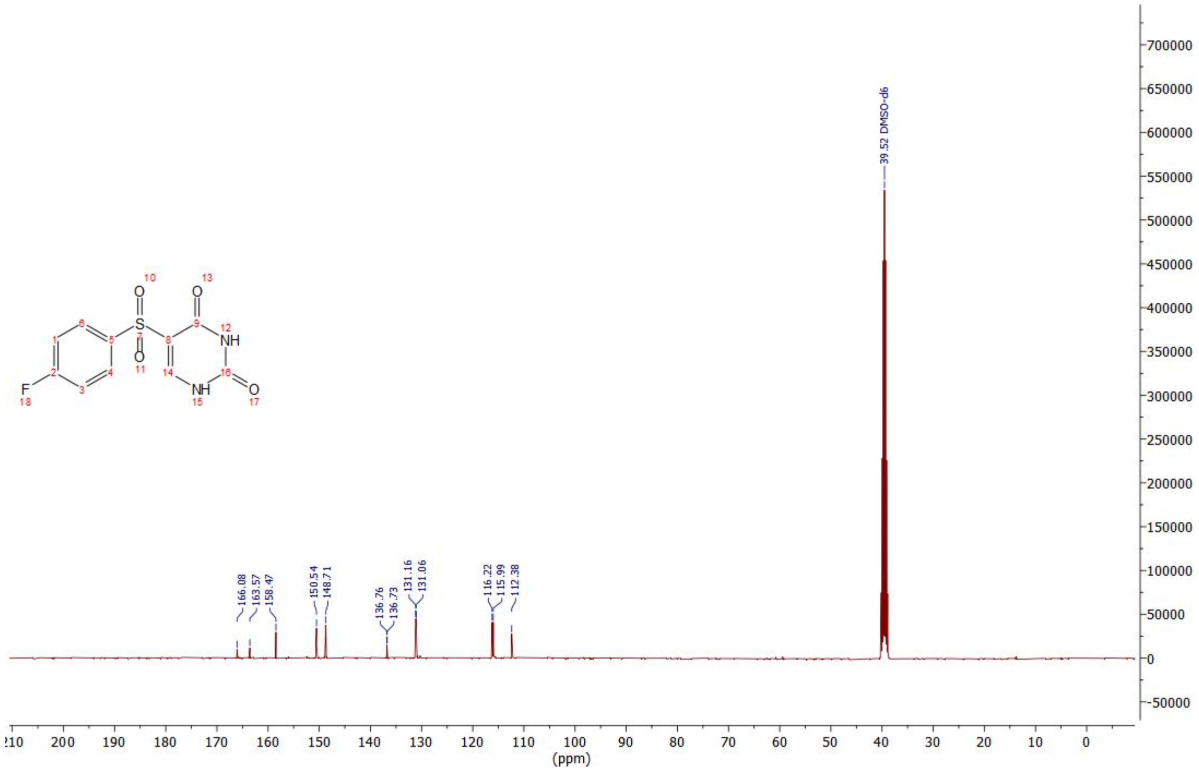

Figure S 18. *^{1}H (400 MHz) and ^{13}C{1H} (101 MHz) NMR-Spectra in DMSO-d_6 of **5b**.*

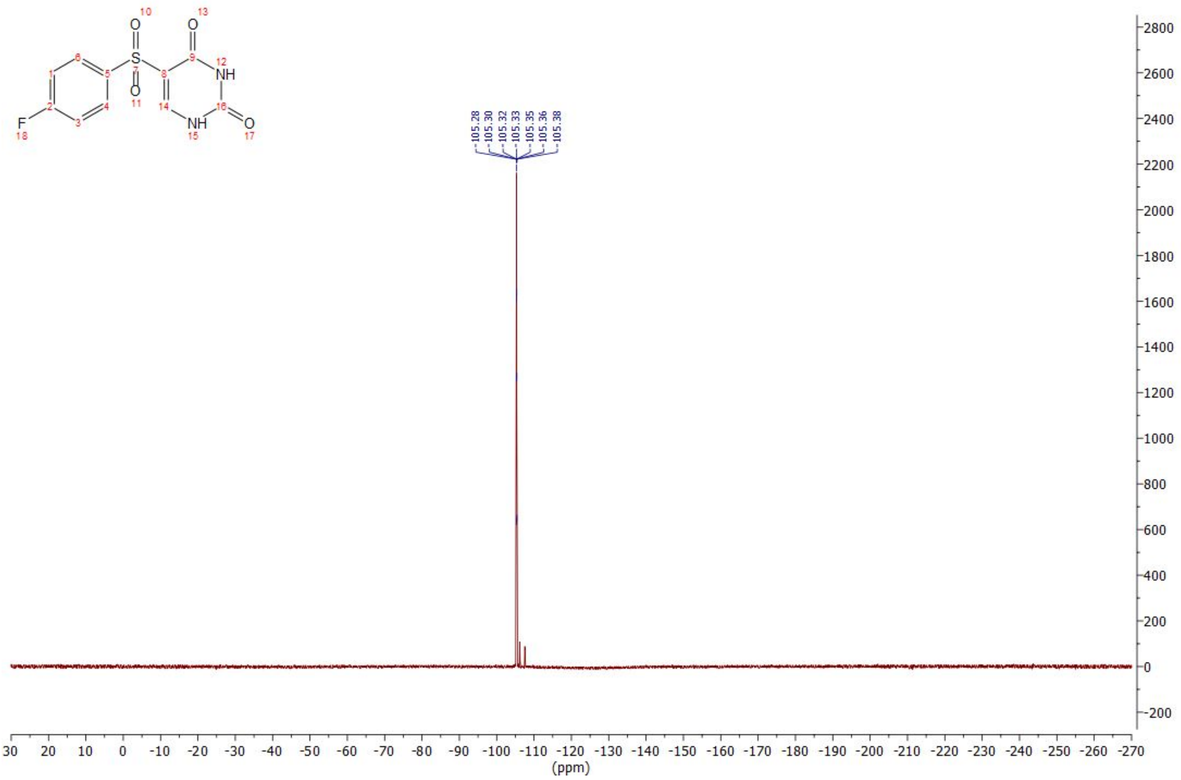

Figure S 19. *^{19}F (282 MHz) NMR-Spectra in DMSO-d_6 of **5b**.*

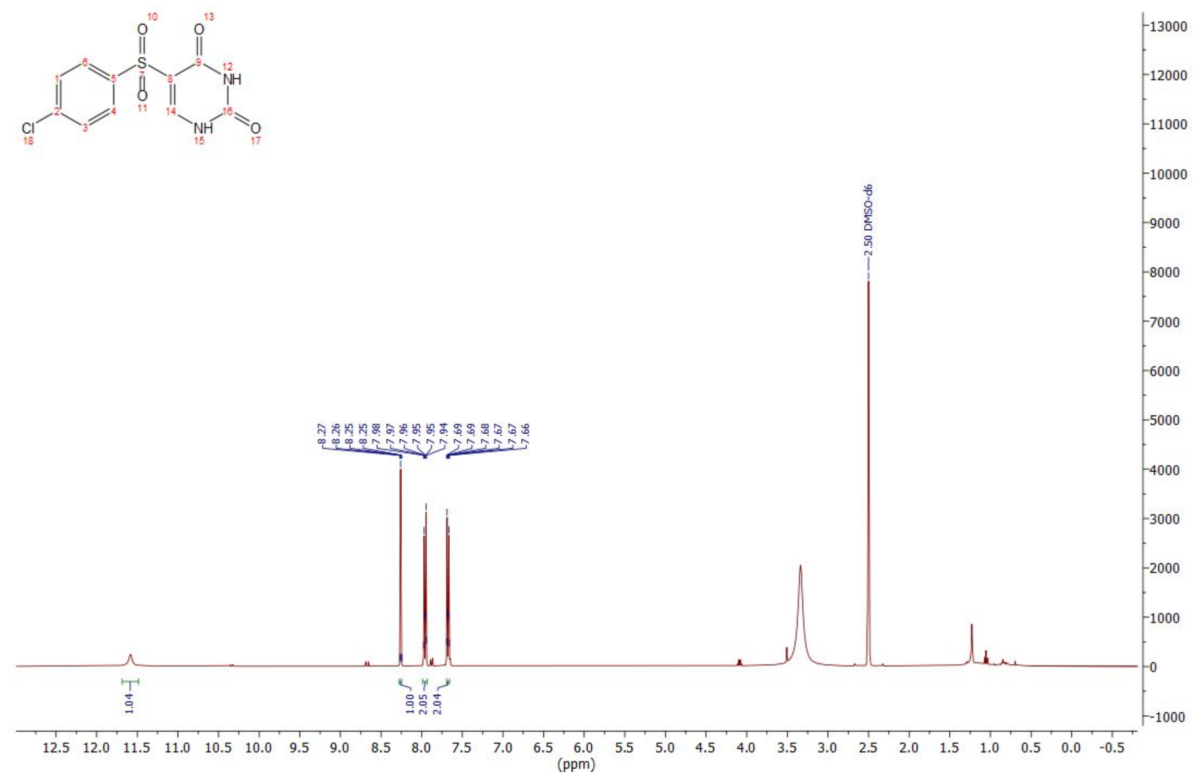

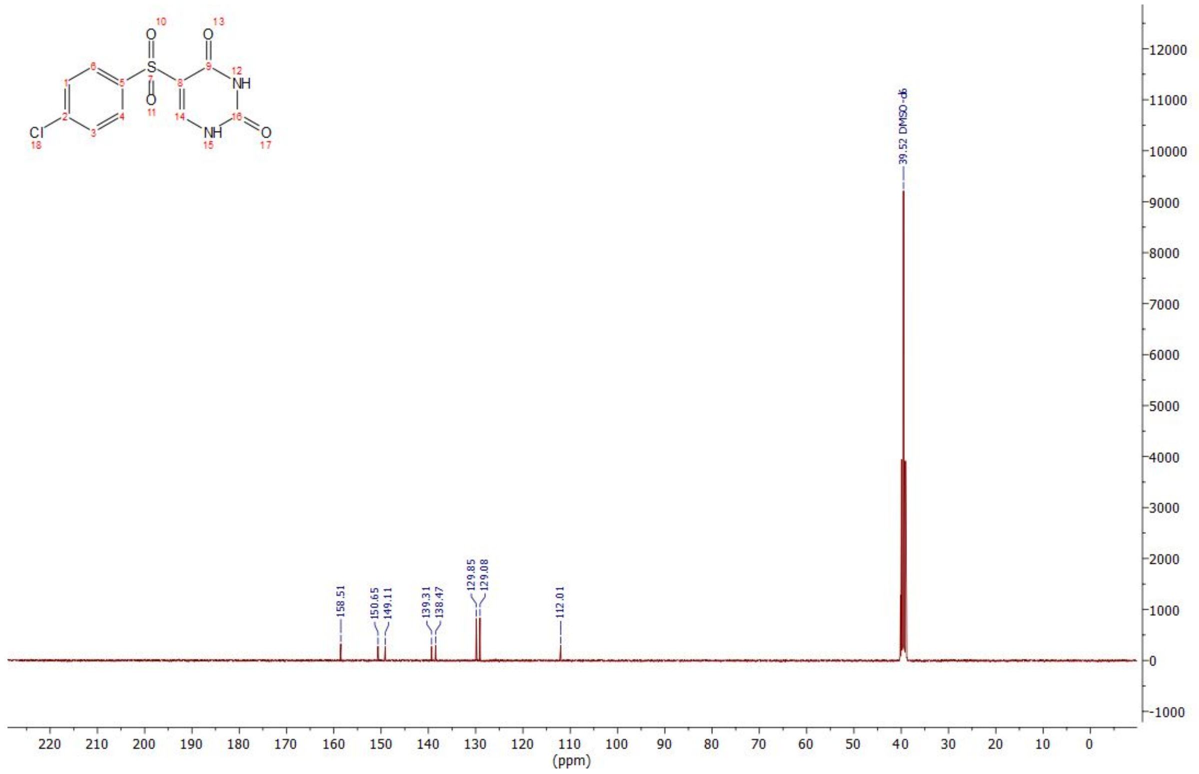

Figure S 20. *^{1}H (400 MHz) and ^{13}C{1H} (101 MHz) NMR-Spectra in DMSO-d$_6$ of **5c**.*

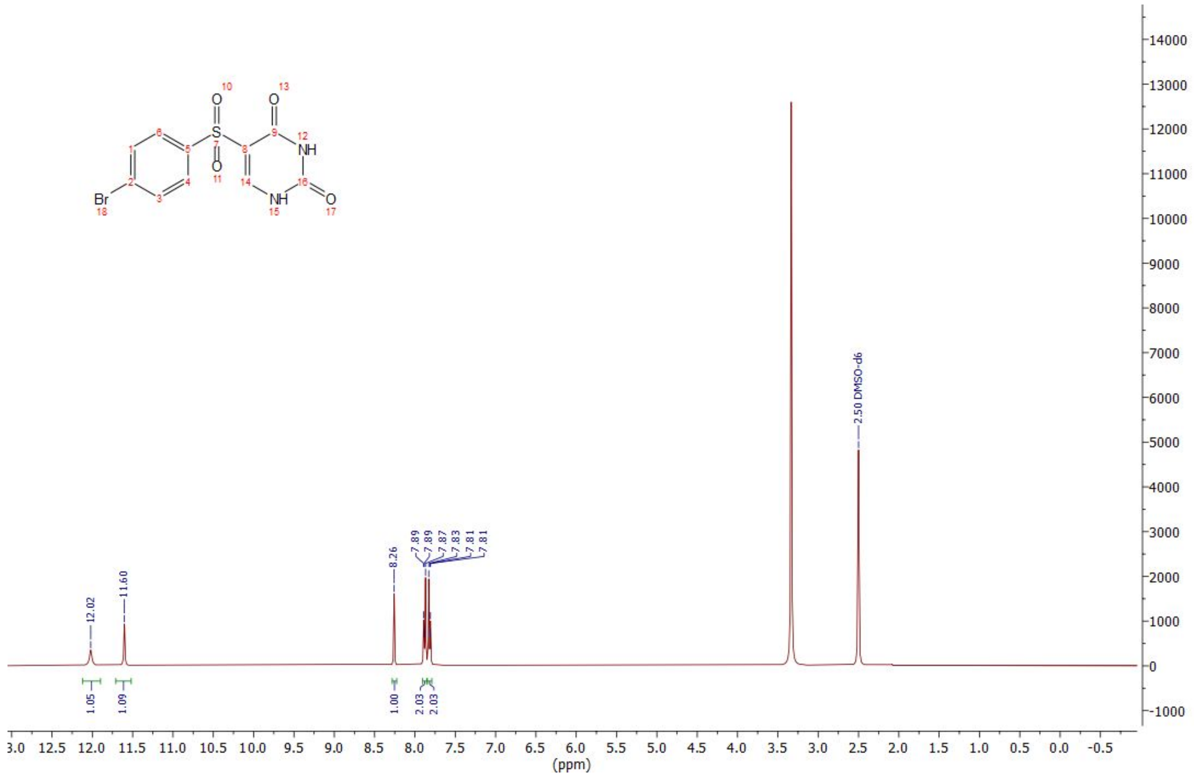

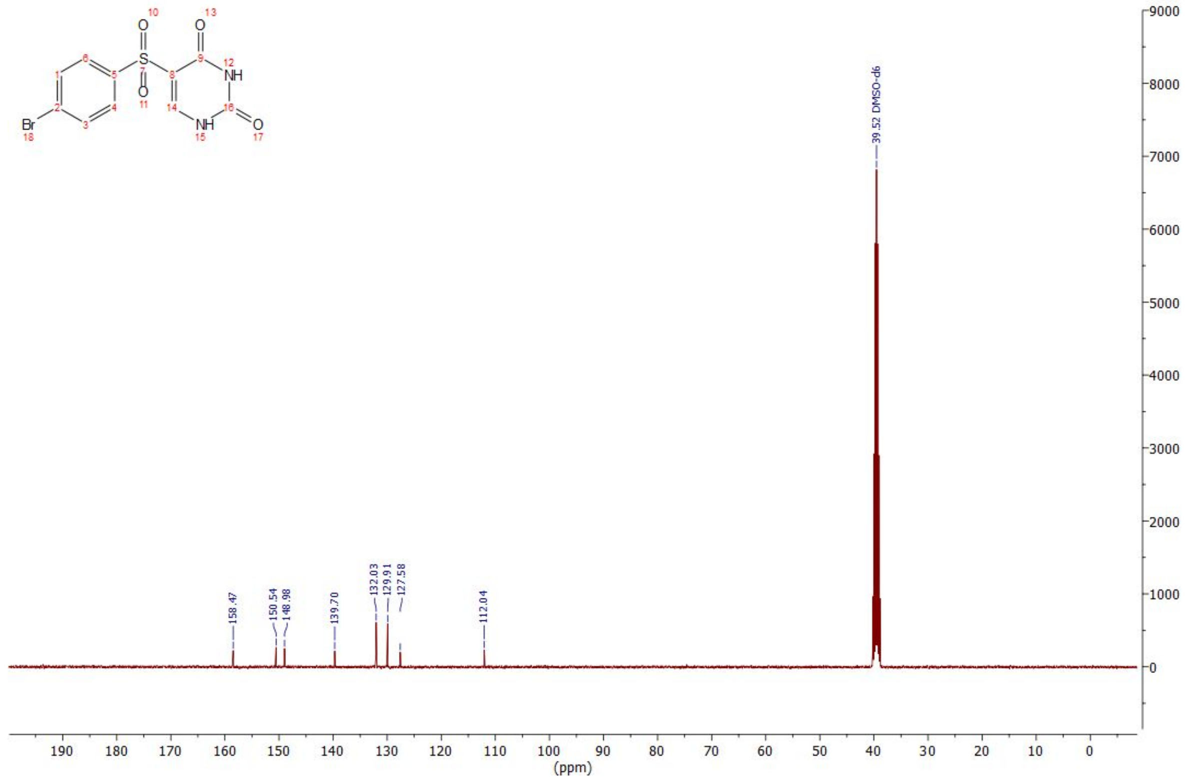

Figure S 21. *^{1}H (400 MHz)and ^{13}C{1H} (101 MHz) NMR-Spectra in DMSO-d$_6$ of* ***5d***.

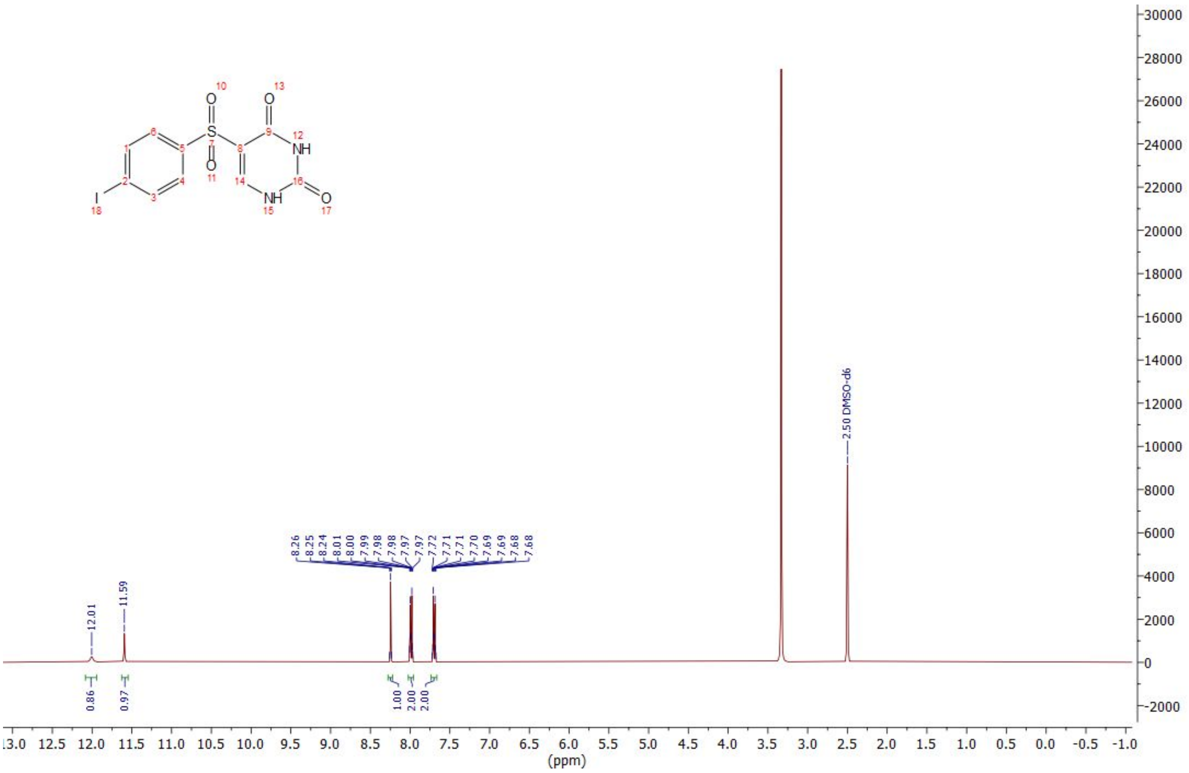

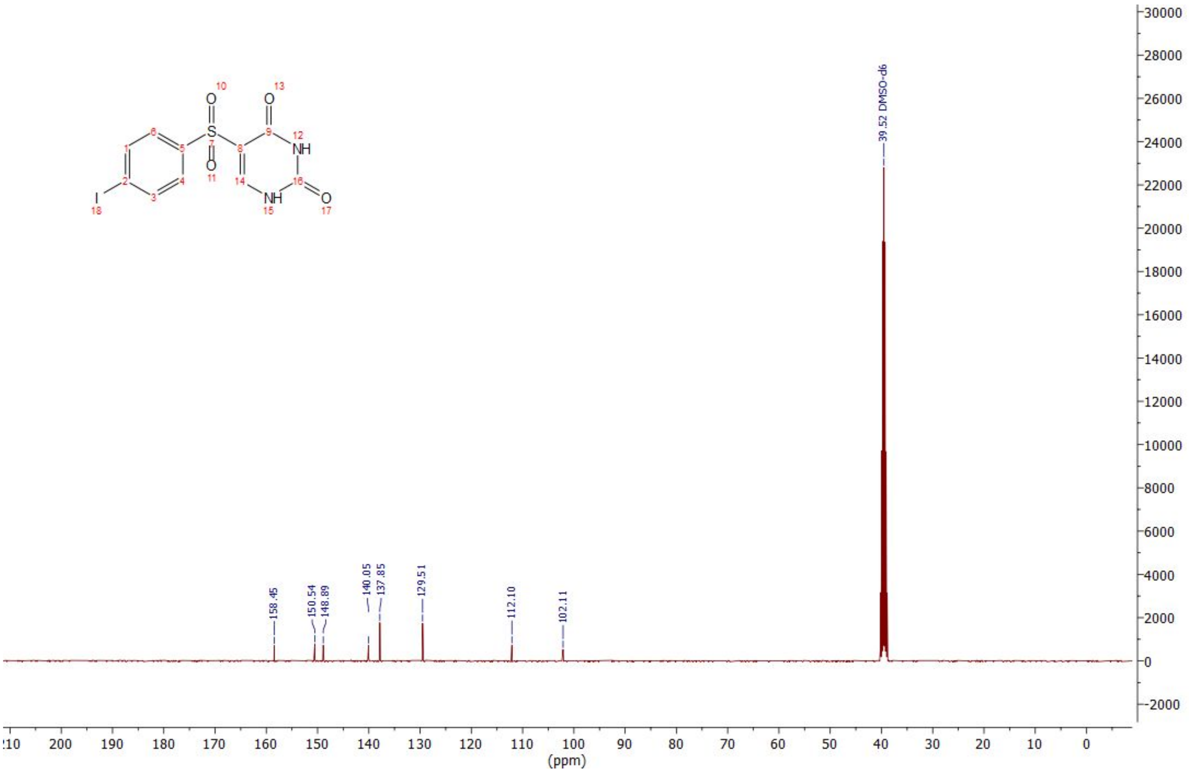

Figure S 22. *^{1}H (400 MHz) and $^{13}C\{1H\}$ (101 MHz) NMR-Spectra in DMSO-d_6 of* ***5e****.*

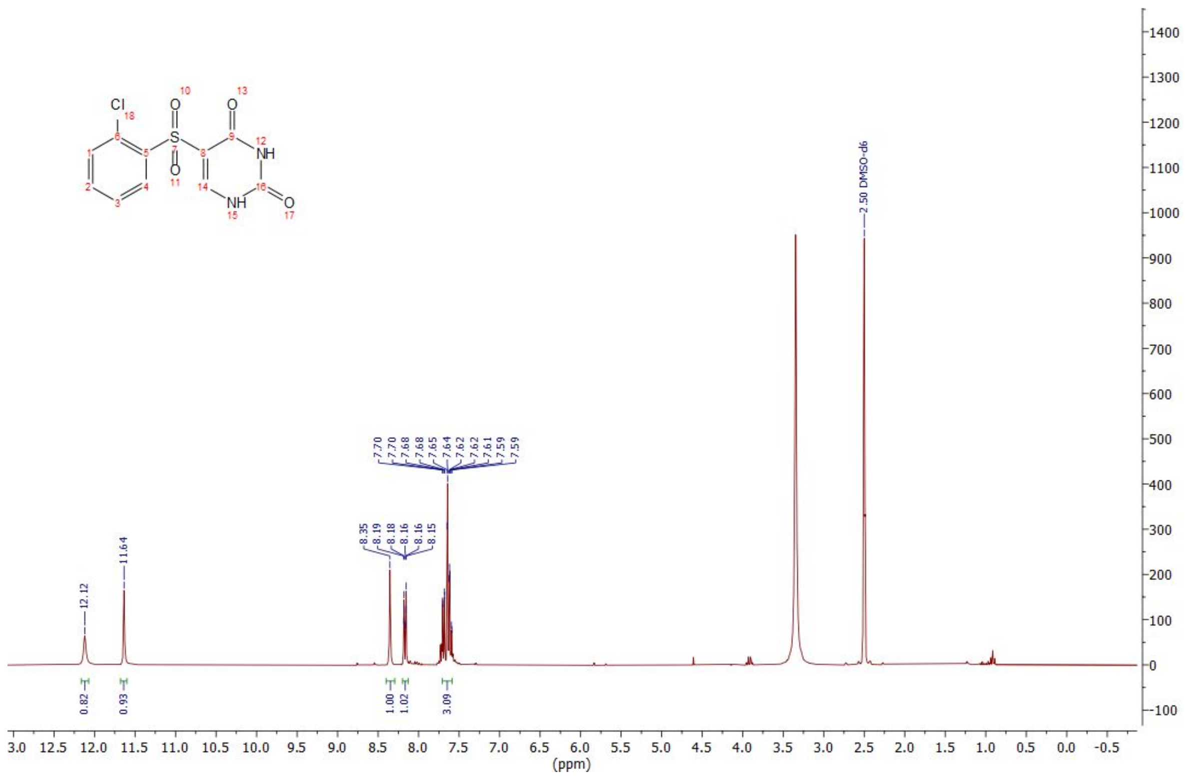

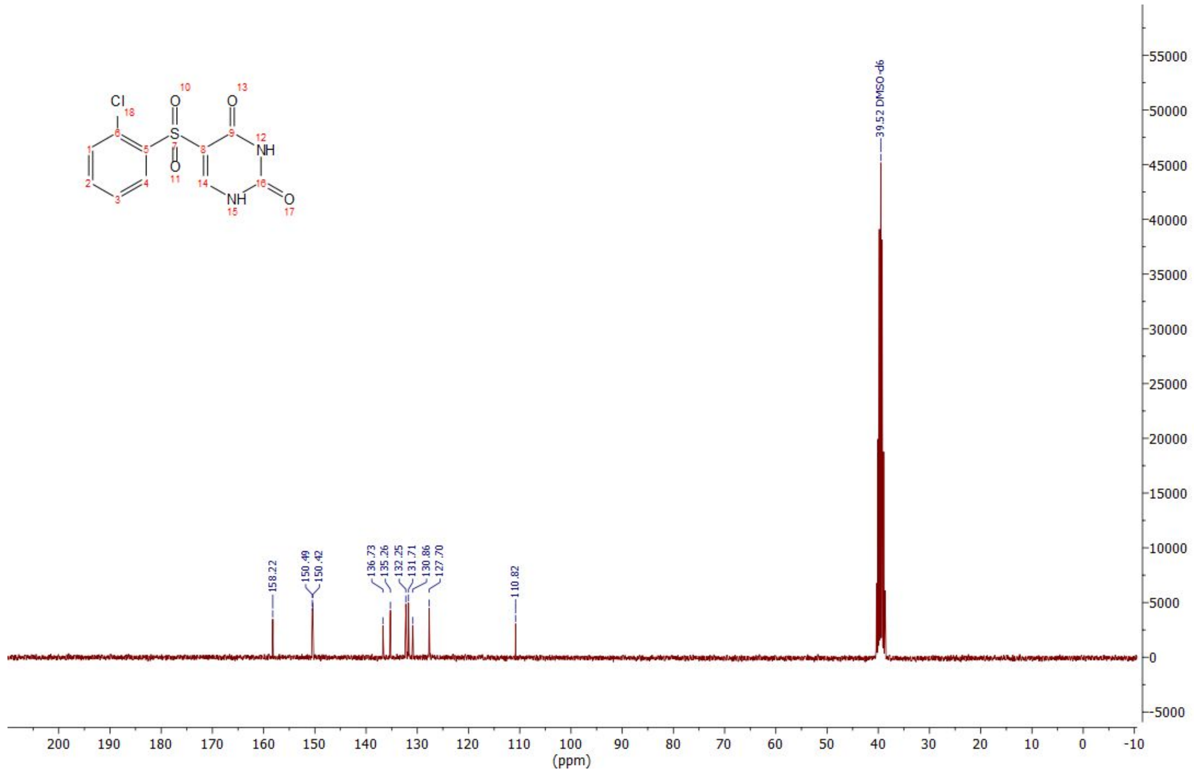

Figure S 23. *^{1}H (300 MHz) and ^{13}C{1H} (75 MHz) NMR-Spectra in DMSO-d_6 of **5f**.*

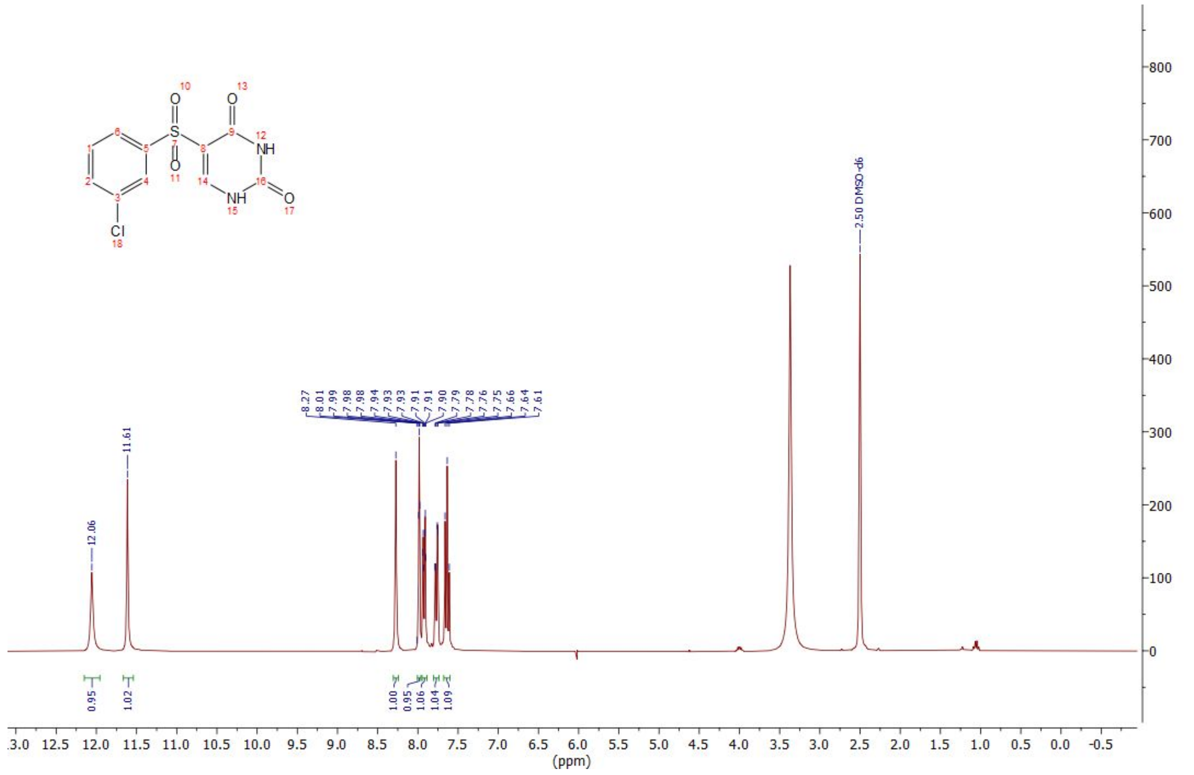

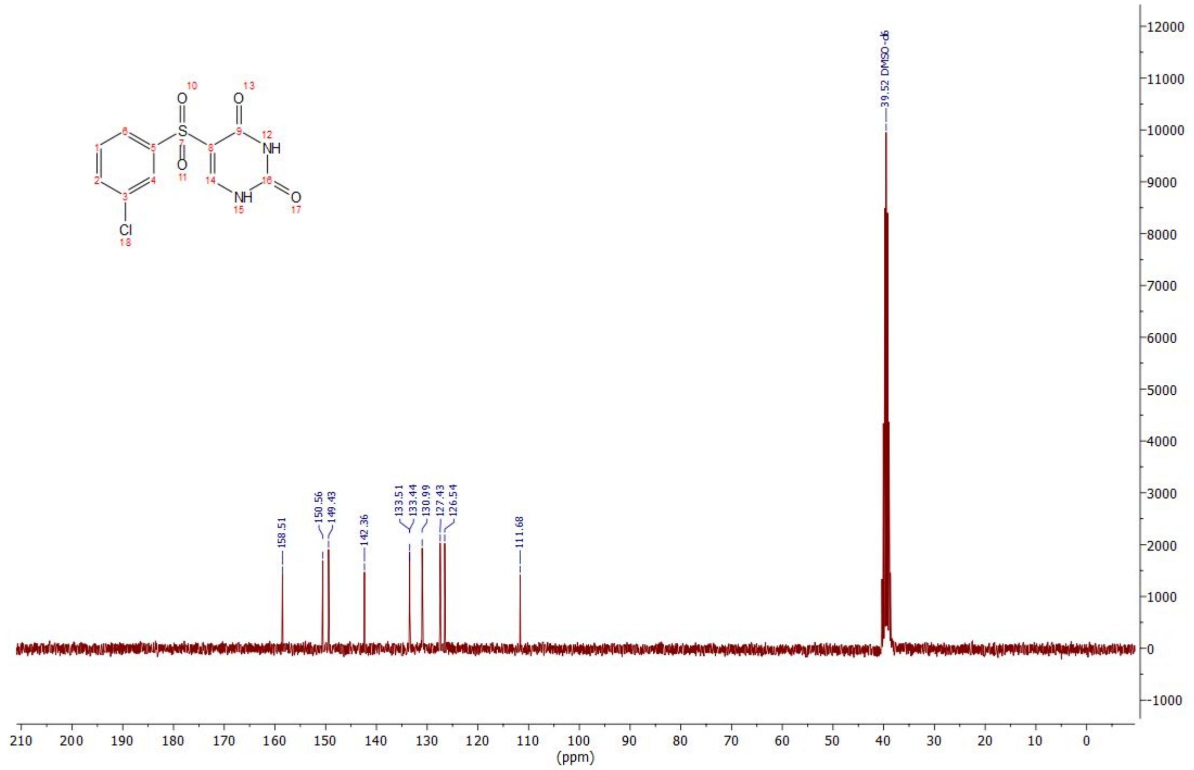

Figure S 24. *^{1}H (300 MHz) and $^{13}C\{1H\}$ (75 MHz) NMR-Spectra in DMSO-d_6 of* ***5g****.*

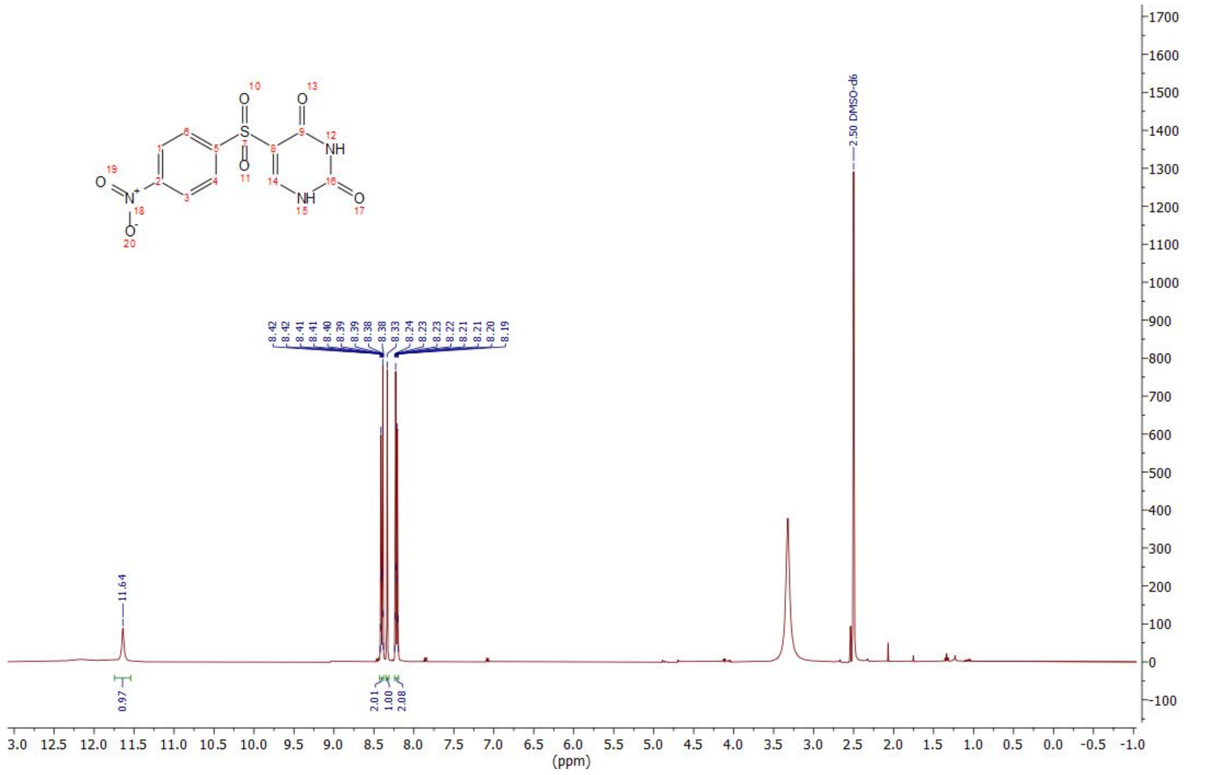

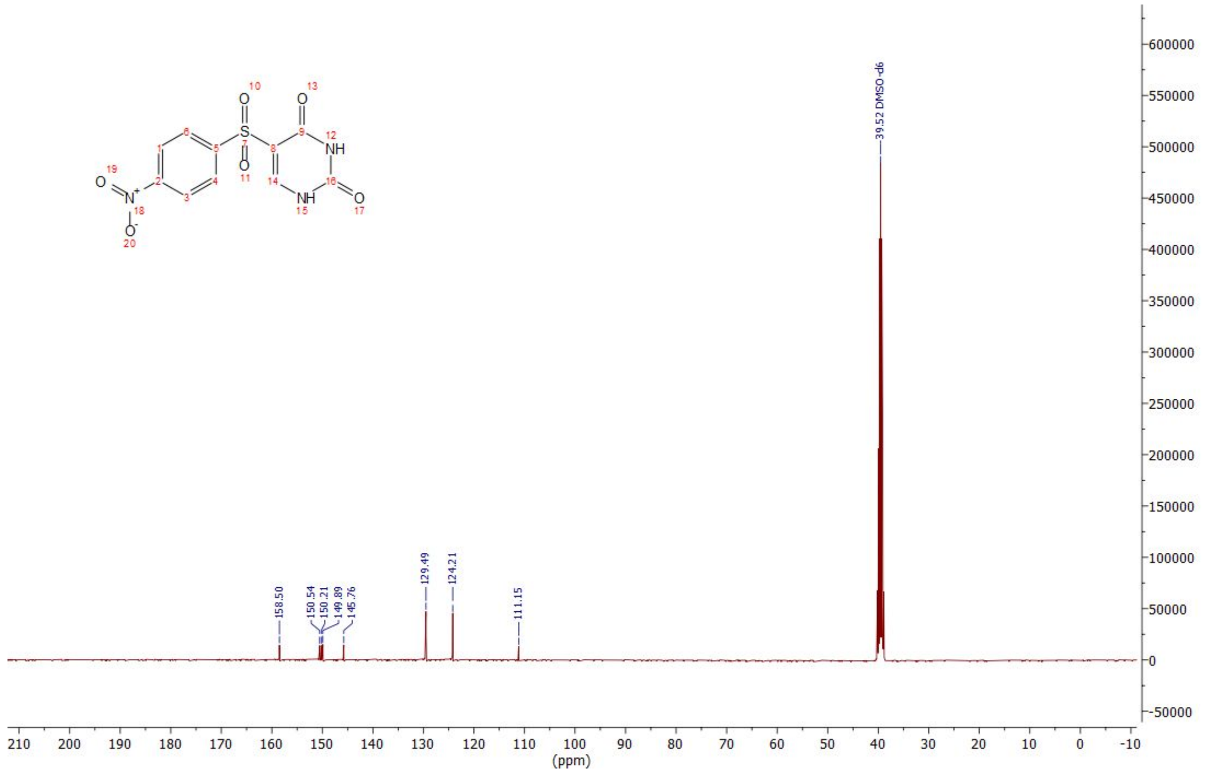

Figure S 25. *^{1}H (400 MHz) and $^{13}C\{1H\}$ (101 MHz) NMR-Spectra in DMSO-d_6 of* ***5h****.*

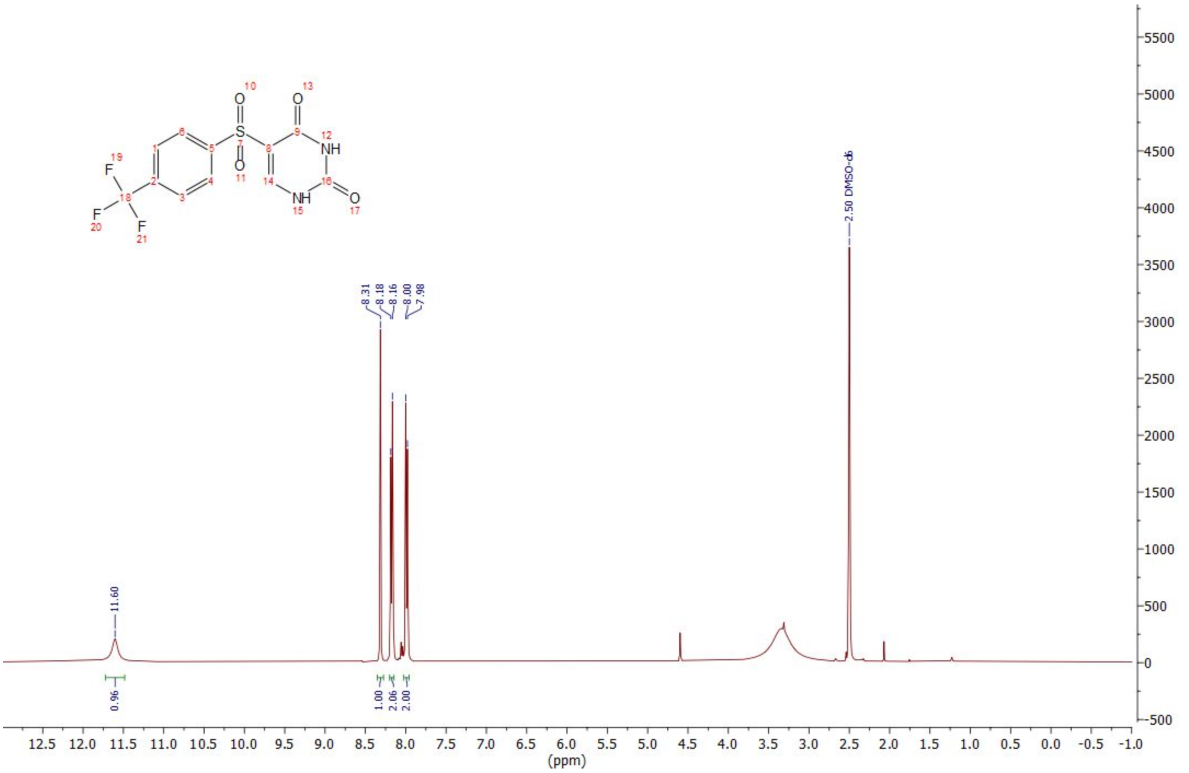

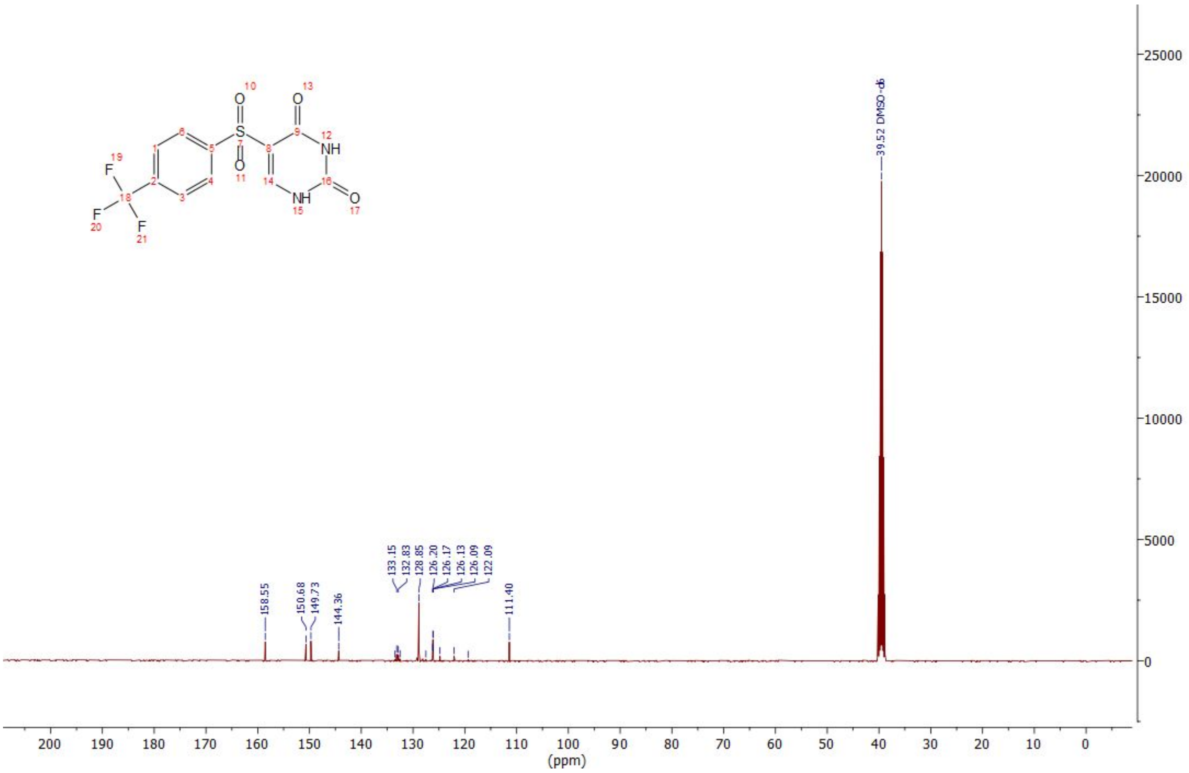

Figure S 26. *^{1}H (400 MHz) and $^{13}C\{1H\}$ (101 MHz) NMR-Spectra in DMSO-d_6 of **5i**.*

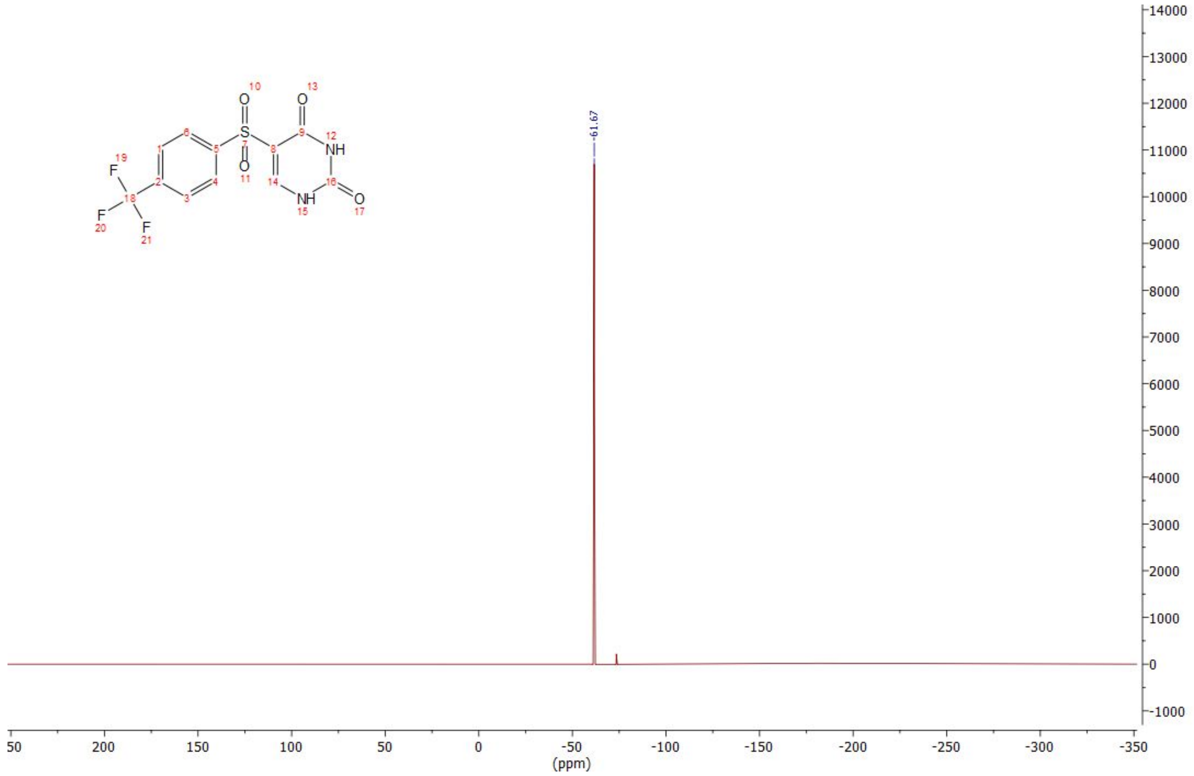

Figure S 27. *^{19}F (376 MHz) NMR-Spectra in DMSO-d_6 of* ***5i****.*

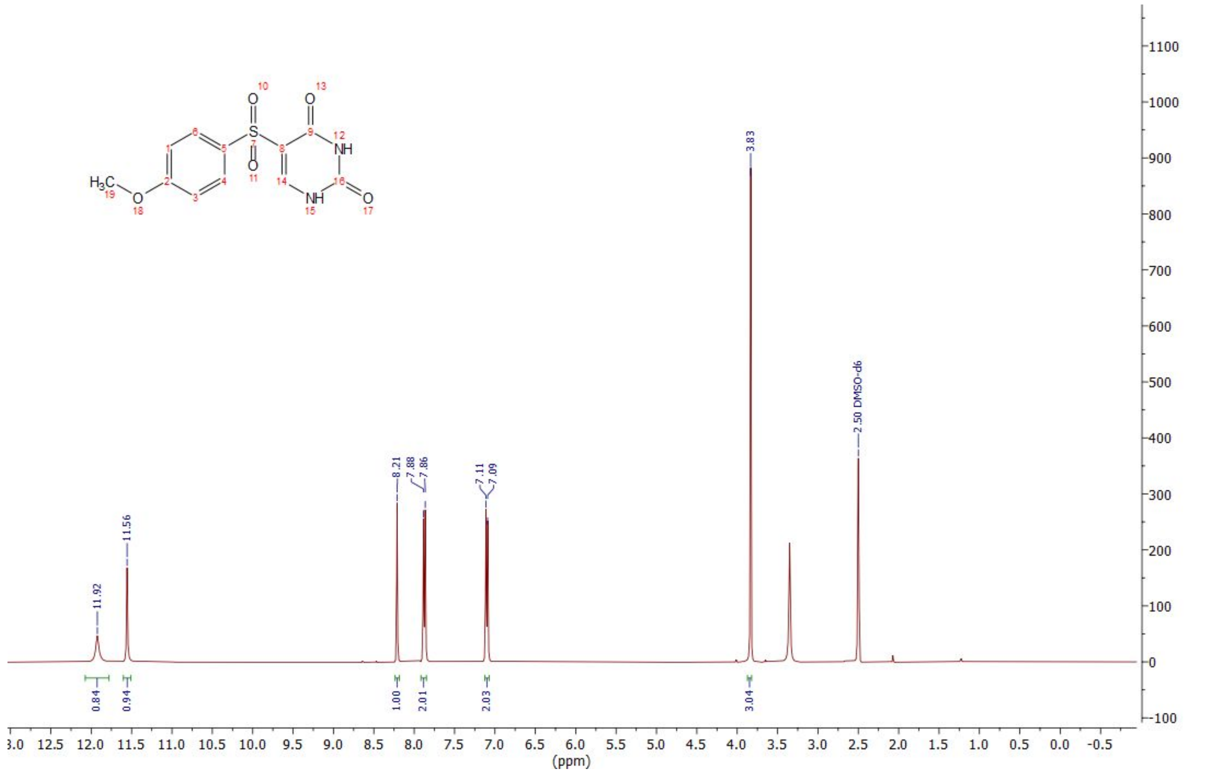

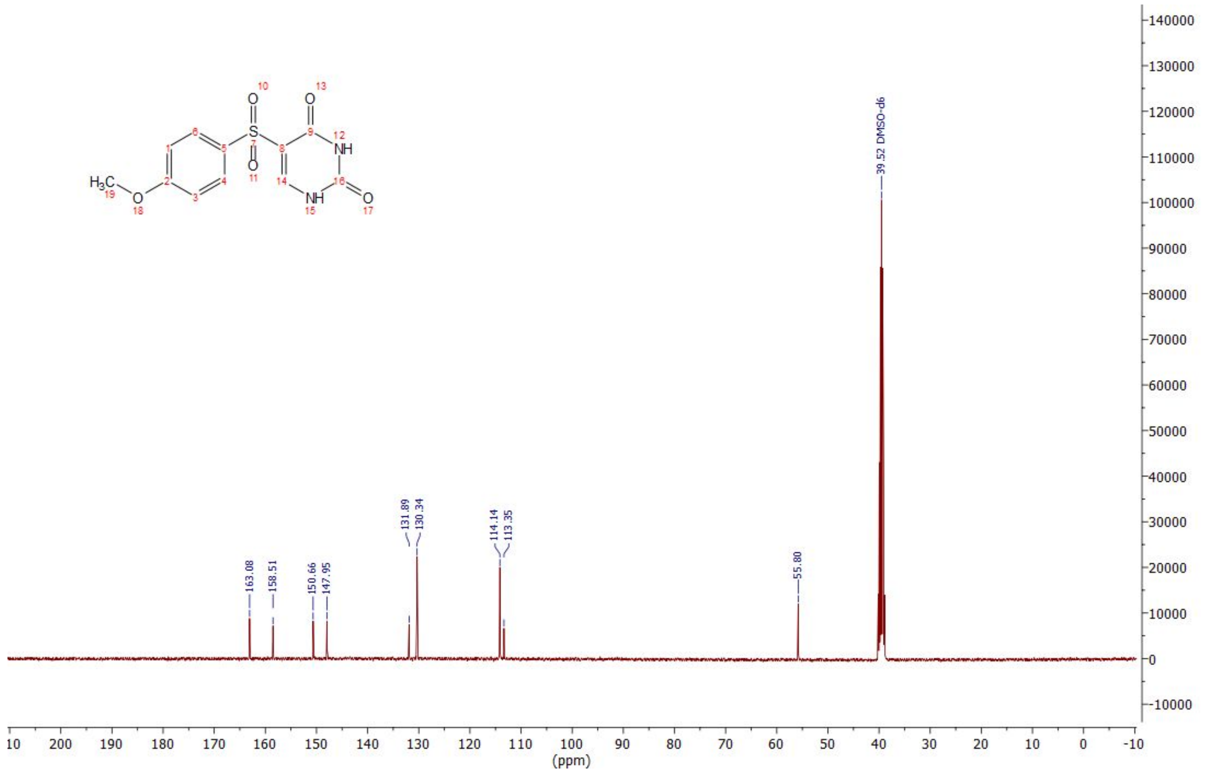

Figure S 28. *^{1}H (400 MHz) and $^{13}C\{1H\}$ (101 MHz) NMR-Spectra in DMSO-d_6 of* ***5j****.*

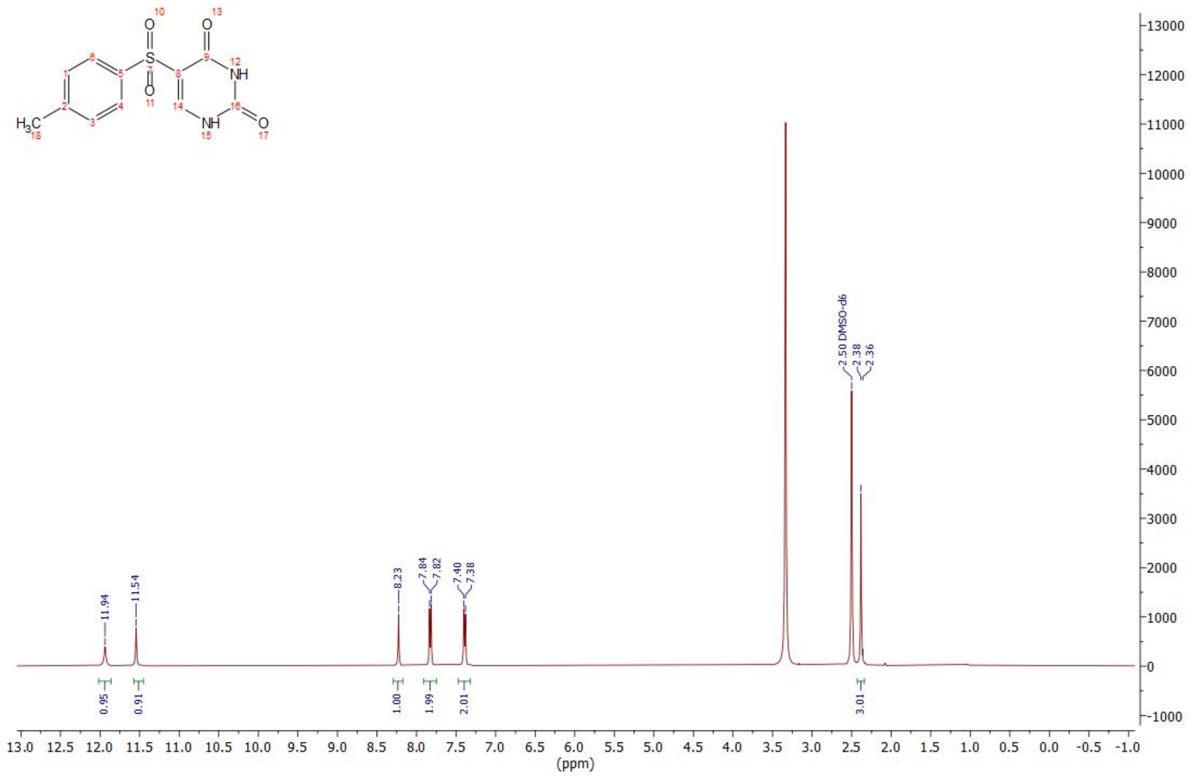

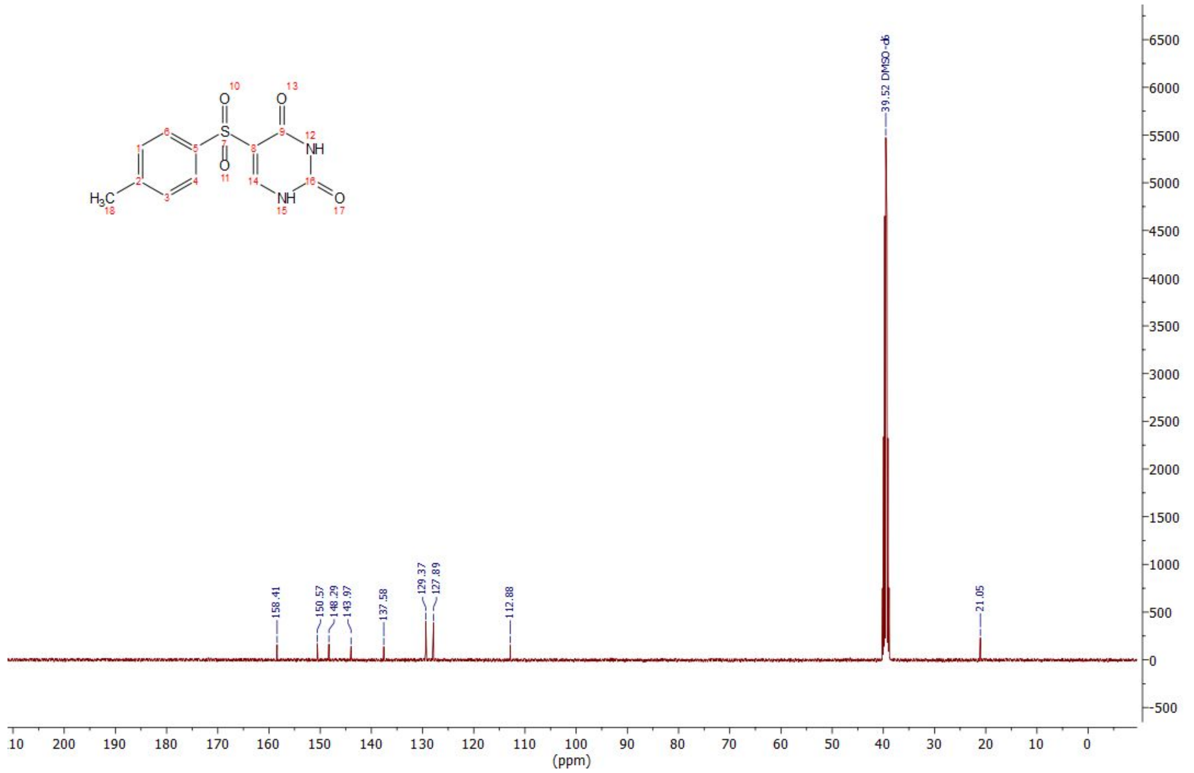

Figure S 29. *^{1}H (400 MHz) and $^{13}C\{1H\}$ (101 MHz) NMR-Spectra in DMSO-d_6 of **5k**.*

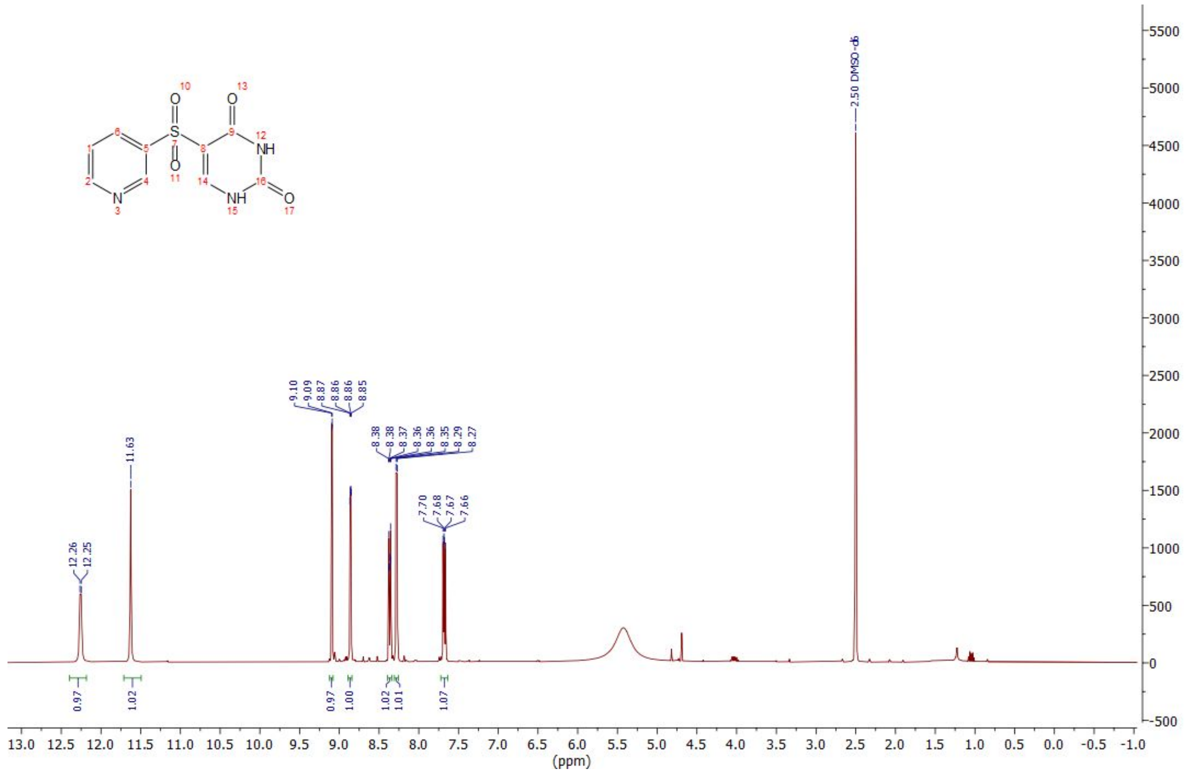

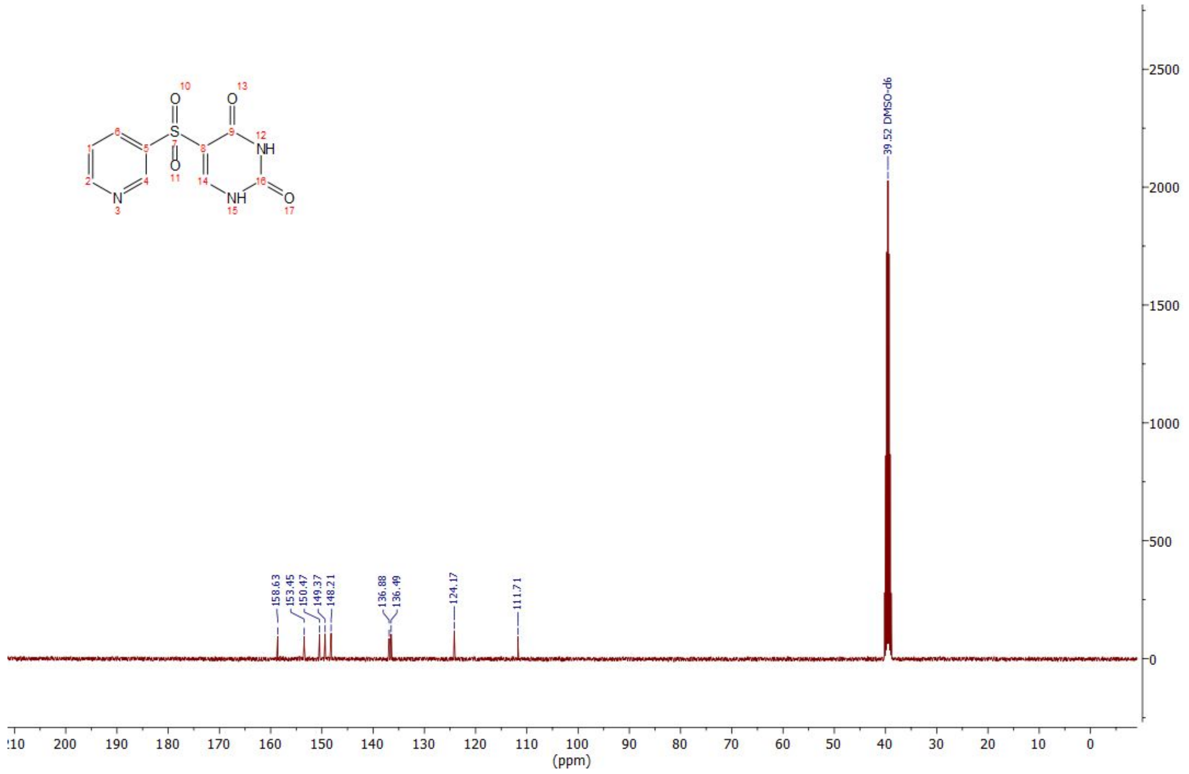

Figure S 30. *^{1}H (400 MHz) and ^{13}C{1H} (101 MHz) NMR-Spectra in DMSO-d_6 of* ***5l***.

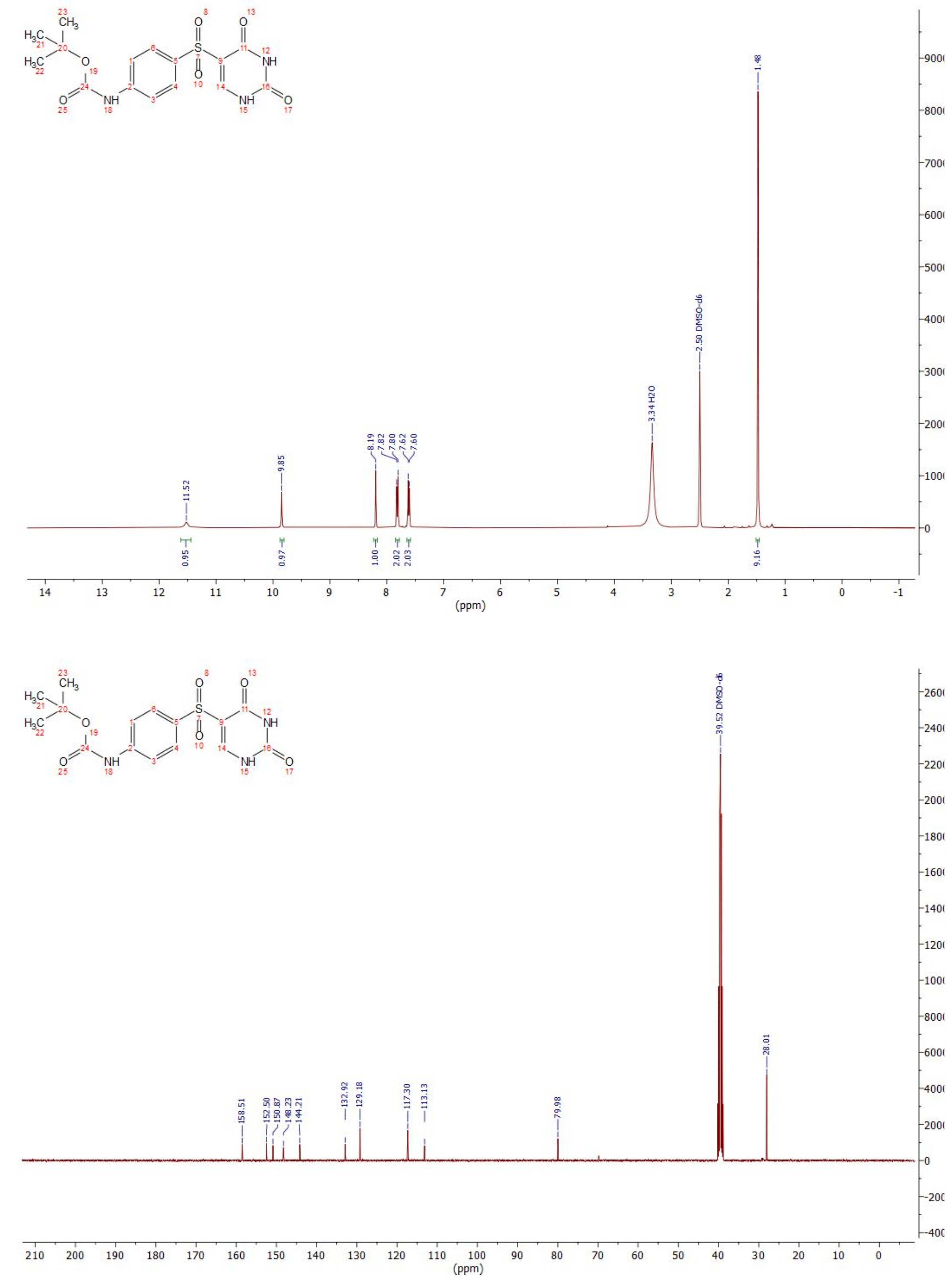

Figure S 31. *^{1}H (400 MHz) and $^{13}C\{^{1}H\}$ (101 MHz) NMR-Spectra in DMSO-d_6 of* ***5m****.*

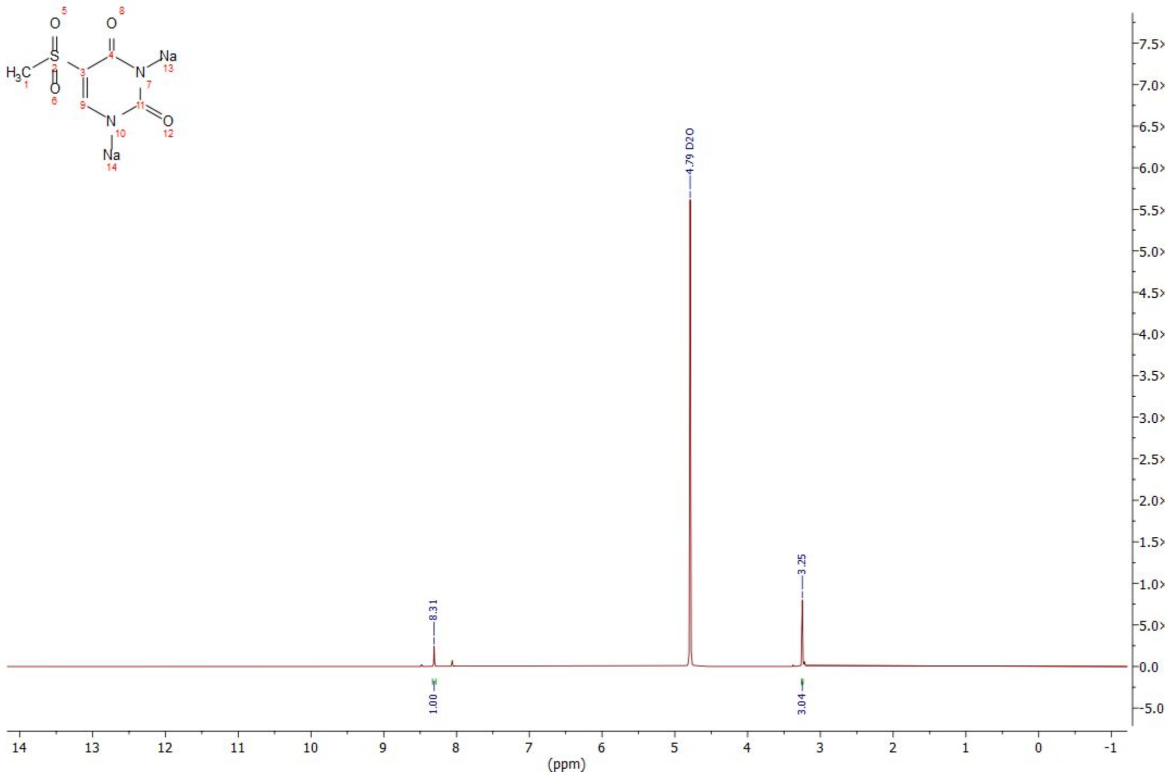

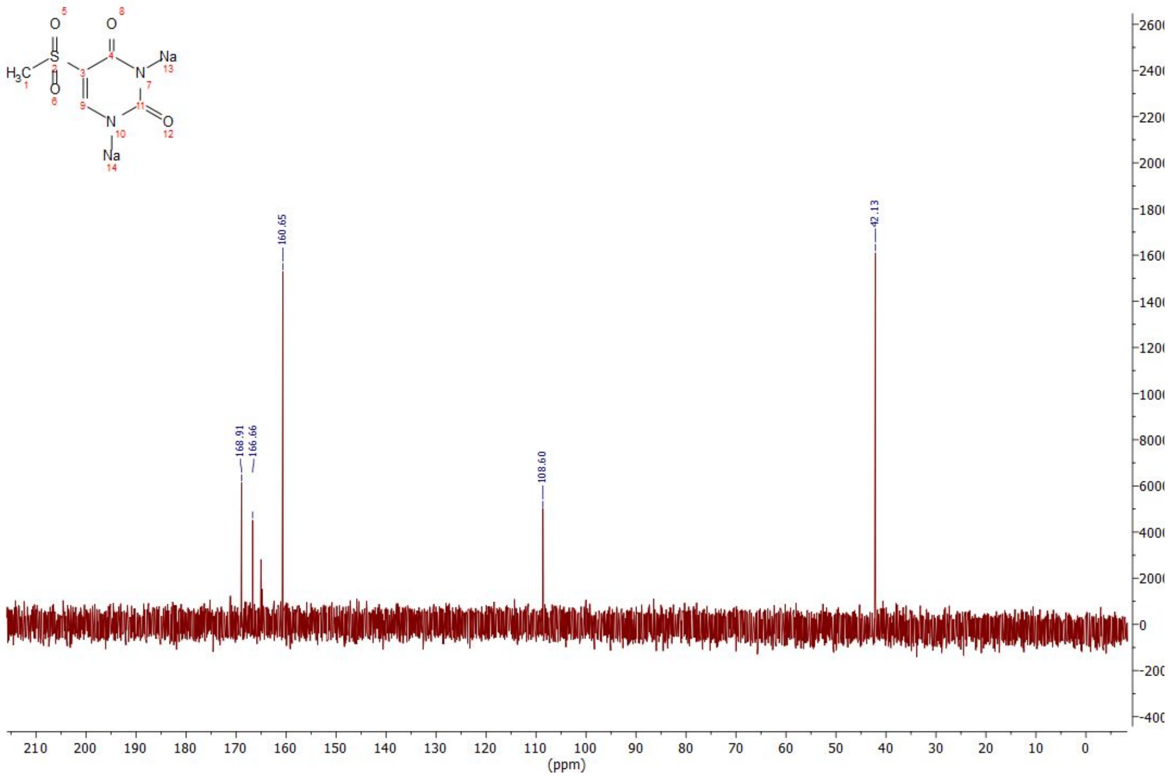

Figure S 32. *^{1}H (400 MHz) and $^{13}C\{^{1}H\}$ (101 MHz) NMR-Spectra in D_2O of* ***5r****.*

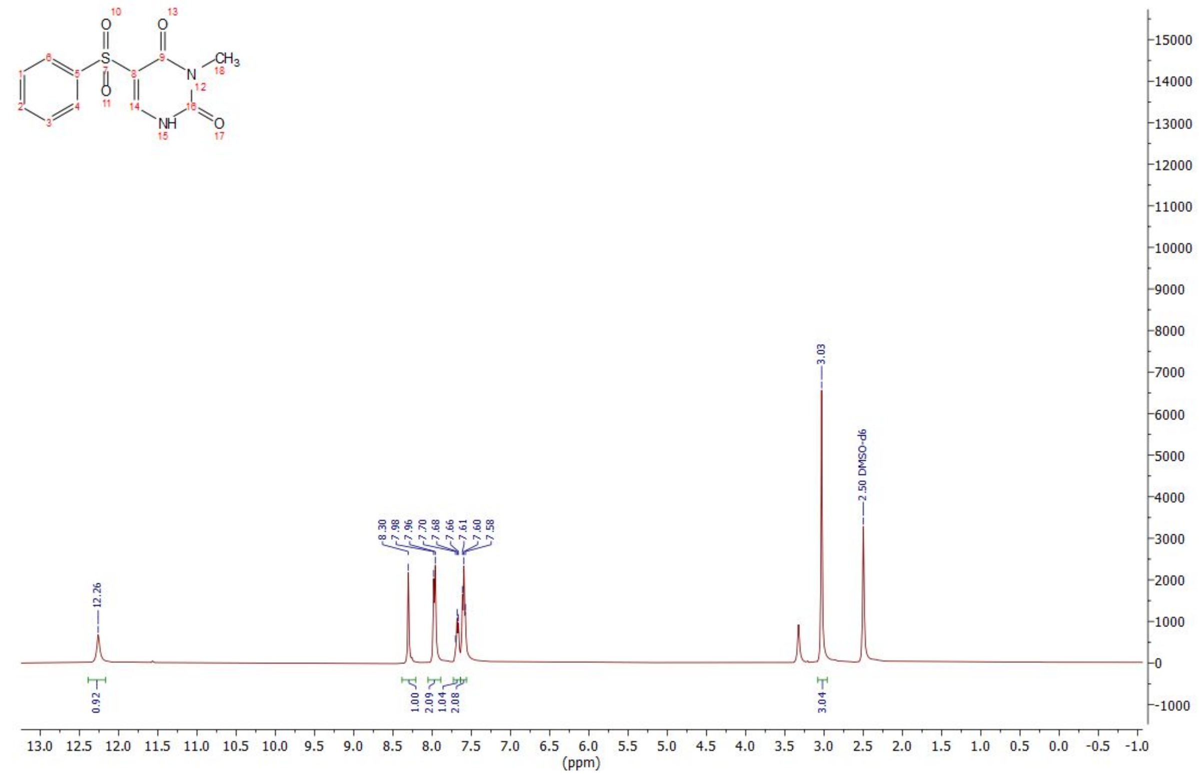

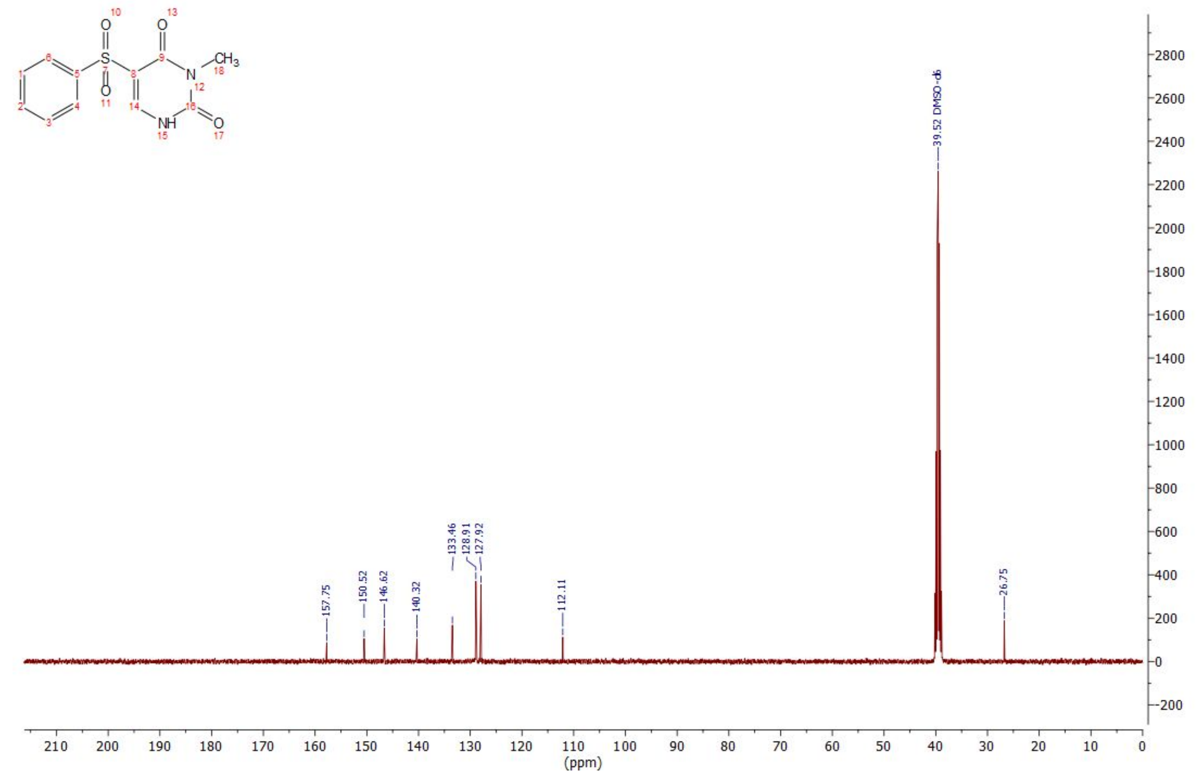

Figure S 33. *^{1}H (400 MHz)and $^{13}C\{1H\}$ (101MHz) NMR-Spectra in DMSO-d_6 of **6a**.*

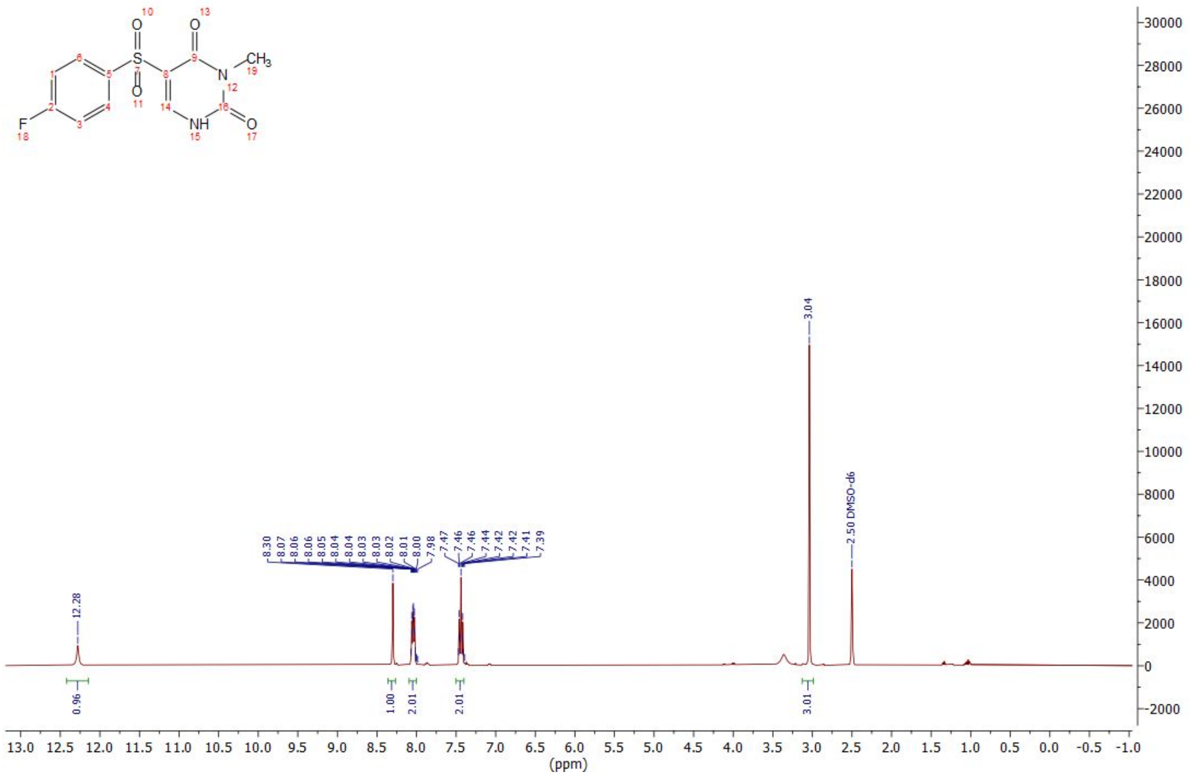

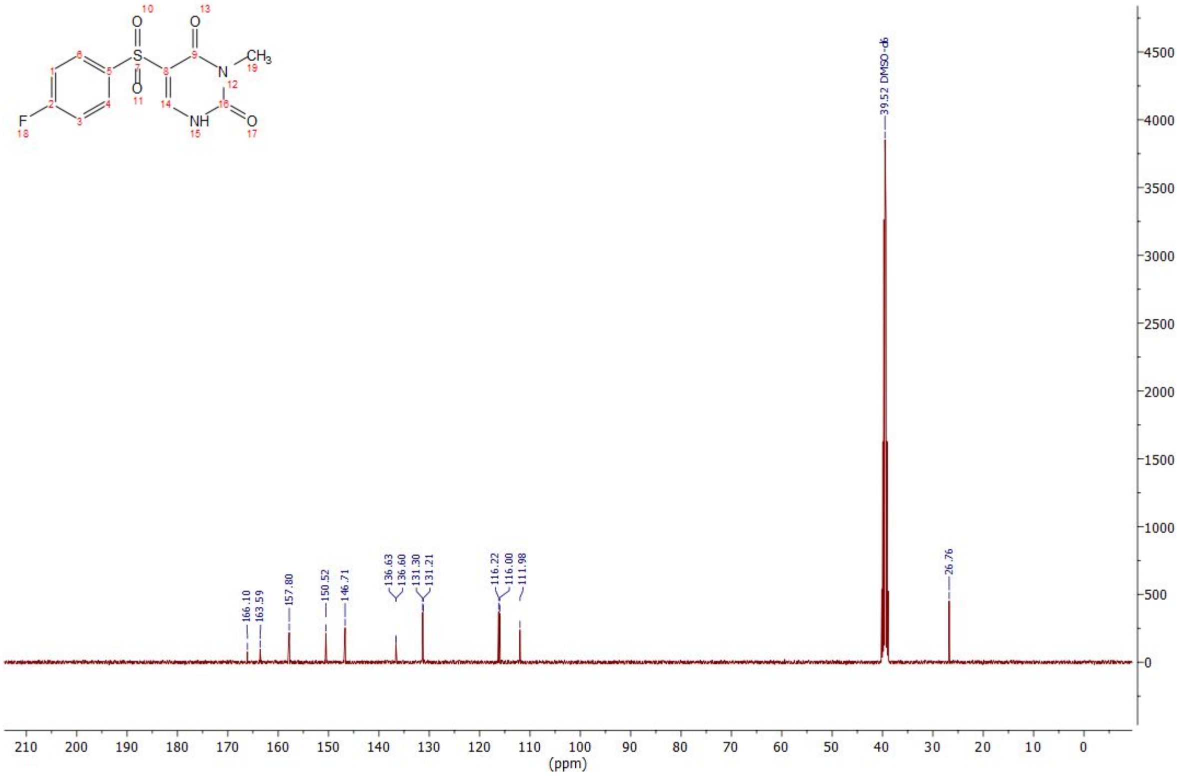

Figure S 34. *^{1}H (400 MHz) and $^{13}C\{1H\}$ (101 MHz) NMR-Spectra in DMSO-d_6 of **6b**.*

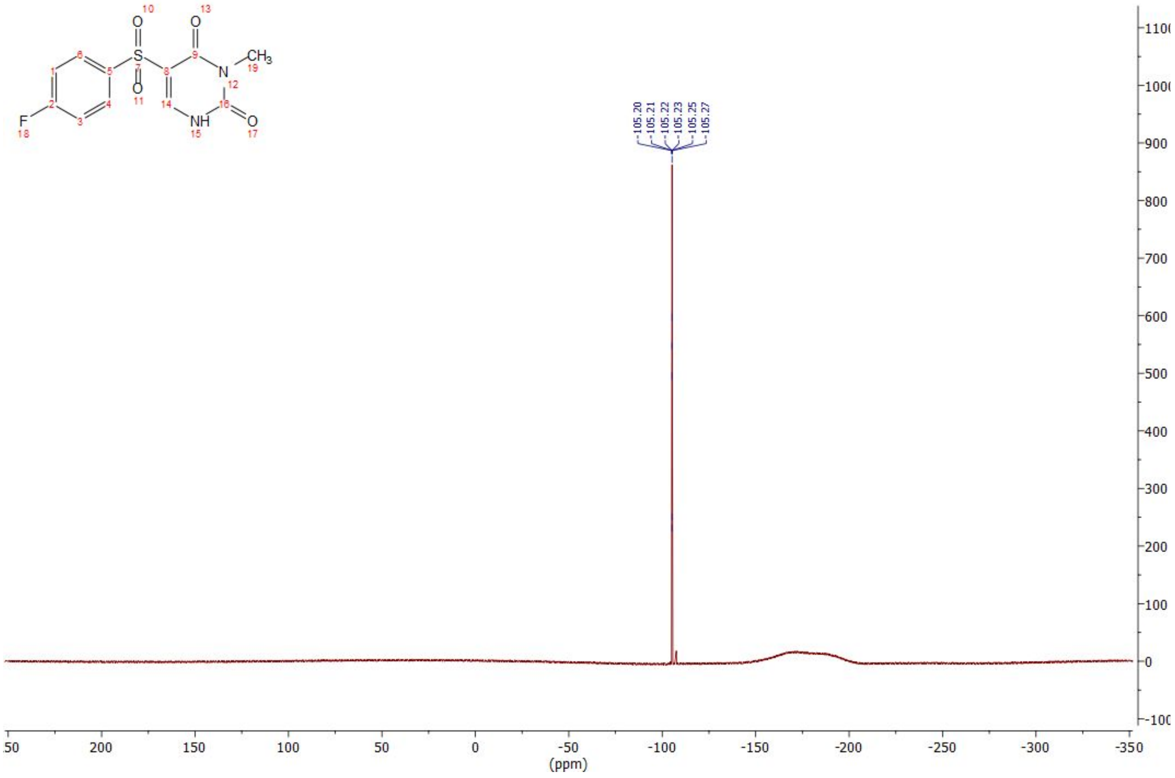

Figure S 35. *^{19}F (376 MHz) NMR-Spectra in DMSO-d_6 of **6b**.*

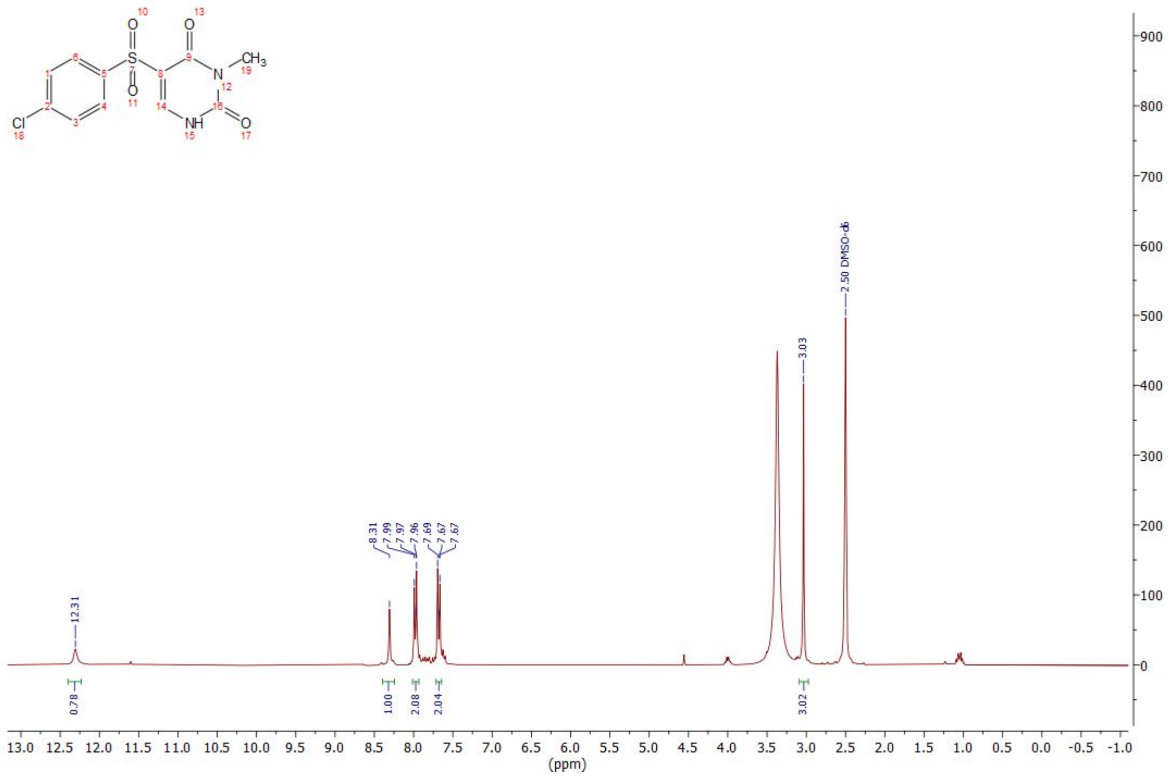

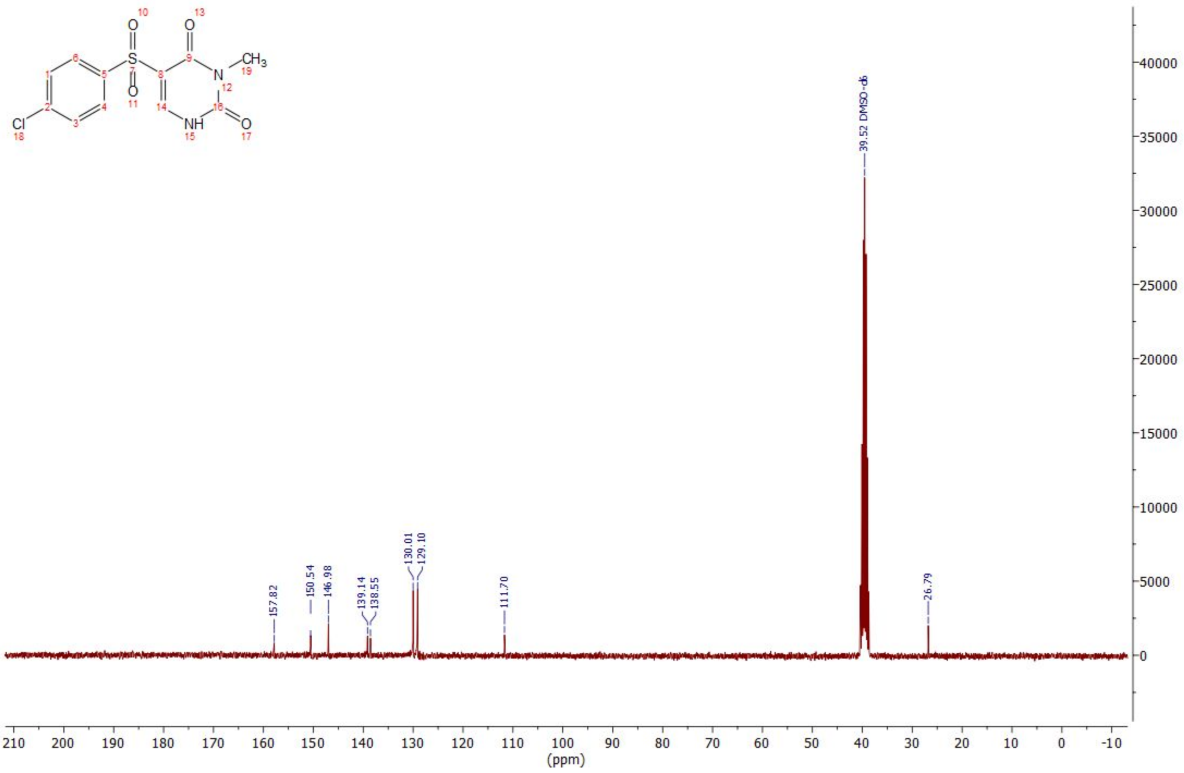

Figure S 36. *^{1}H (300 MHz) and ^{13}C{1H} (75 MHz) NMR-Spectra in DMSO-d_6 of **6c**.*

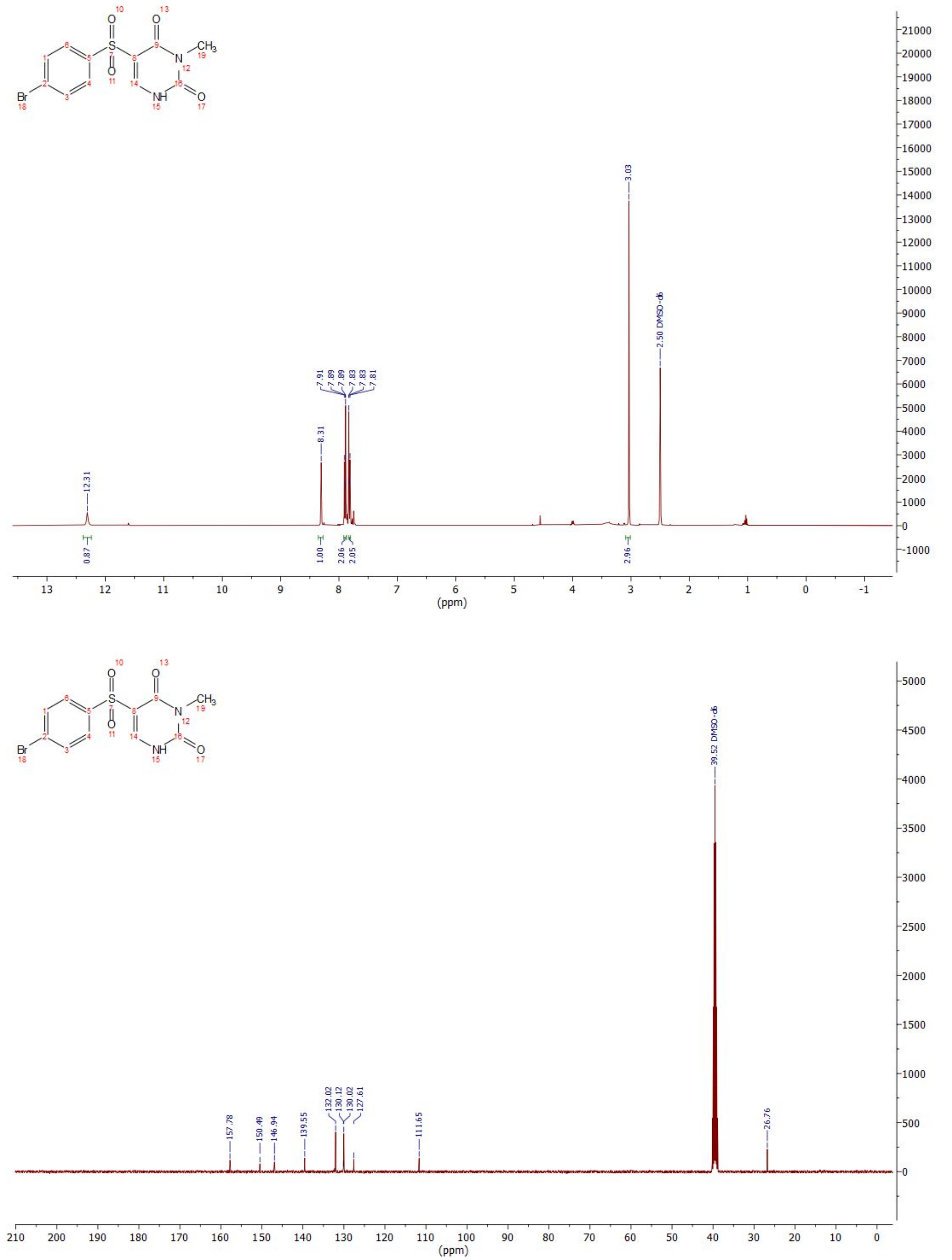

Figure S 37. *^{1}H (400 MHz) and $^{13}C\{1H\}$ (101 MHz) NMR-Spectra in DMSO-d_6 of **6d**.*

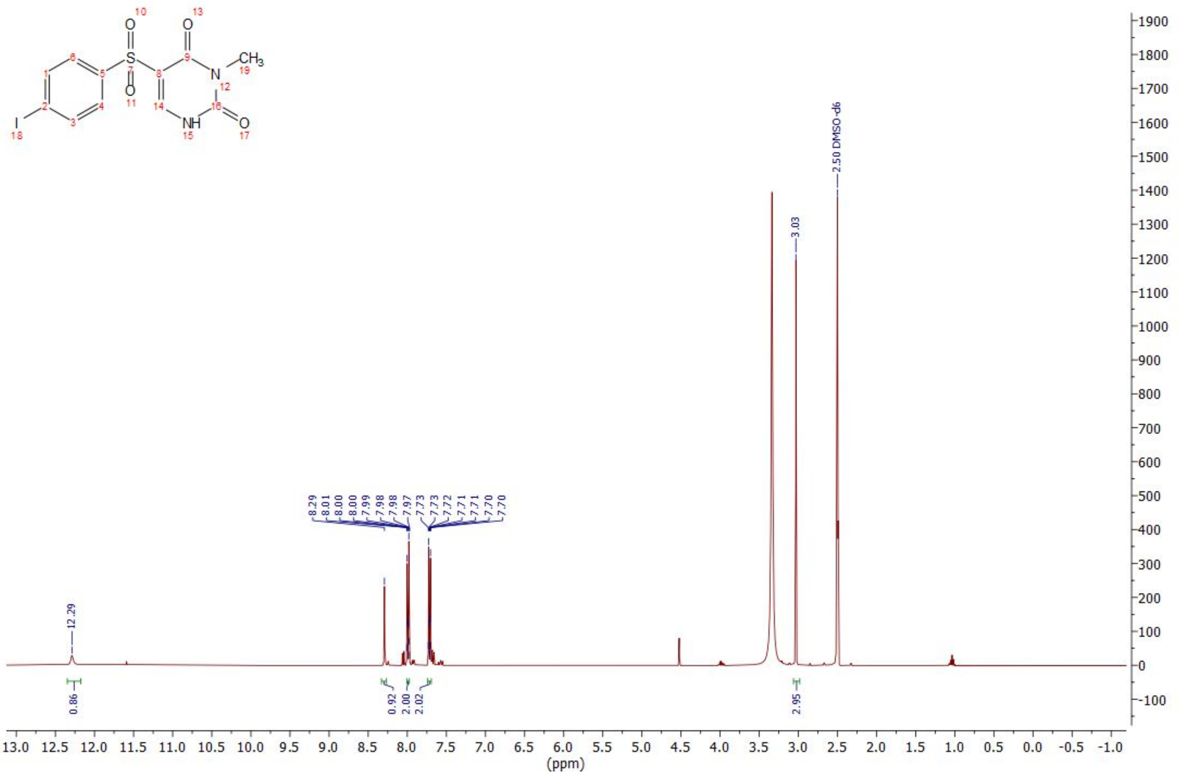

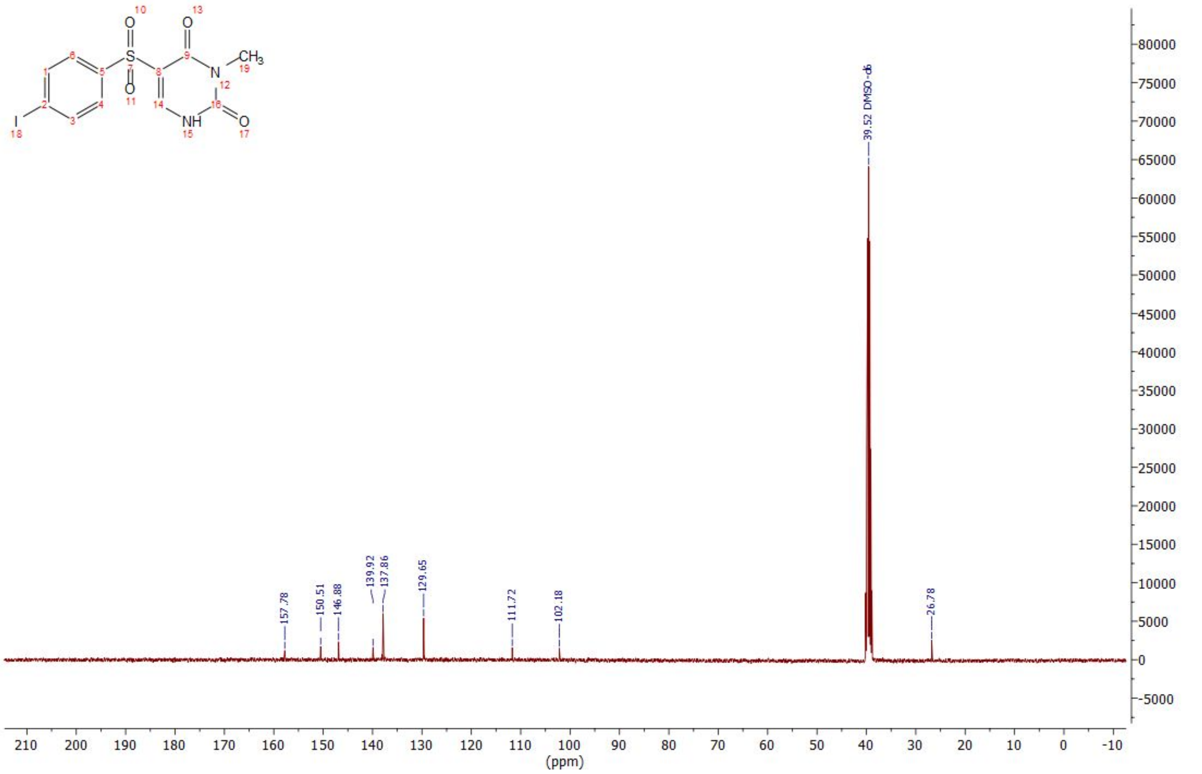

Figure S 38. *^{1}H (400 MHz) and ^{13}C{1H} (101 MHz) NMR-Spectra in DMSO-d_6 of* ***6e****.*

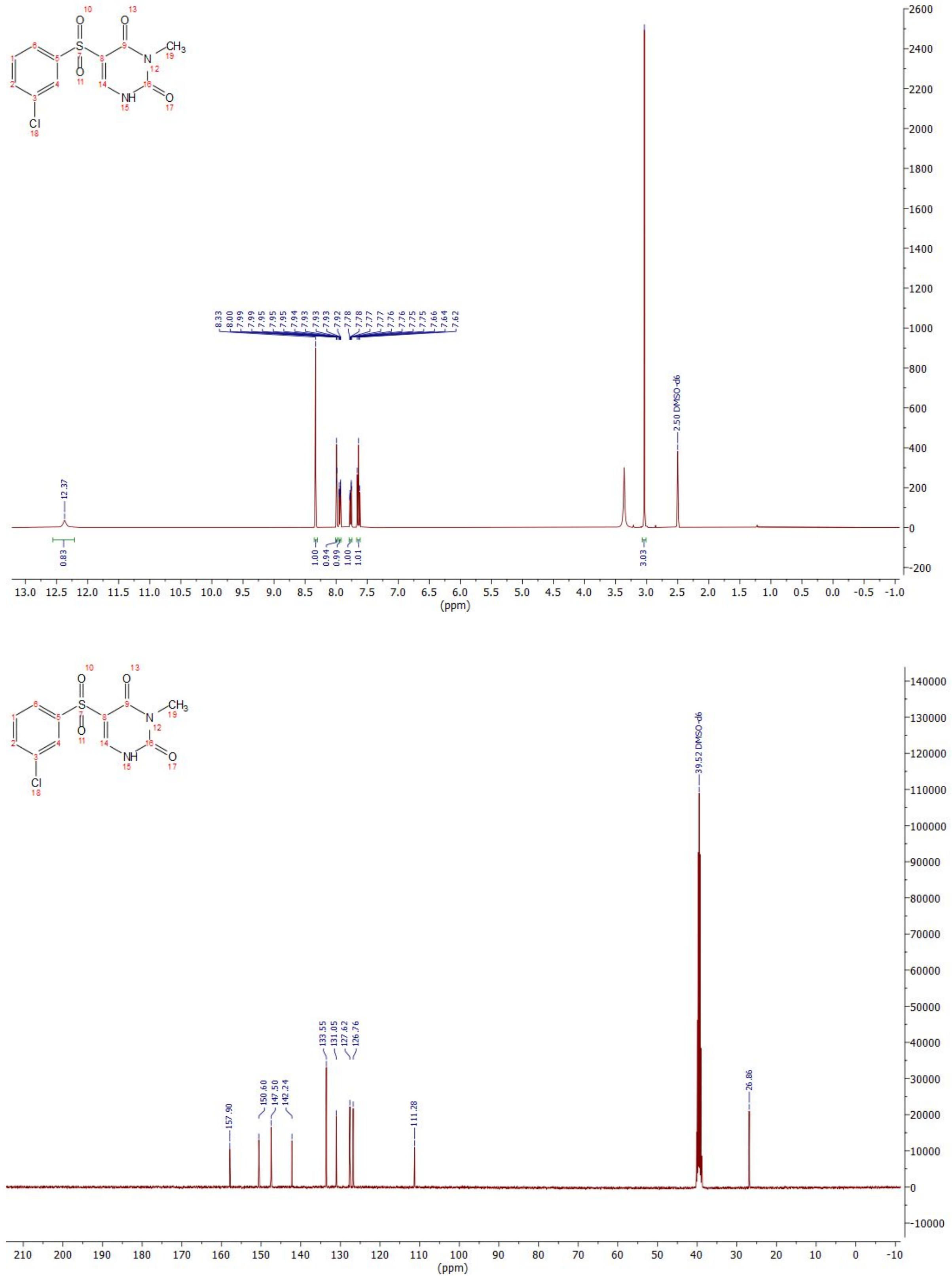

Figure S 39. *^{1}H (400 MHz) and ^{13}C{1H} (101 MHz) NMR-Spectra in DMSO-d_6 of **6f**.*

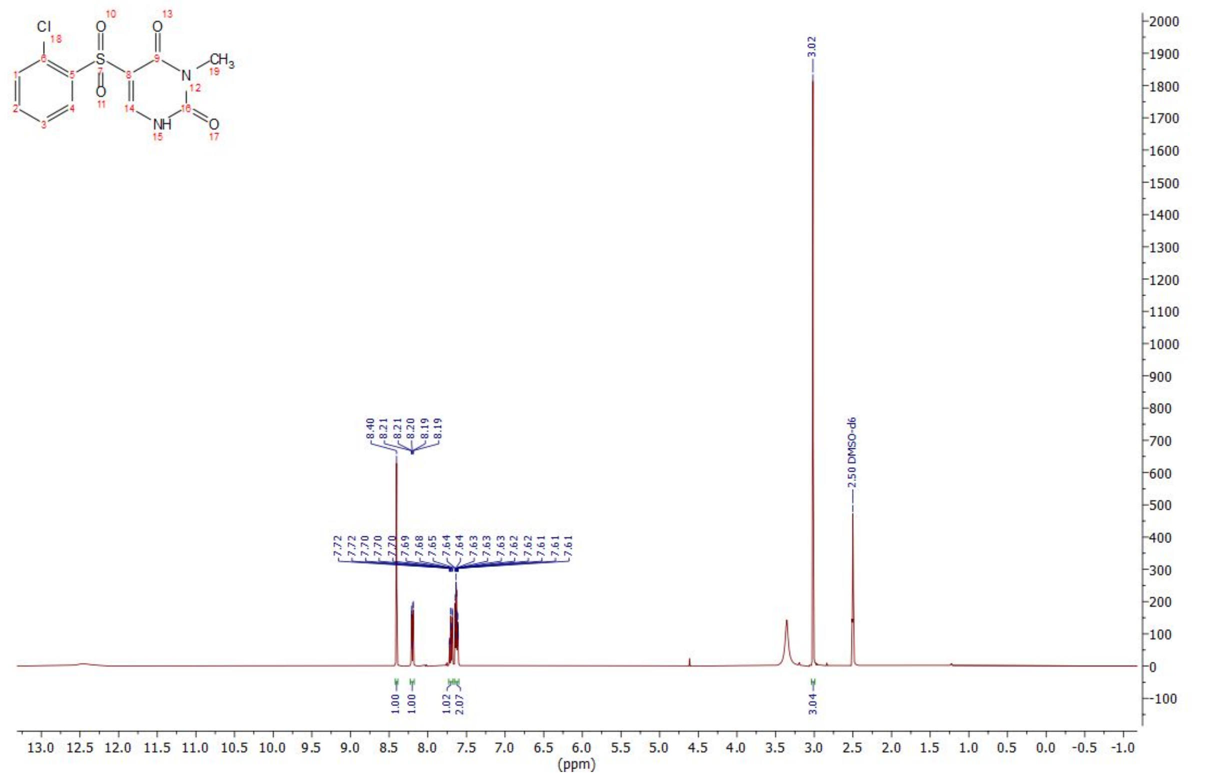

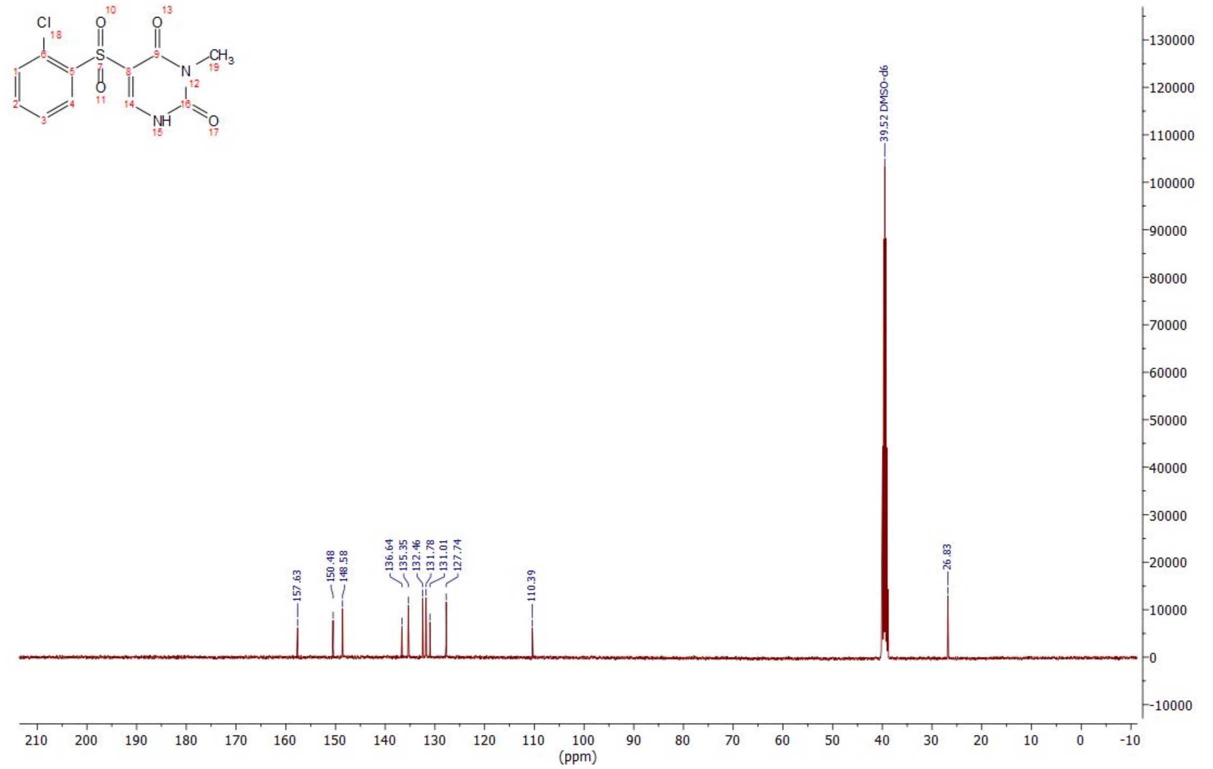

Figure S 40. *^{1}H (400 MHz) and $^{13}C\{1H\}$ (101 MHz) NMR-Spectra in DMSO-d_6 of* ***6g****.*

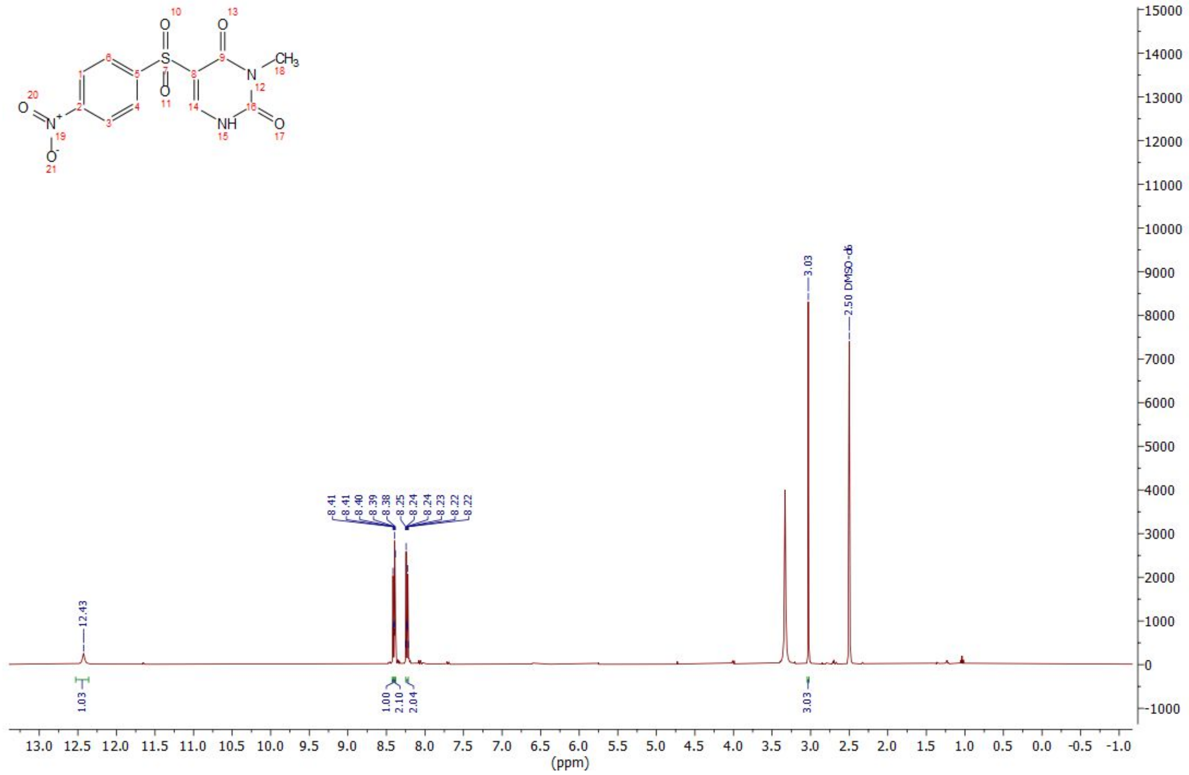

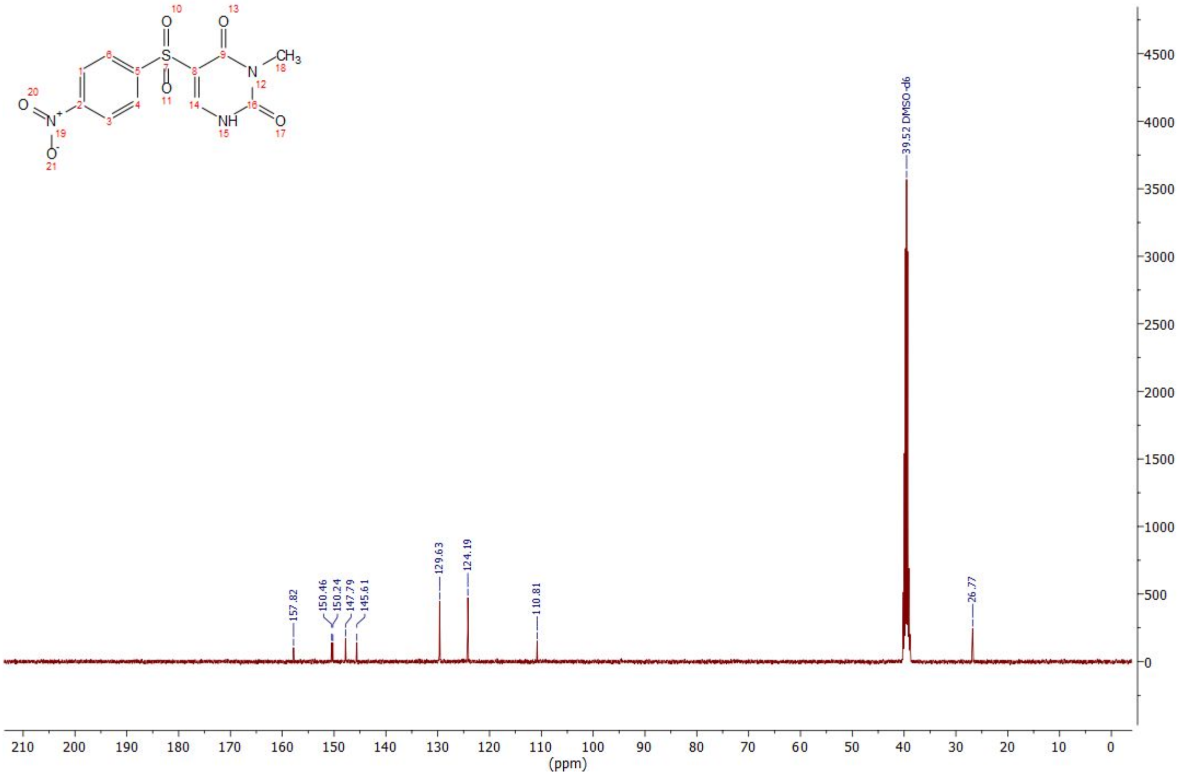

Figure S 41. *^{1}H (400 MHz) and ^{13}C{1H} (101 MHz) NMR-Spectra in DMSO-d$_6$ of* ***6h****.*

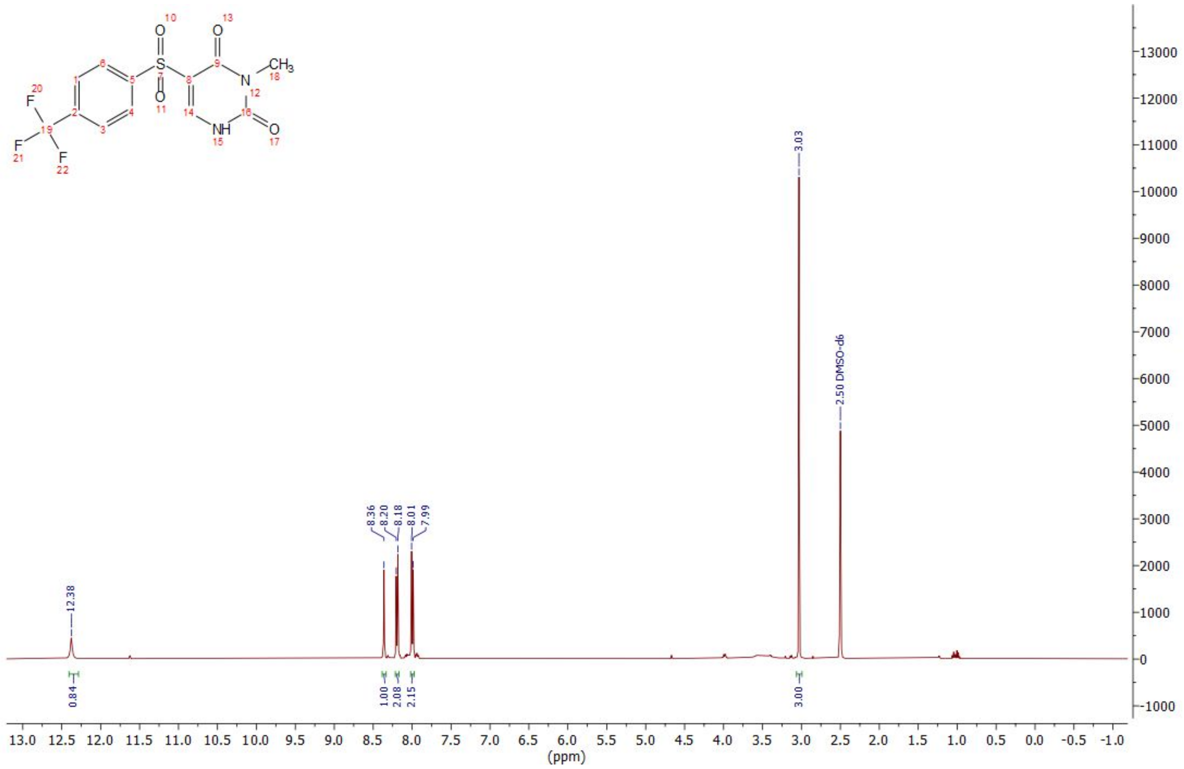

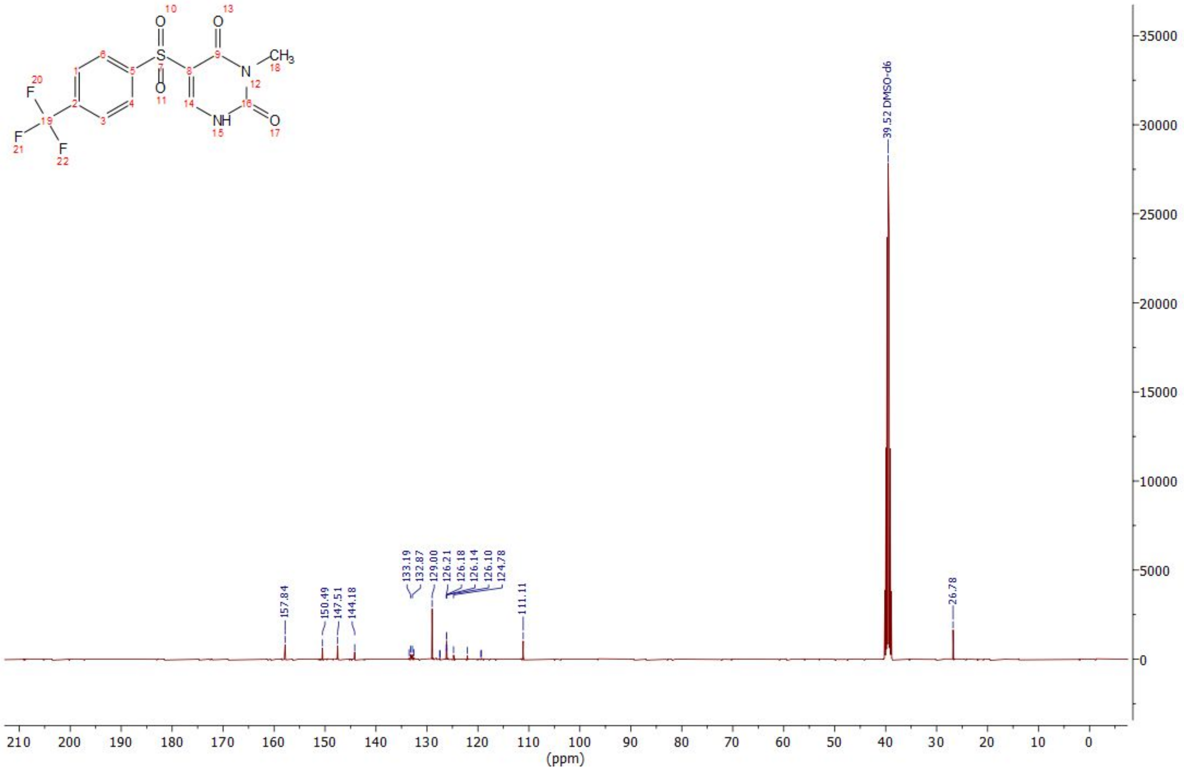

Figure S 42. *^{1}H (400 MHz) and $^{13}C\{1H\}$ (101 MHz) NMR-Spectra in DMSO-d_6 of **6i**.*

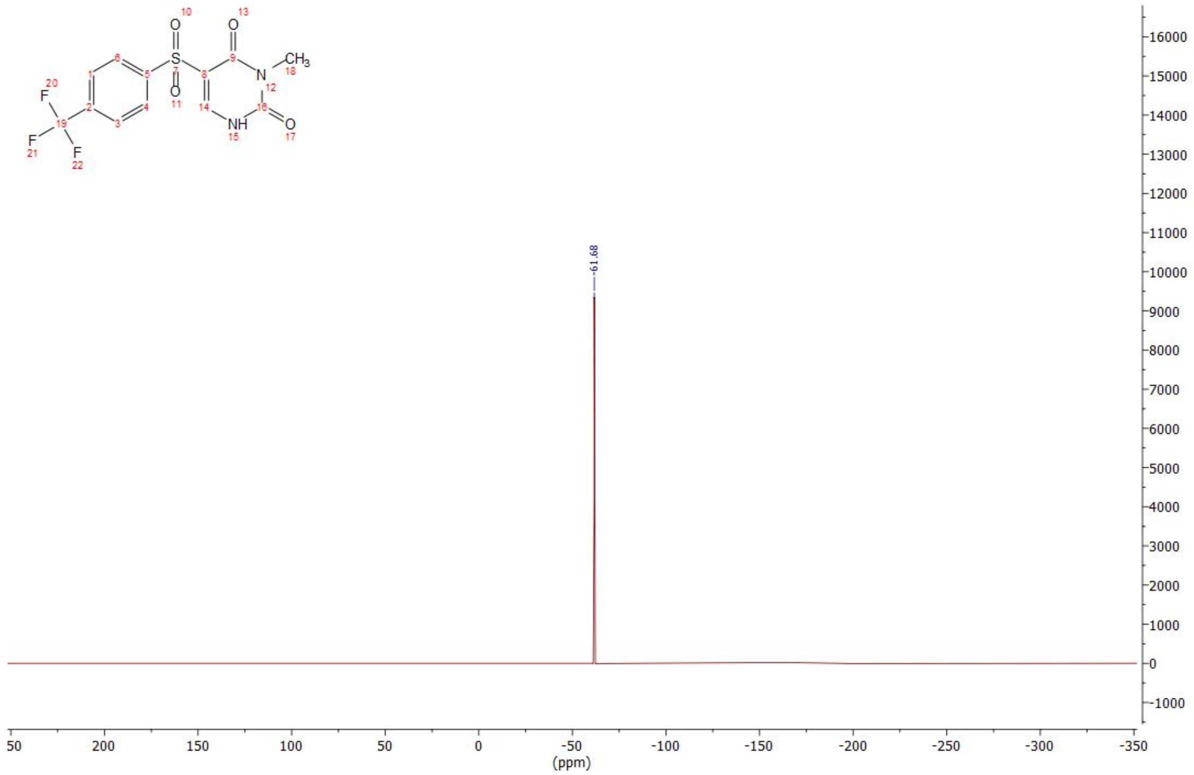

Figure S 43. *^{19}F (376 MHz) NMR-Spectra in DMSO-d_6 of **6i**.*

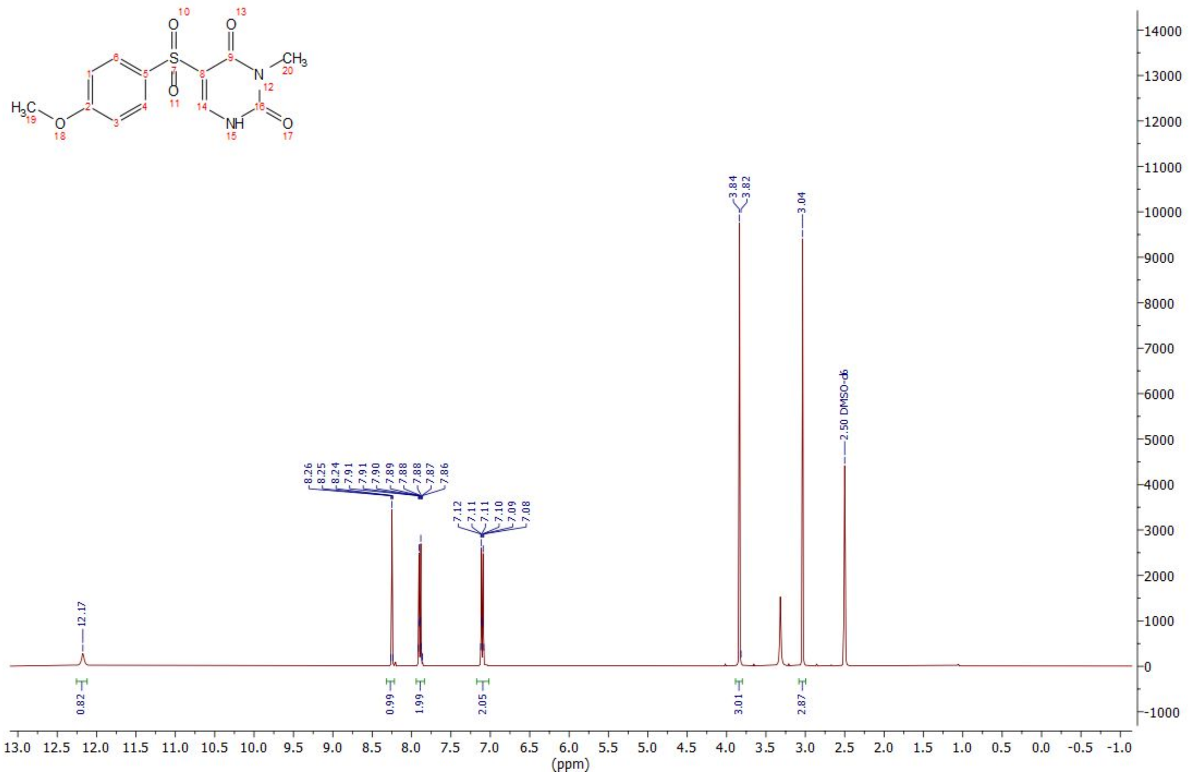

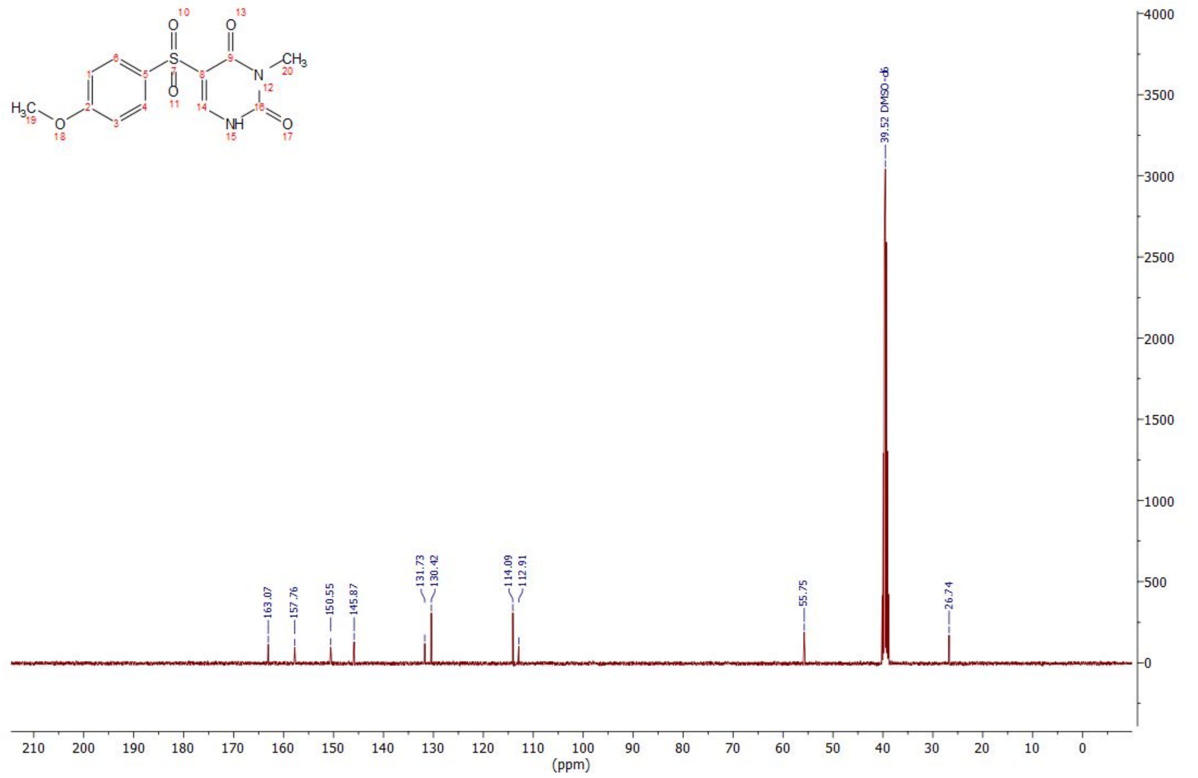

Figure S 44. *¹H (400 MHz) and ¹³C{1H} (101 MHz) NMR-Spectra in DMSO-d$_6$ of **6j**.*

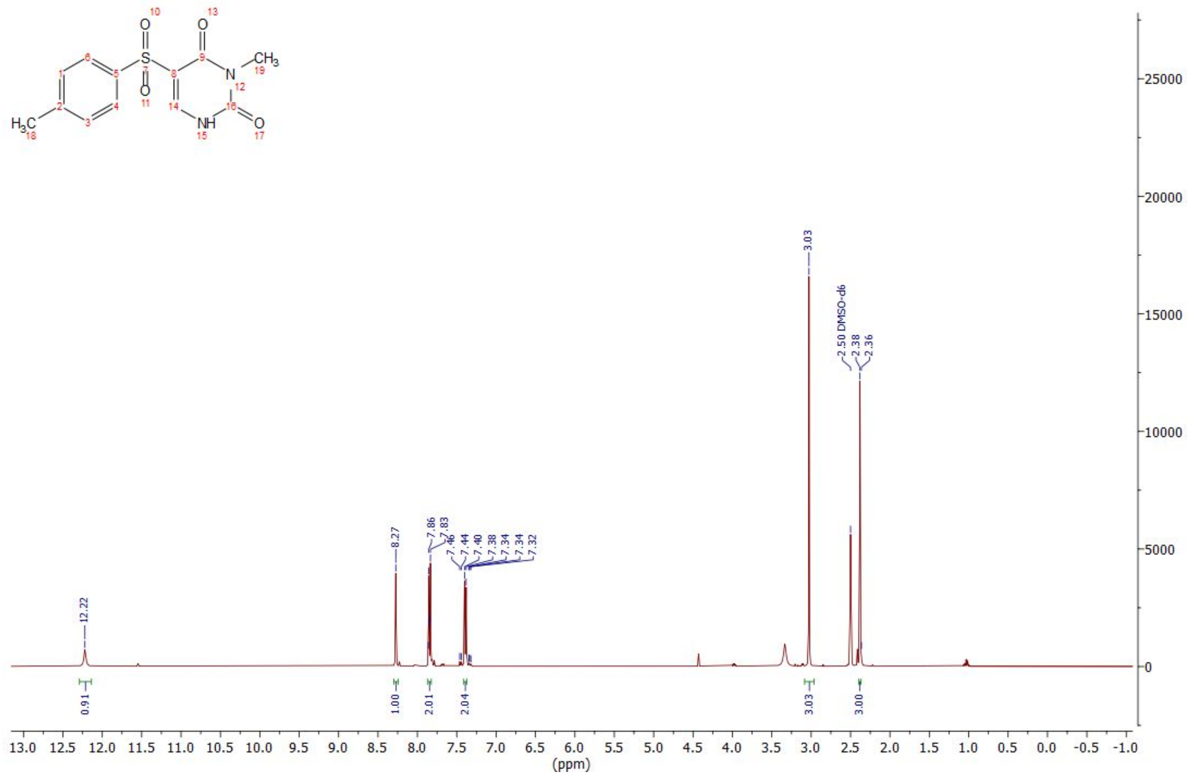

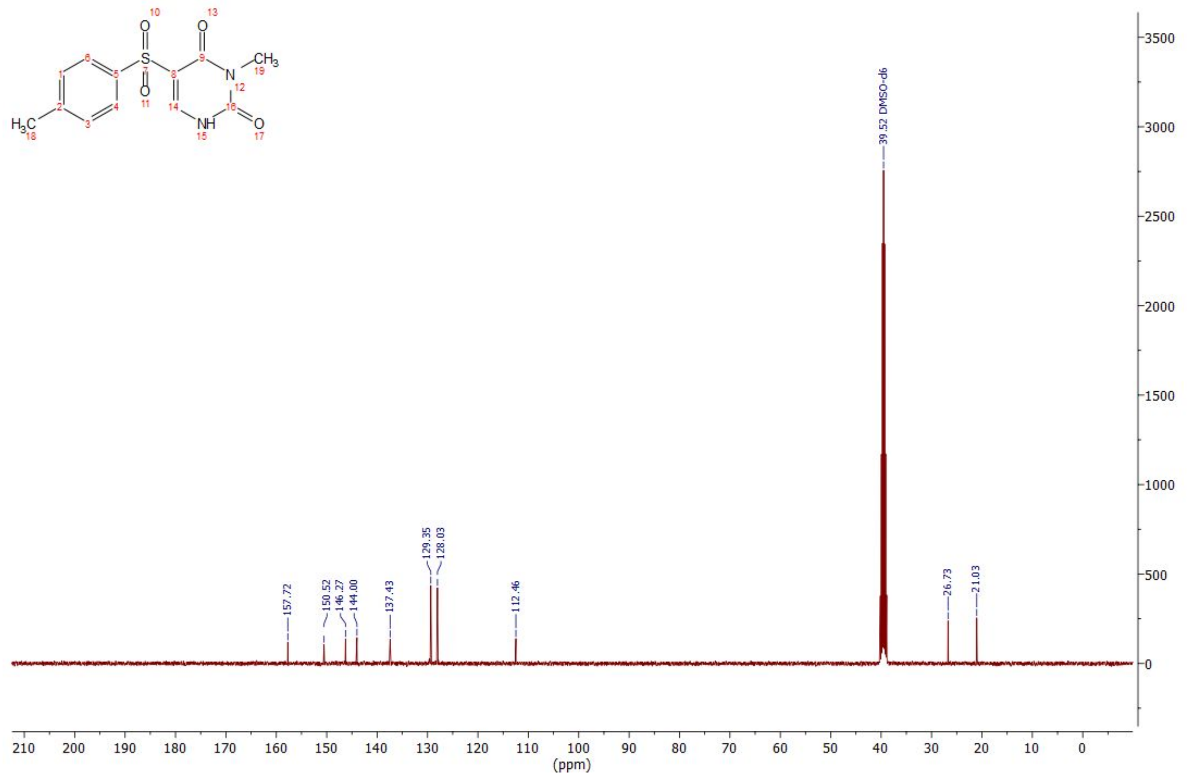

Figure S 45. *^{1}H (400 MHz) and $^{13}C\{1H\}$ (101 MHz) NMR-Spectra in DMSO-d_6 of* ***6k****.*

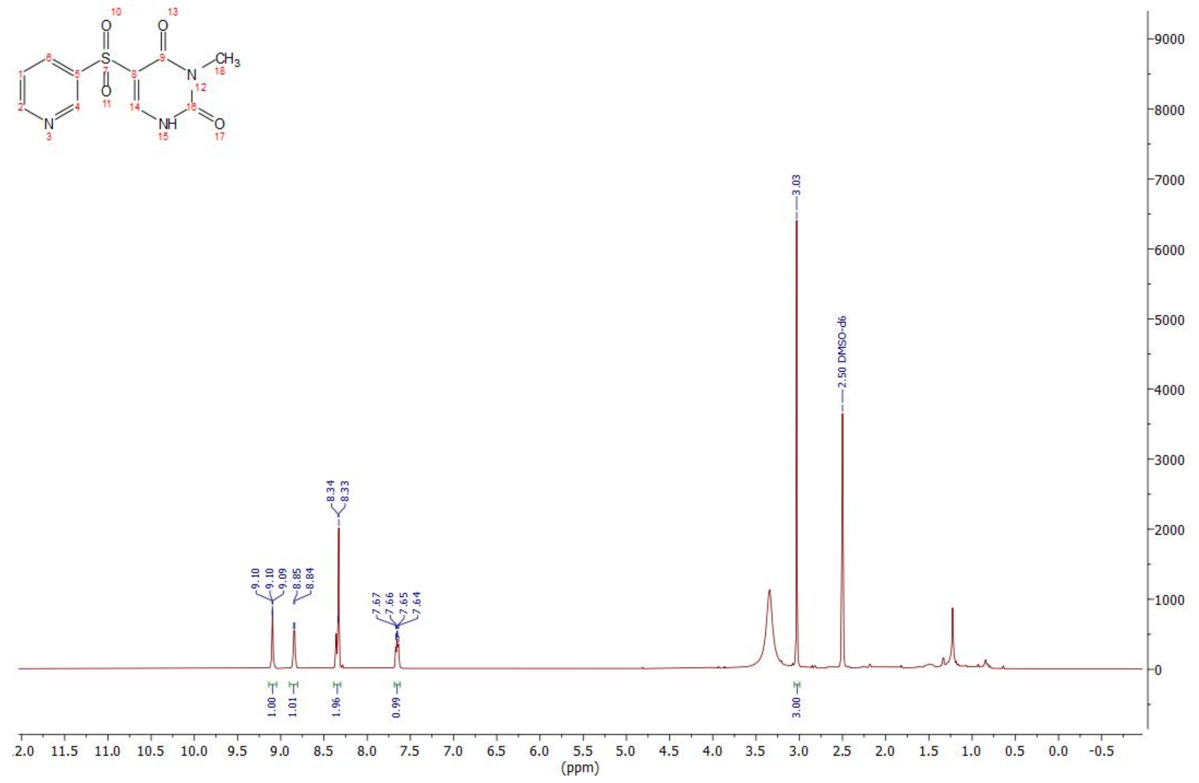

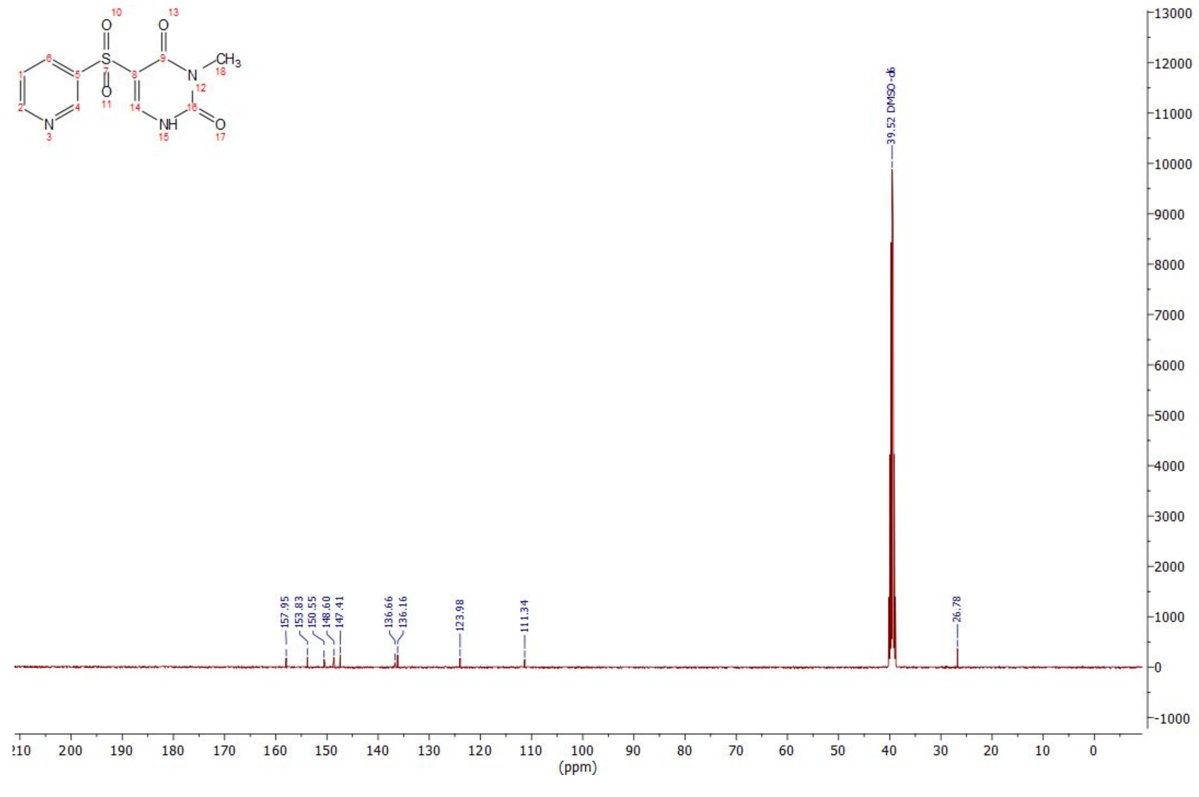

Figure S 46. *^{1}H (400 MHz) and $^{13}C\{1H\}$ (101 MHz) NMR-Spectra in DMSO-d_6 of **6l**.*

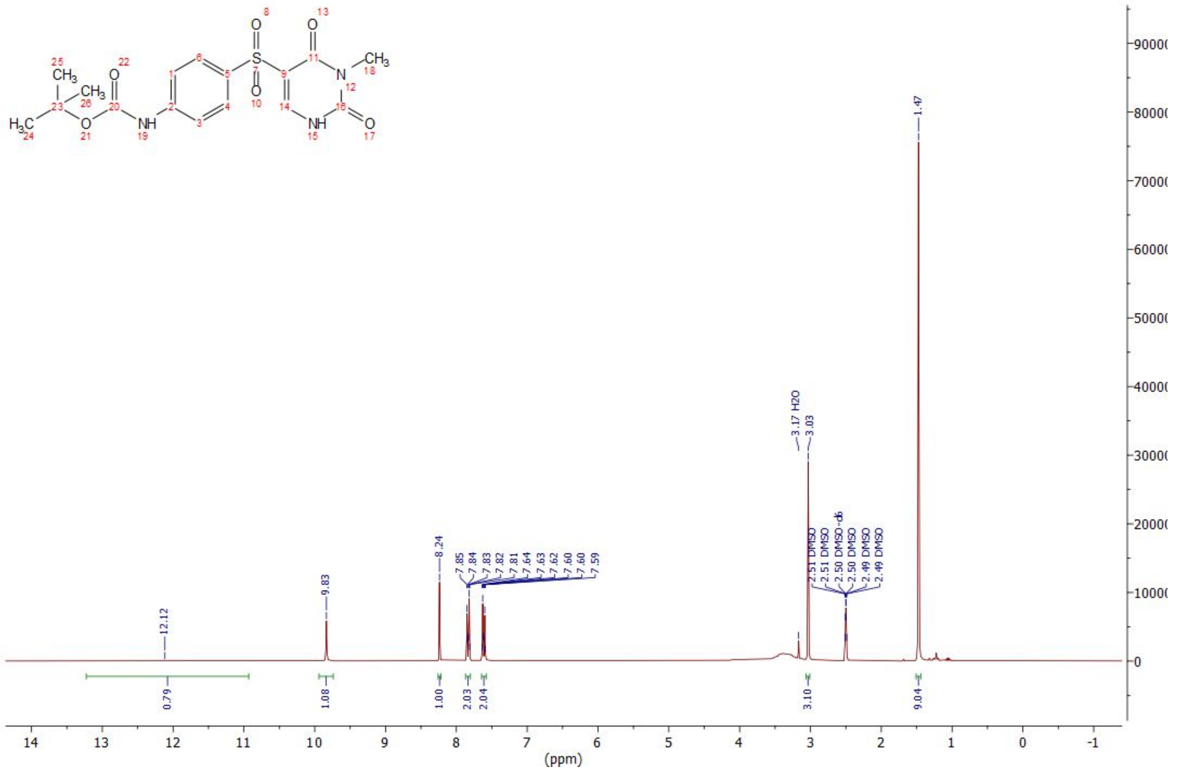

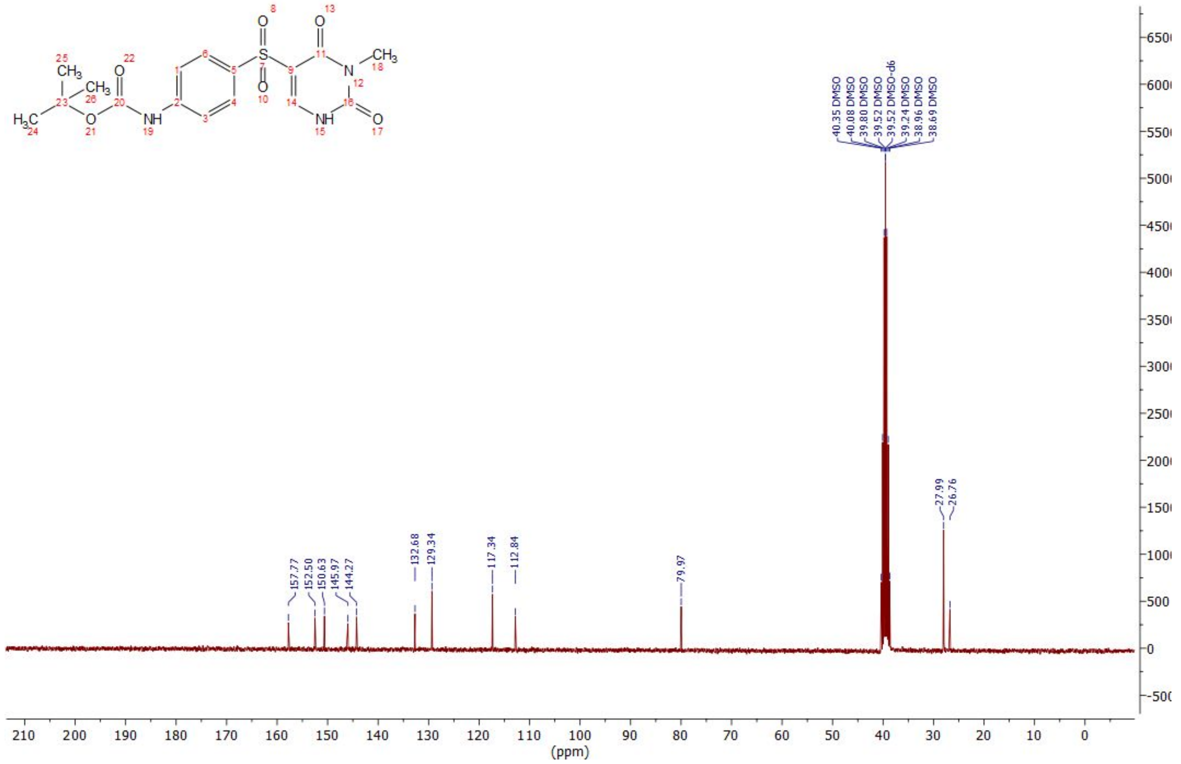

Figure S 47. *^{1}H (300 MHz) and $^{13}C\{^{1}H\}$ (76 MHz) NMR-Spectra in DMSO-d_6 of* ***6m****.*

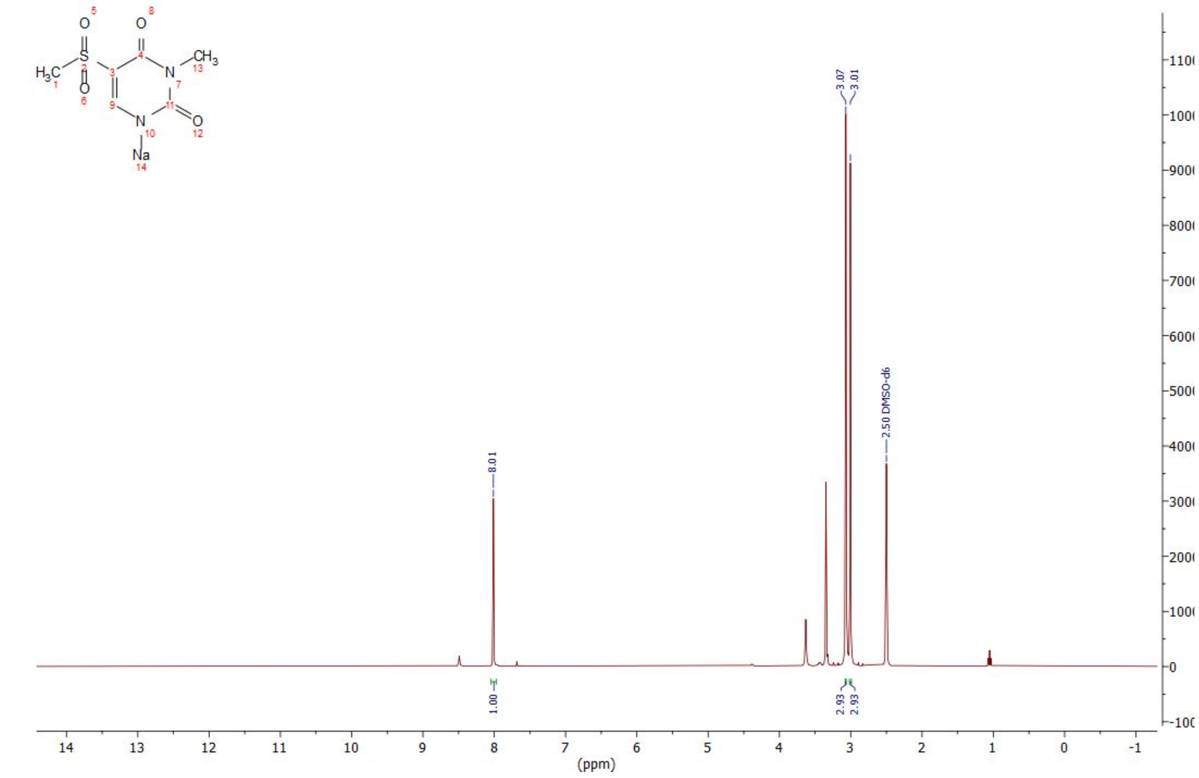

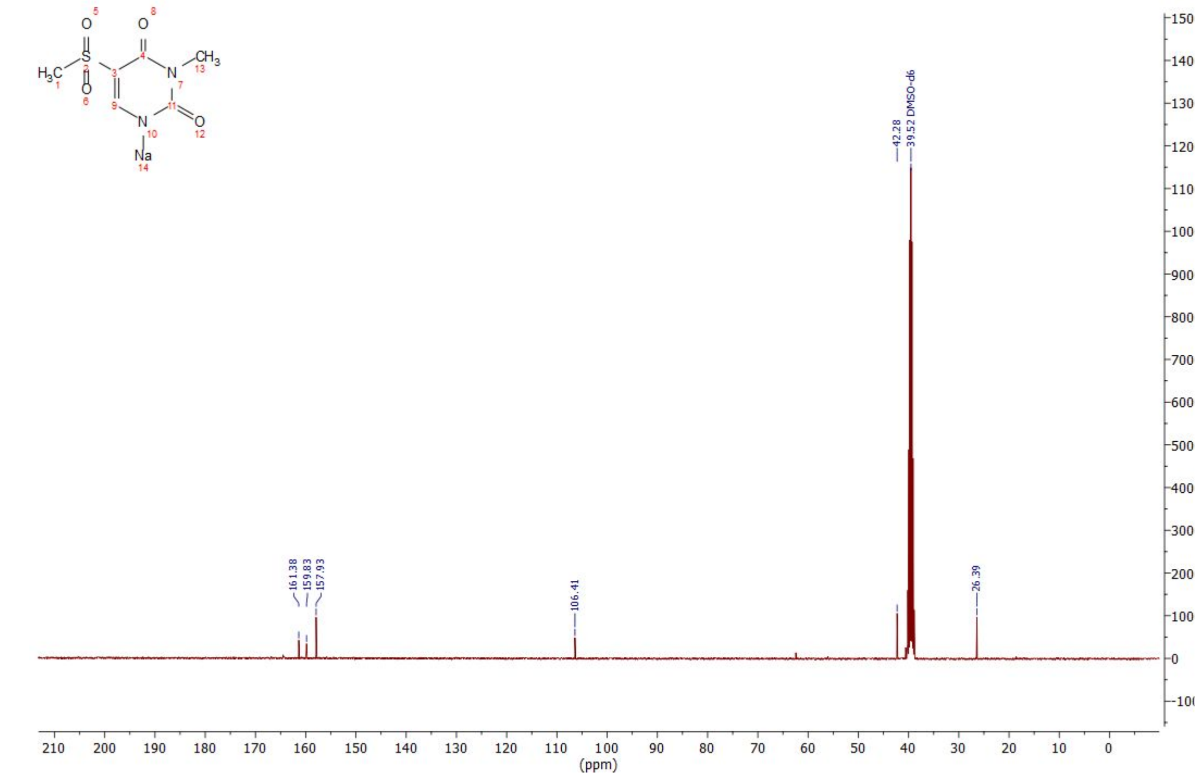

Figure S 48. *^{1}H (400 MHz) and $^{13}C\{^{1}H\}$ (101 MHz) NMR-Spectra in DMSO-d_6 of **6r**.*

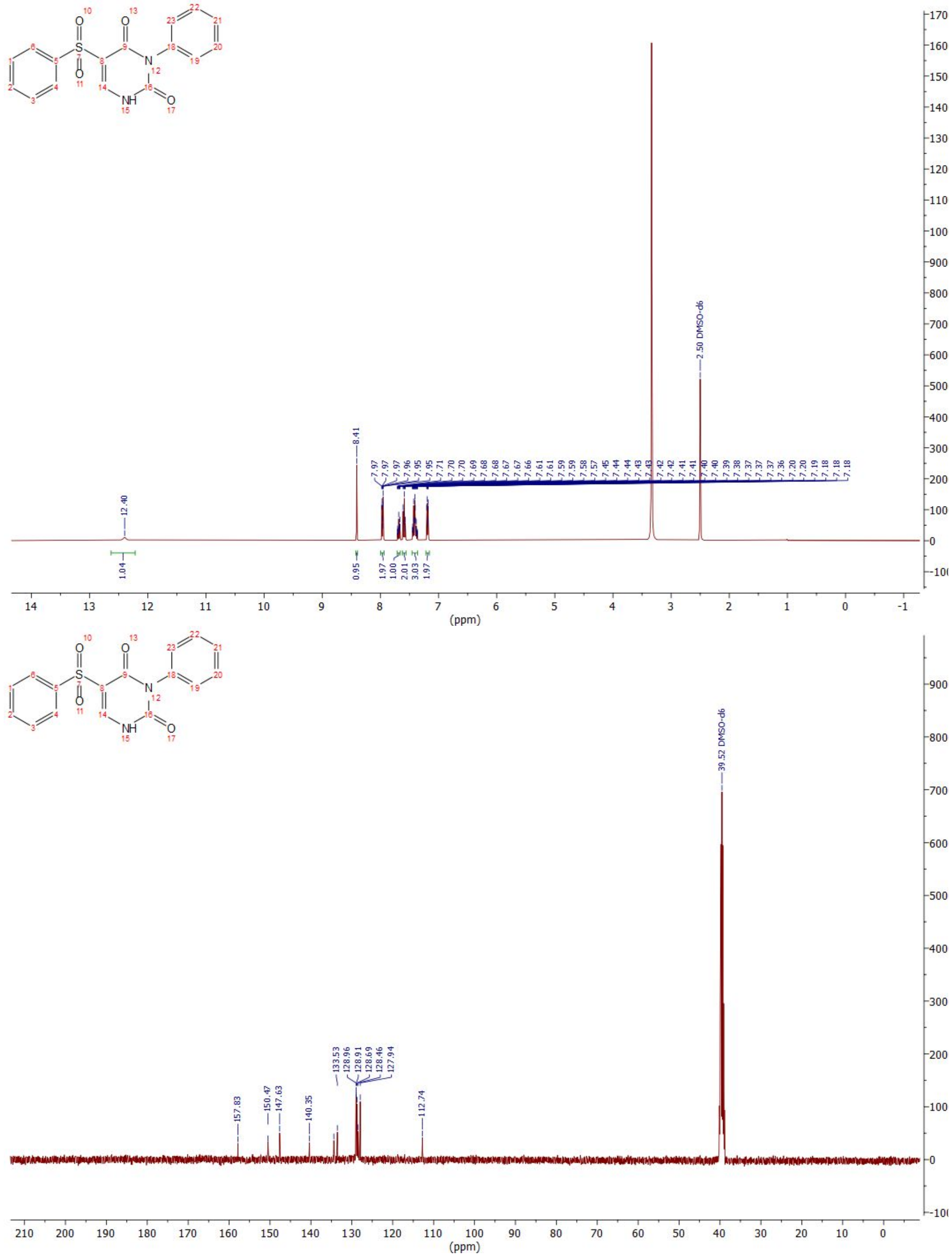

Figure S 49. *^{1}H (400 MHz) and ^{13}C{^{1}H} (101 MHz) NMR-Spectra in DMSO-d$_6$ of* ***7****.*

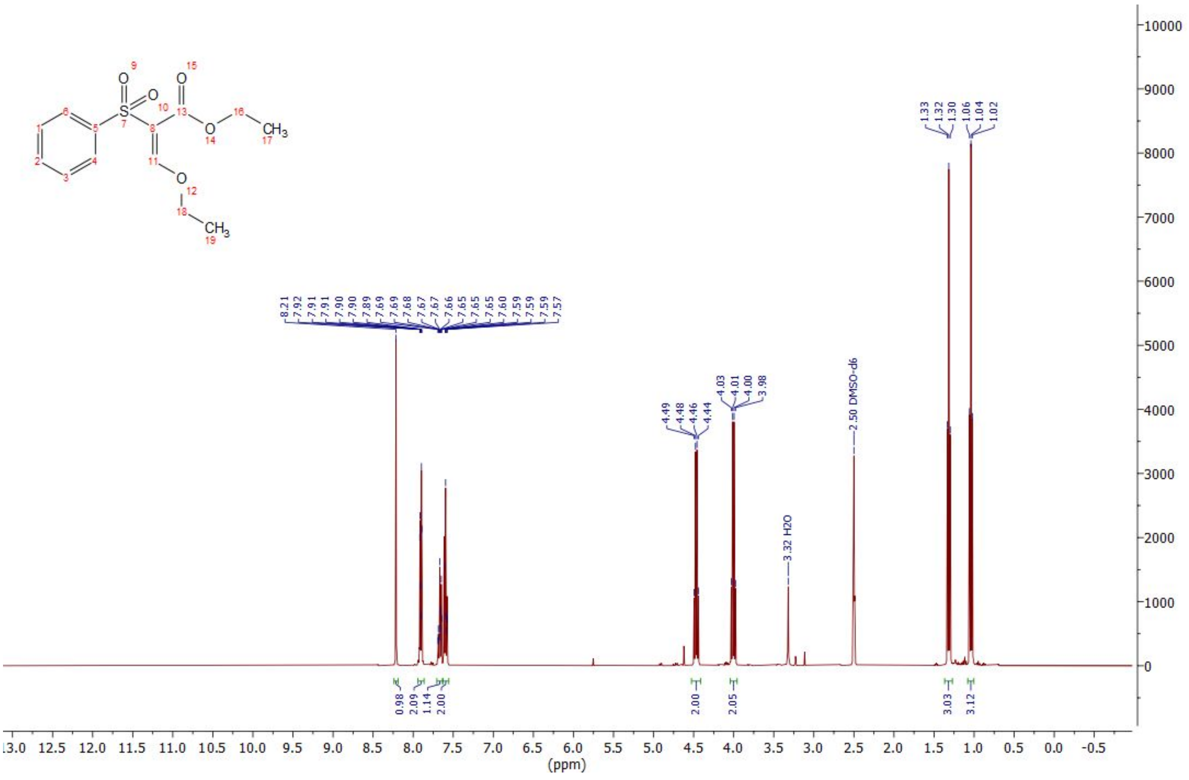

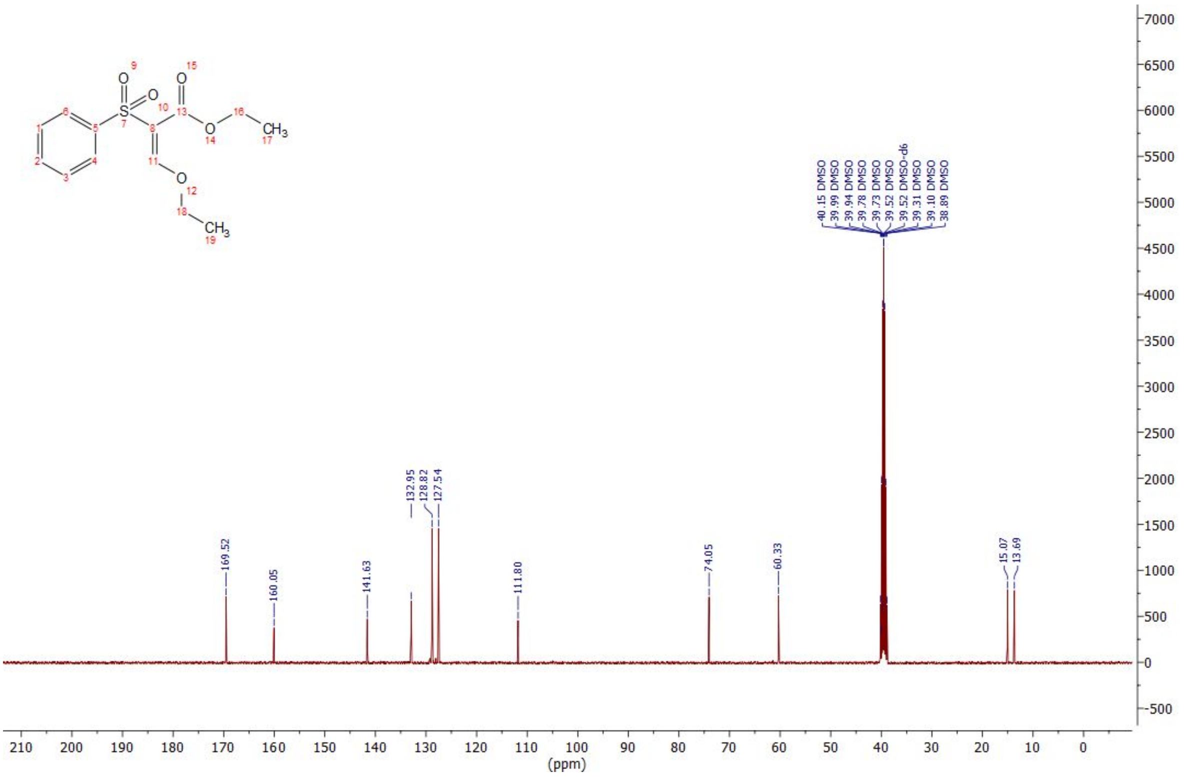

Figure S 50. *^{1}H (400 MHz) and $^{13}C\{1H\}$ (101 MHz) NMR-Spectra in DMSO-d_6 of **9a**.*

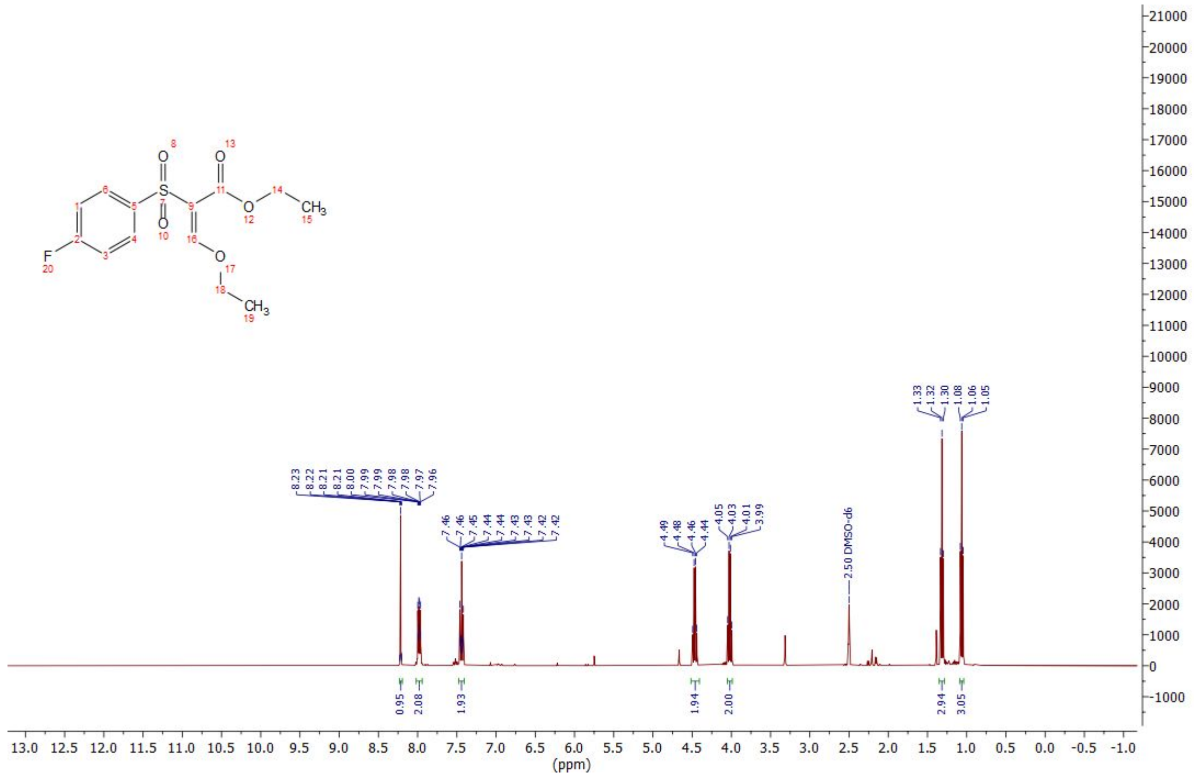

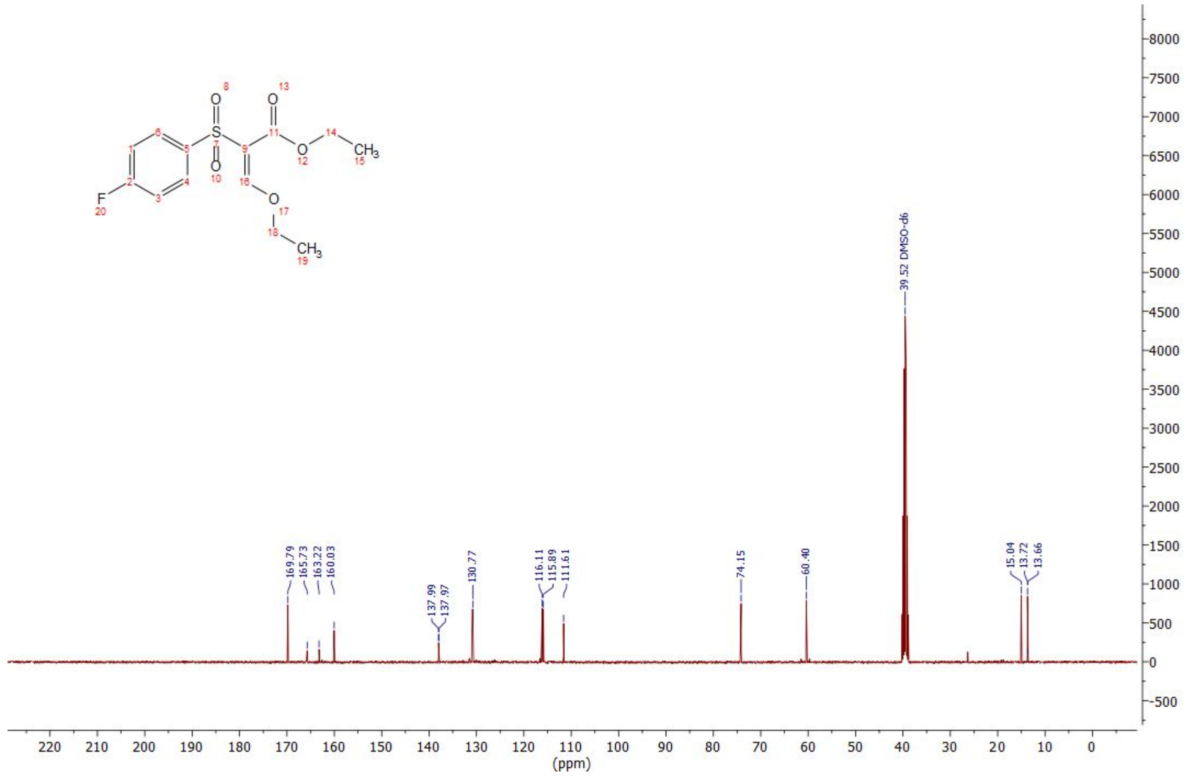

Figure S 51. *^{1}H (400 MHz) and ^{13}C{1H} (101 MHz)NMR-Spectra in DMSO-d$_6$ of **9b**.*

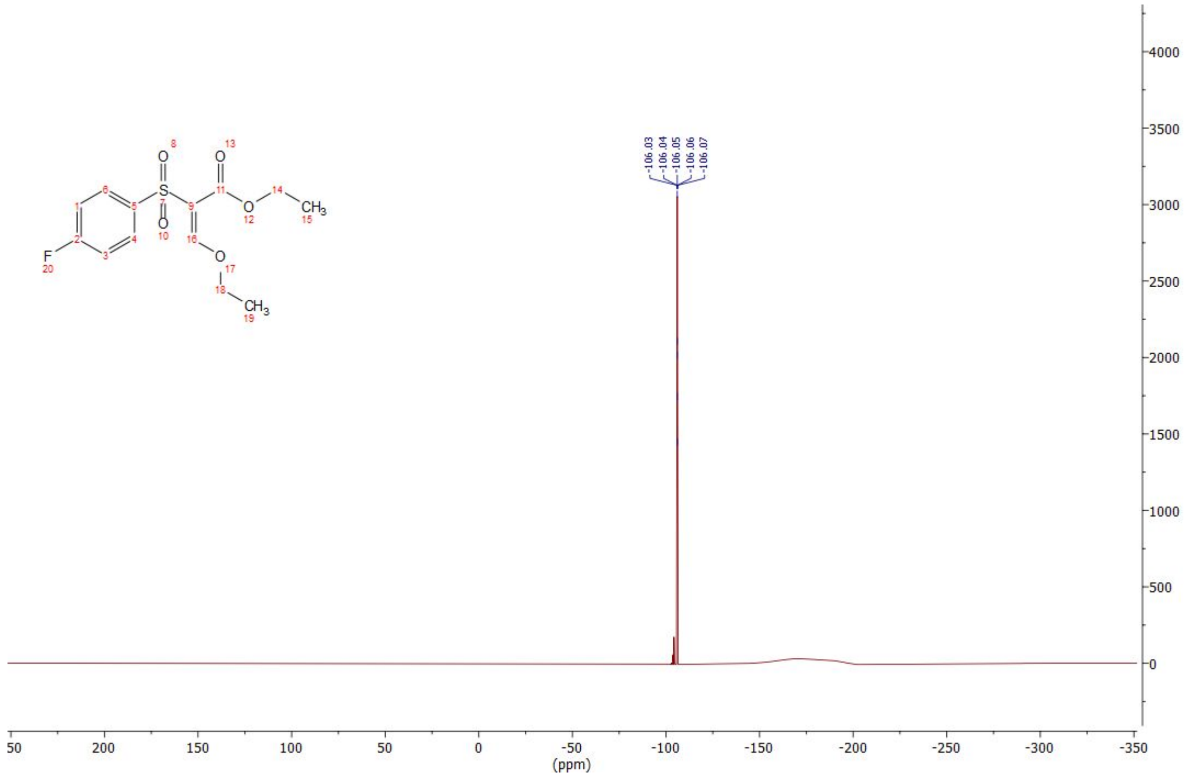

Figure S 52. *^{19}F (376 MHz) NMR-Spectra in DMSO-d$_6$ of* ***9b****.*

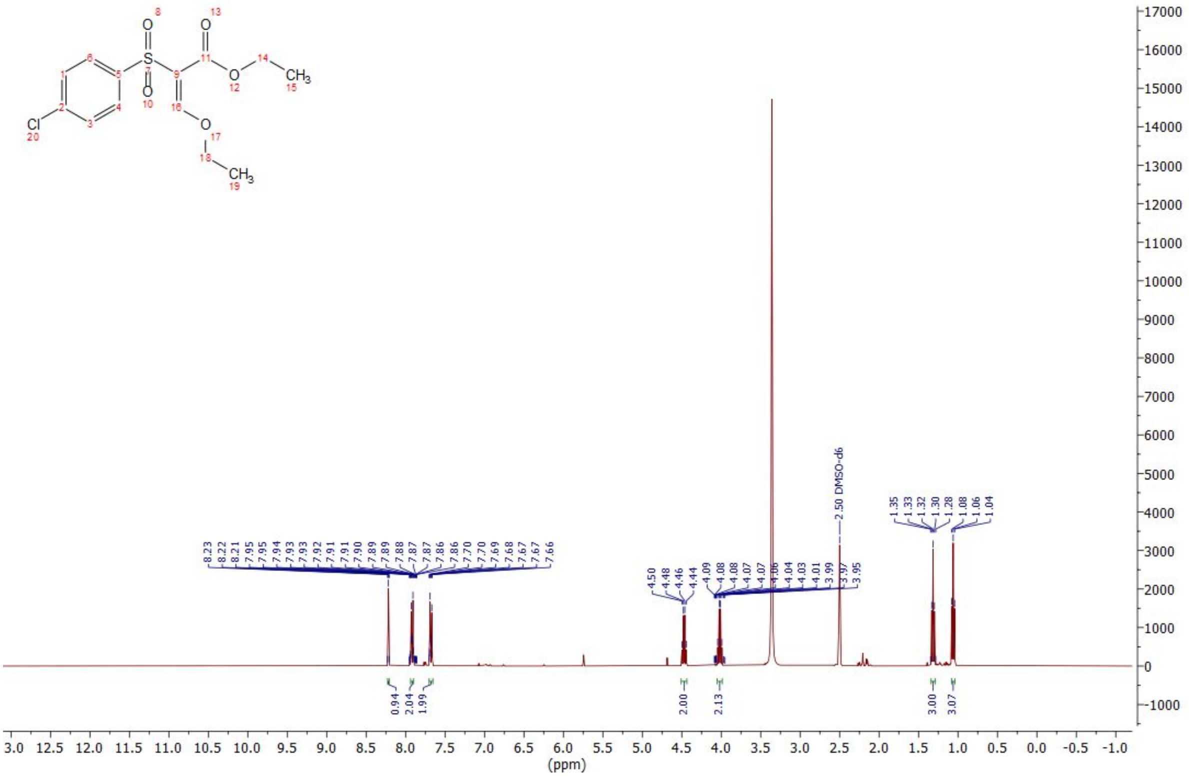

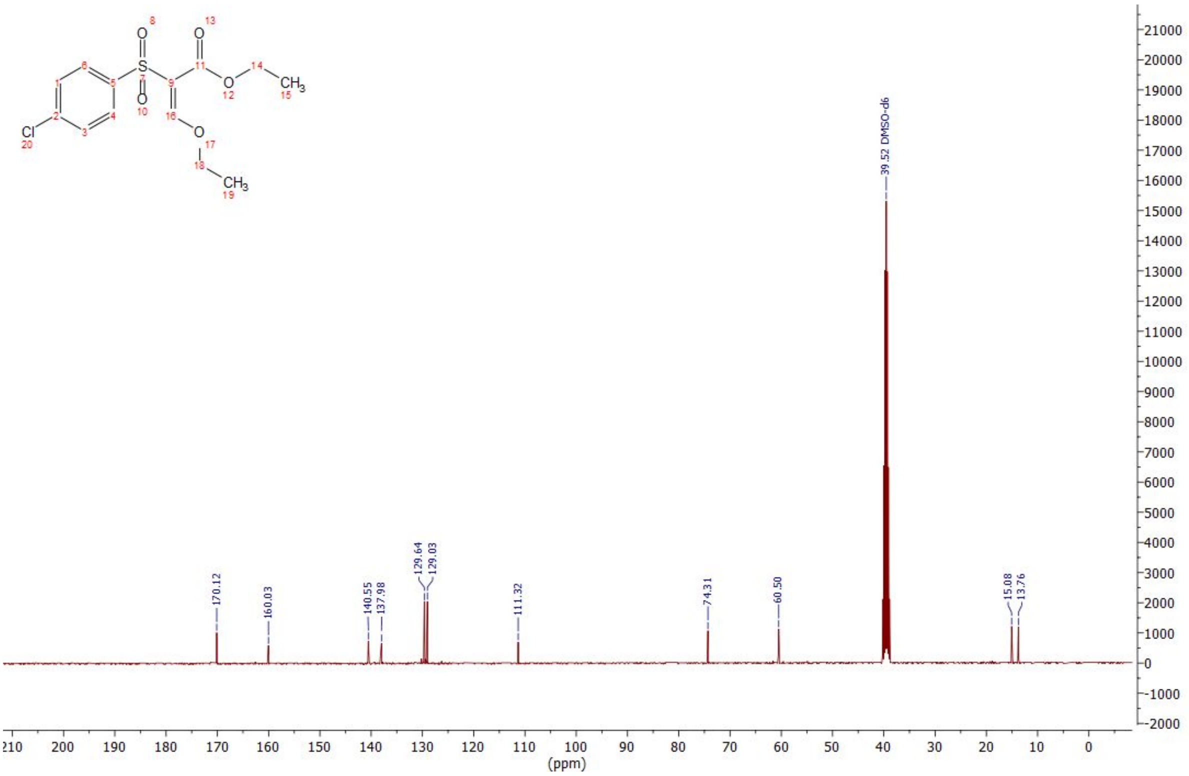

Figure S 53. *^{1}H (400 MHz) and $^{13}C\{1H\}$ (101 MHz) NMR-Spectra in DMSO-d_6 of **9c**.*

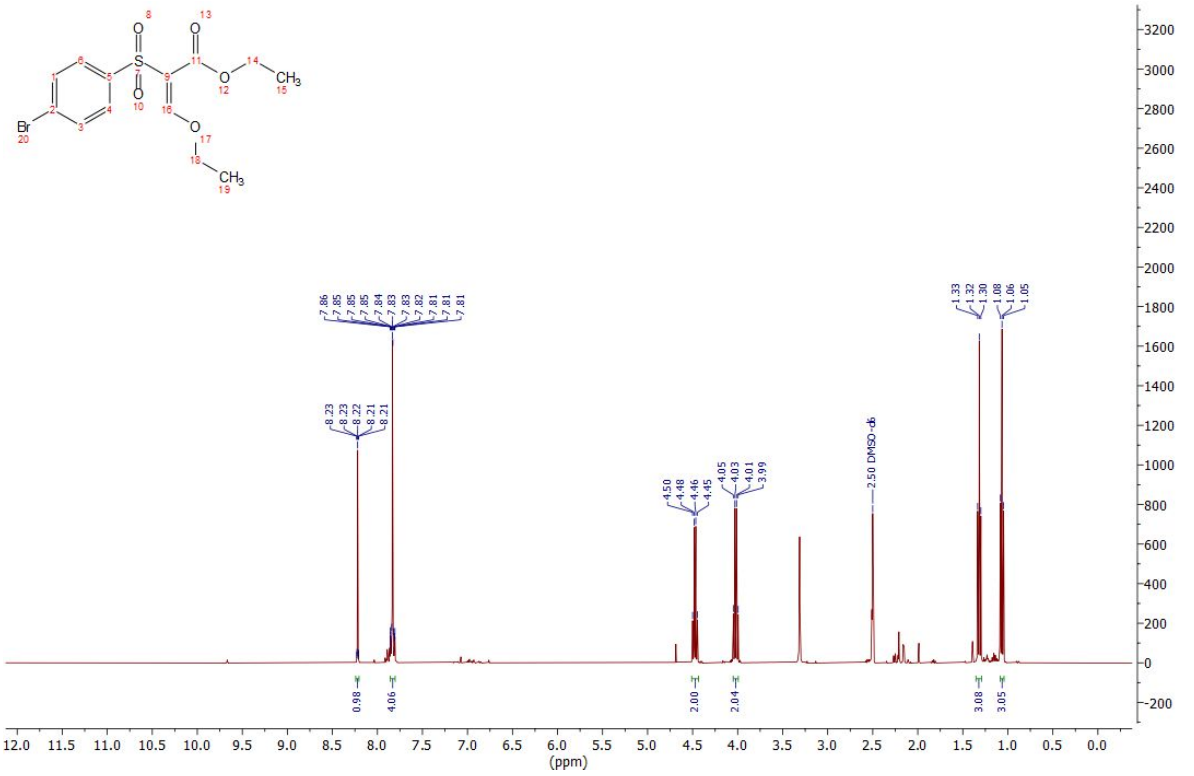

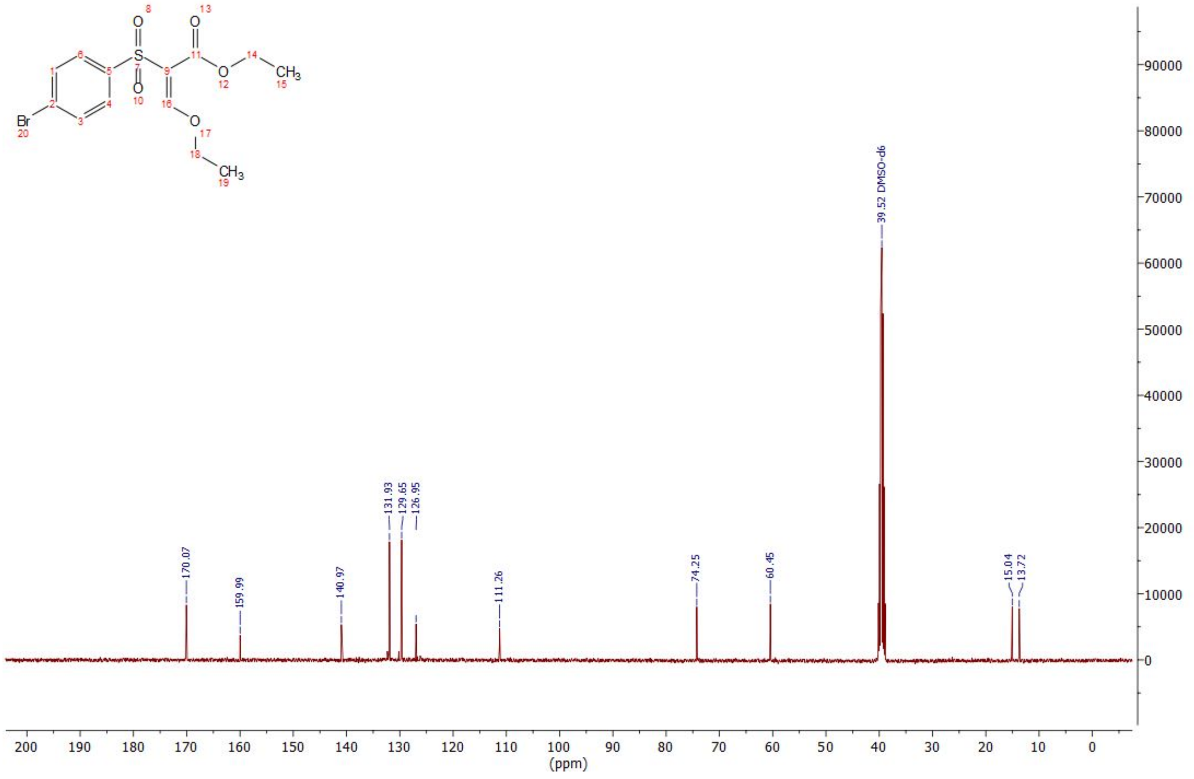

Figure S 54. *^{1}H (400 MHz) and ^{13}C{1H} (101 MHz) NMR-Spectra in DMSO-d_6 of **9d**.*

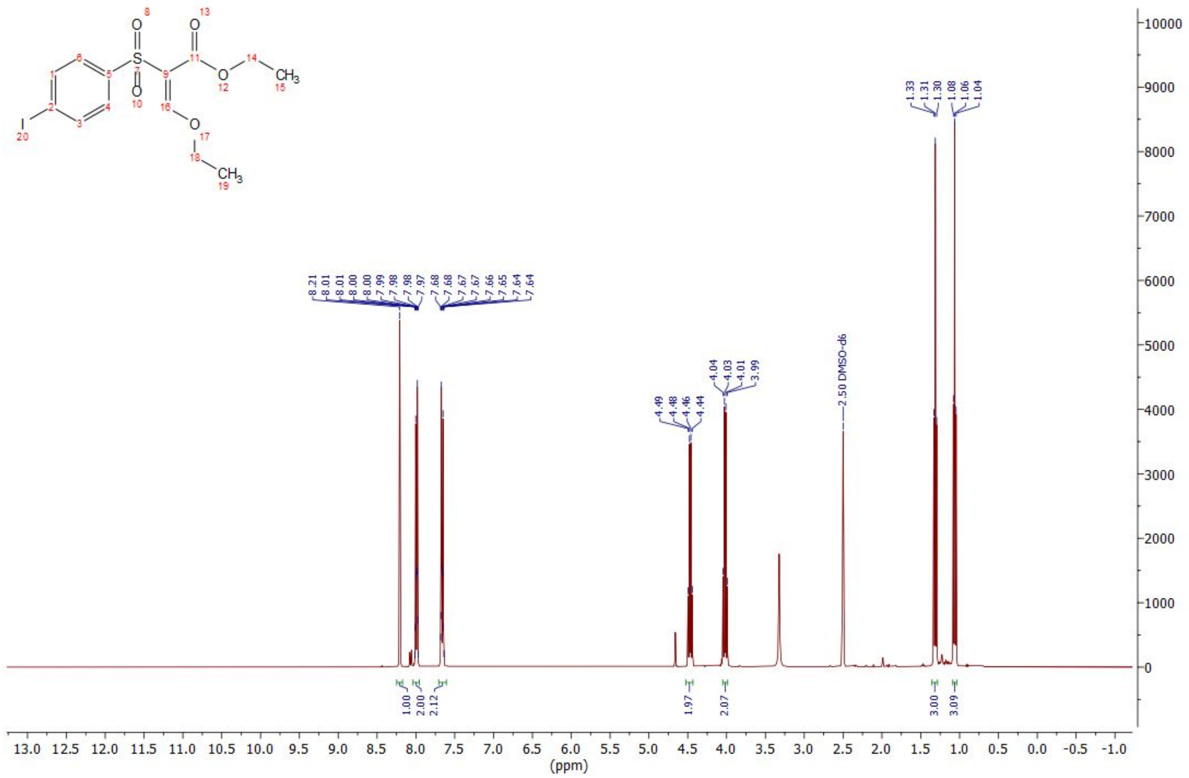

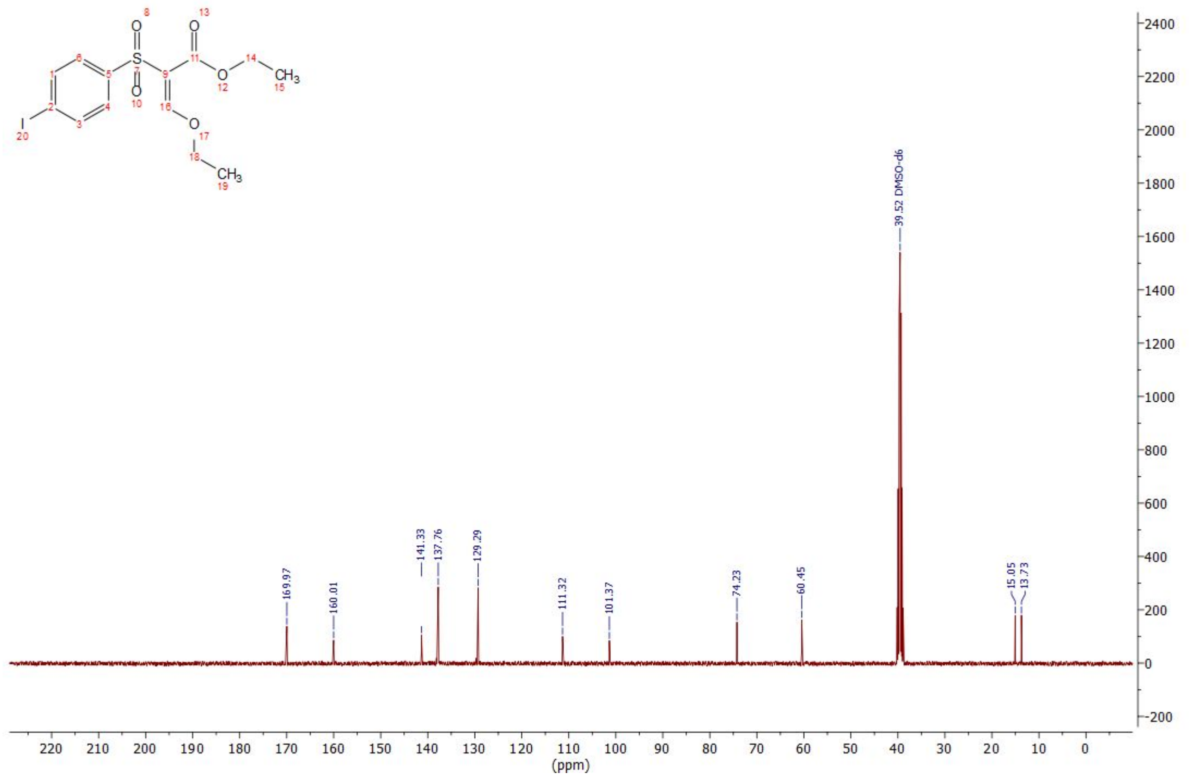

Figure S 55. *^{1}H (400 MHz) and $^{13}C\{1H\}$ (101 MHz) NMR-Spectra in DMSO-d_6 of* ***9e***.

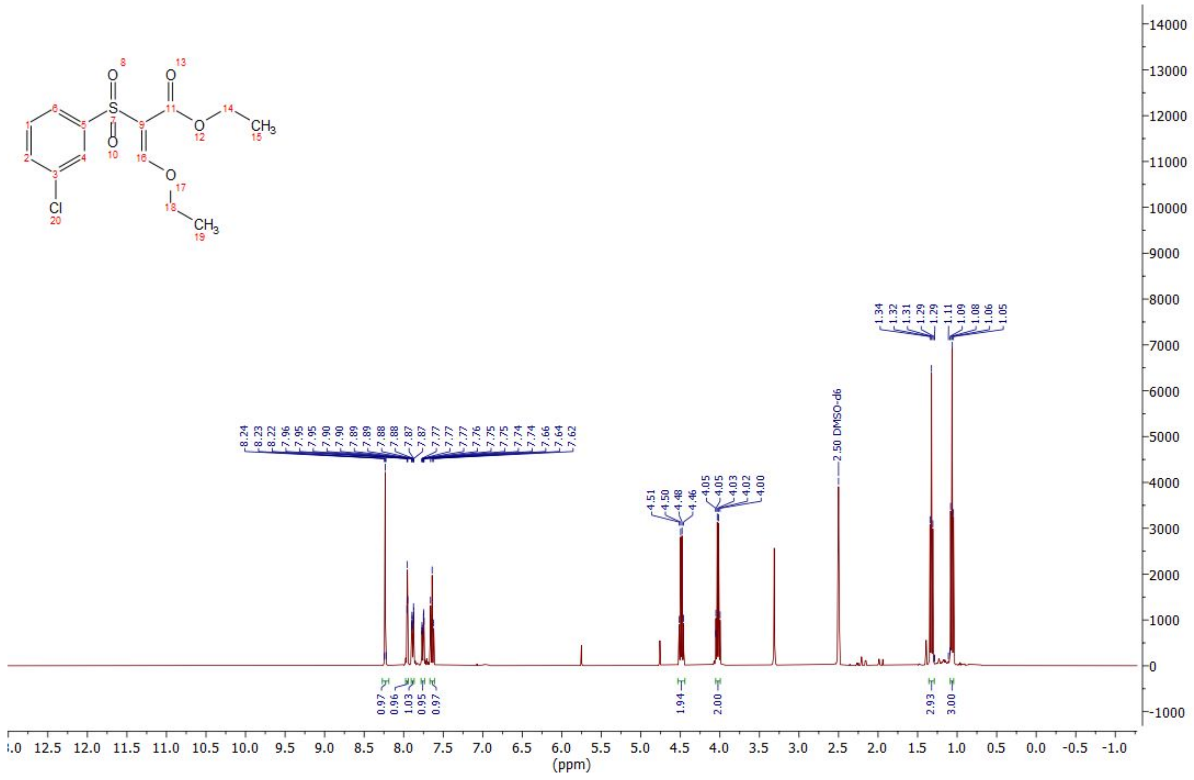

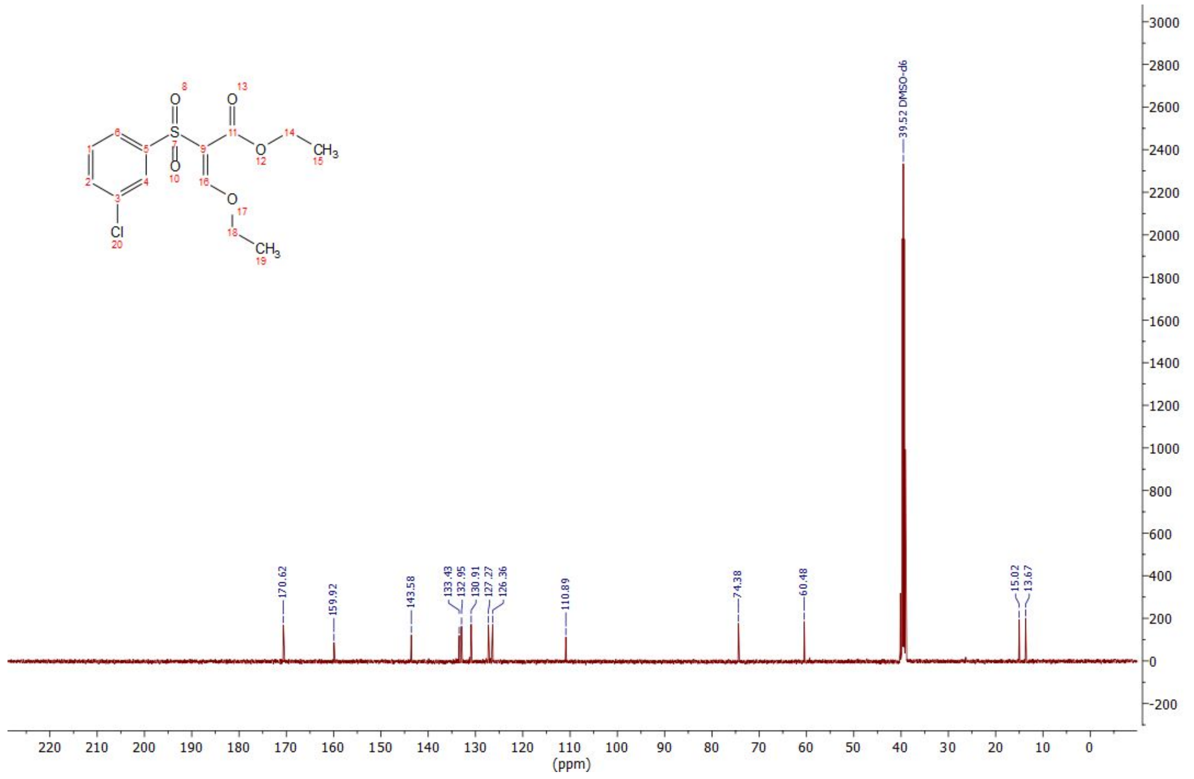

Figure S 56. *^{1}H (400 MHz) and ^{13}C{1H} (101 MHz) NMR-Spectra in DMSO-d_6 of **9f**.*

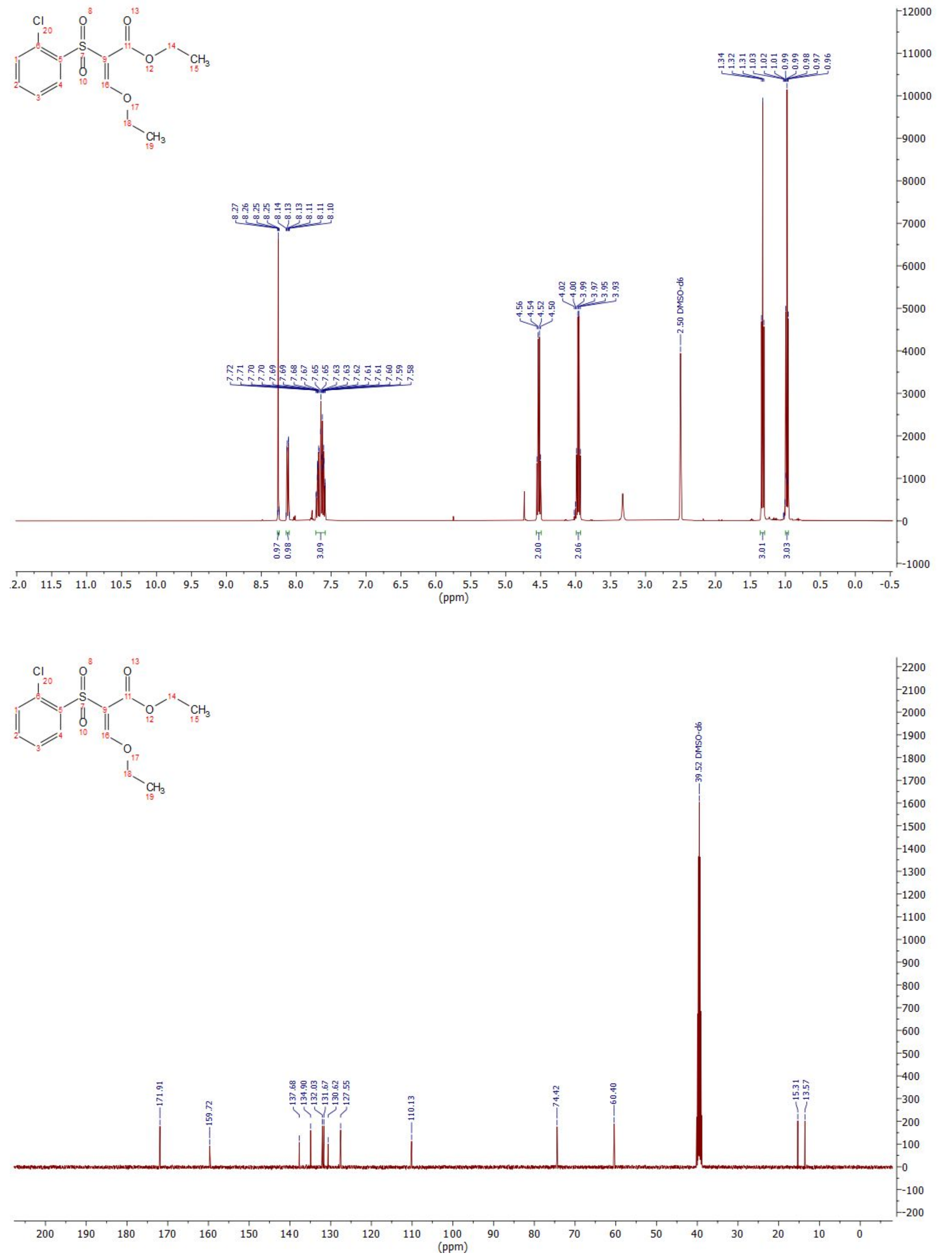

Figure S 57. *^{1}H (400 MHz) and $^{13}C\{1H\}$ (101 MHz) NMR-Spectra in DMSO-d_6 of **9g**.*

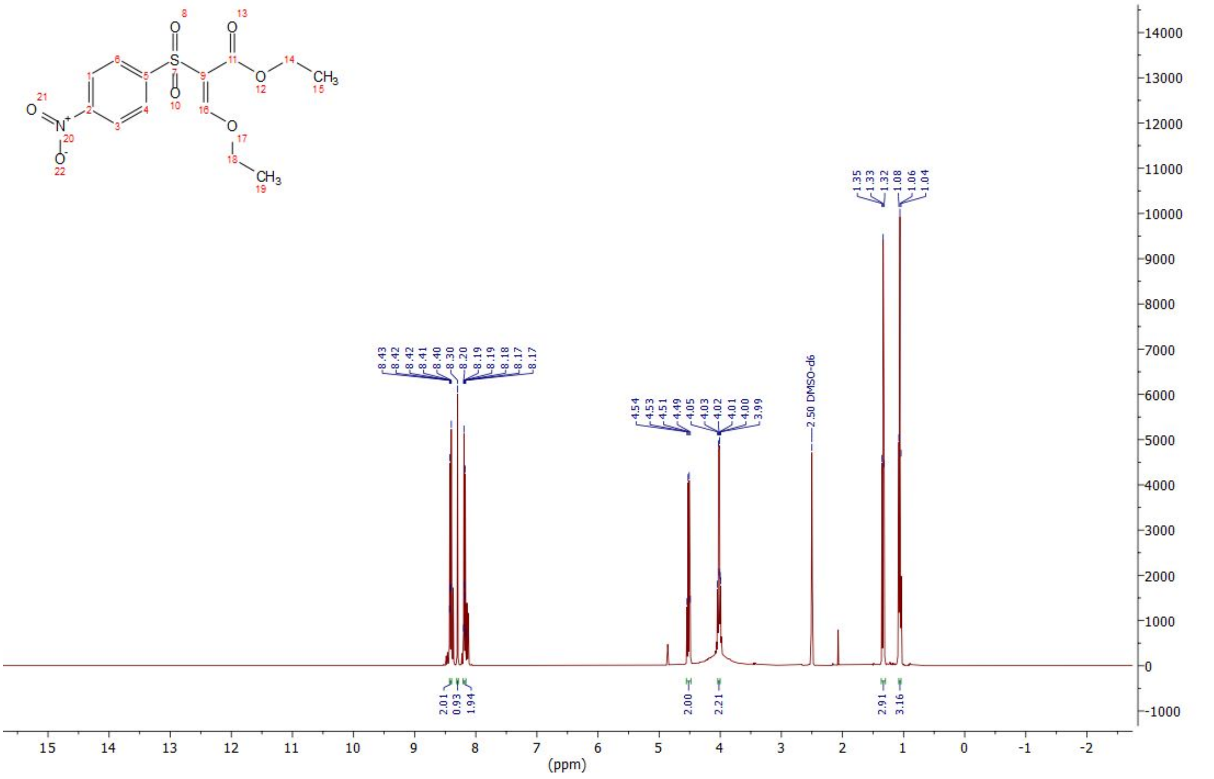

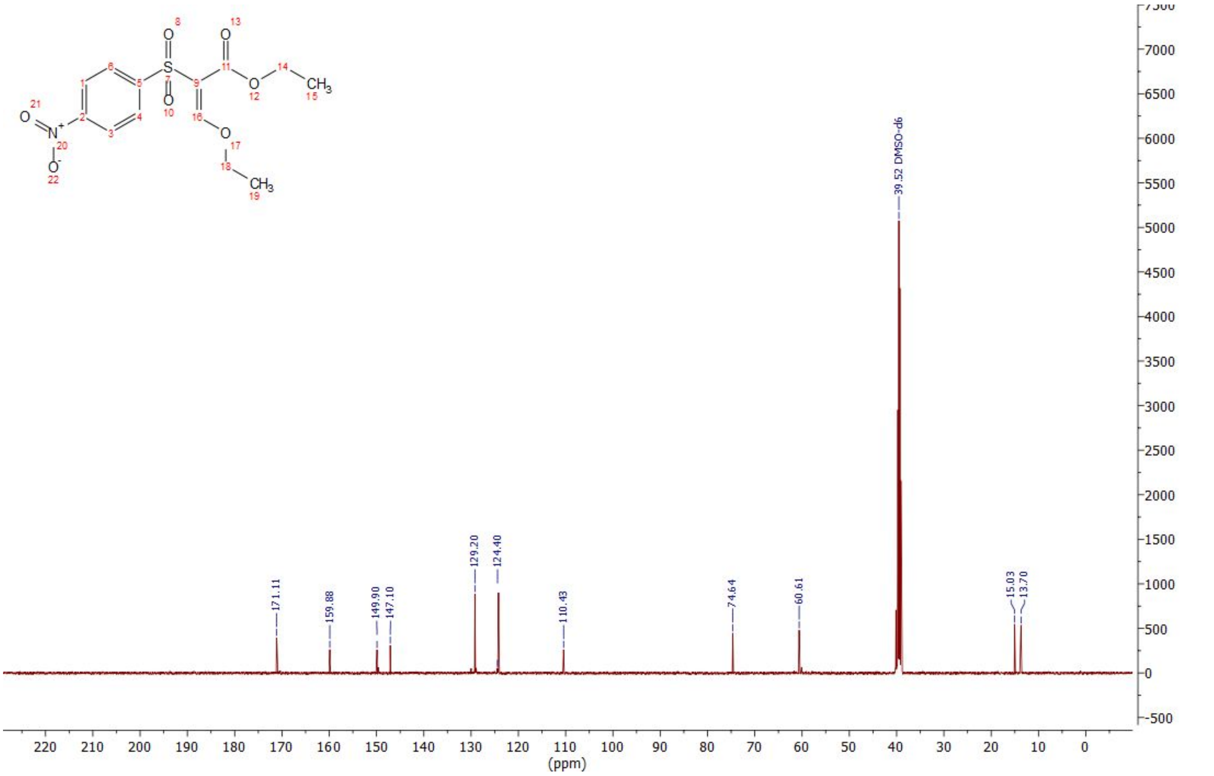

Figure S 58. *^{1}H (400 MHz) and $^{13}C\{1H\}$ (101 MHz) NMR-Spectra in DMSO-d_6 of* ***9h****.*

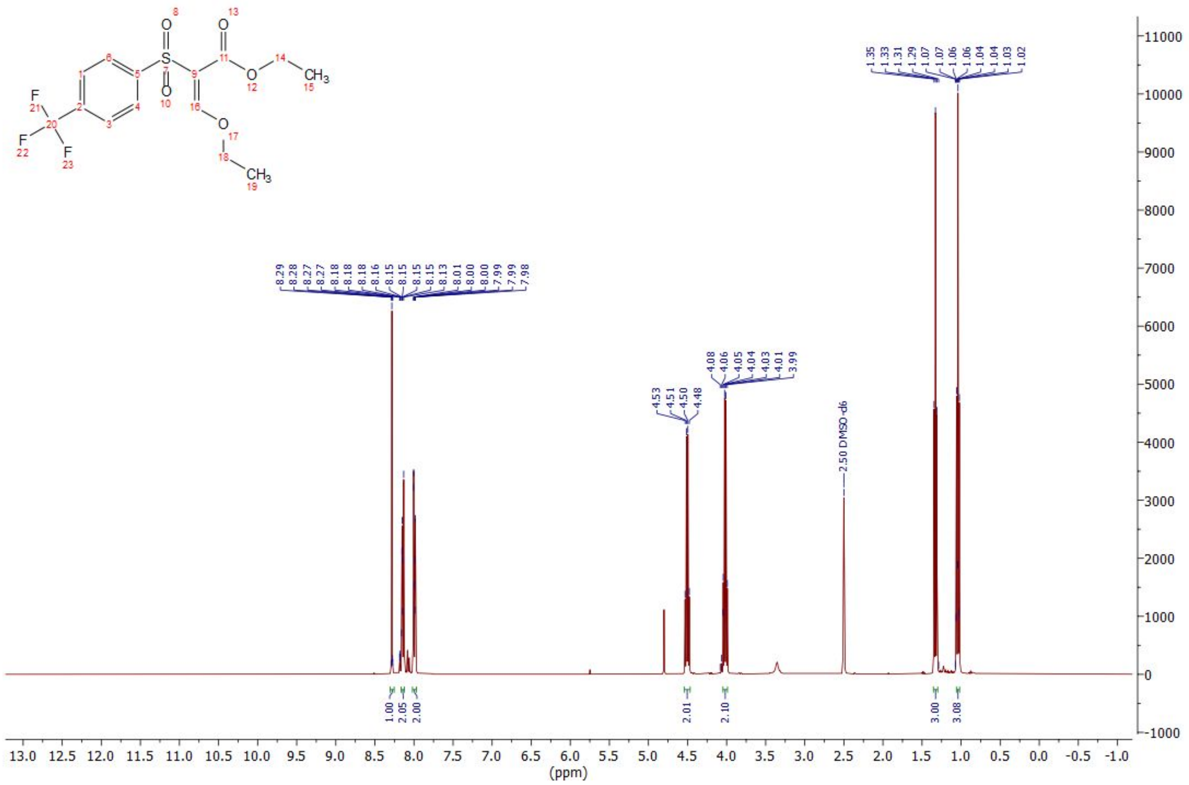

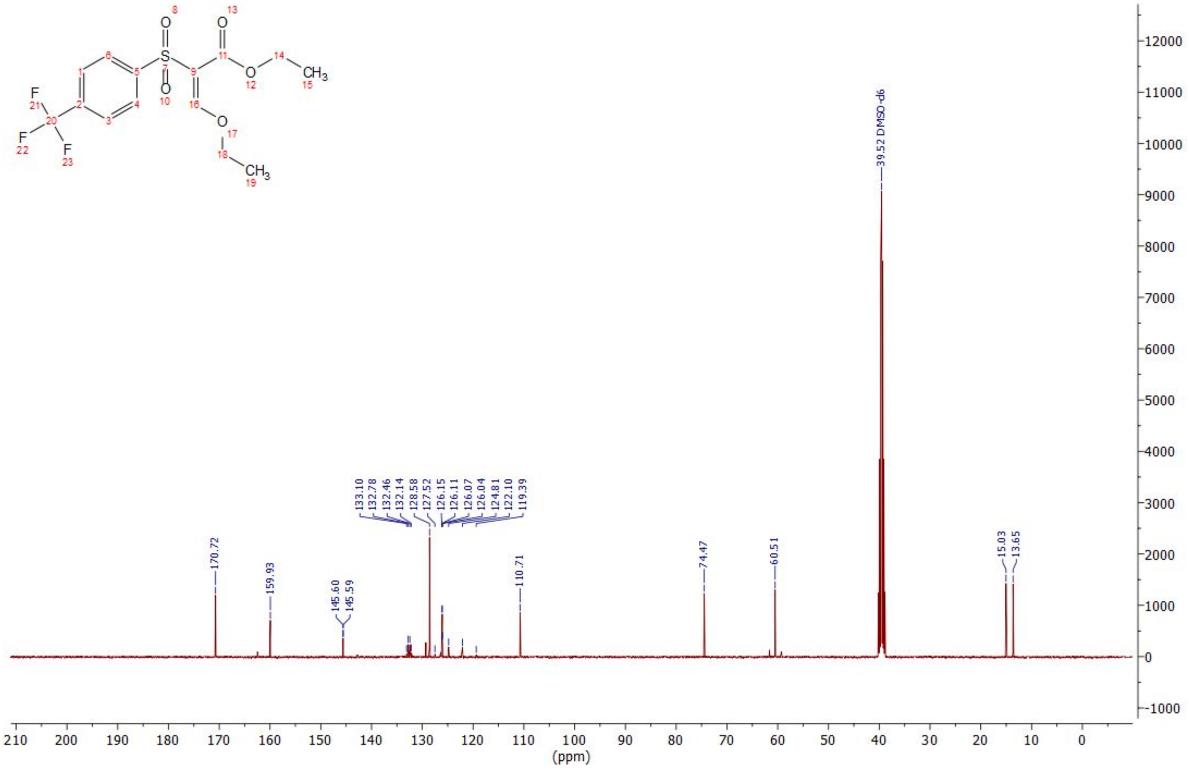

Figure S 59. *^{1}H (400 MHz) and $^{13}C\{1H\}$ (101 MHz) NMR-Spectra in DMSO-d_6 of **9i**.*

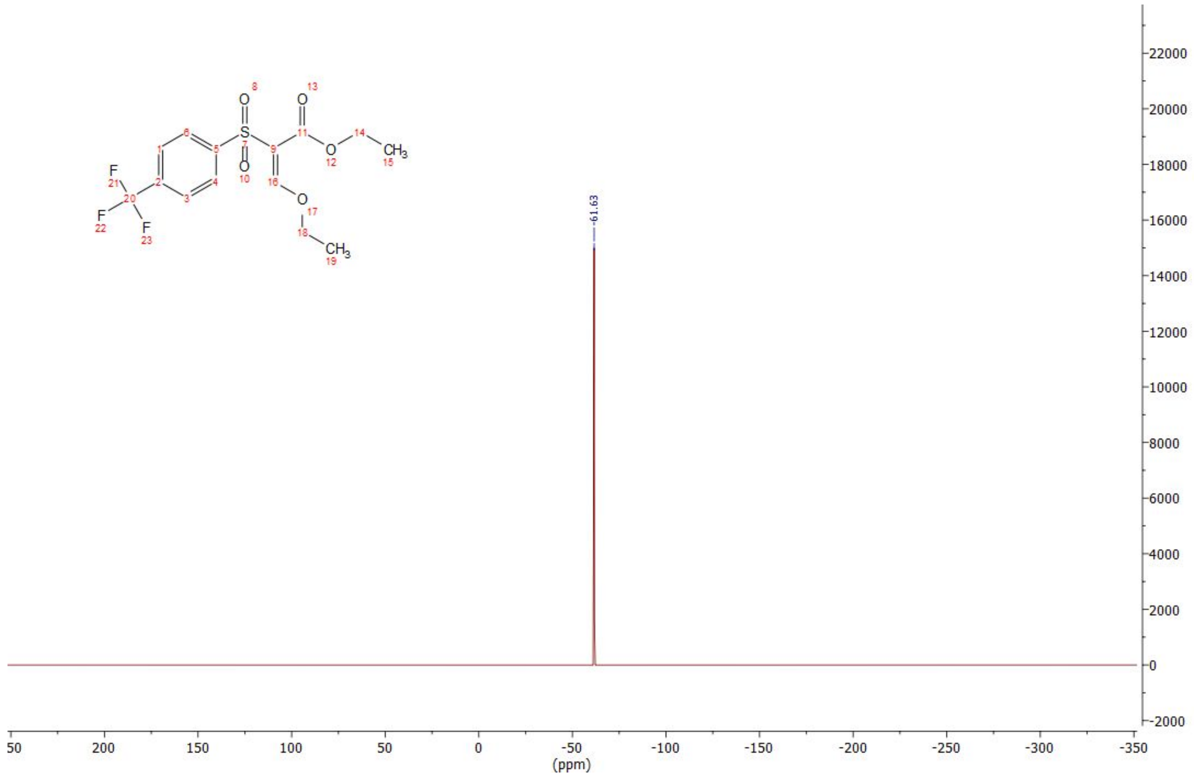

Figure S 60. *^{19}F (376 MHz) NMR-Spectra in DMSO-d_6 of **9i**.*

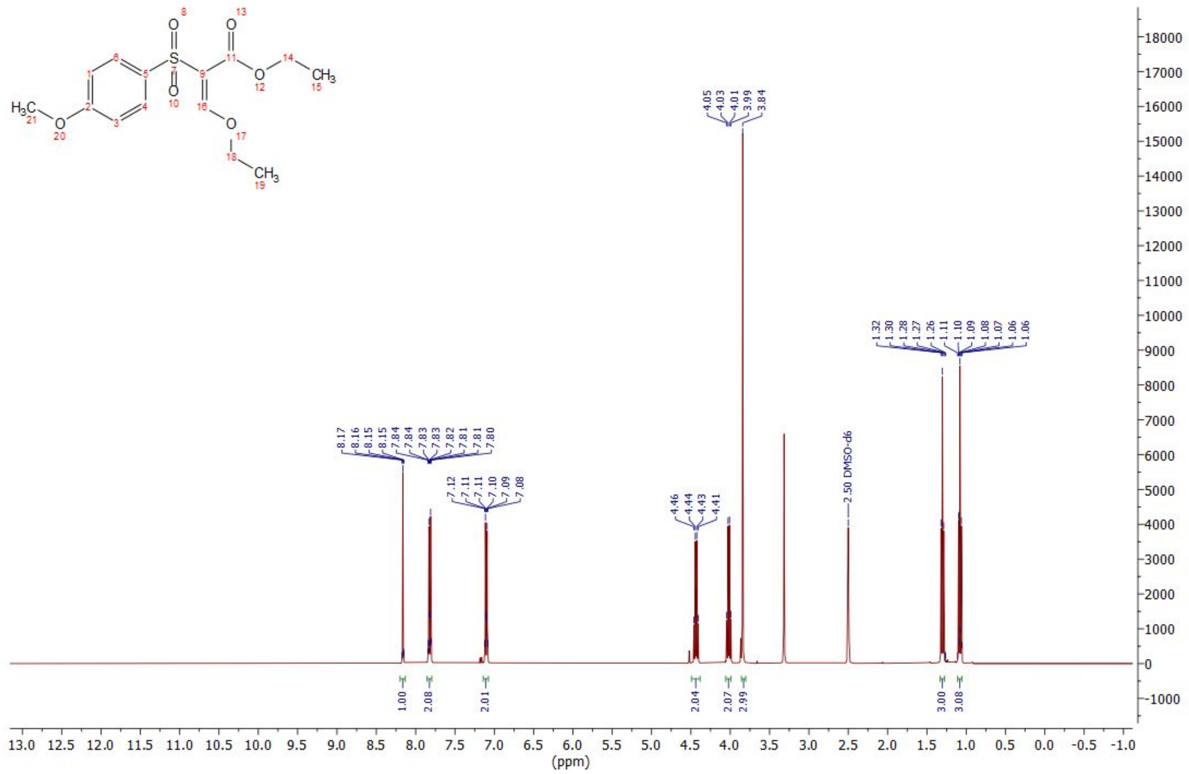

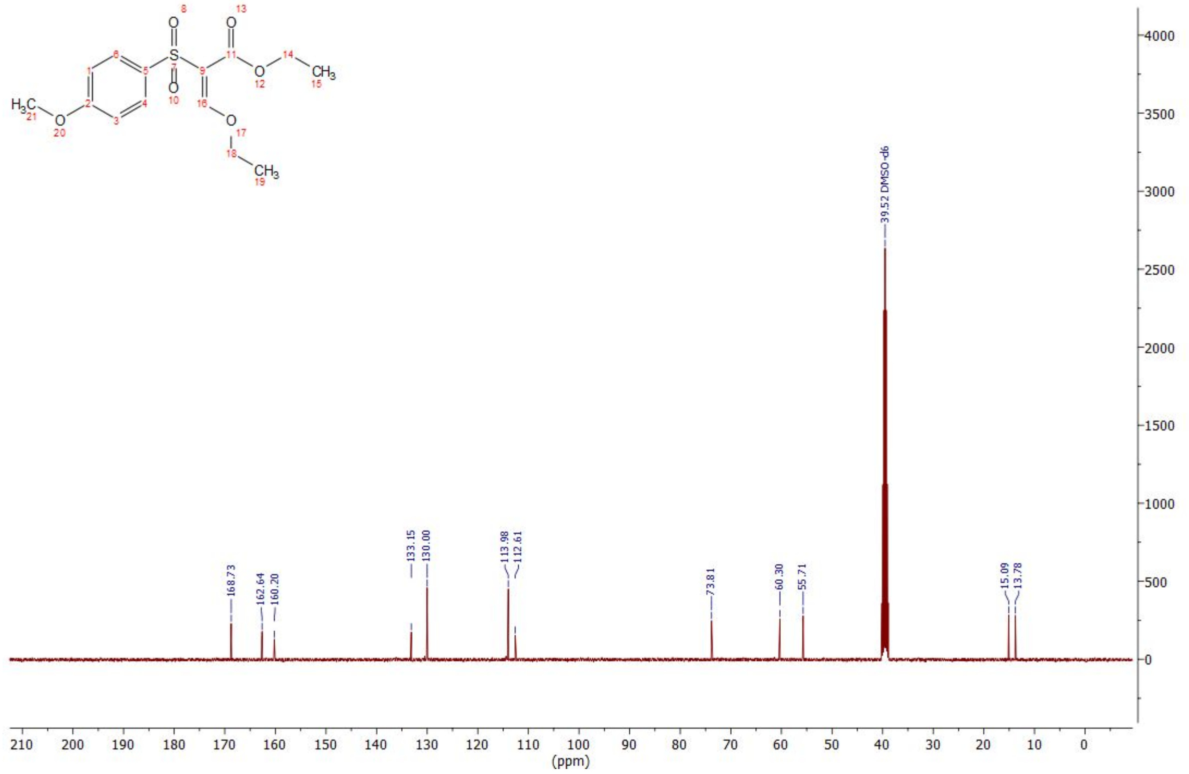

Figure S 61. *^{1}H (400 MHz) and $^{13}C\{1H\}$ (101 MHz) NMR-Spectra in DMSO-d_6 of* ***9j****.*

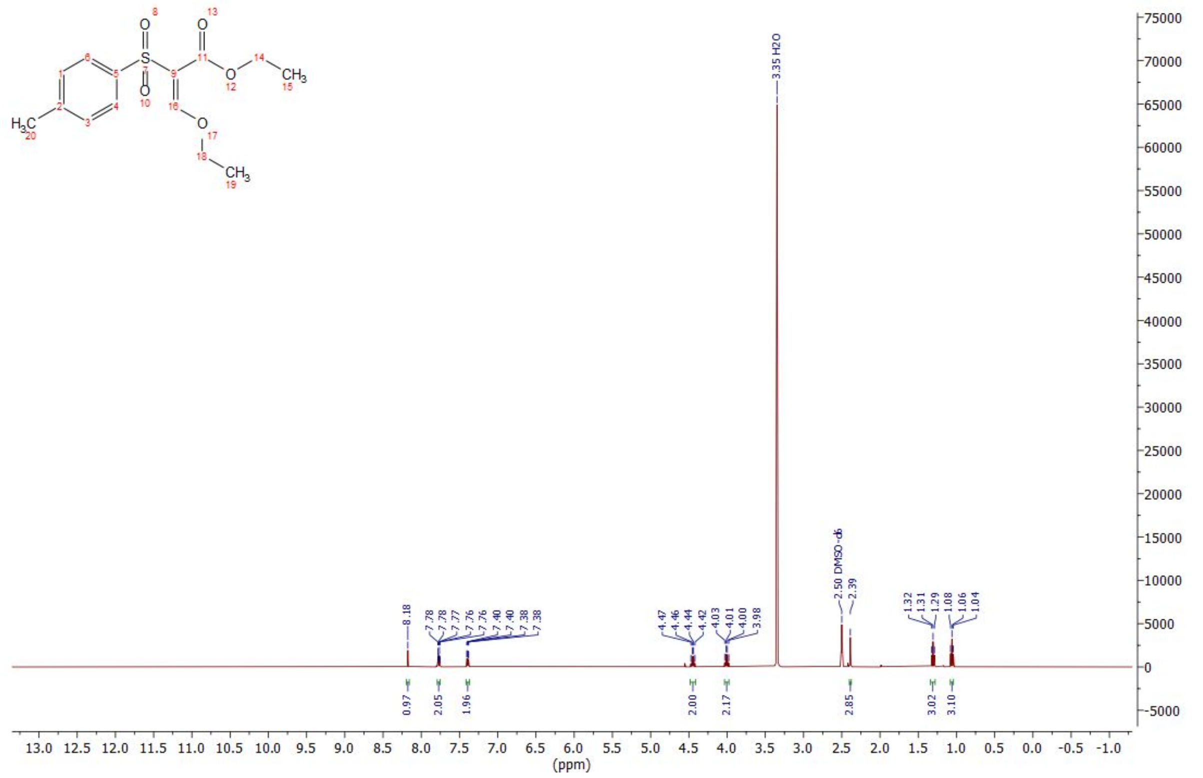

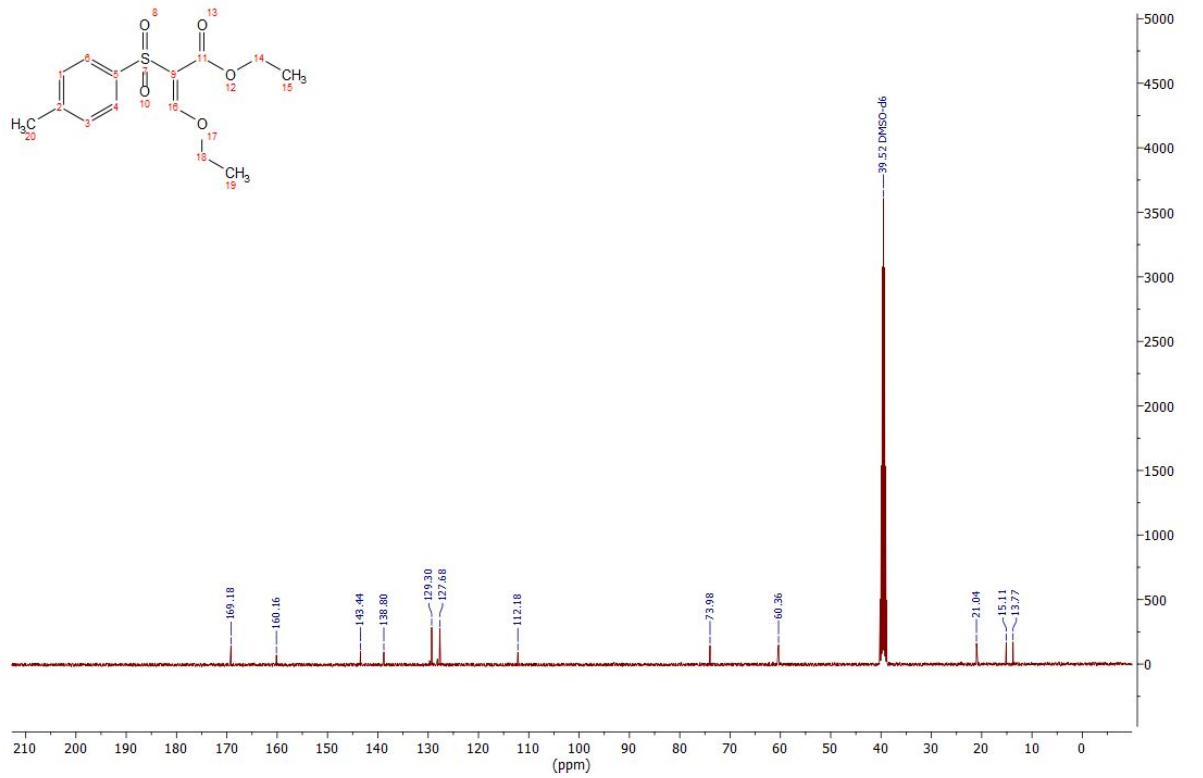

Figure S 62. *^{1}H (400 MHz) and $^{13}C\{1H\}$ (101 MHz) NMR-Spectra in DMSO-d_6 of **9k**.*

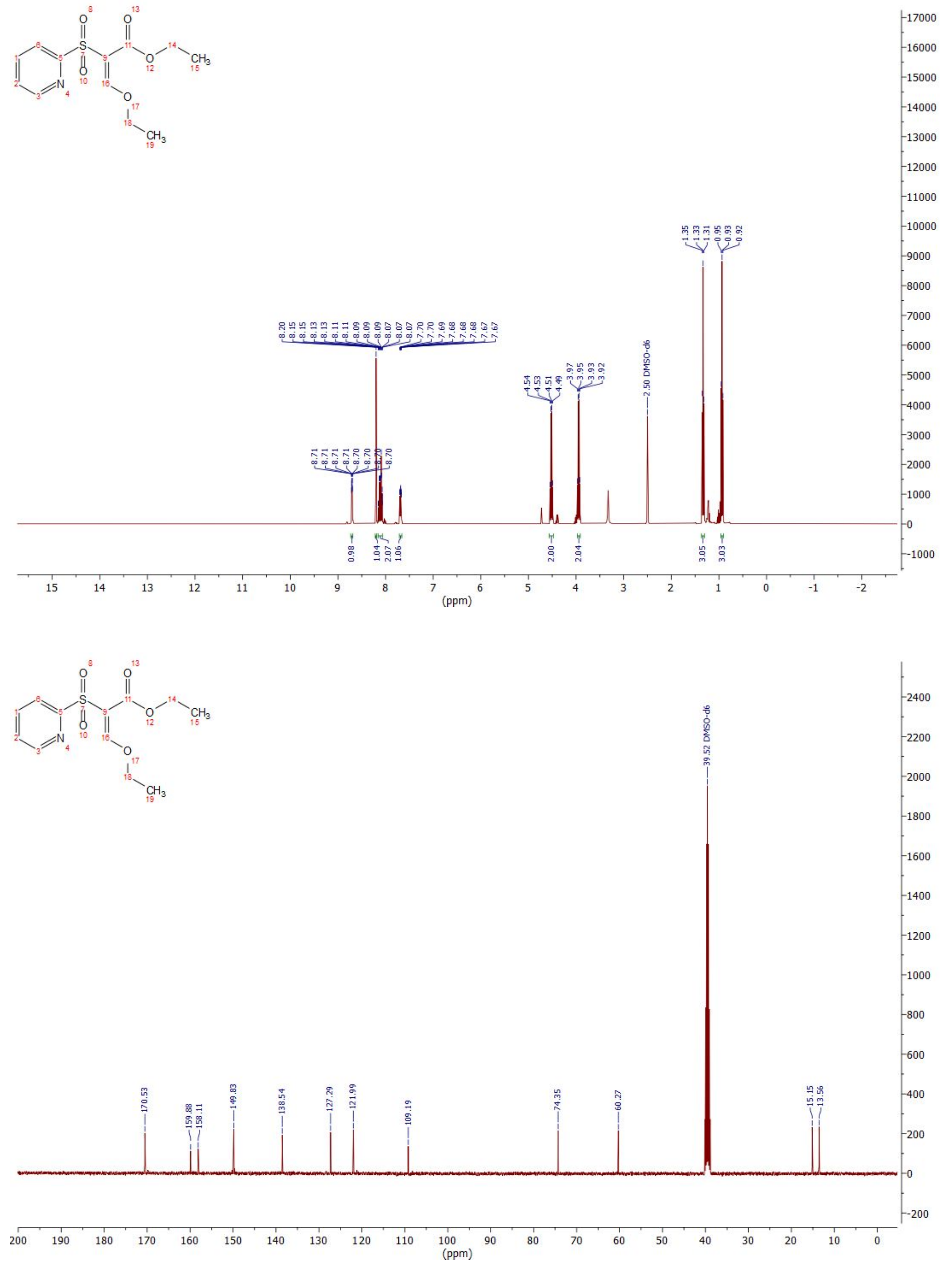

Figure S 63. *^{1}H (400 MHz) and $^{13}C\{1H\}$ (101 MHz) NMR-Spectra in DMSO-d_6 of* ***9n****.*

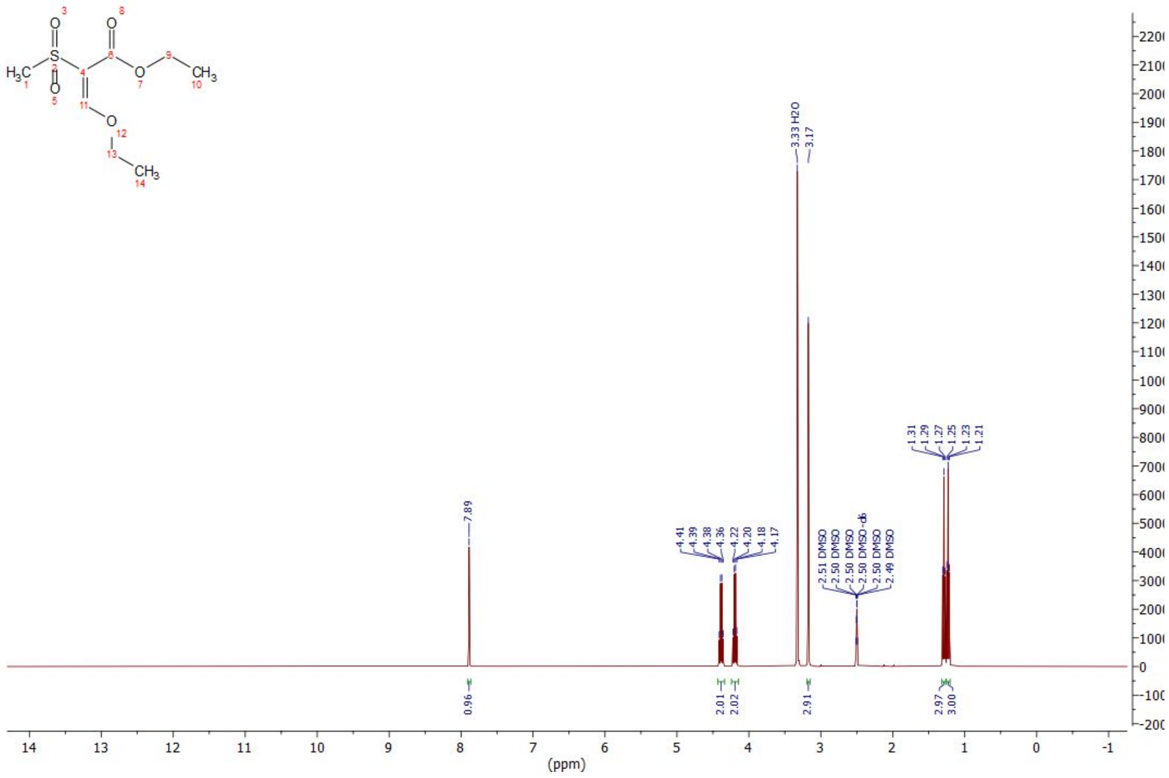

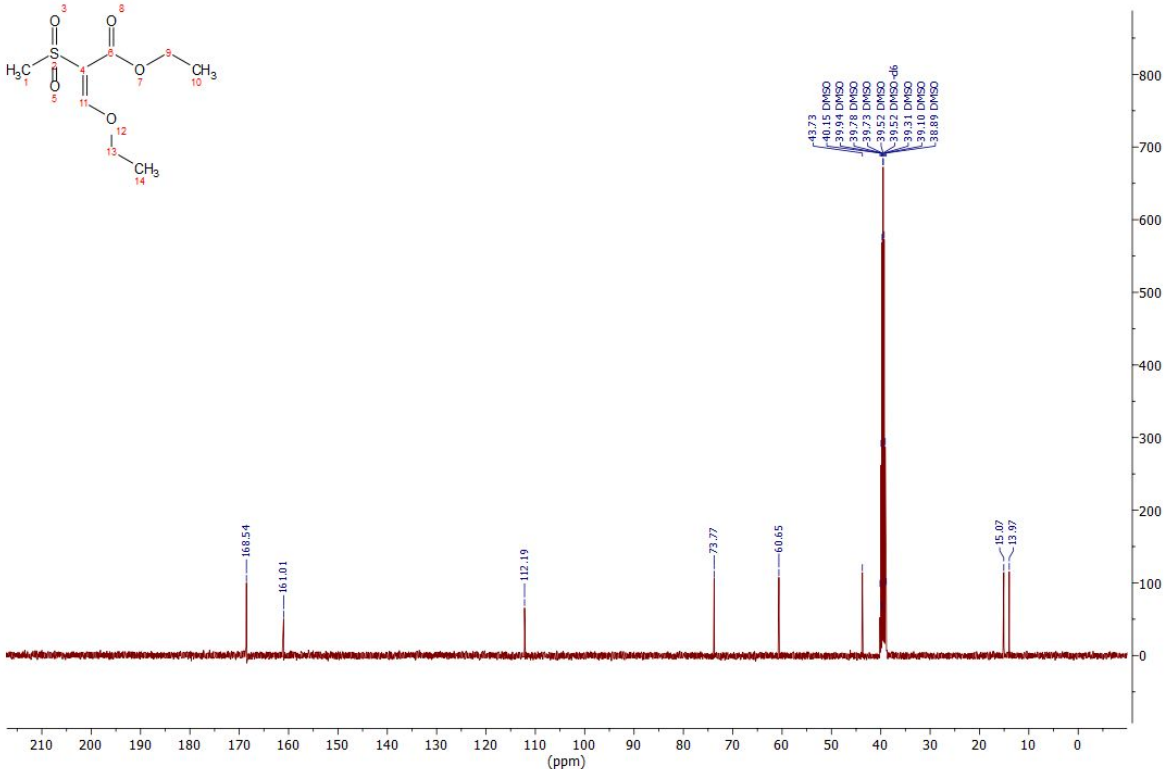

Figure S 64. *^{1}H (400 MHz) and $^{13}C\{^{1}H\}$ (101 MHz) NMR-Spectra in DMSO-d_6 of* ***9r****.*

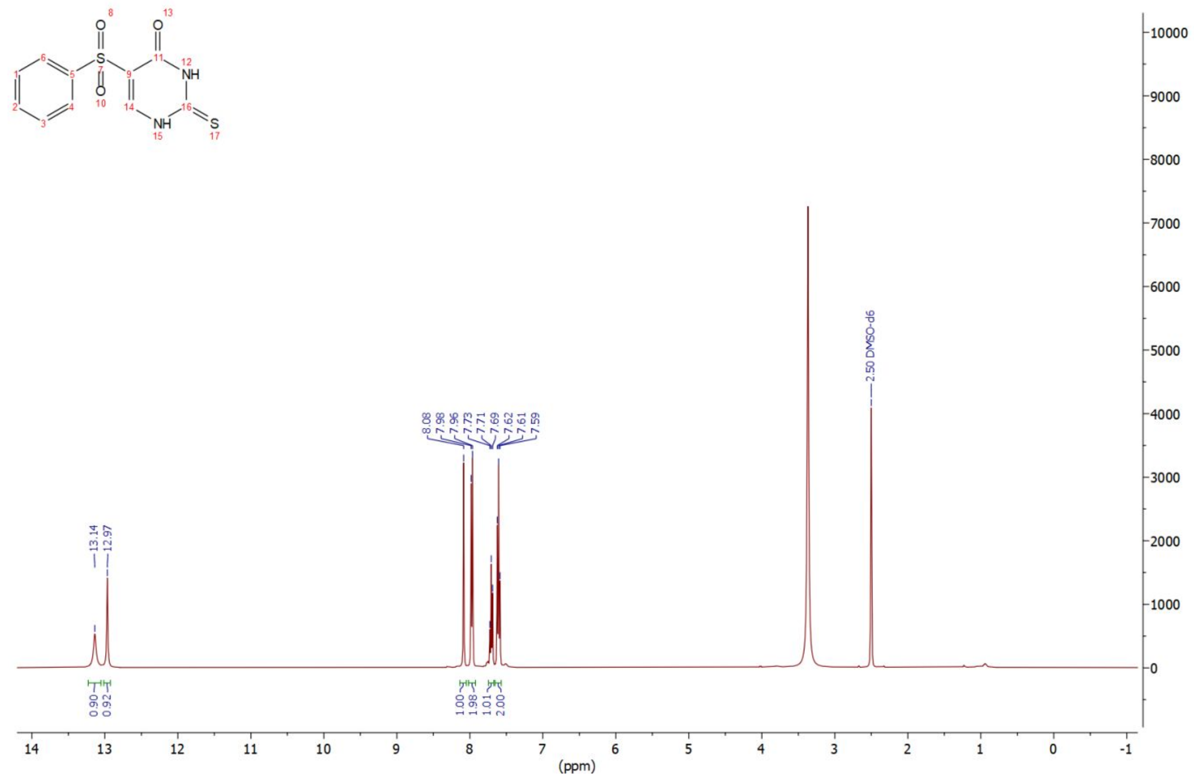

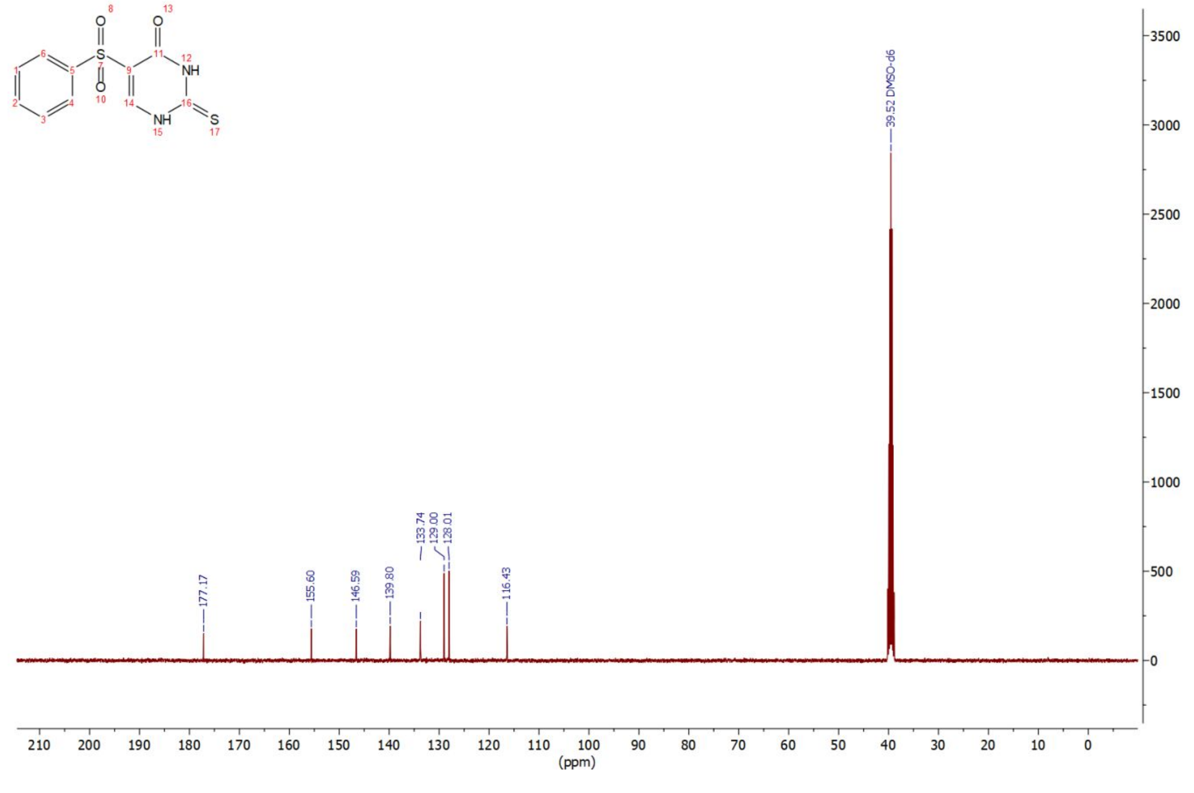

Figure S 65. *^{1}H (400 MHz) and ^{13}C{1H} (101 MHz) NMR-Spectra in DMSO-d_6 of* ***11a****.*

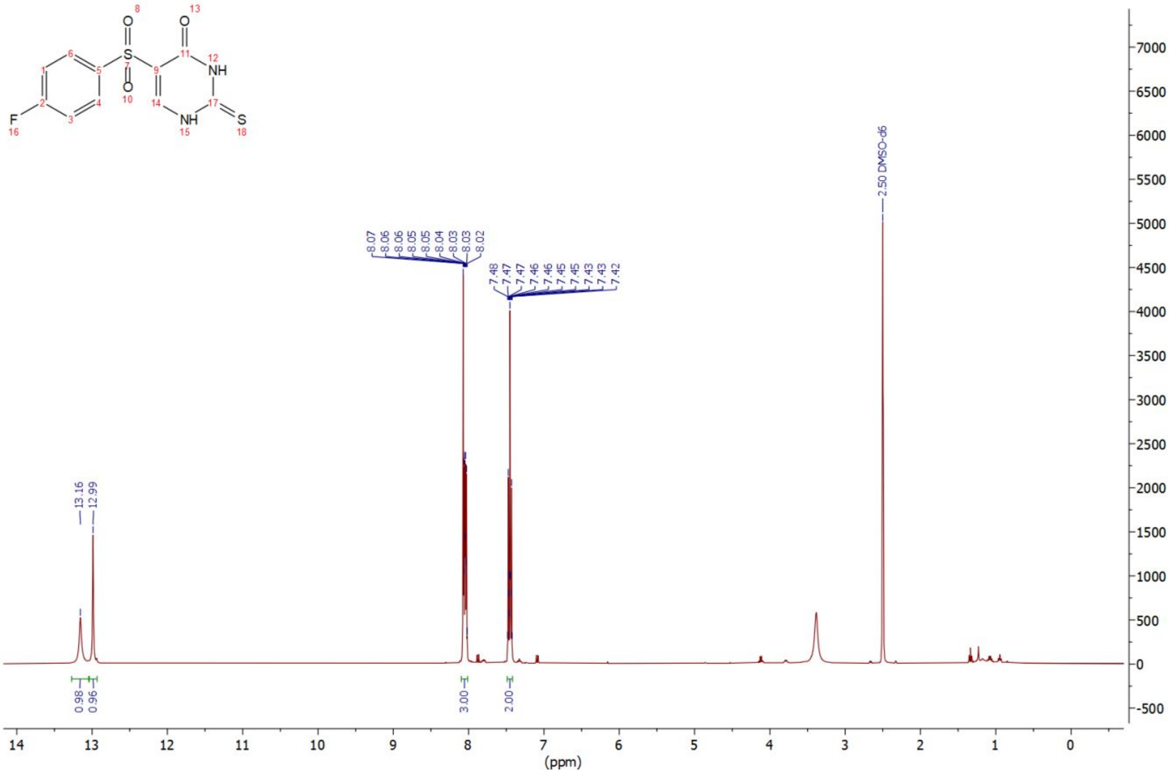

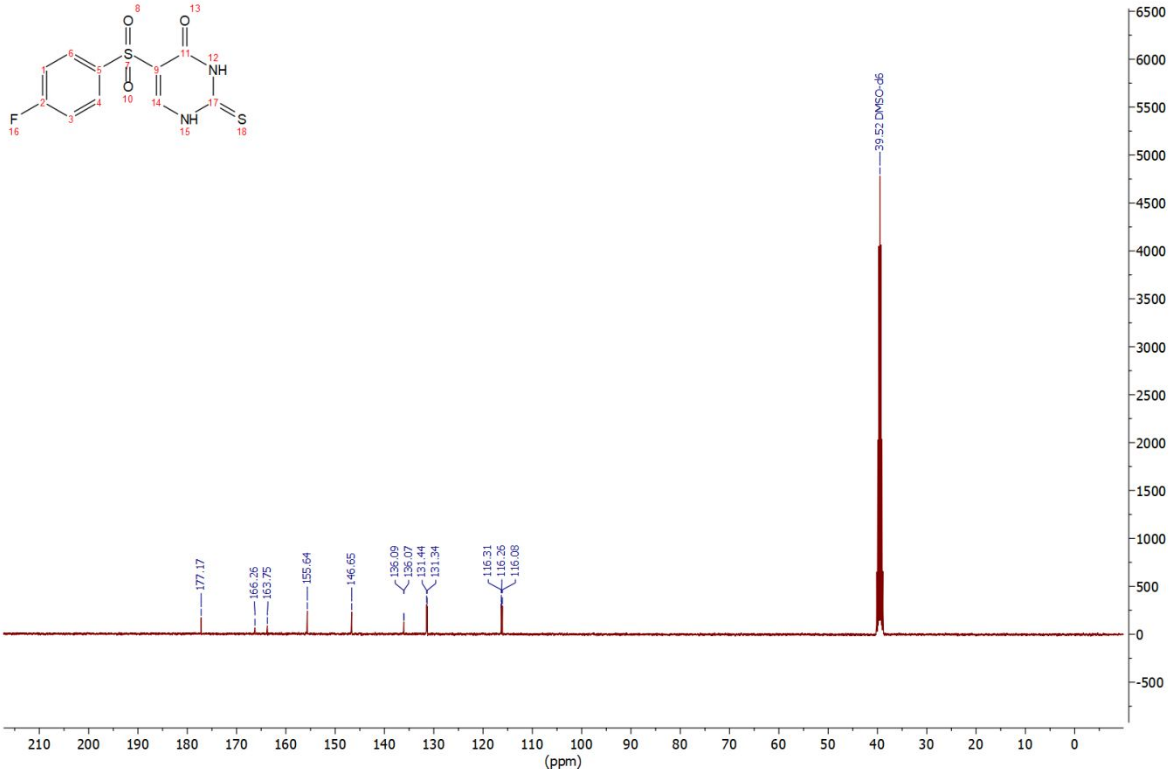

Figure S 66. *^{1}H (400 MHz) and $^{13}C\{1H\}$ (101 MHz) NMR-Spectra in DMSO-d_6 of* ***11b****.*

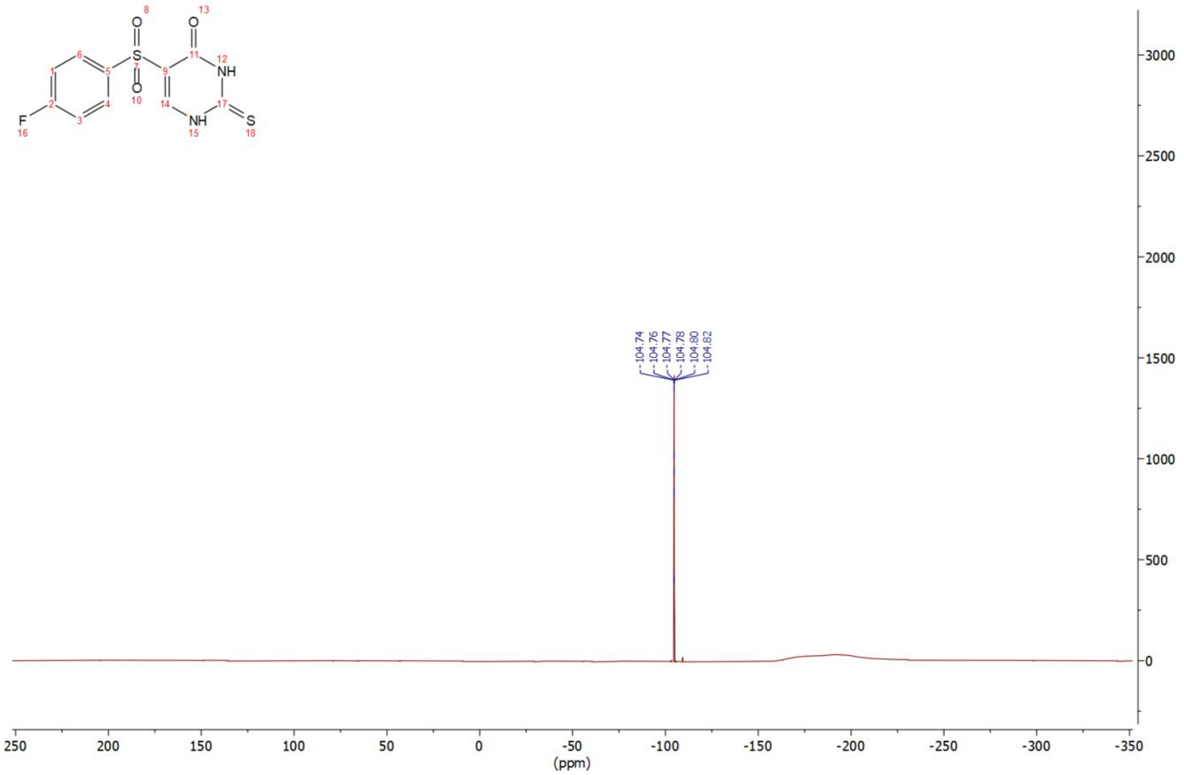

Figure S 67. *^{19}F (376 MHz) NMR-Spectra in DMSO-d_6 of **11b**.*

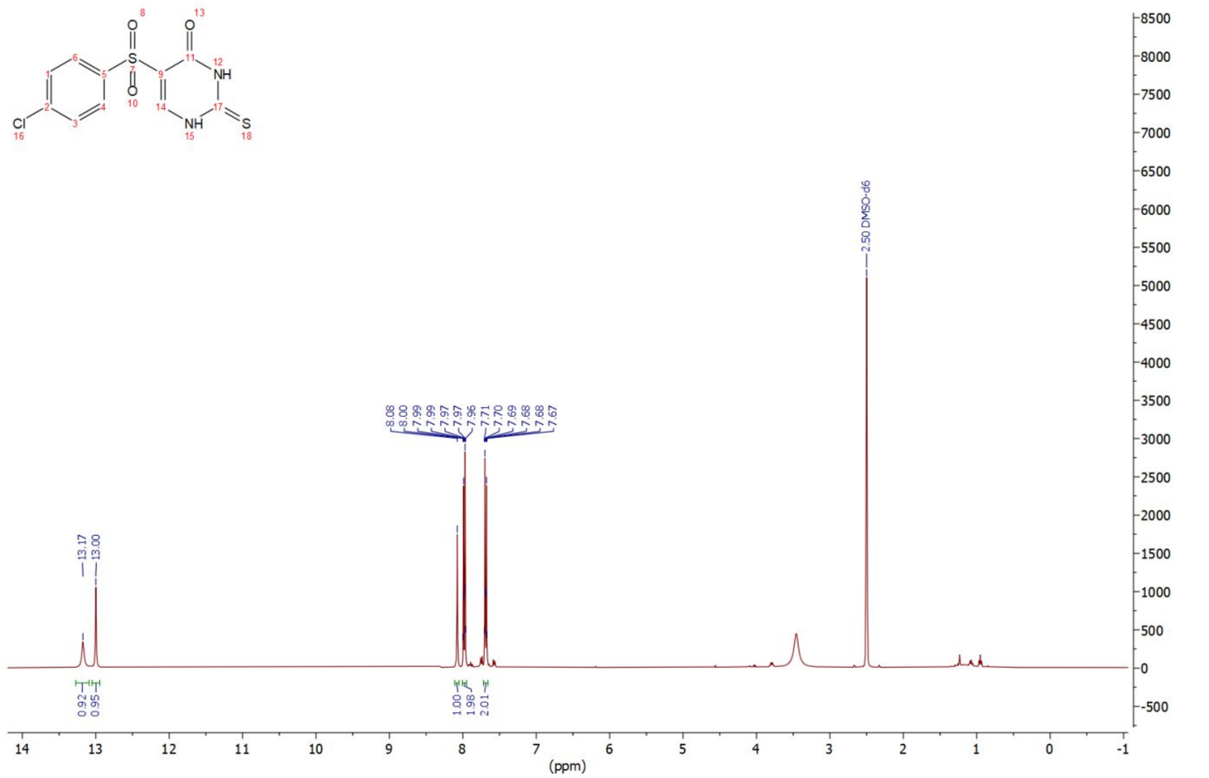

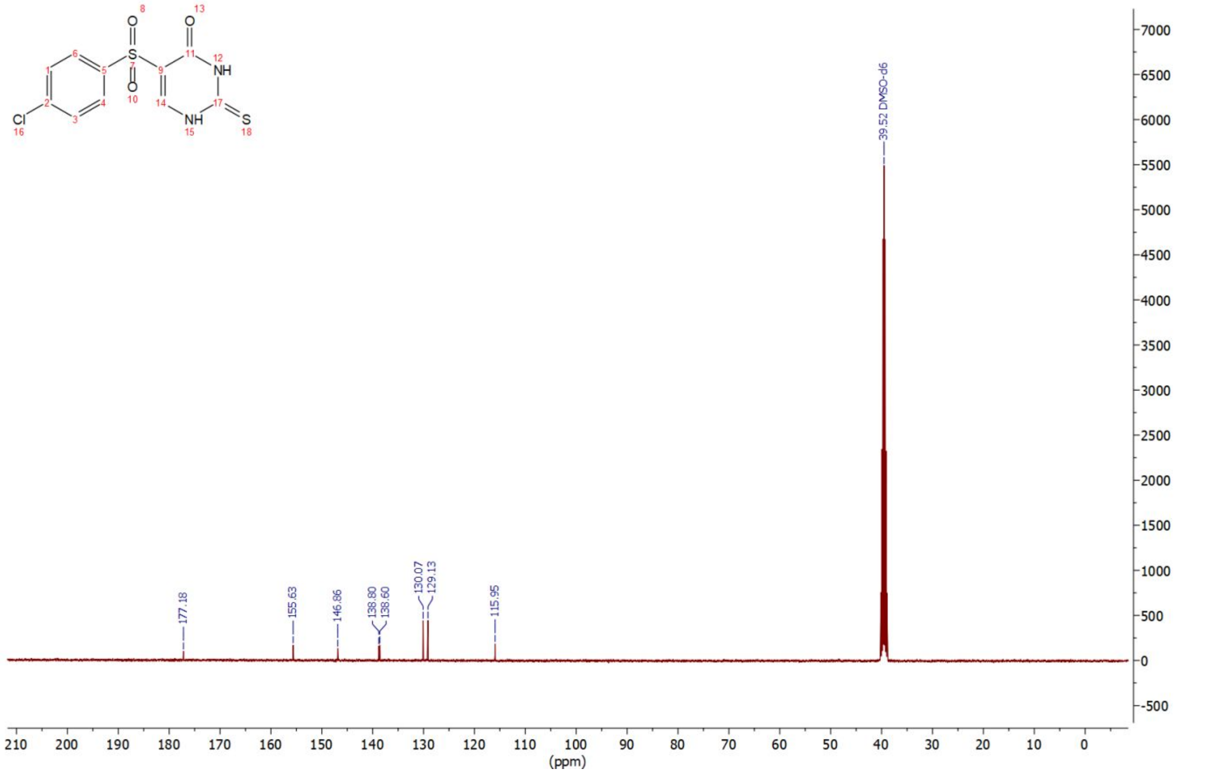

Figure S 68. *^{1}H (400 MHz) and ^{13}C{1H} (101 MHz) NMR-Spectra in DMSO-d_6 of* ***11c****.*

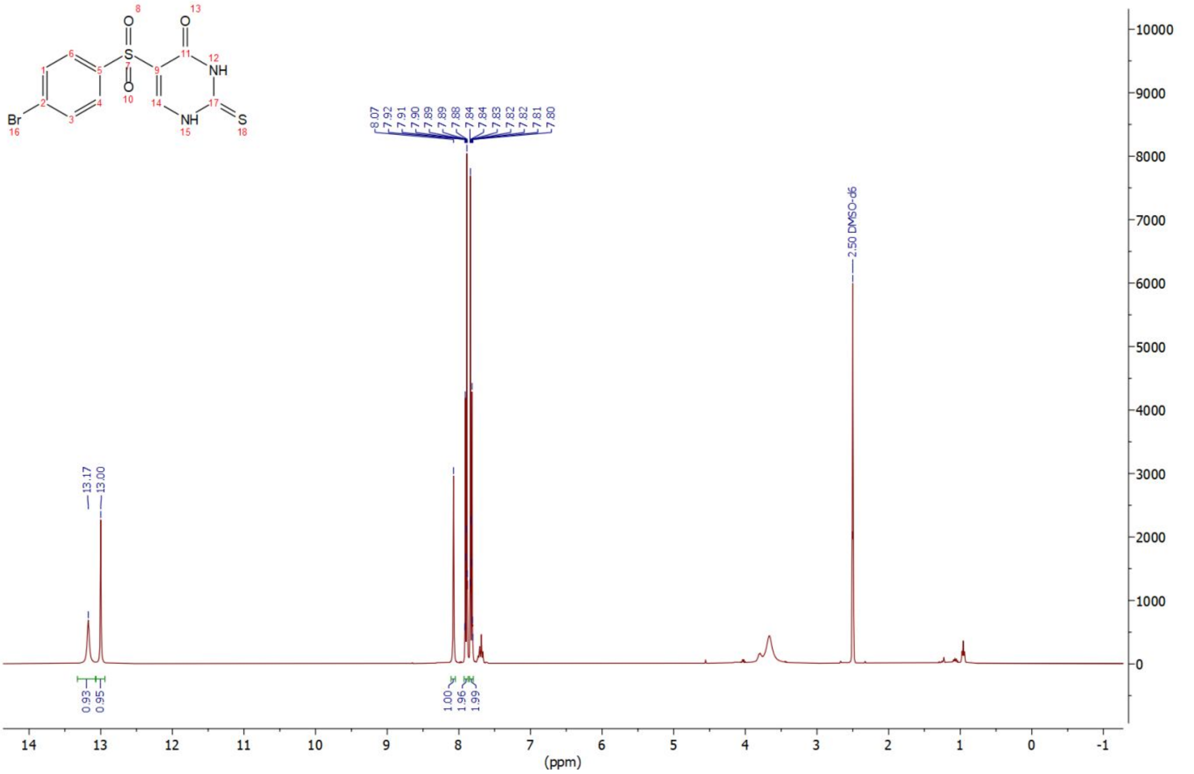

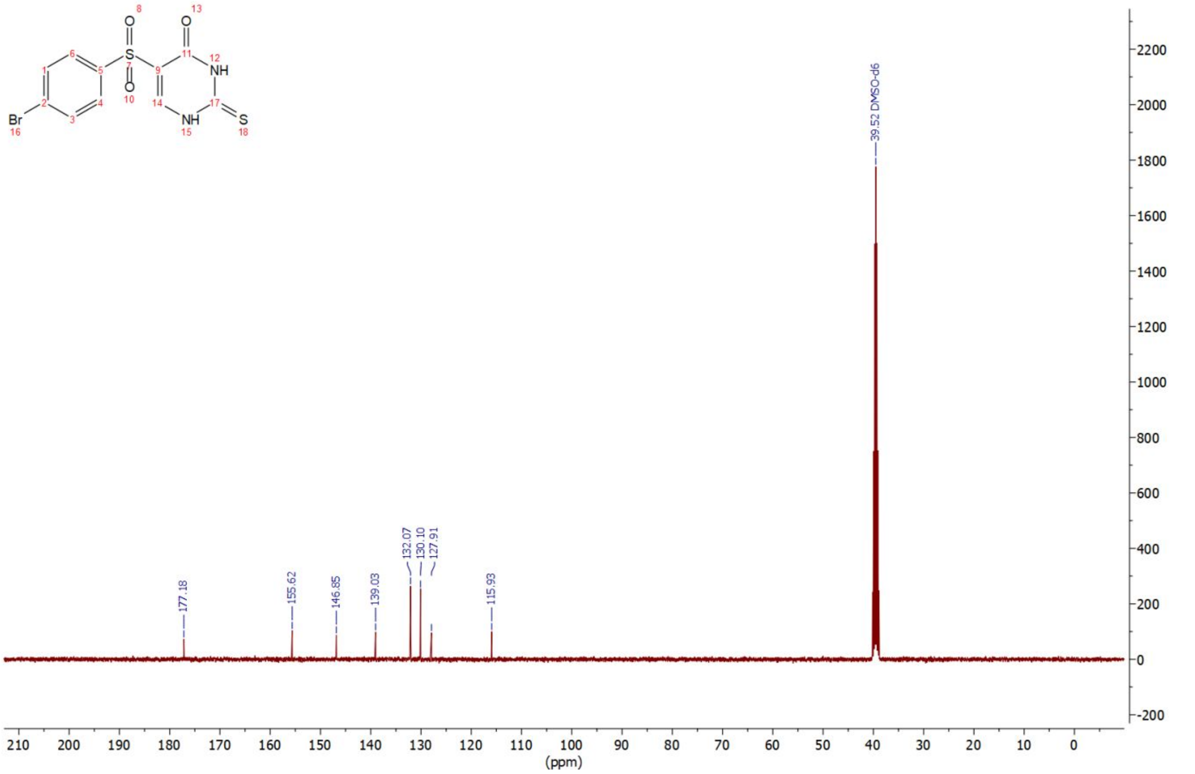

Figure S 69. *^{1}H (400 MHz) nd $^{13}C\{1H\}$ (101 MHz) NMR-Spectra in DMSO-d_6 of **11d**.*

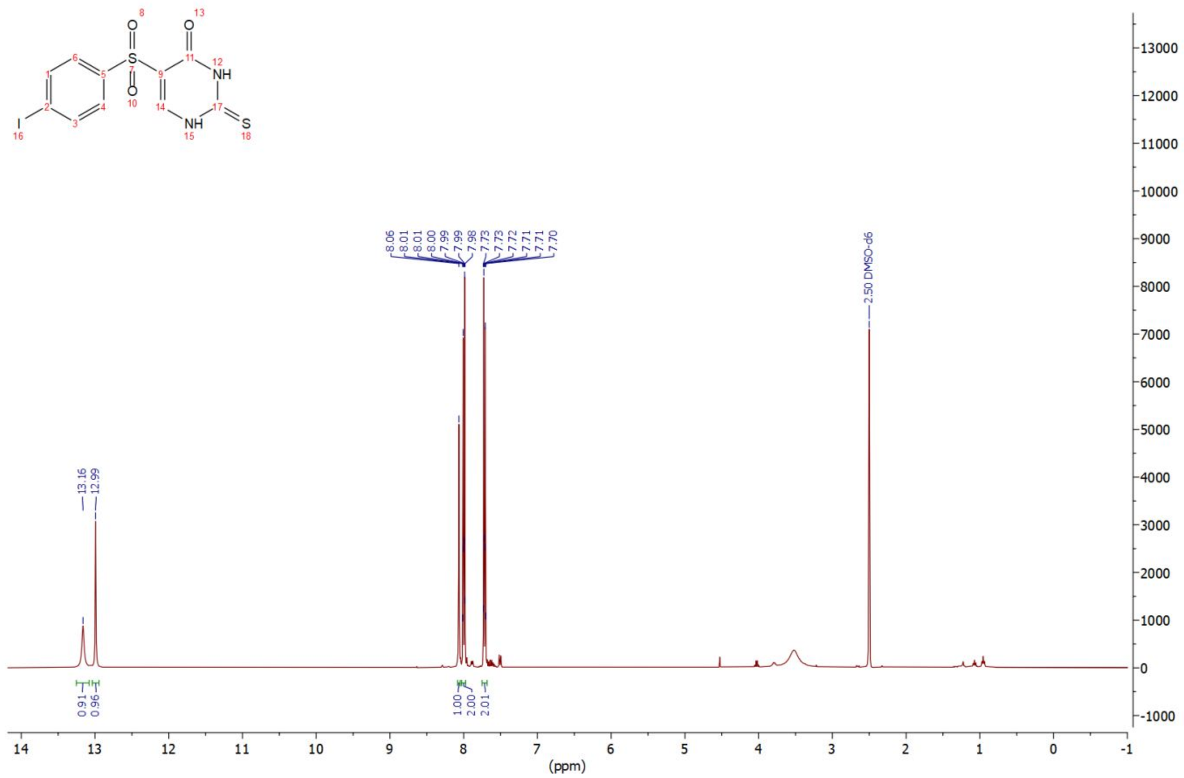

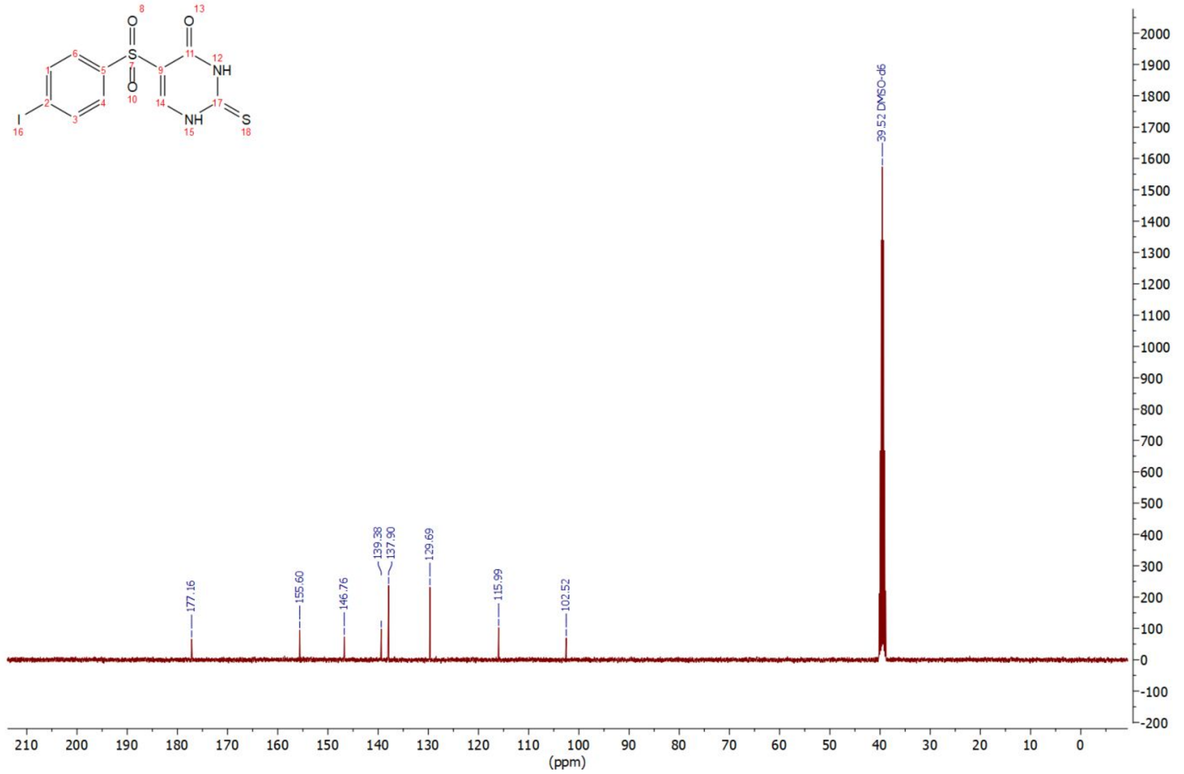

Figure S 70. *^{1}H (400 MHz) and ^{13}C{1H} (101 MHz) NMR-Spectra in DMSO-d_6 of* ***11e****.*

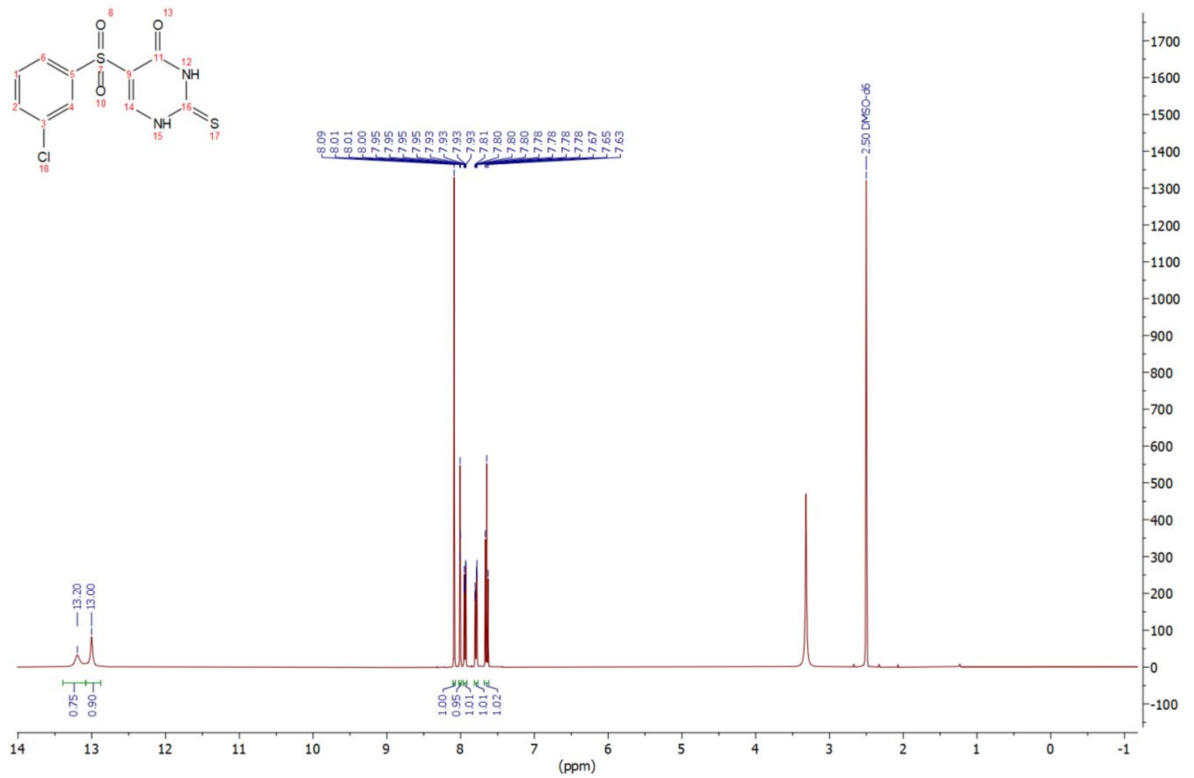

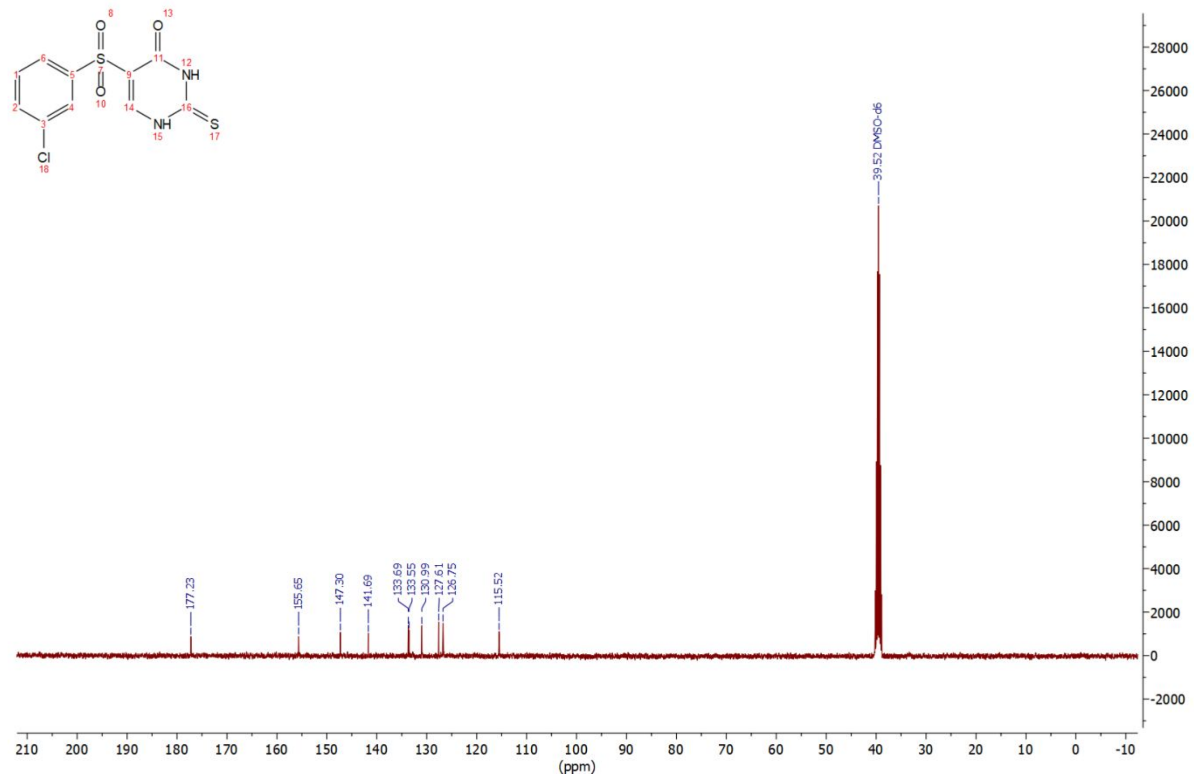

Figure S 71. *^{1}H (400 MHz) and $^{13}C\{1H\}$ (101 MHz) NMR-Spectra in DMSO-d_6 of **11f**.*

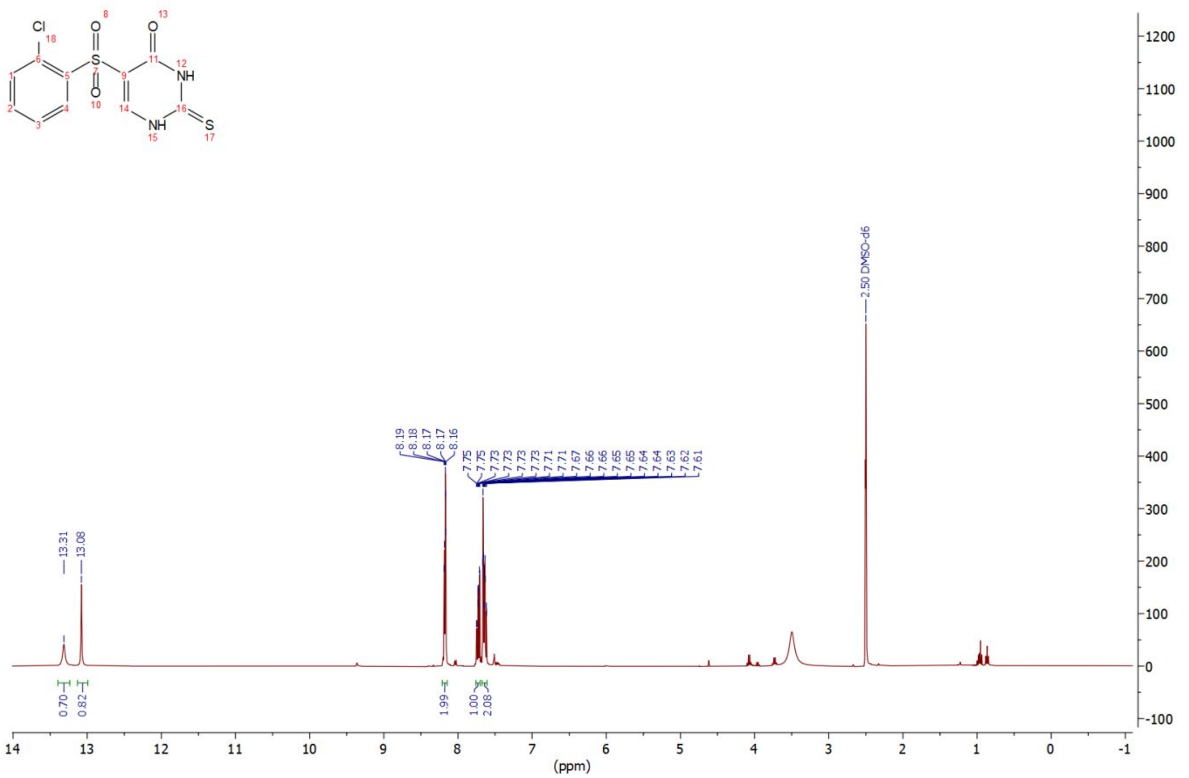

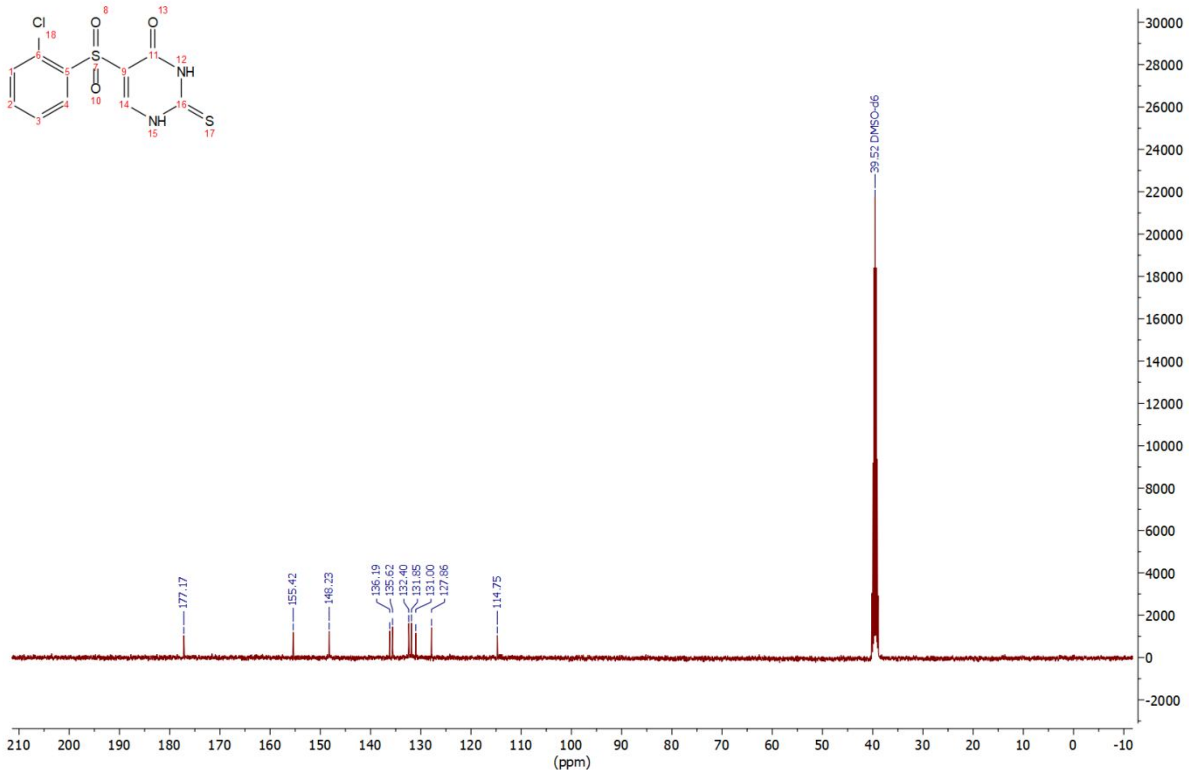

Figure S 72. *^{1}H (400 MHz) and ^{13}C{1H} (101 MHz) NMR-Spectra in DMSO-d$_6$ of* ***11g****.*

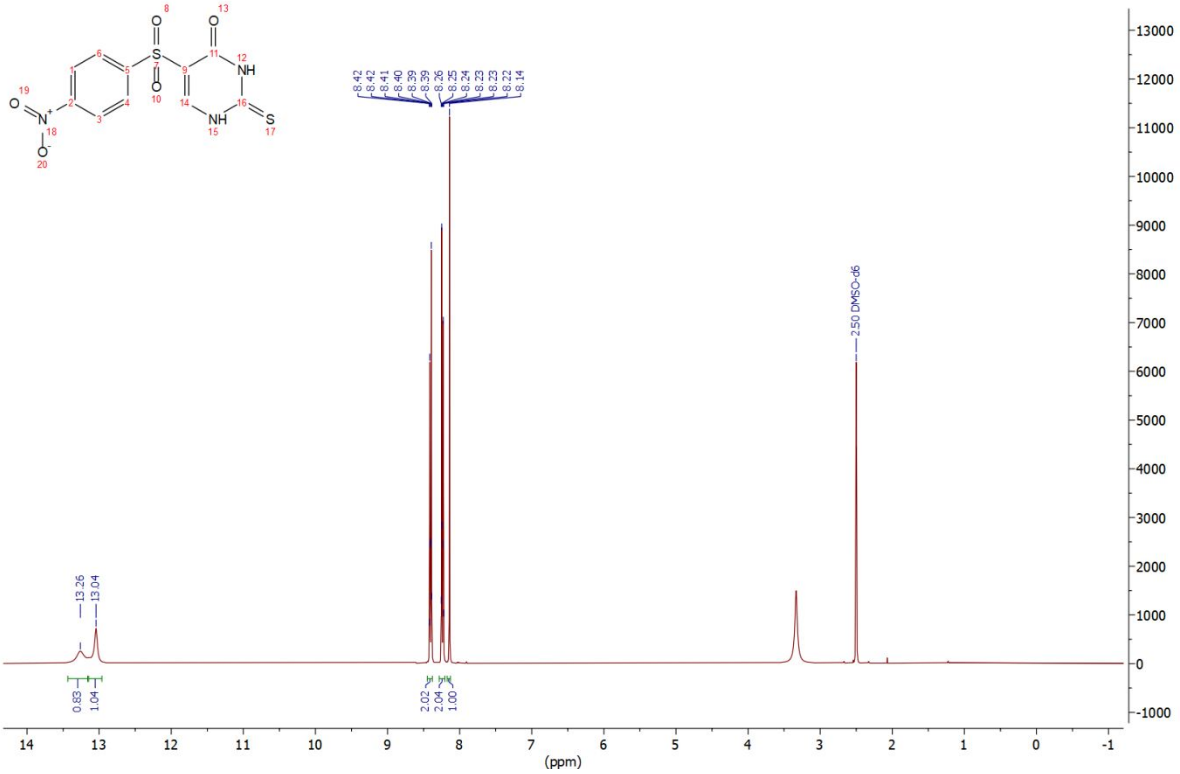

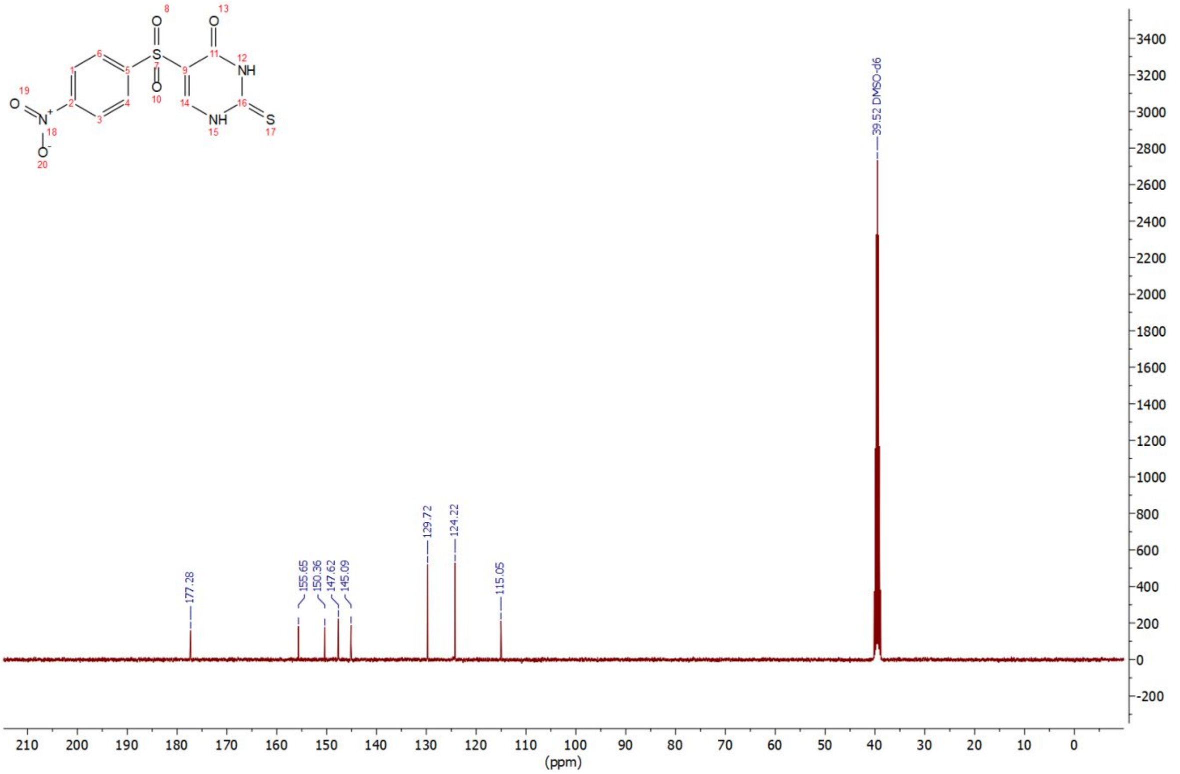

Figure S 73. *^1H (400 MHz) and $^{13}C\{1H\}$ (101 MHz) NMR-Spectra in DMSO-d_6 of* ***11h****.*

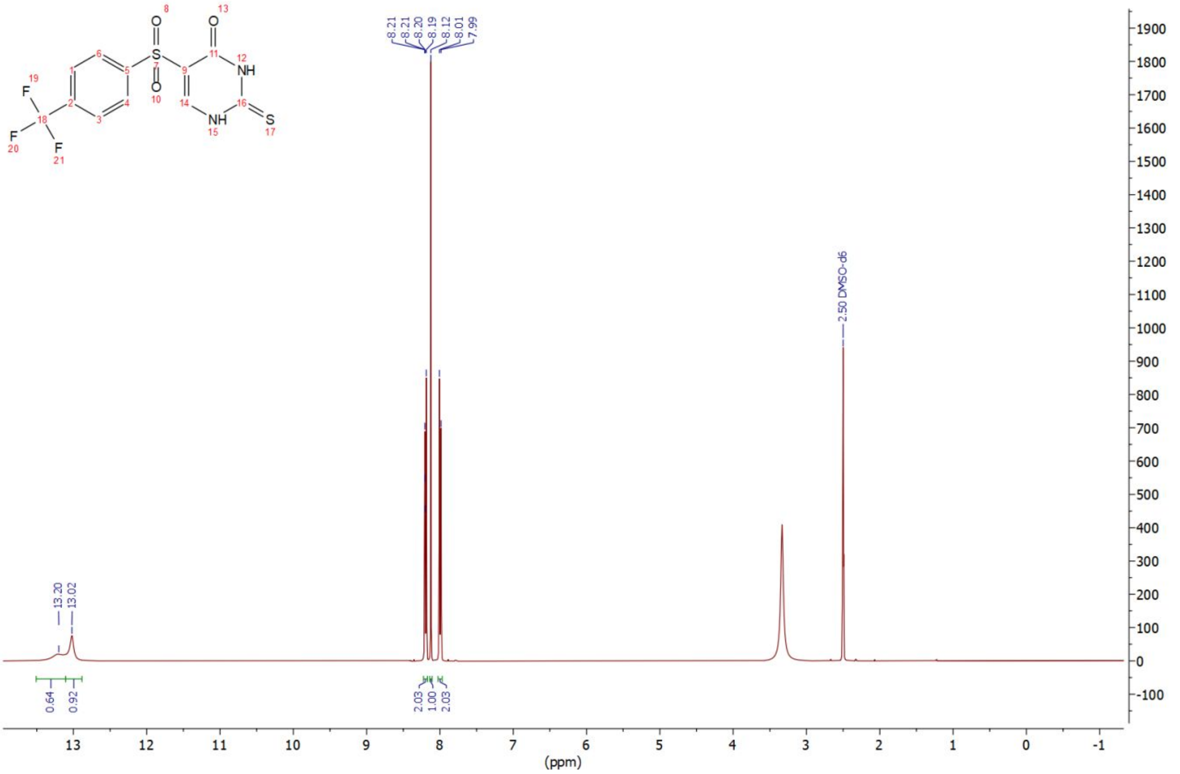

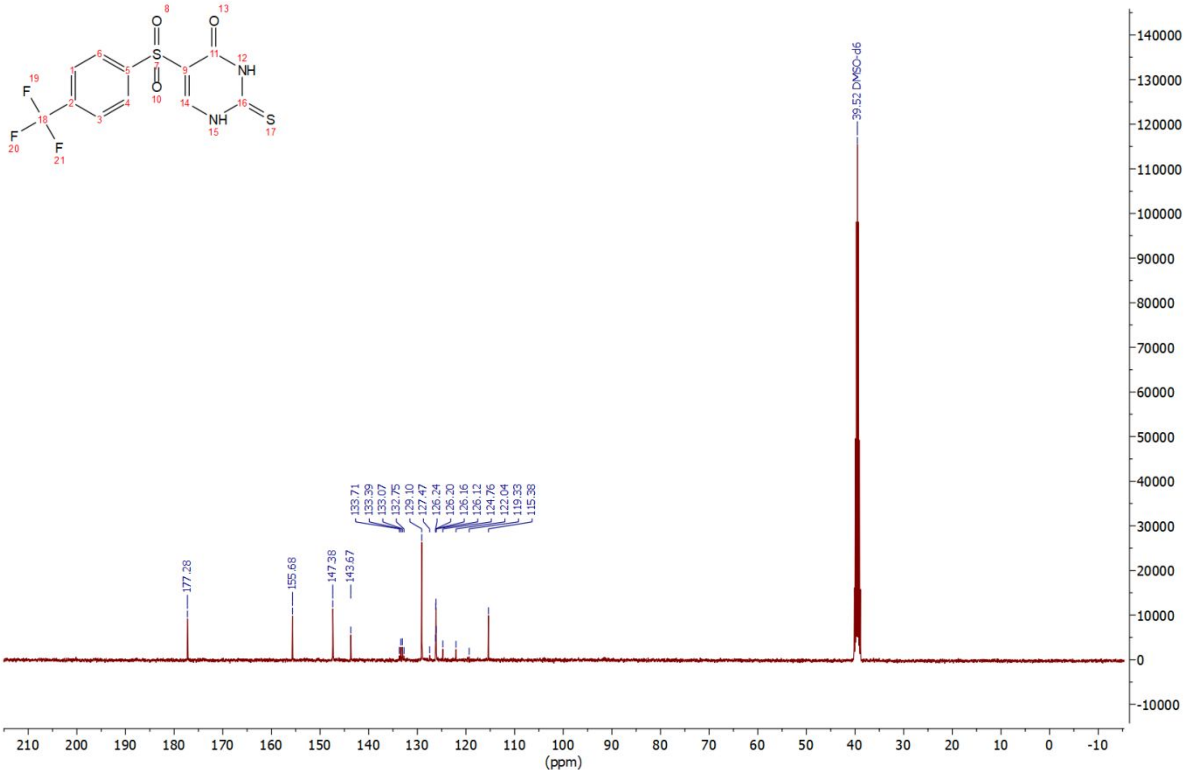

Figure S 74. *^{1}H (400 MHz) and $^{13}C\{1H\}$ (101 MHz) NMR-Spectra in DMSO-d_6 of **11i**.*

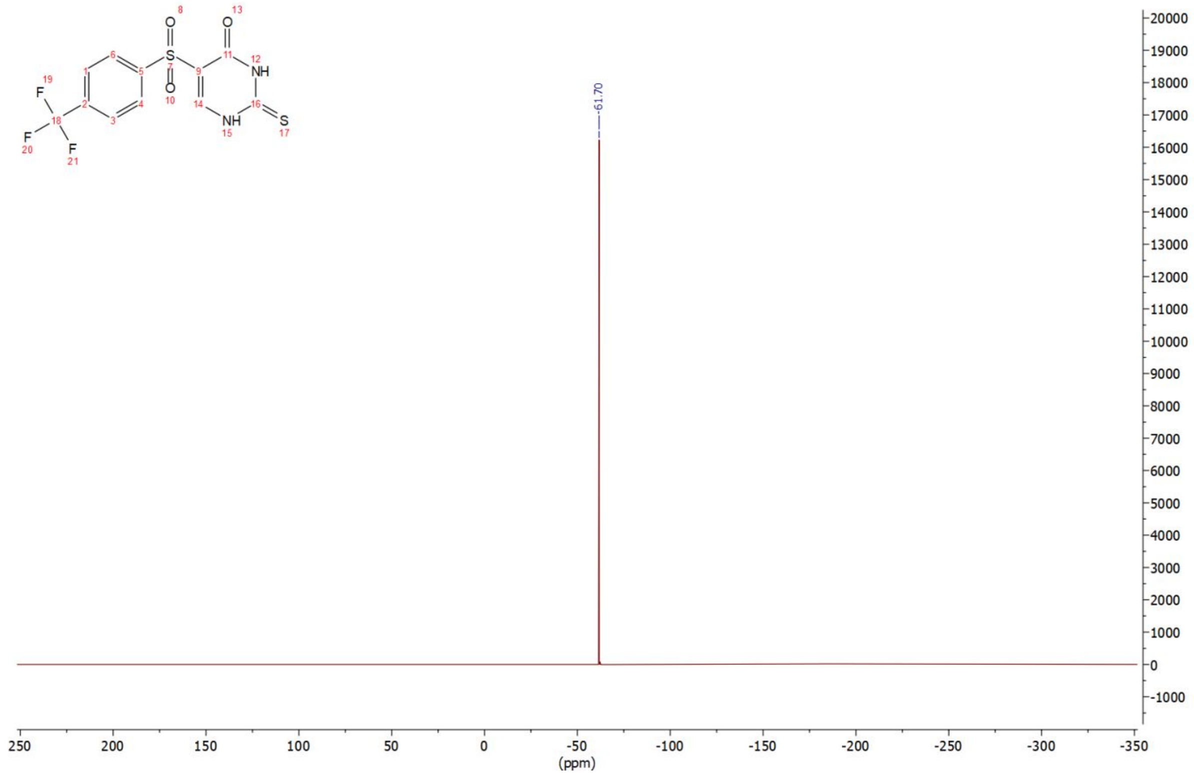

Figure S 75. *^{19}F (376 MHz) NMR-Spectra in DMSO-d_6 of **11i**.*

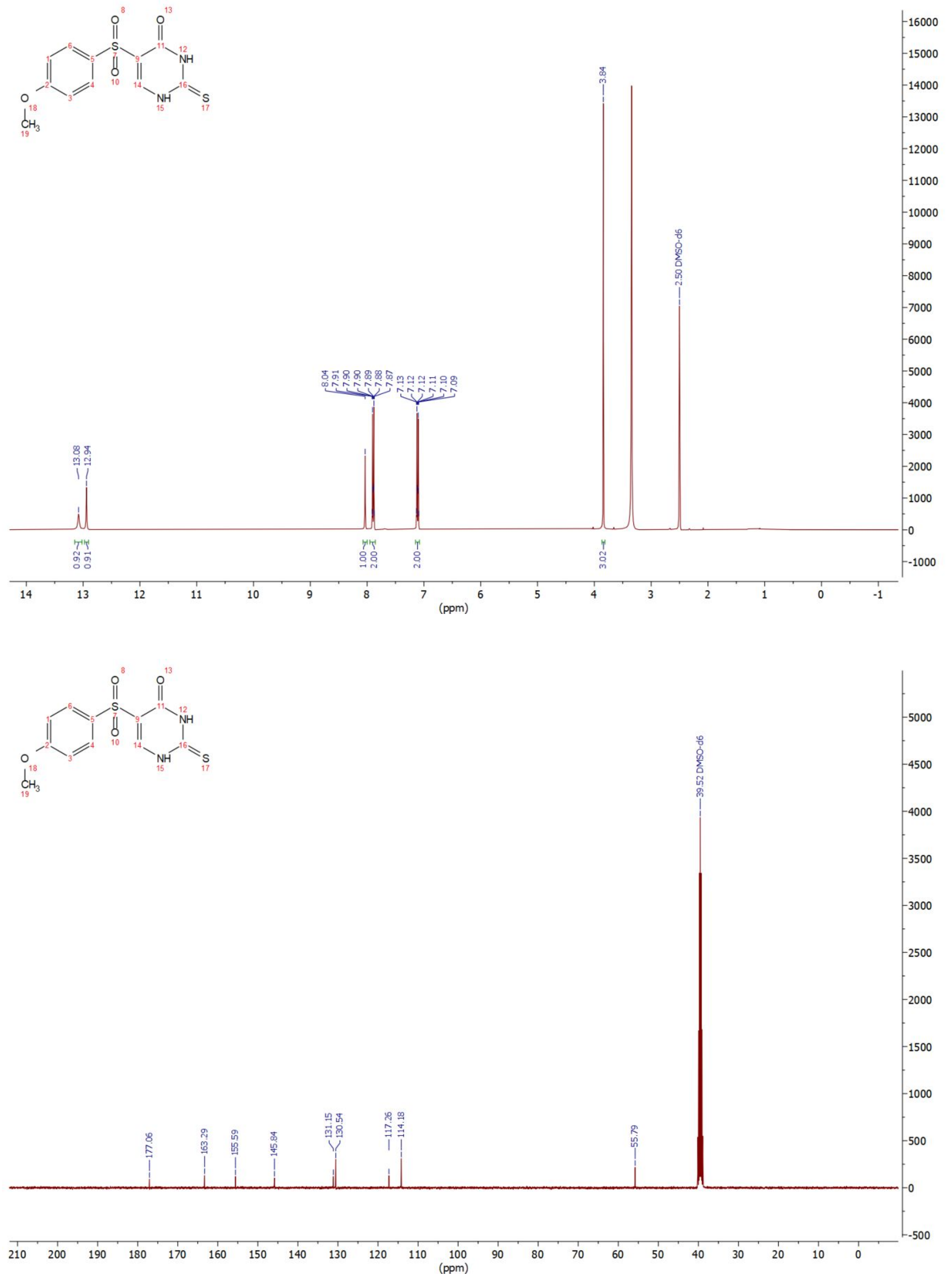

Figure S 76. *^{1}H (400 MHz) and $^{13}C\{1H\}$ (101 MHz) NMR-Spectra in DMSO-d_6 of* ***11j****.*

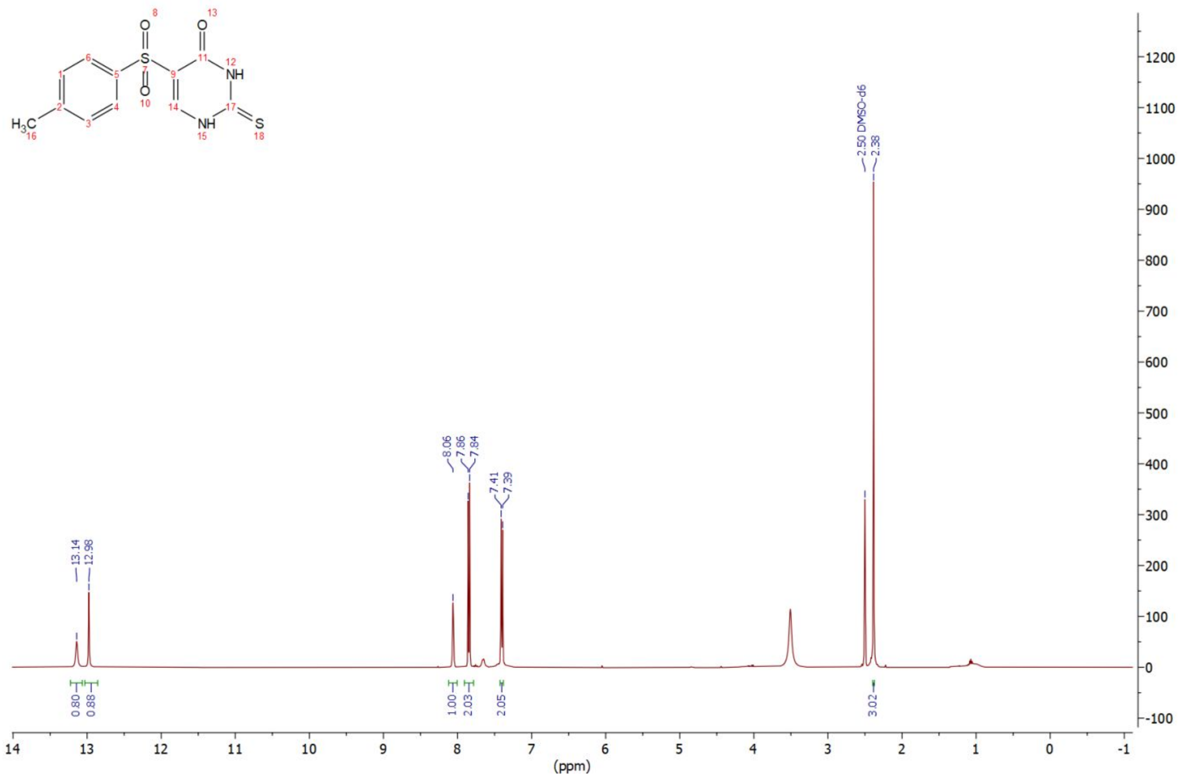

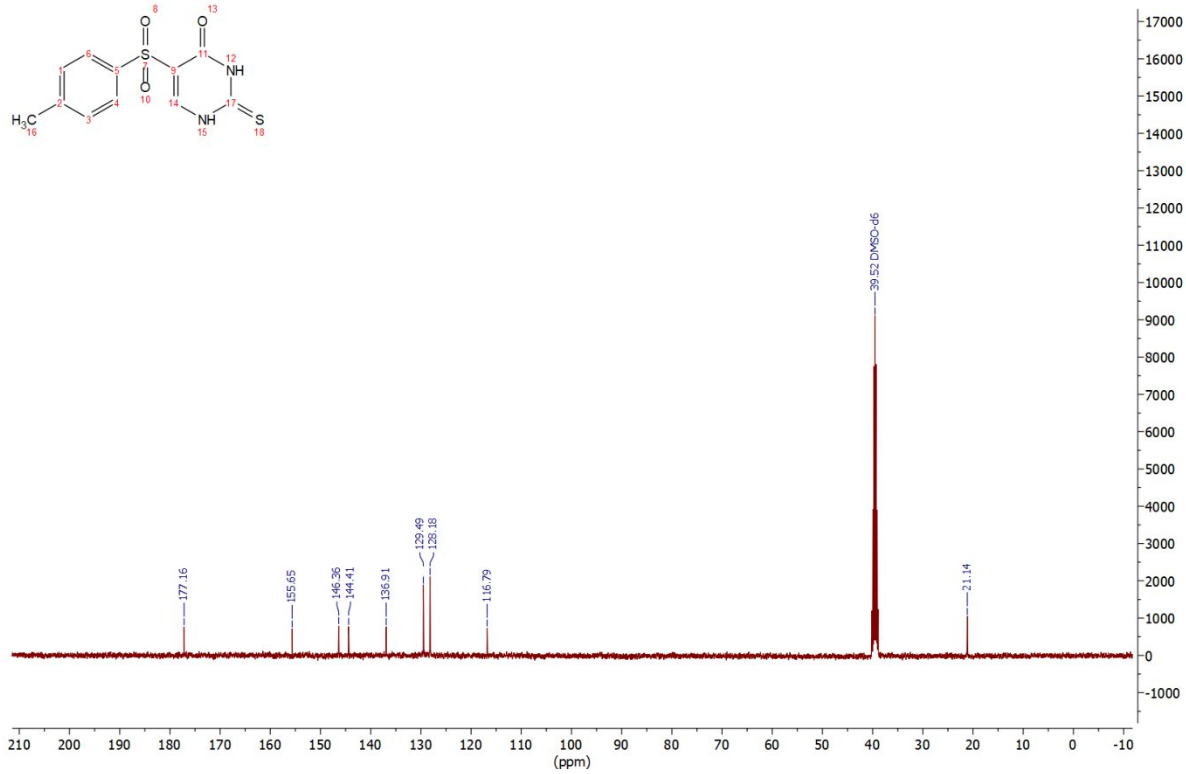

Figure S 77. *^{1}H (400 MHz) and $^{13}C\{1H\}$ (101 MHz) NMR-Spectra in DMSO-d_6 of* ***11k****.*

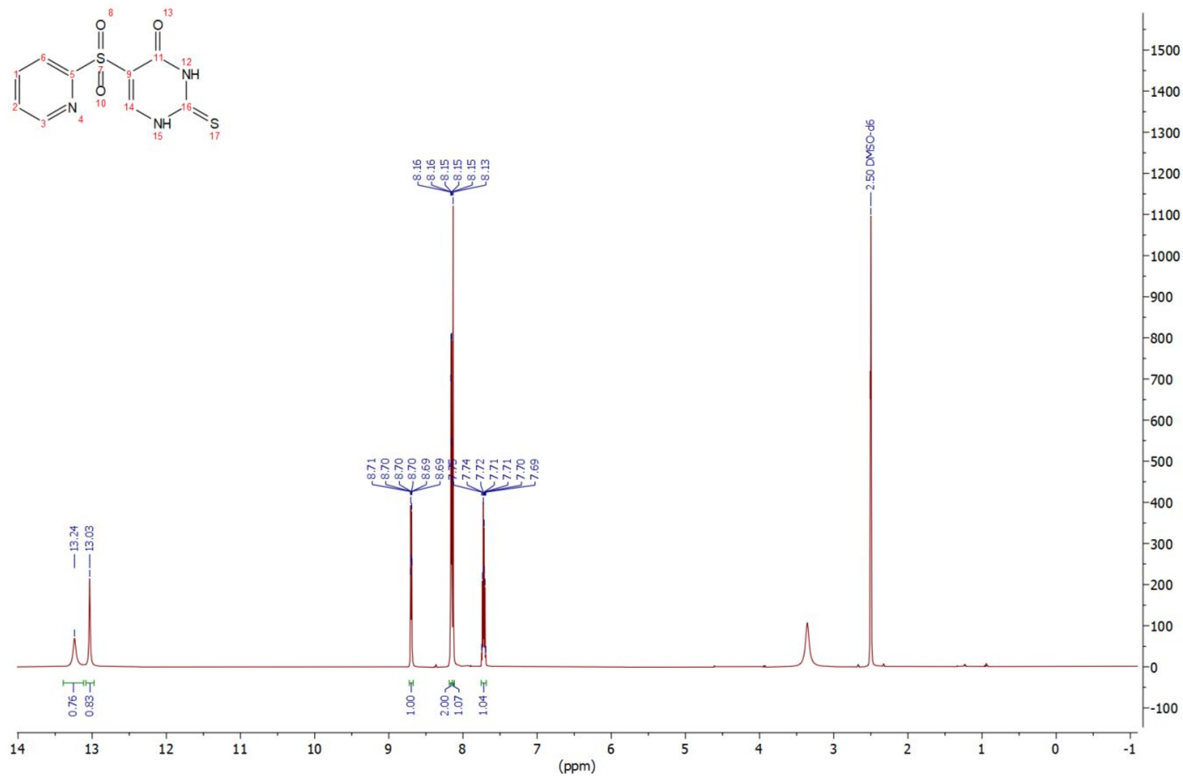

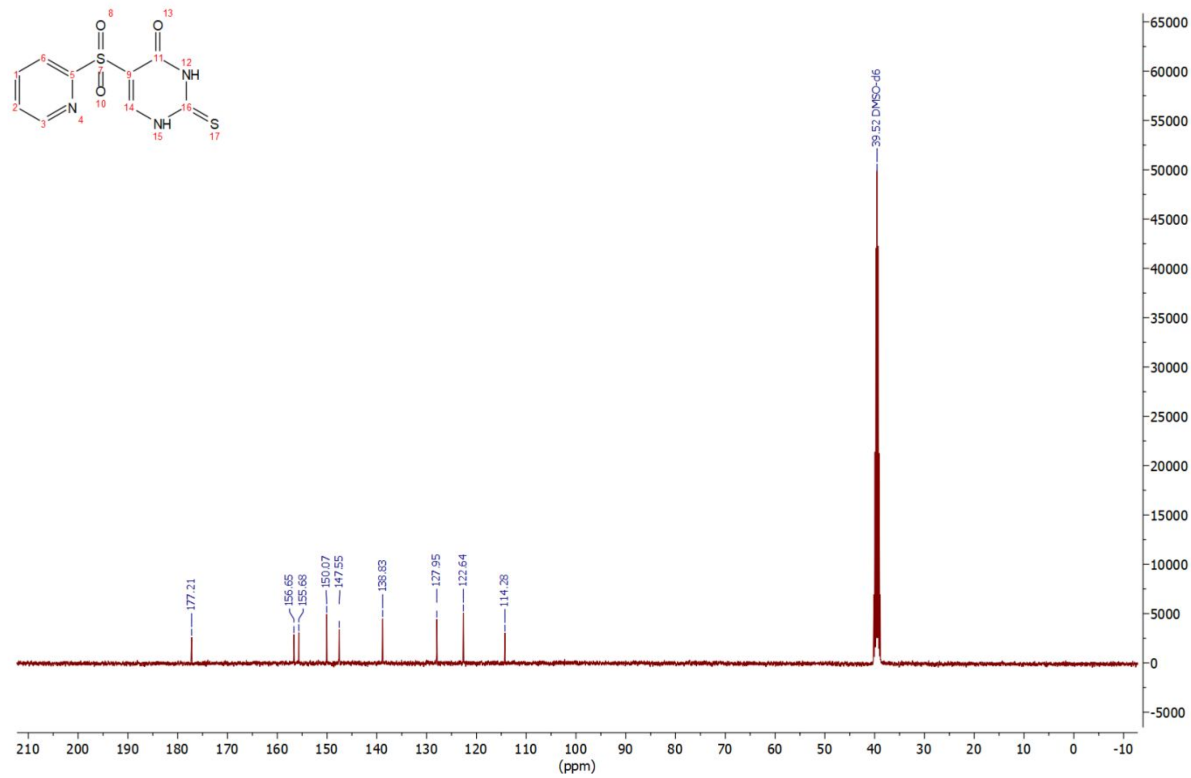

Figure S 78. *^{1}H (400 MHz) and ^{13}C{1H} (101 MHz) NMR-Spectra in DMSO-d$_6$ of **11n**.*

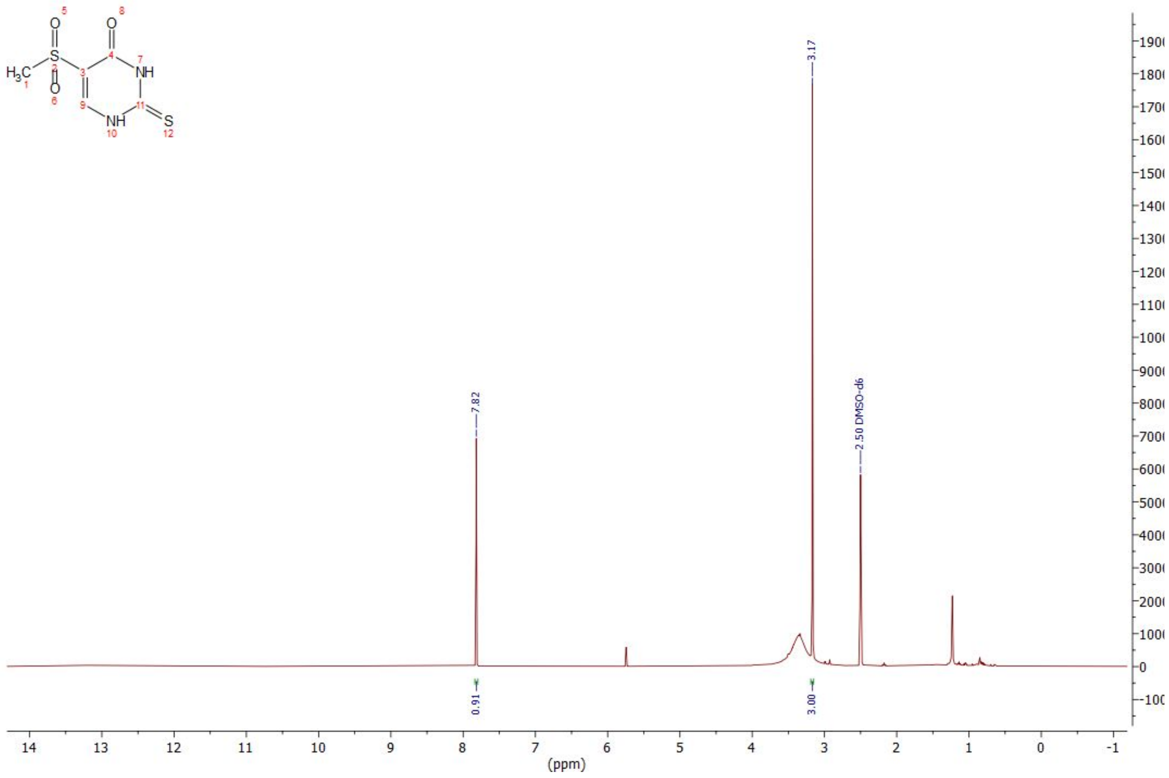

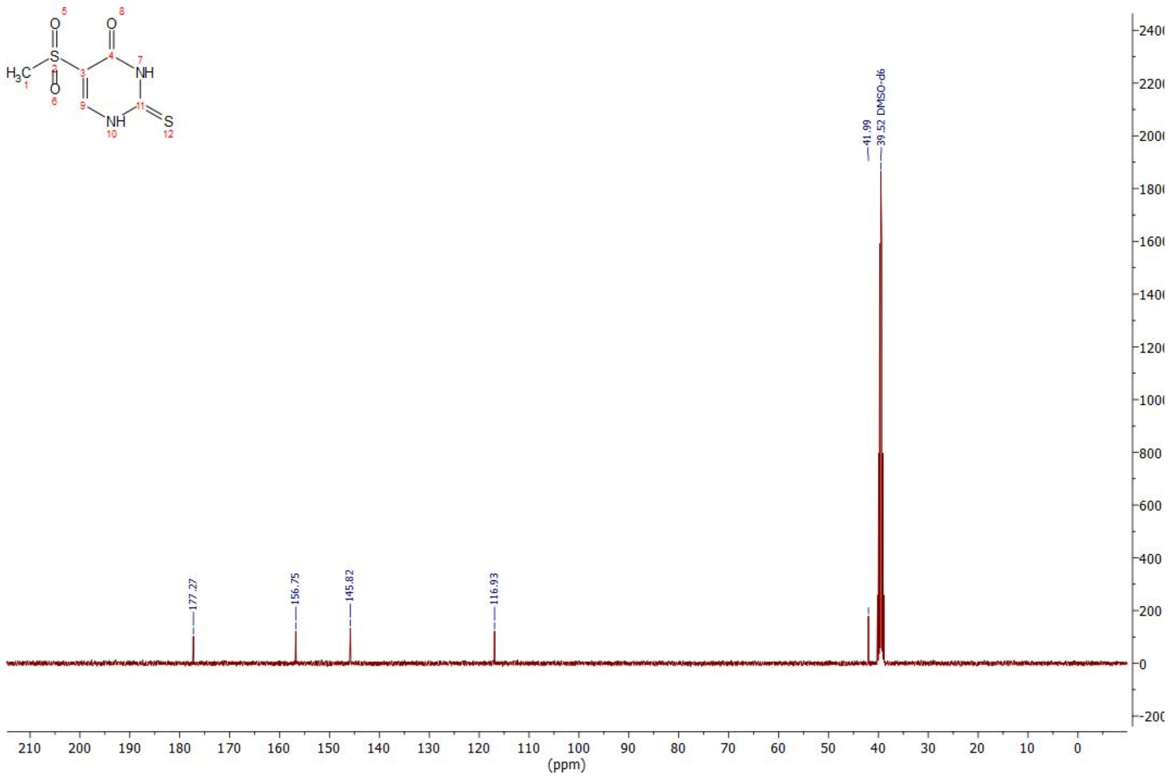

Figure S 79. *^{1}H (400 MHz) and $^{13}C\{^{1}H\}$ (101 MHz) NMR-Spectra in DMSO-d_6 of **11r**.*

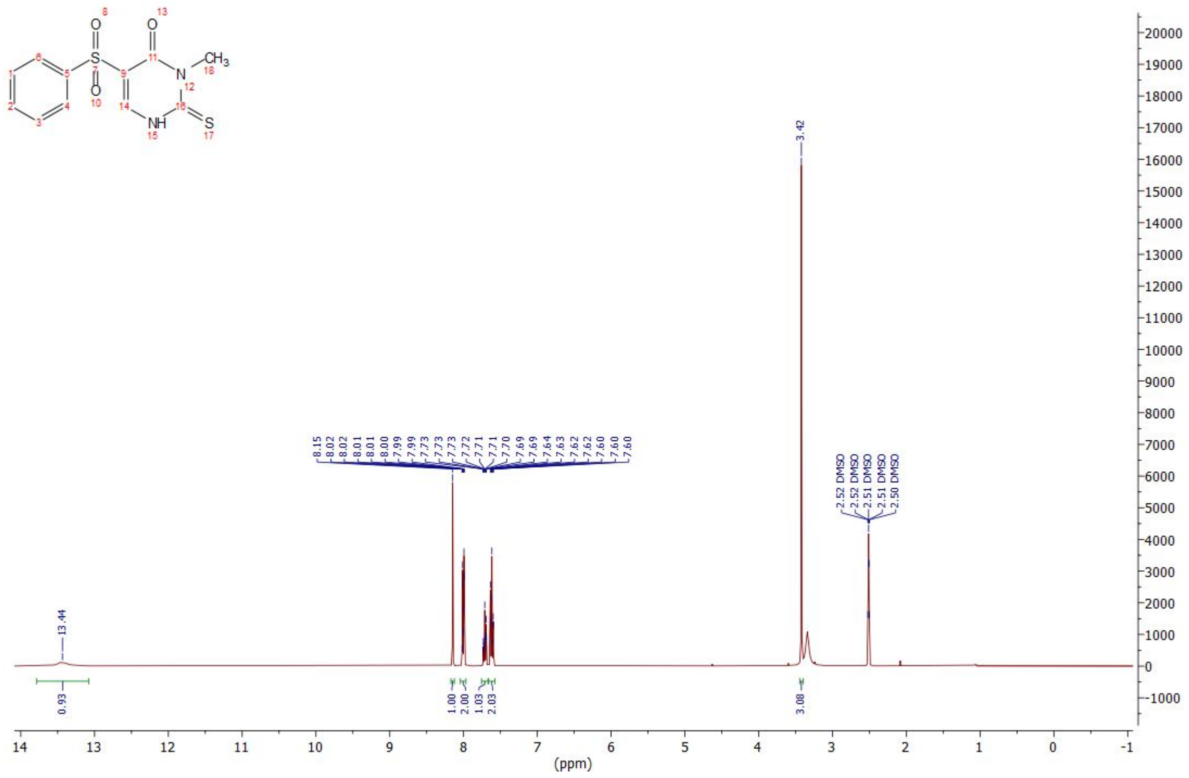

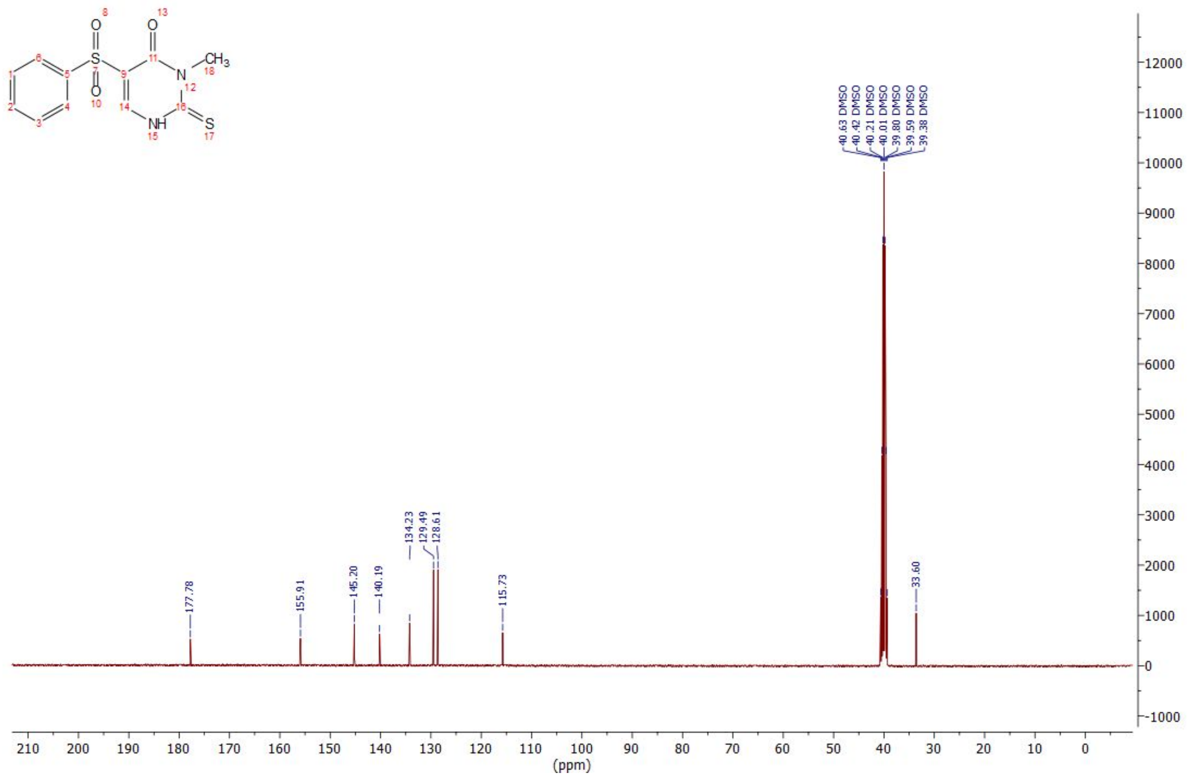

Figure S 80. *^{1}H (400 MHz) and $^{13}C\{1H\}$ (101 MHz) NMR-Spectra in DMSO-d_6 of **12a**.*

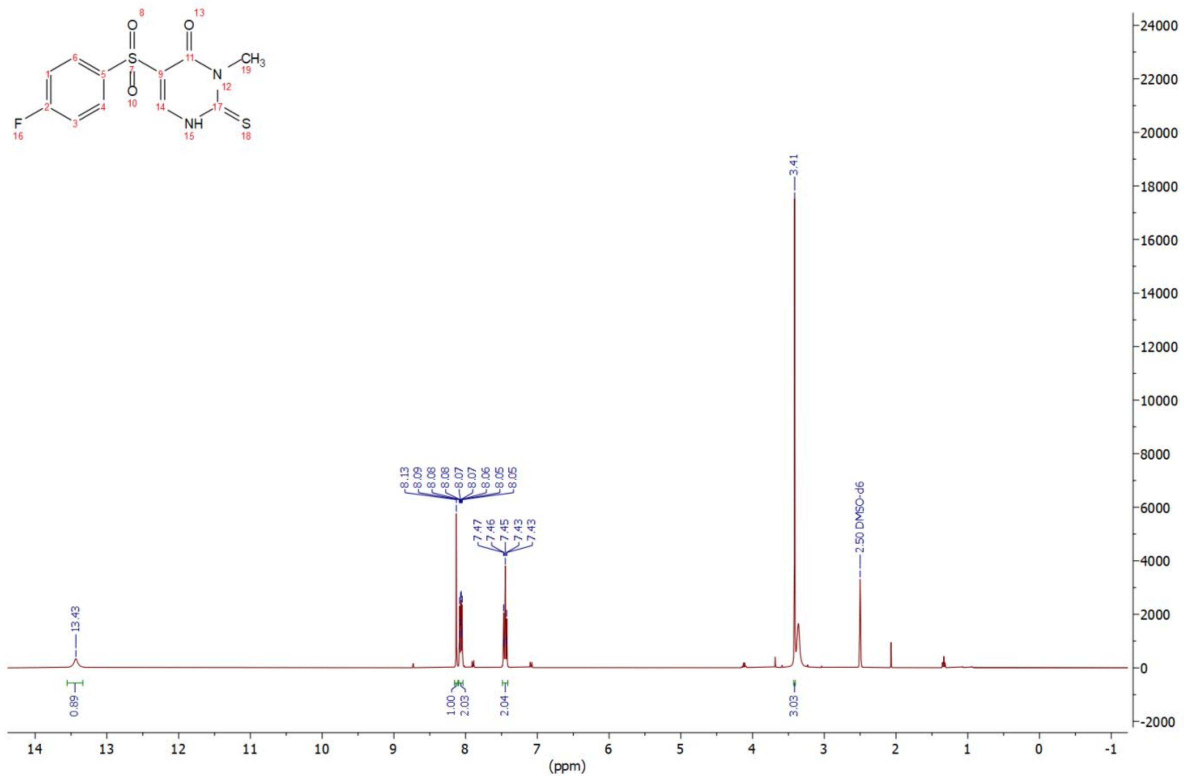

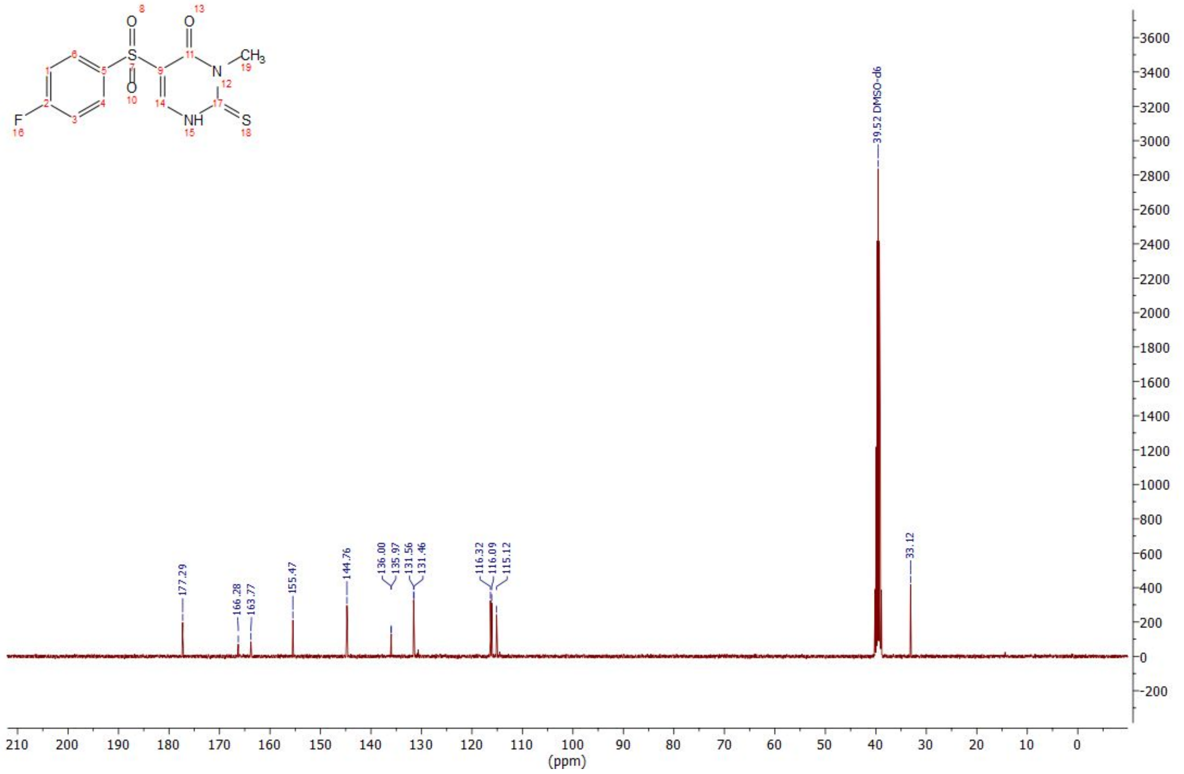

Figure S 81. *^{1}H (400 MHz) and $^{13}C\{1H\}$ (101 MHz) NMR-Spectra in DMSO-d_6 of **12b**.*

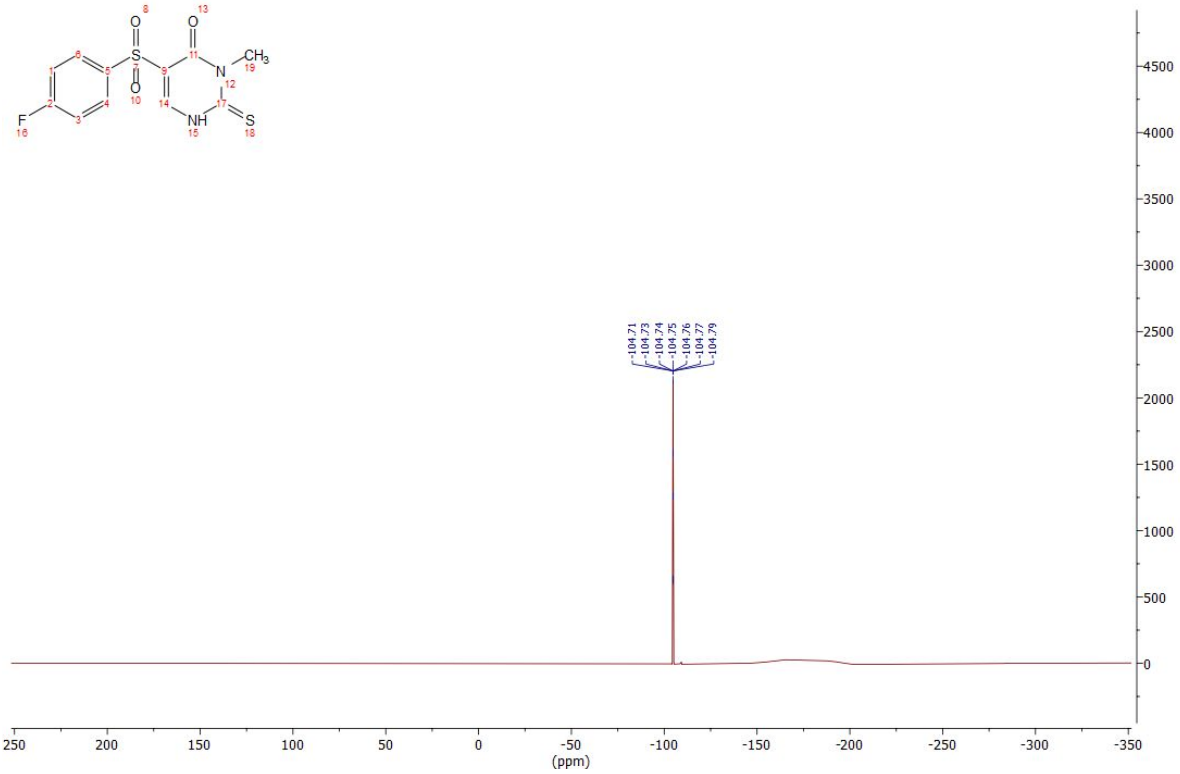

Figure S 82. *^{19}F (376 MHz) NMR-Spectra in DMSO-d$_6$ of **12b**.*

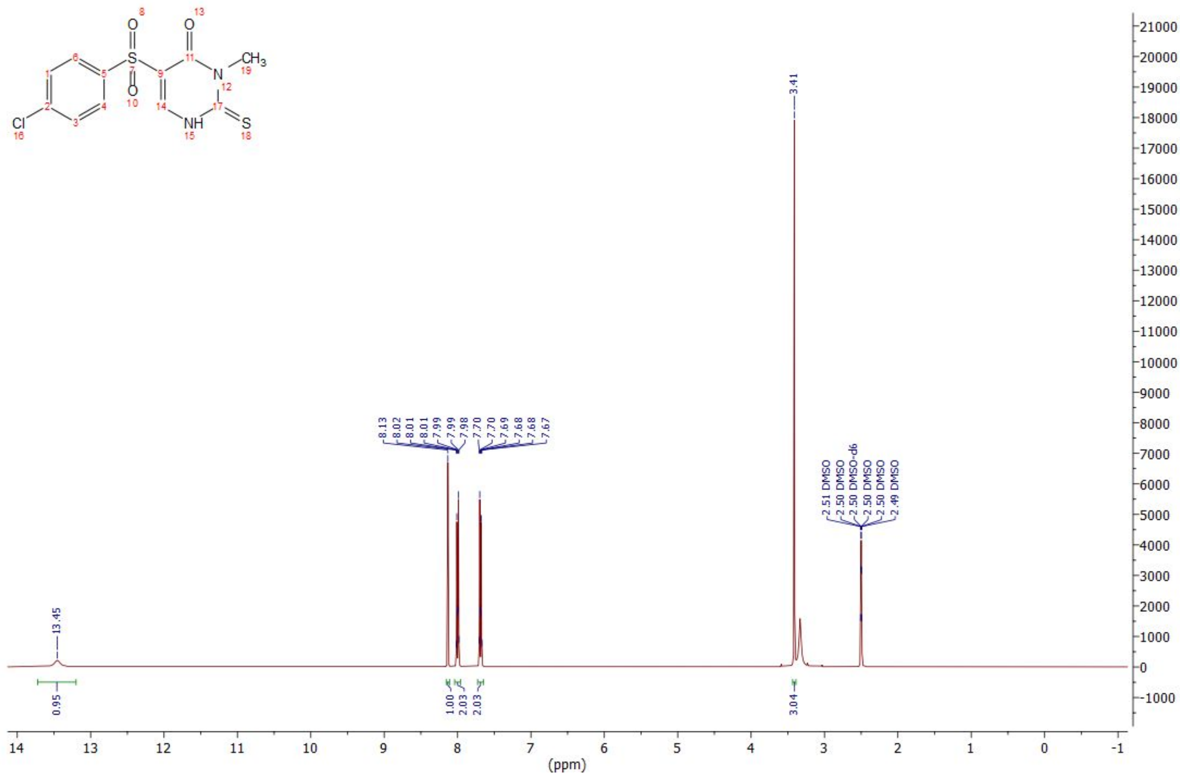

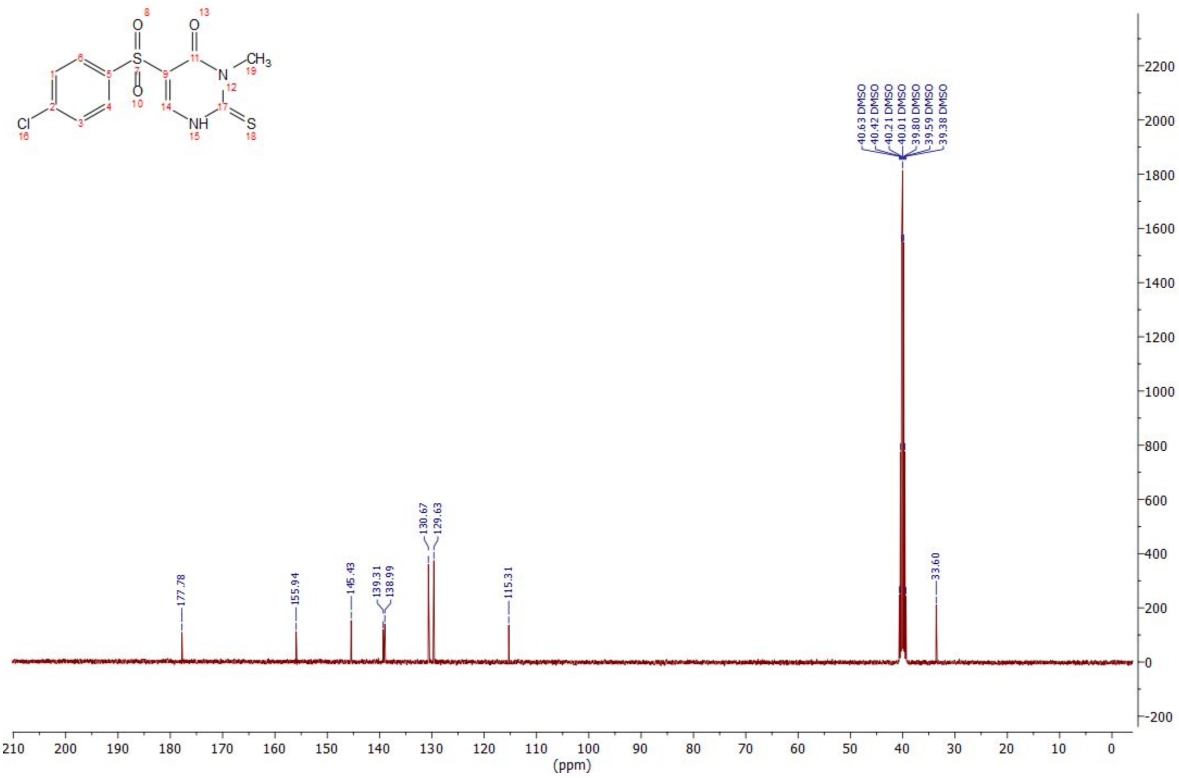

Figure S 83. *^{1}H (400 MHz) and $^{13}C\{1H\}$ (101 MHz) NMR-Spectra in DMSO-d_6 of **12c**.*

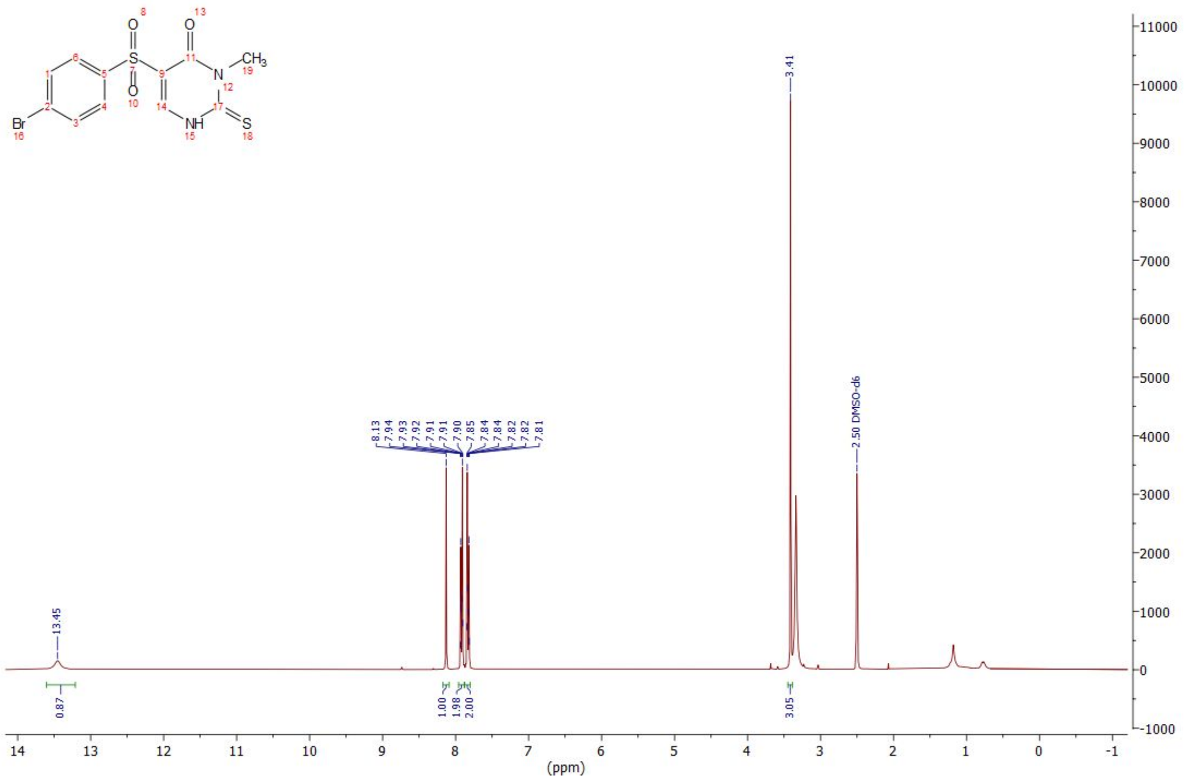

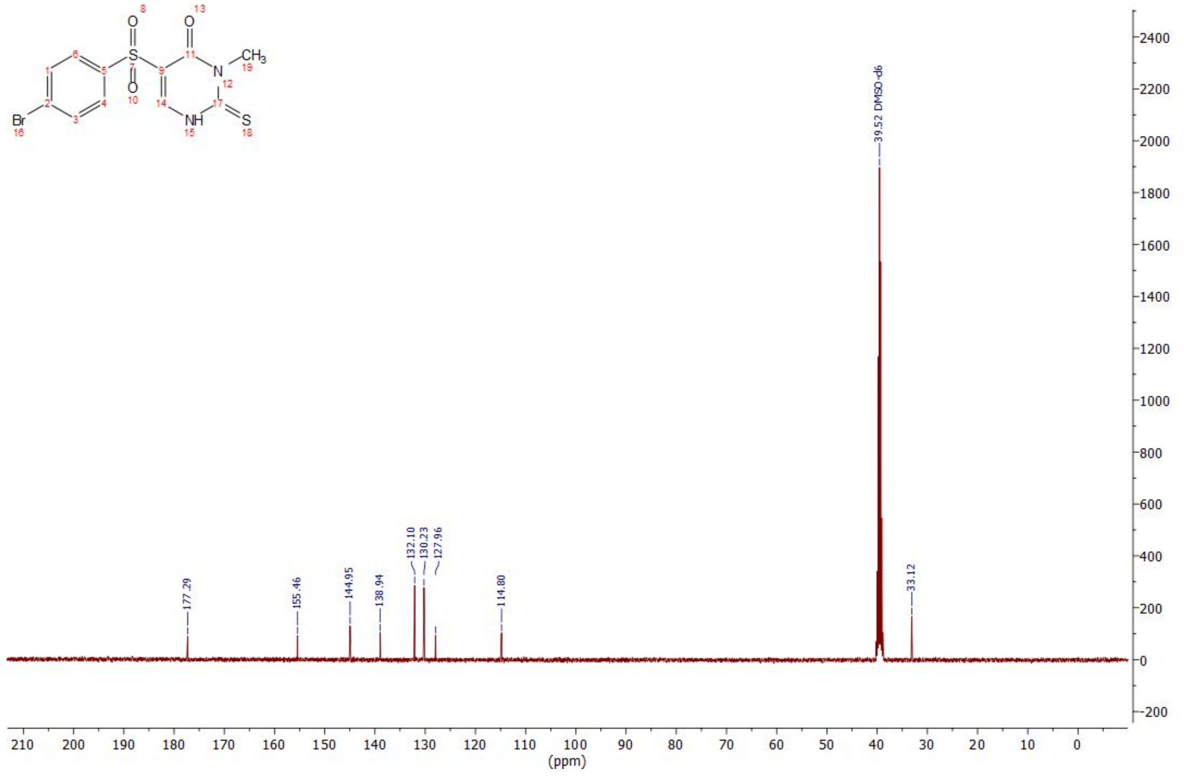

Figure S 84. *^{1}H (400 MHz) and $^{13}C\{1H\}$ (101 MHz) NMR-Spectra in DMSO-d_6 of **12d**.*

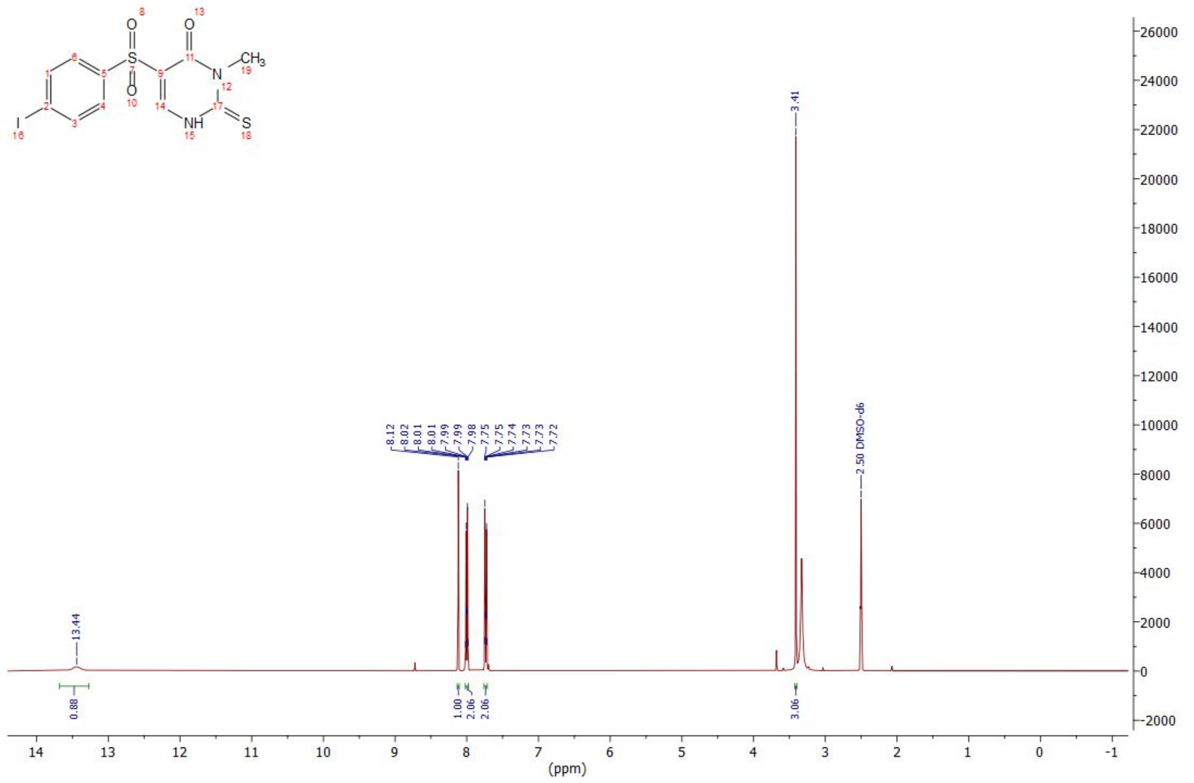

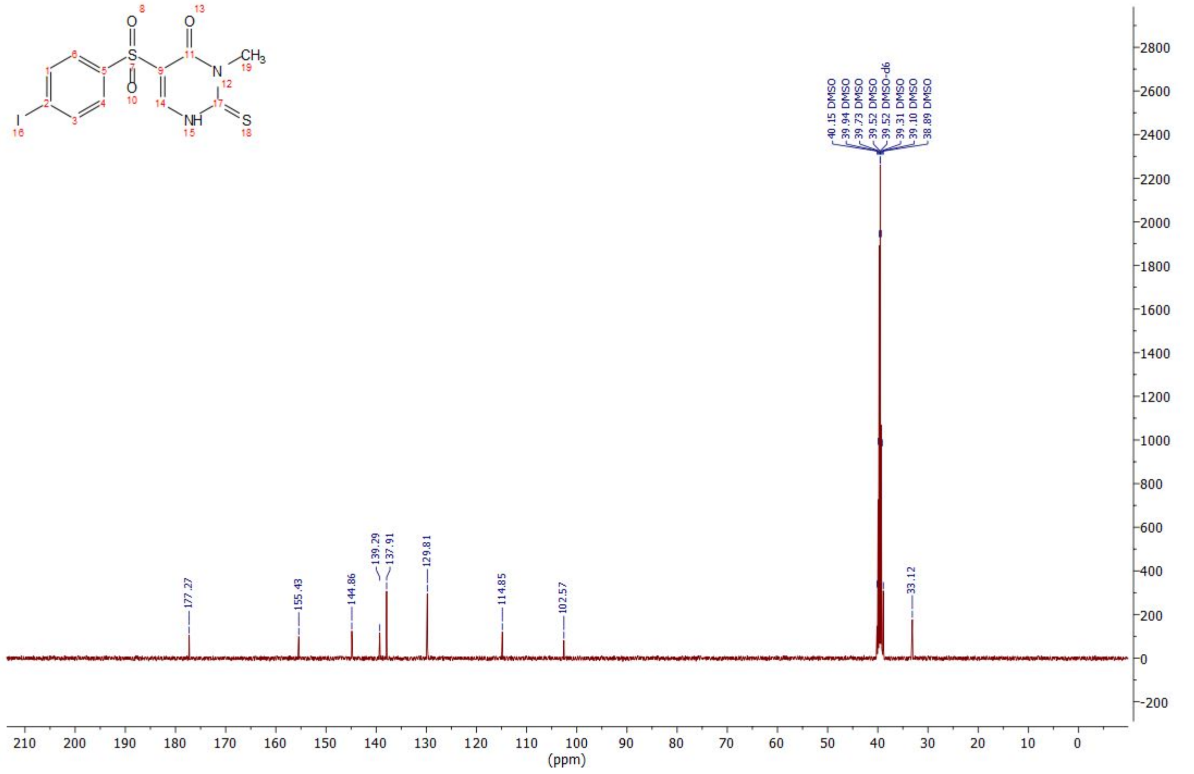

Figure S 85. *^{1}H (400 MHz) and ^{13}C{1H} (101 MHz) NMR-Spectra in DMSO-d$_6$ of **12e**.*

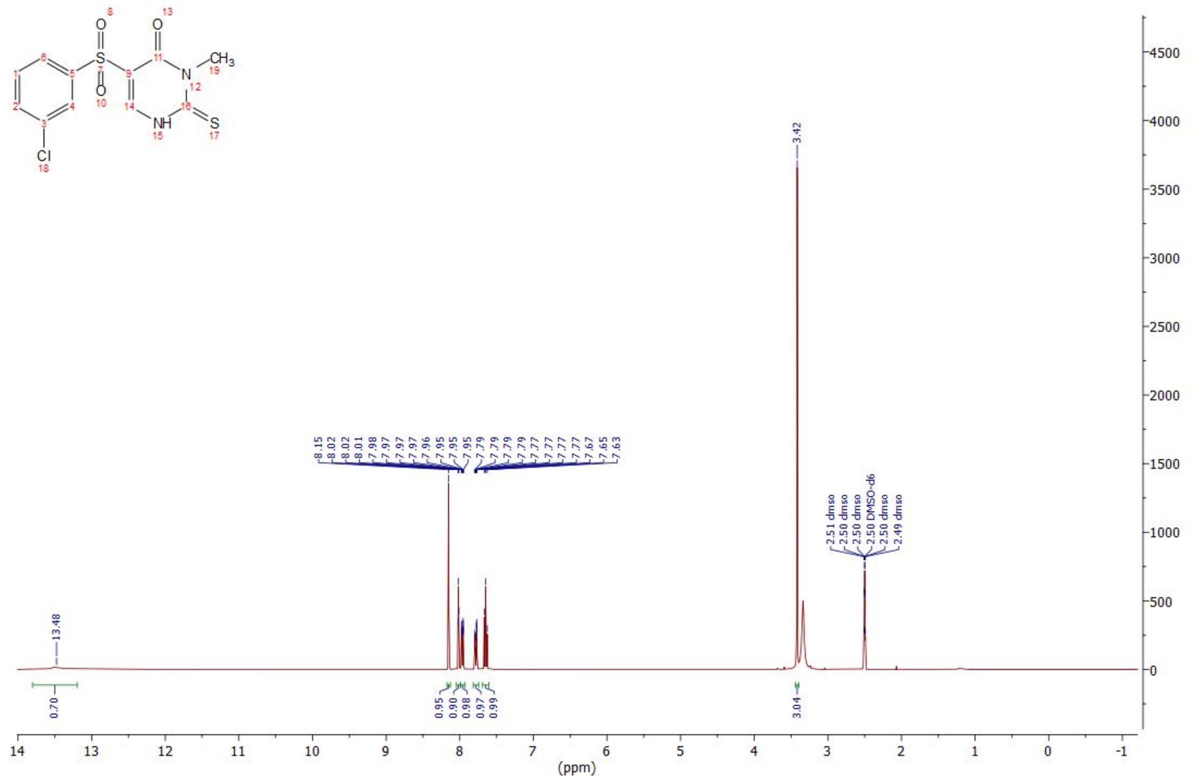

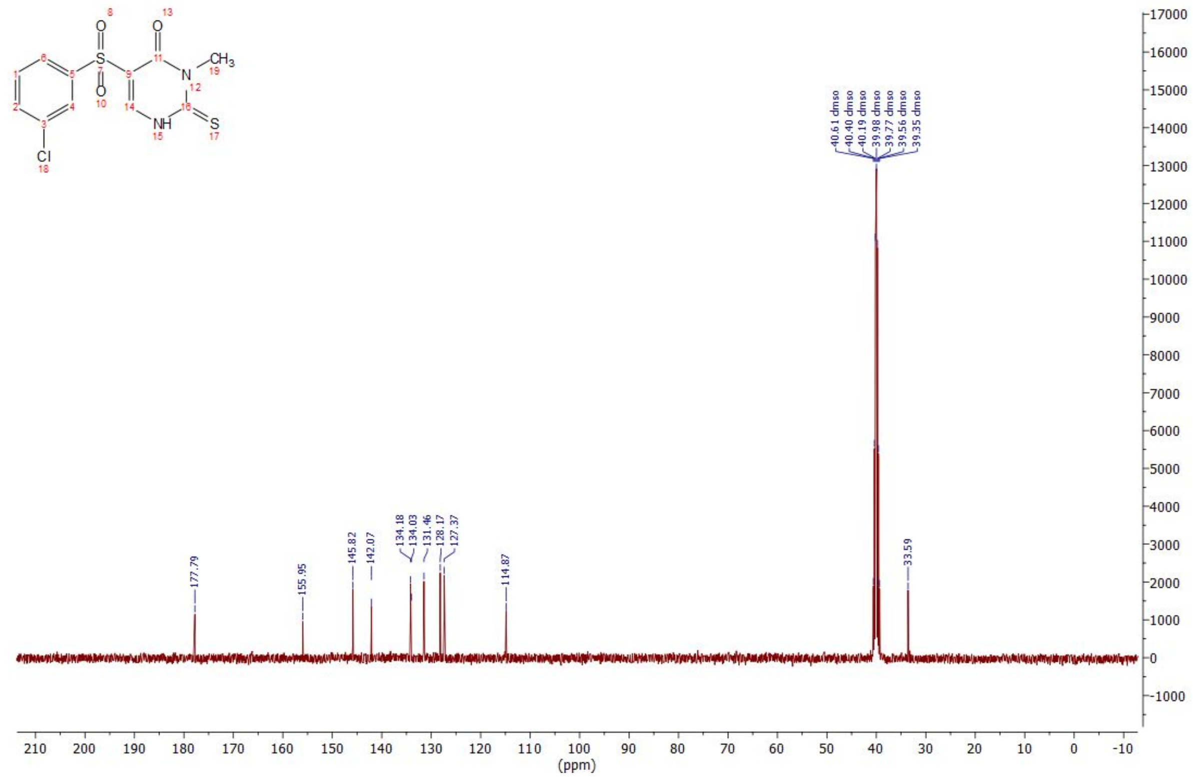

Figure S 86. *^{1}H (400 MHz) and ^{13}C{1H} (101 MHz) NMR-Spectra in DMSO-d$_6$ of **12f**.*

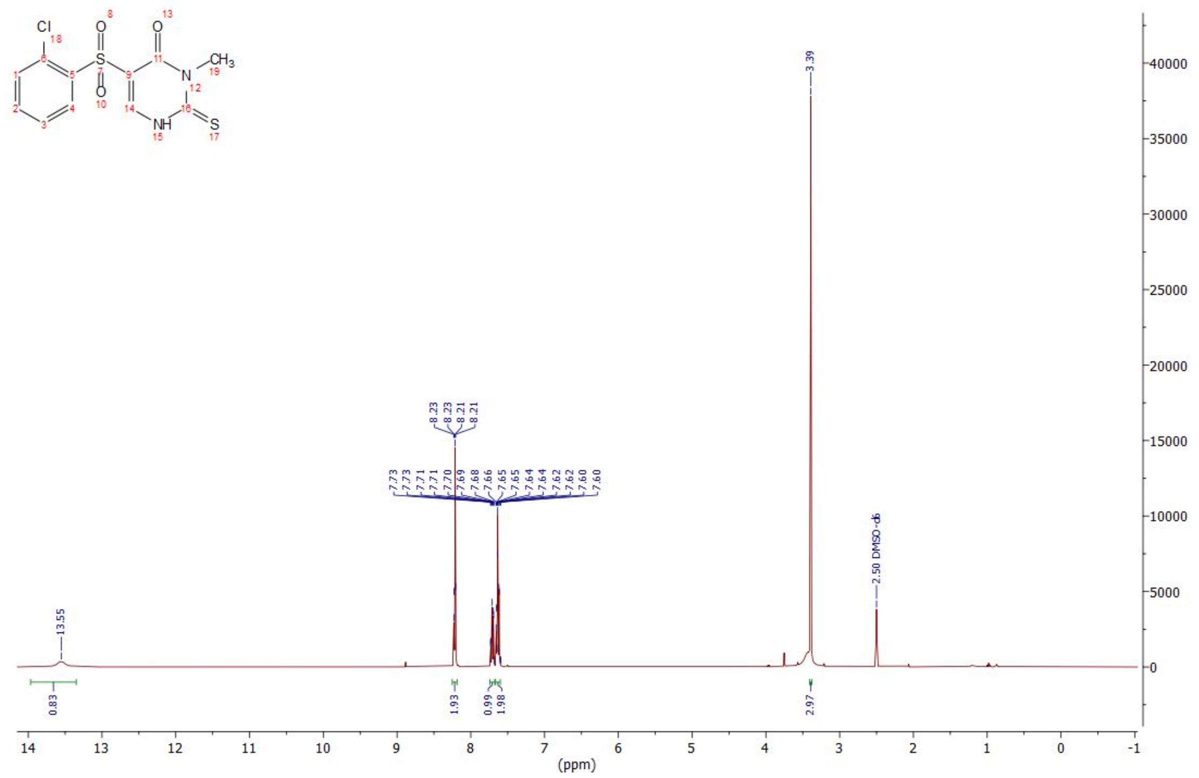

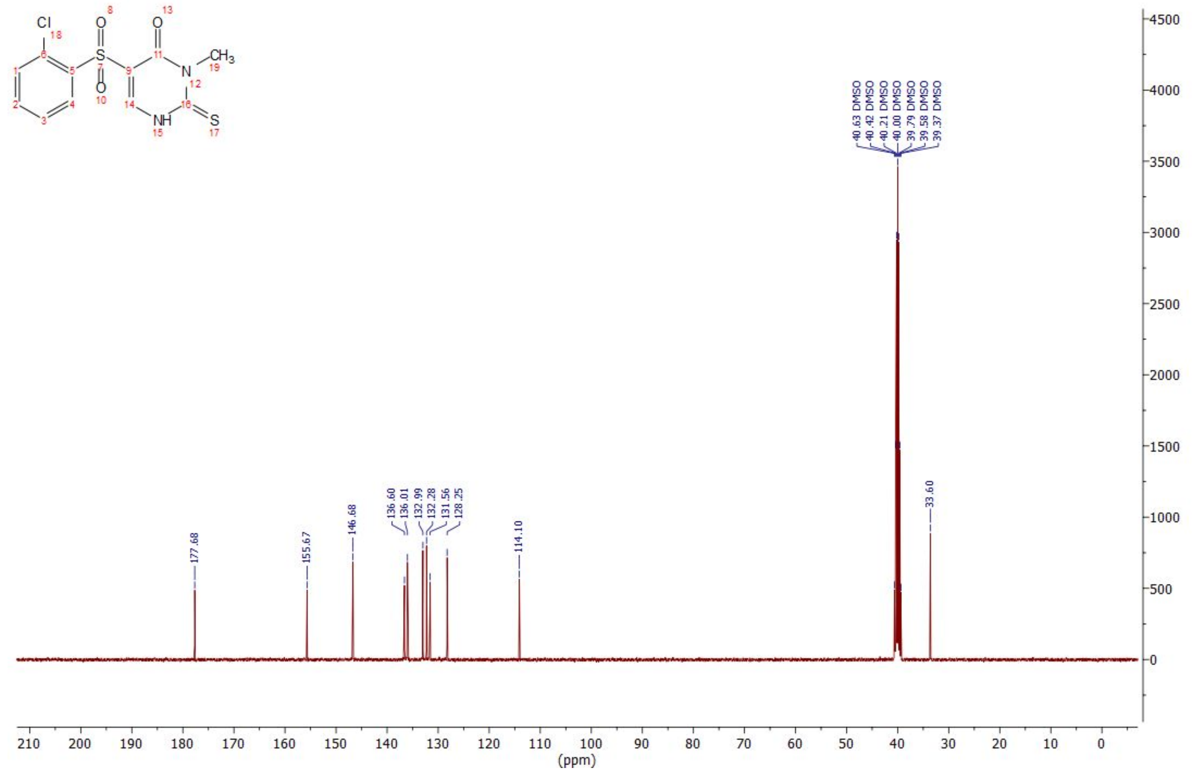

Figure S 87. *^1H (400 MHz) and $^{13}C\{1H\}$ (101 MHz) NMR-Spectra in DMSO-d_6 of **12g**.*

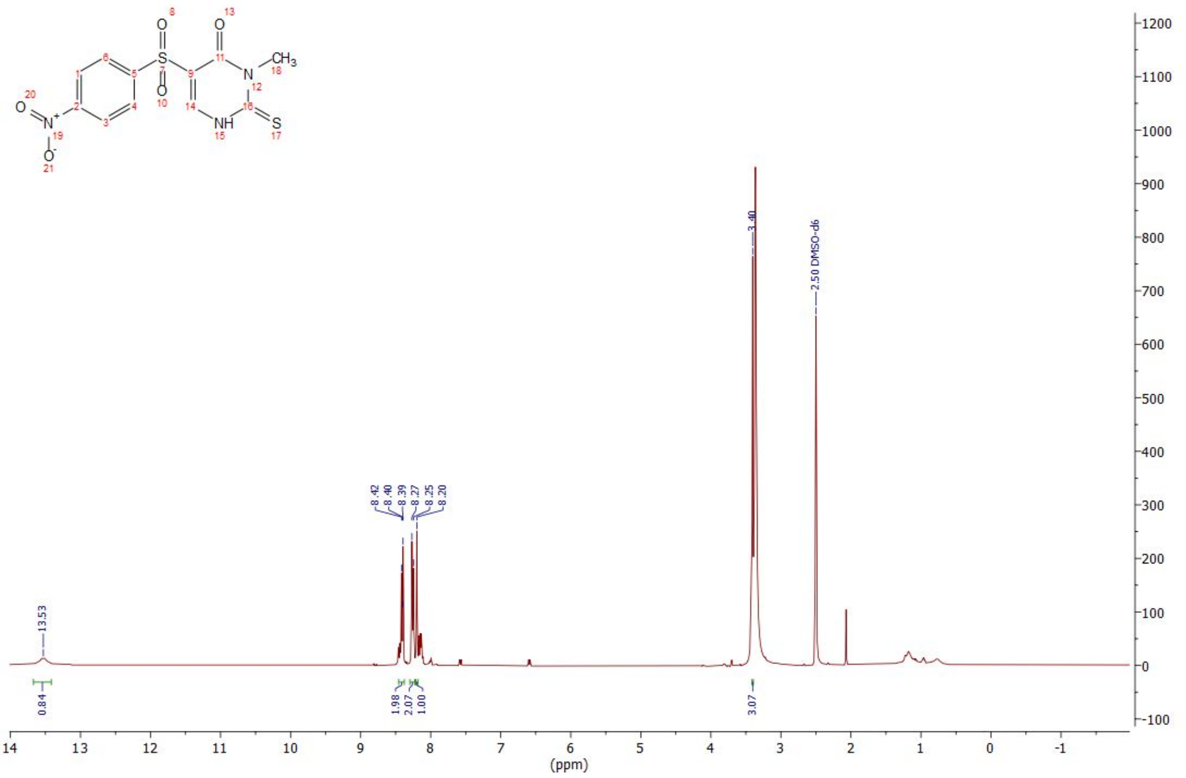

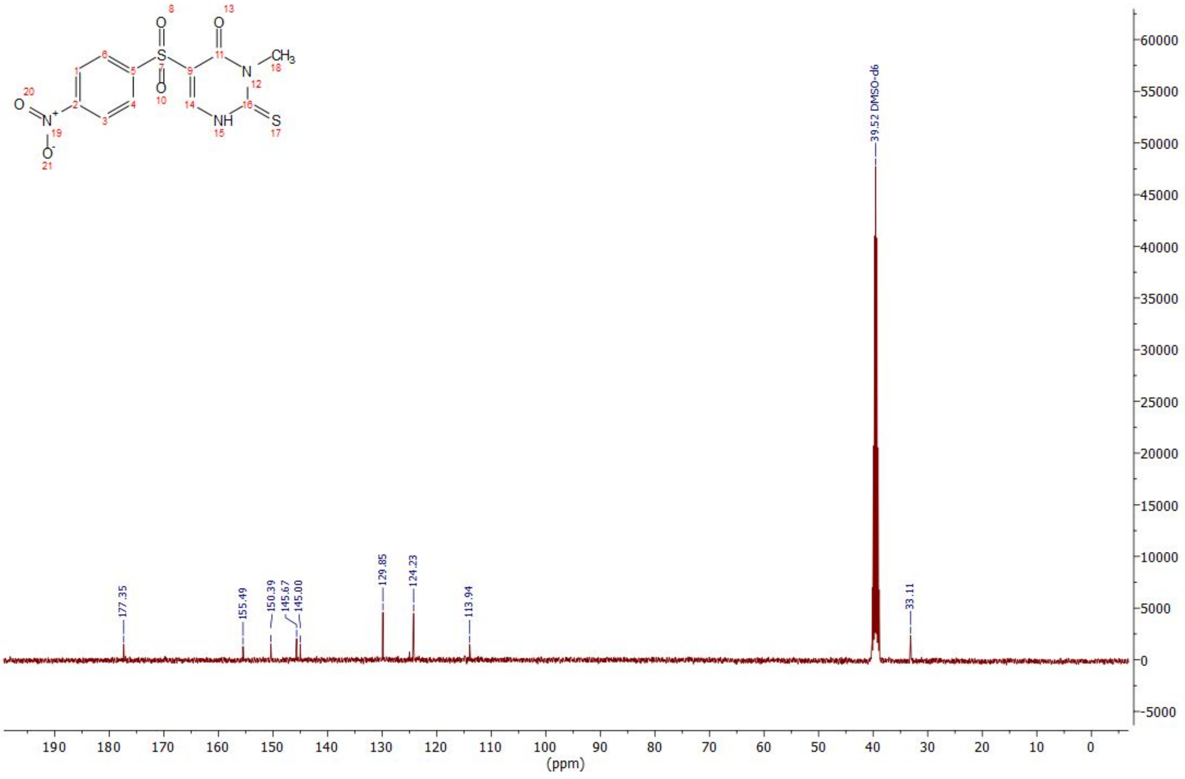

Figure S 88. *^{1}H (400 MHz) and $^{13}C\{1H\}$ (101 MHz) NMR-Spectra in DMSO-d_6 of* ***12h****.*

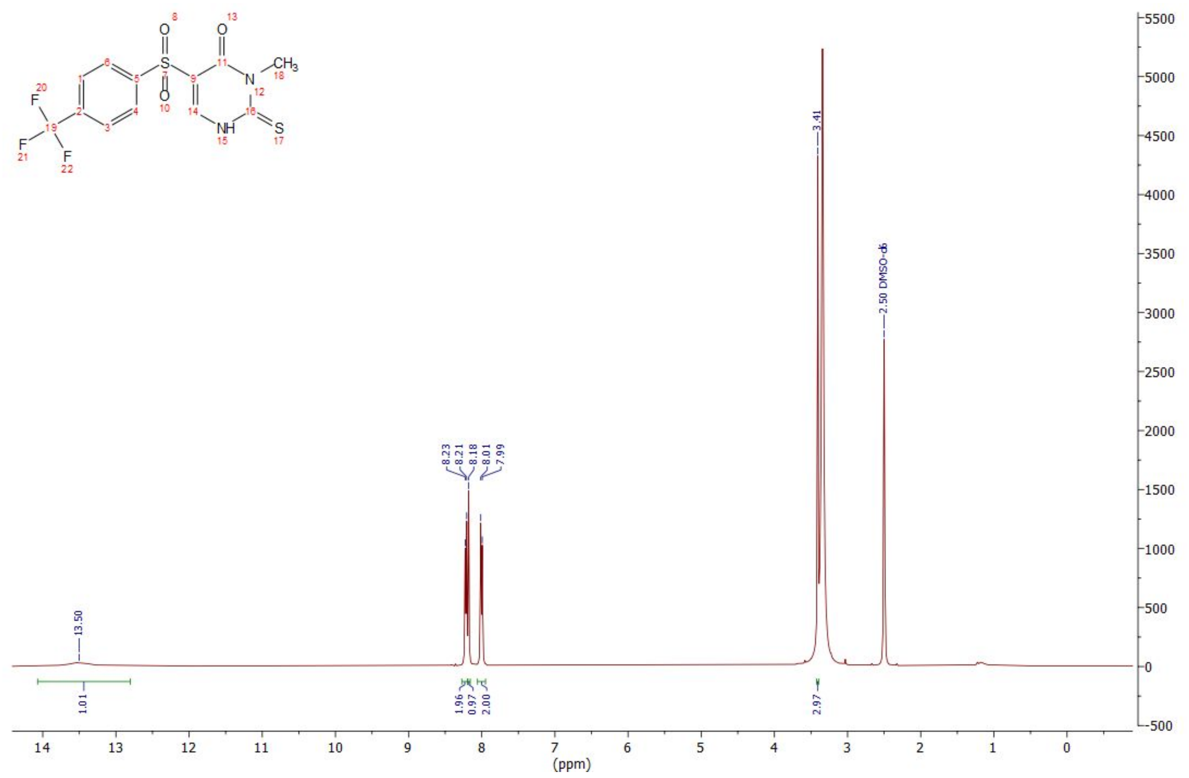

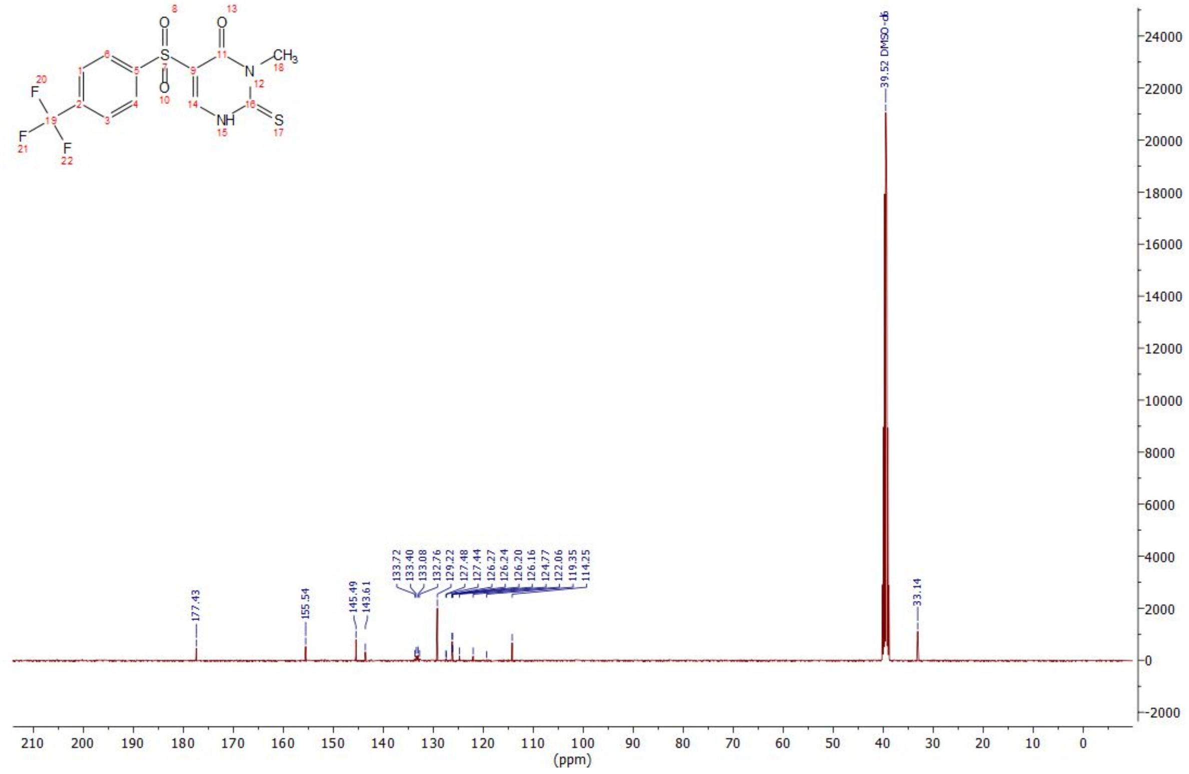

Figure S 89. *^{1}H (400 MHz) and ^{13}C{1H} (101 MHz) NMR-Spectra in DMSO-d$_6$ of **12i**.*

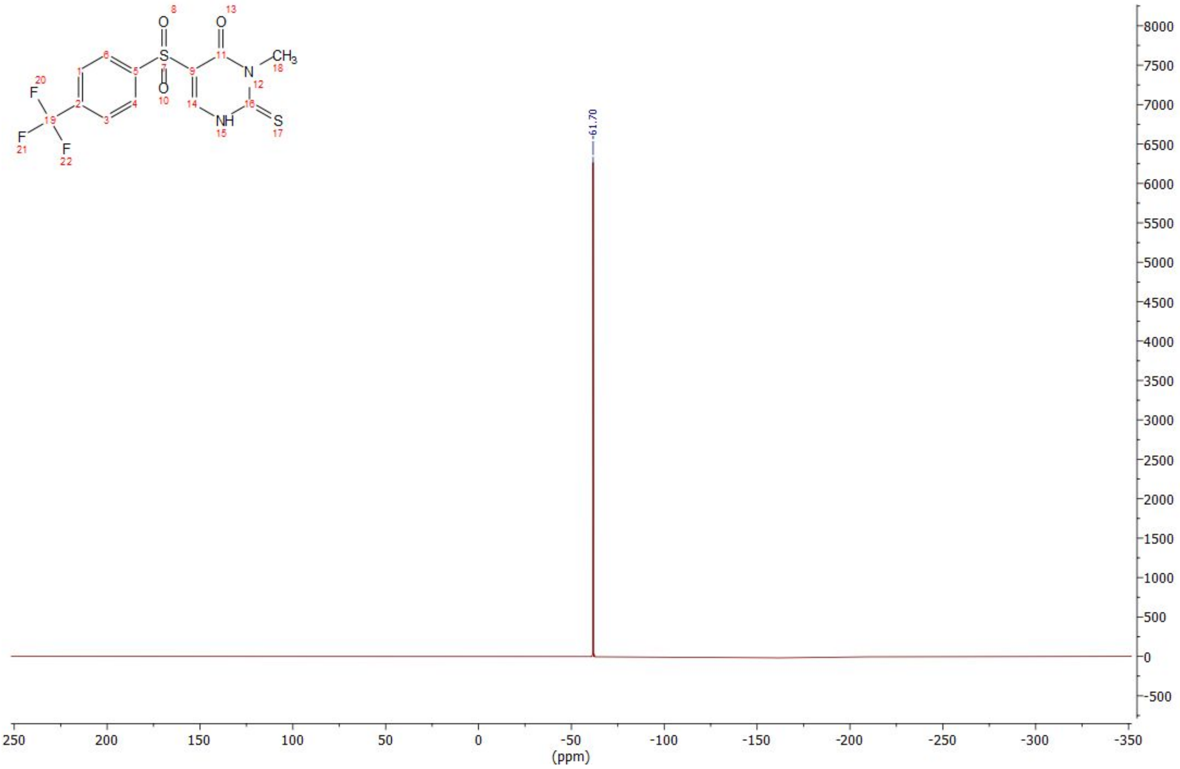

Figure S 90. *^{19}F (376 MHz) NMR-Spectra in DMSO-d_6 of **12i**.*

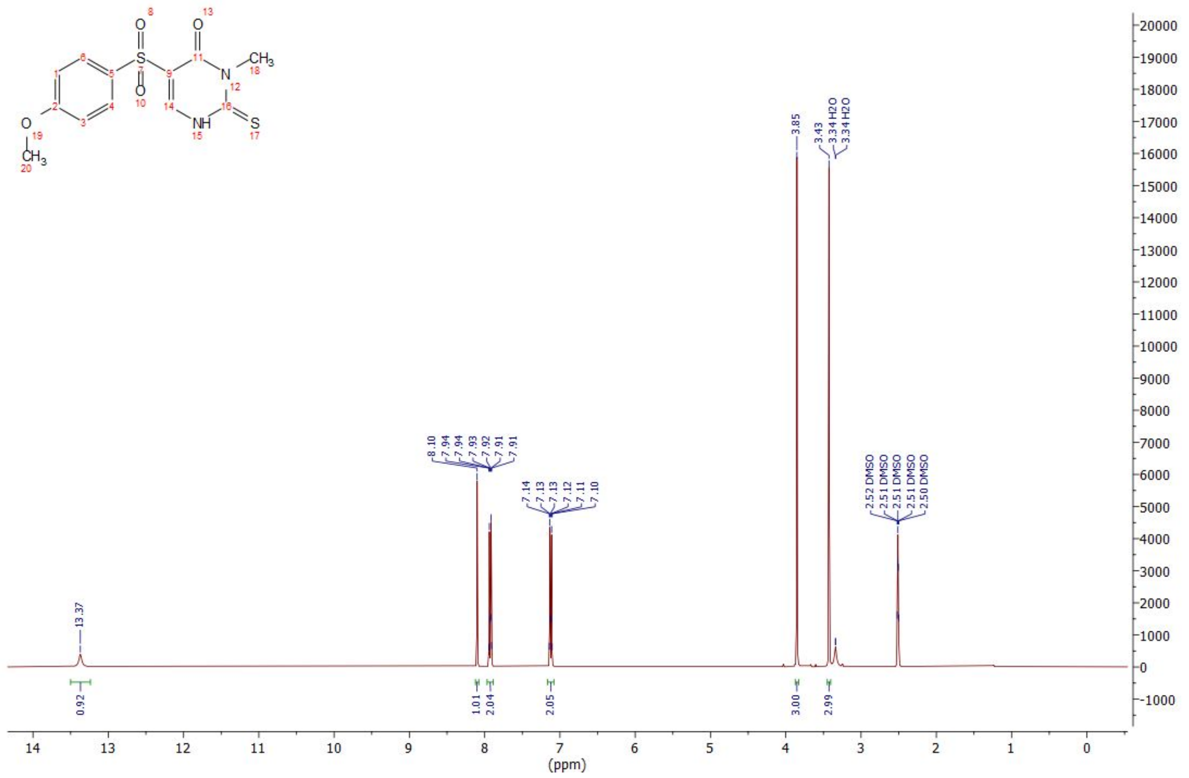

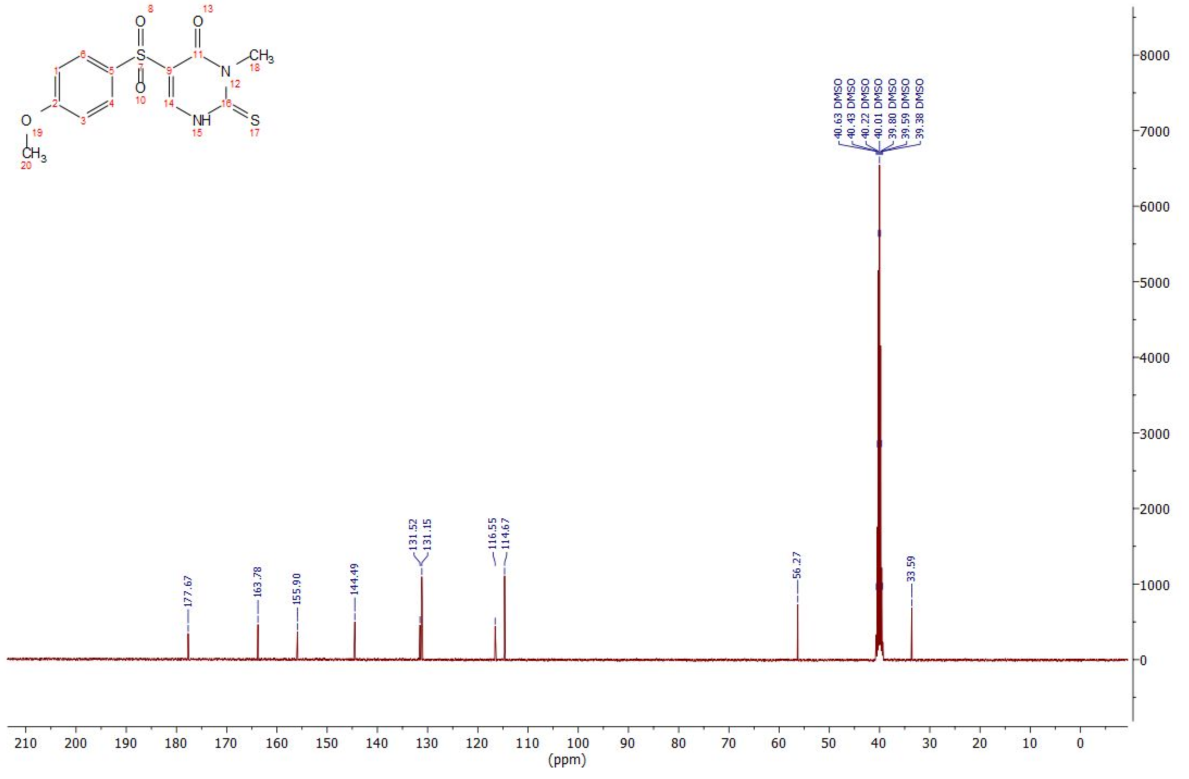

Figure S 91. *^{1}H (400 MHz) and $^{13}C\{1H\}$ (101 MHz) NMR-Spectra in DMSO-d_6 of **12j**.*

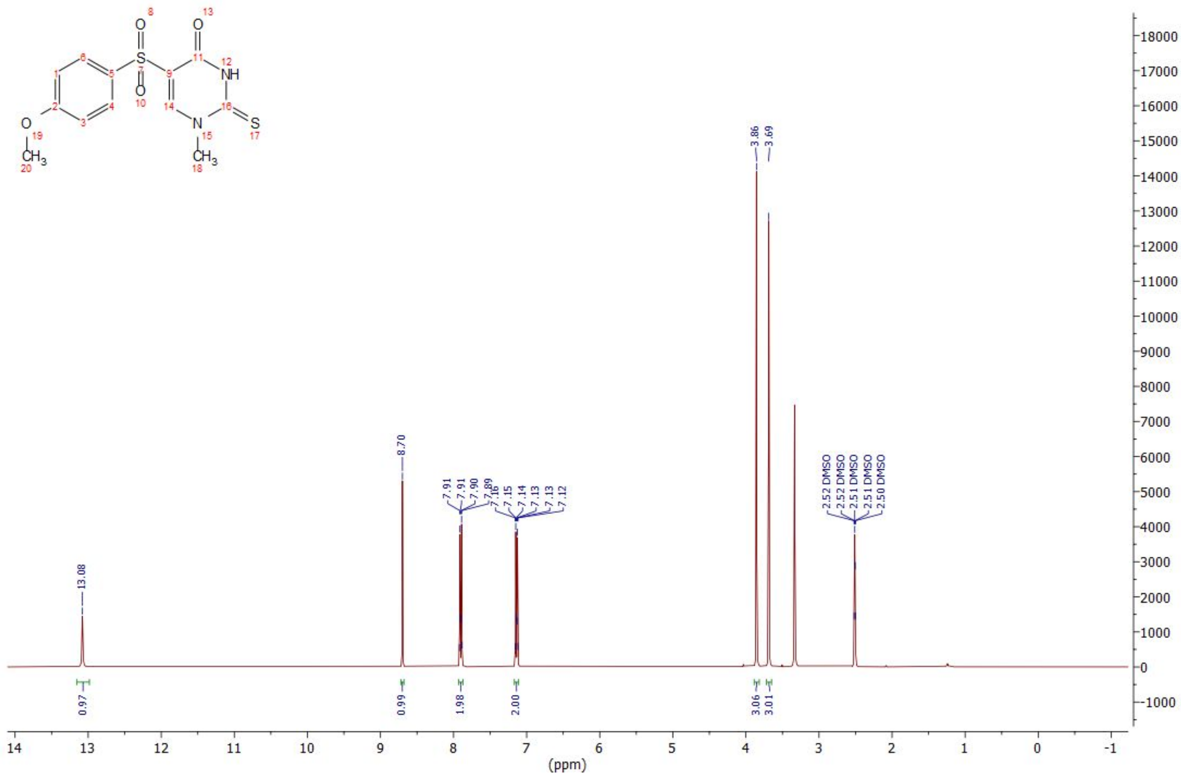

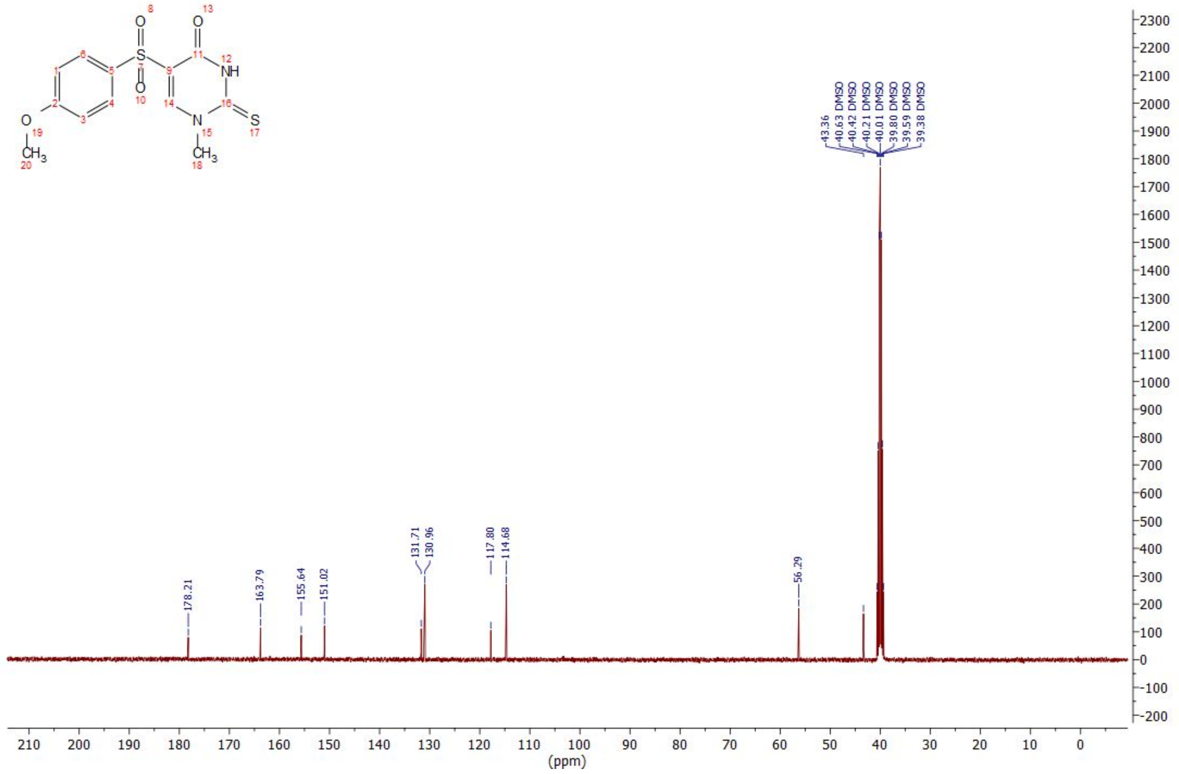

Figure S 92. *^{1}H (400 MHz) and $^{13}C\{1H\}$ (101 MHz) NMR-Spectra in DMSO-d_6 of* ***S5****.*

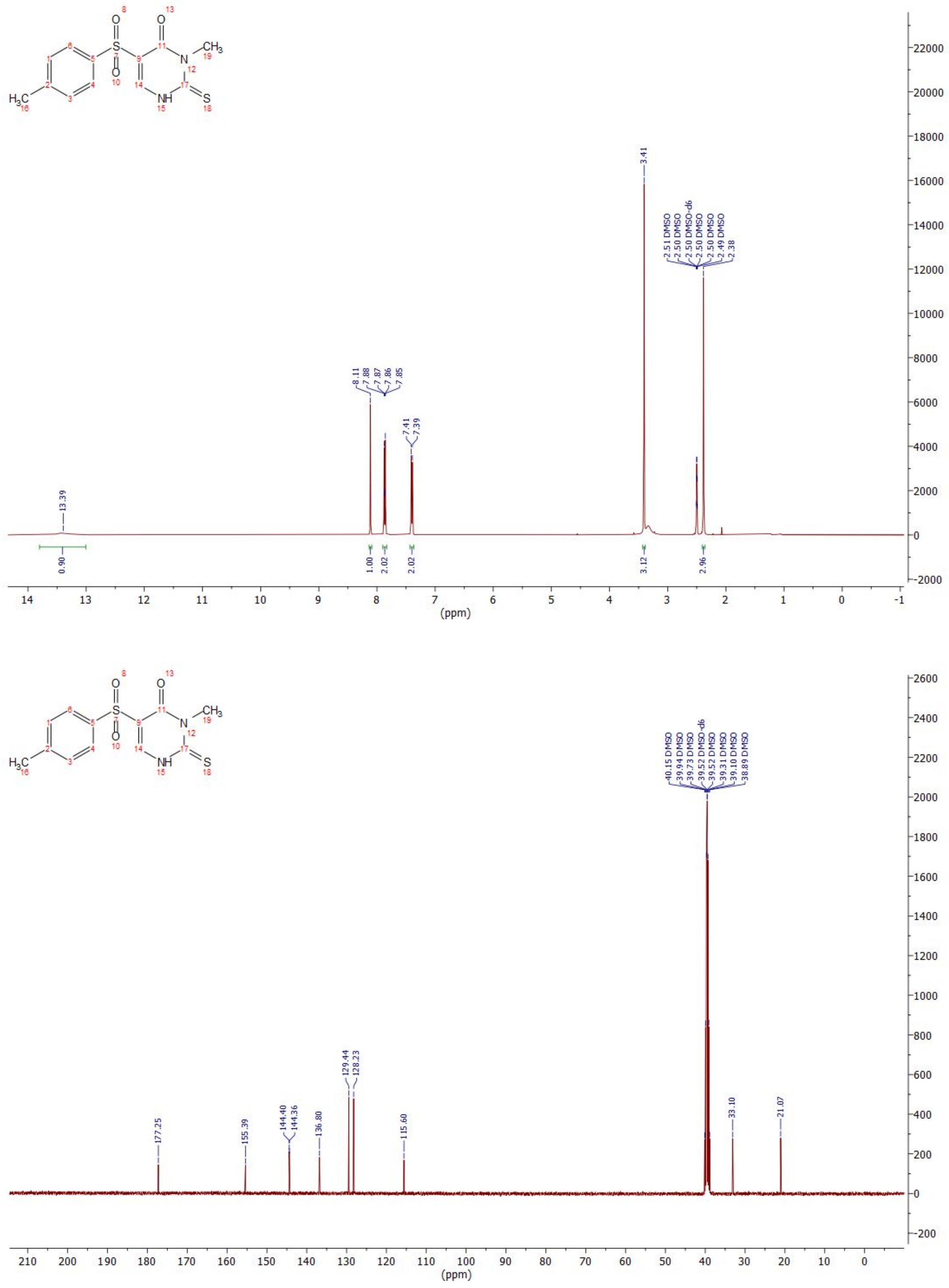

Figure S 93. *^{1}H (400 MHz) and $^{13}C\{1H\}$ (101 MHz) NMR-Spectra in DMSO-d_6 of **12k**.*

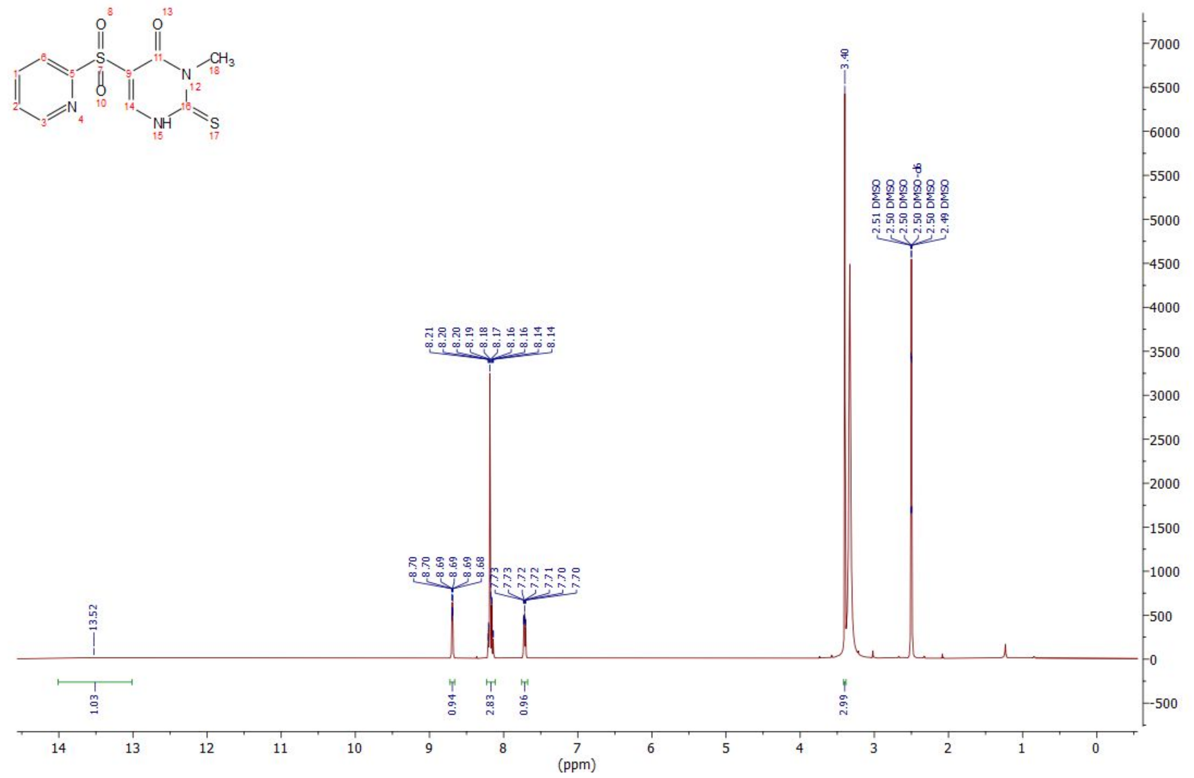

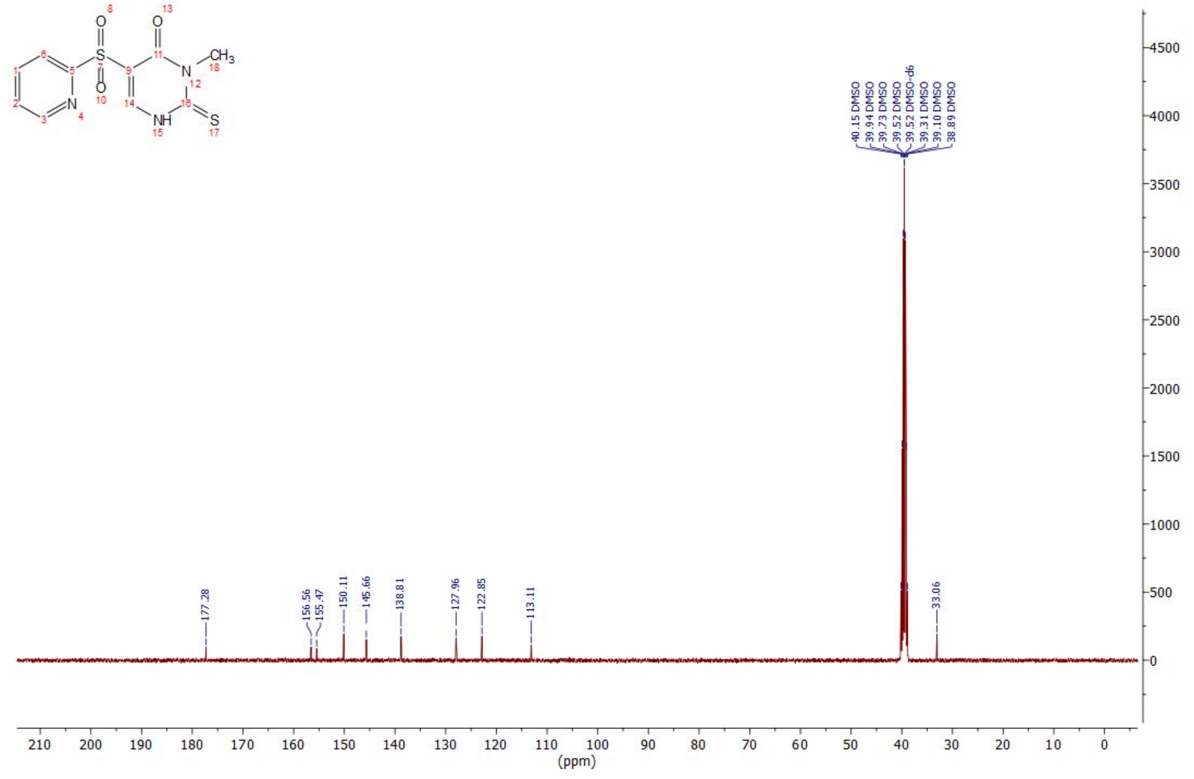

Figure S 94. *^{1}H (400 MHz) and ^{13}C{1H} (101 MHz) NMR-Spectra in DMSO-d_6 of* ***12n****.*

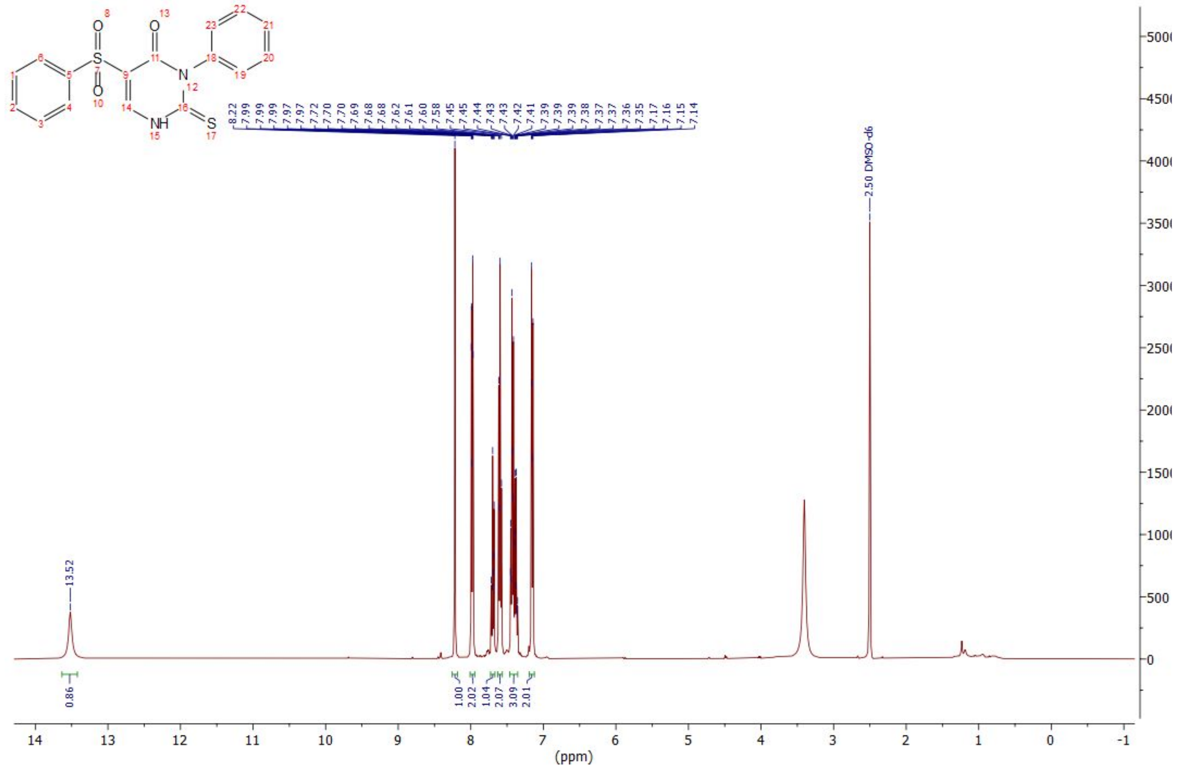

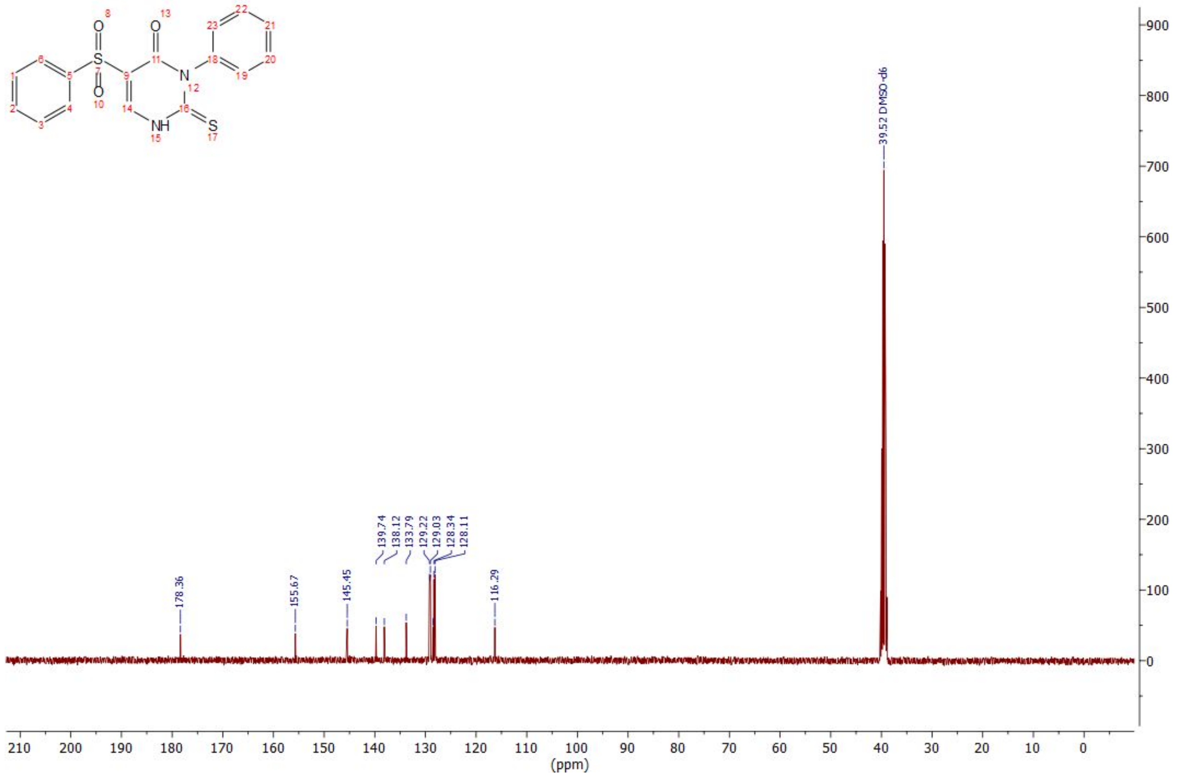

Figure S 95. *^{1}H (400 MHz) and $^{13}C\{^{1}H\}$ (101 MHz) NMR-Spectra in DMSO-d_6 of **13**.*

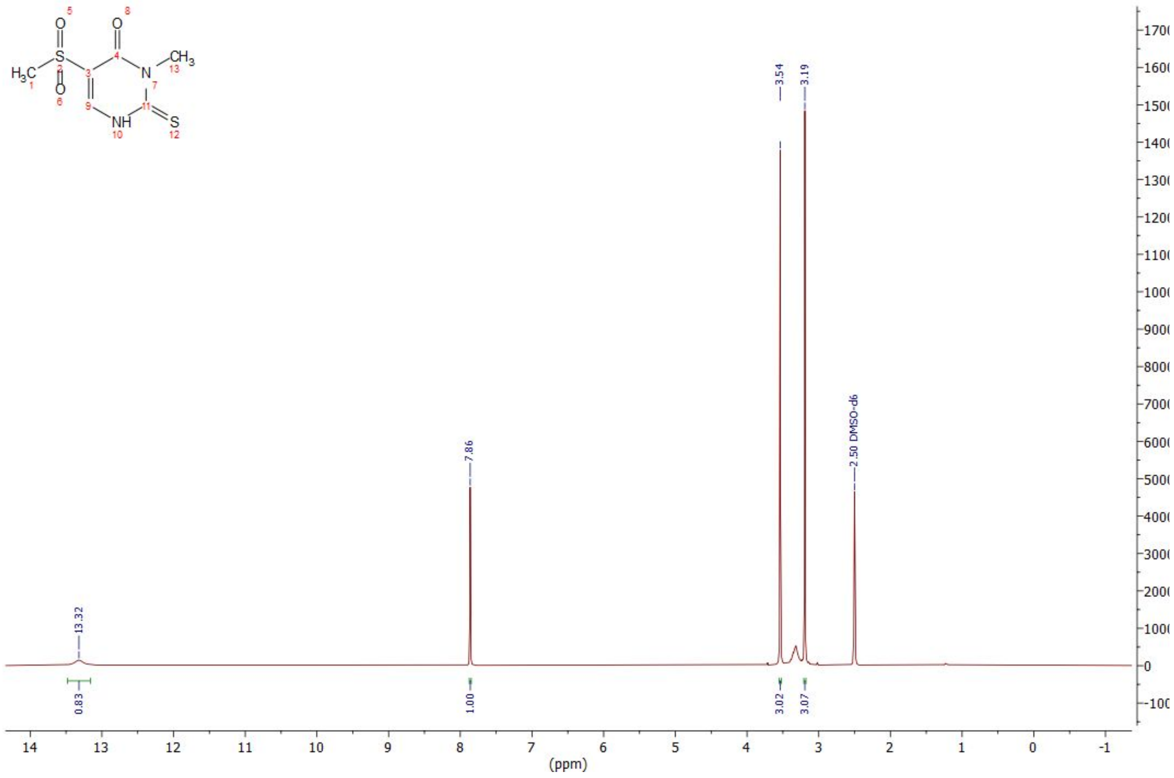

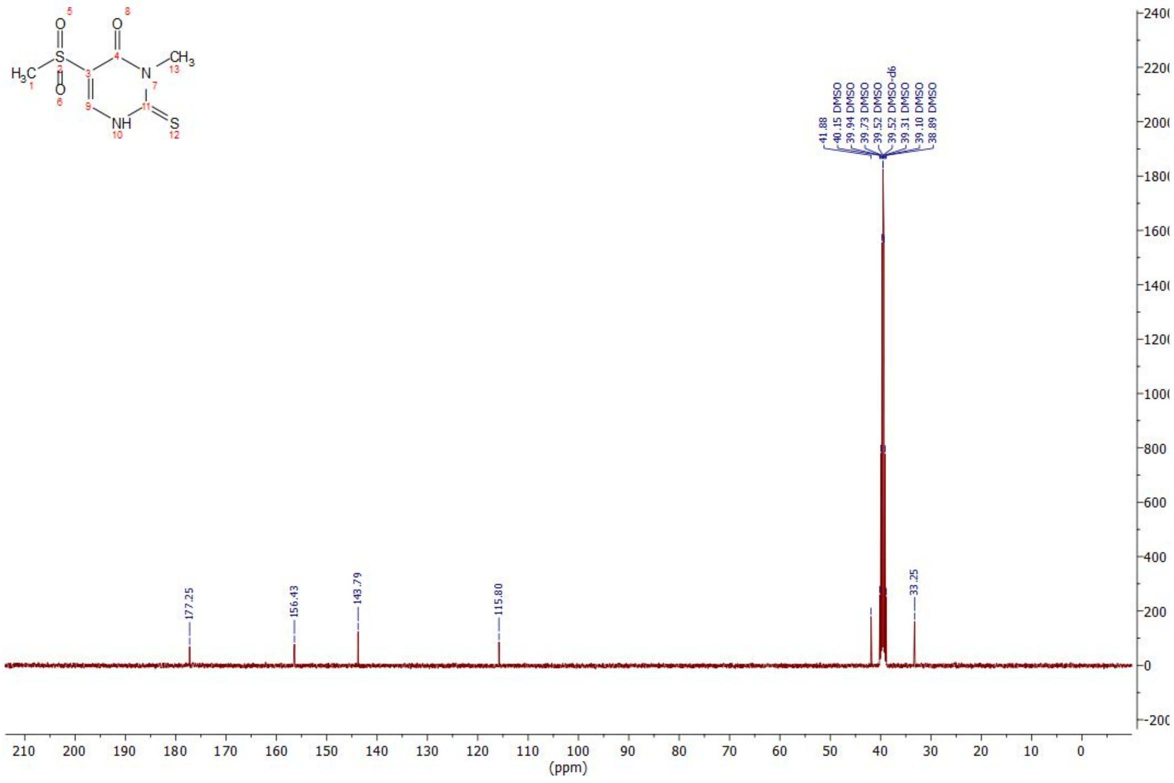

Figure S 96. *^{1}H (400 MHz) and $^{13}C\{^{1}H\}$ (101 MHz) NMR-Spectra in DMSO-d_6 of **12r**.*

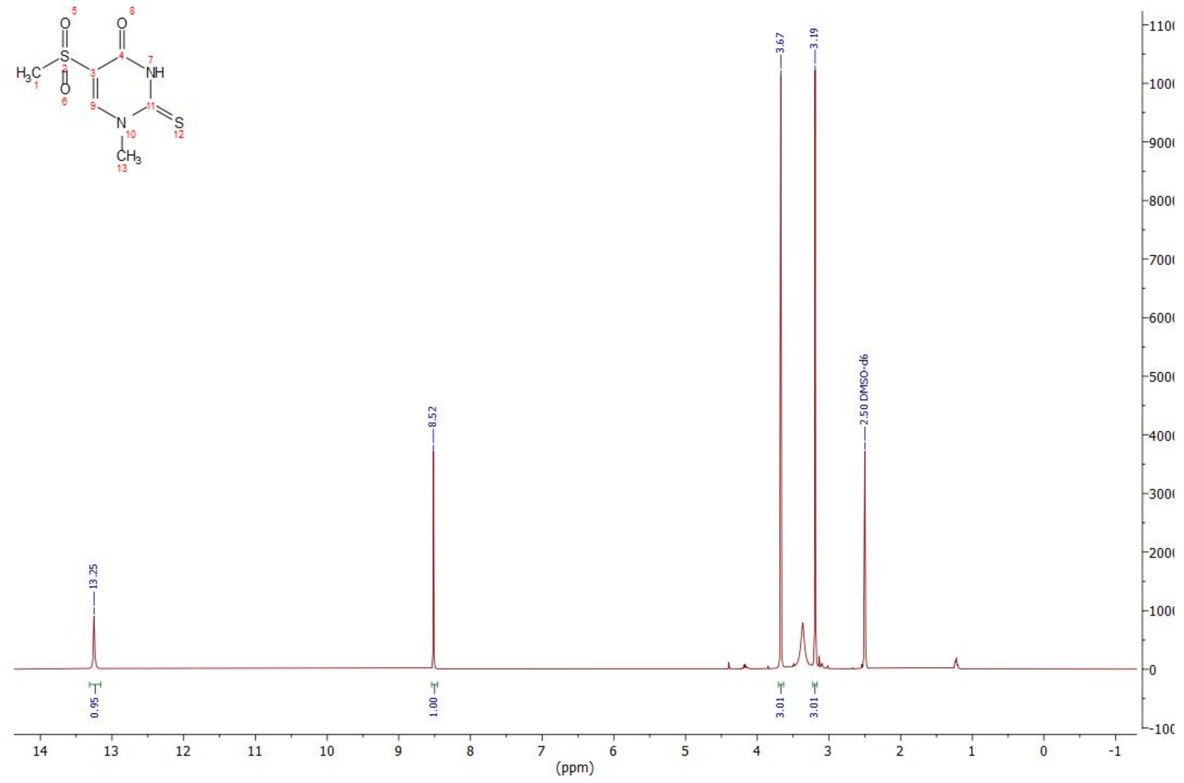

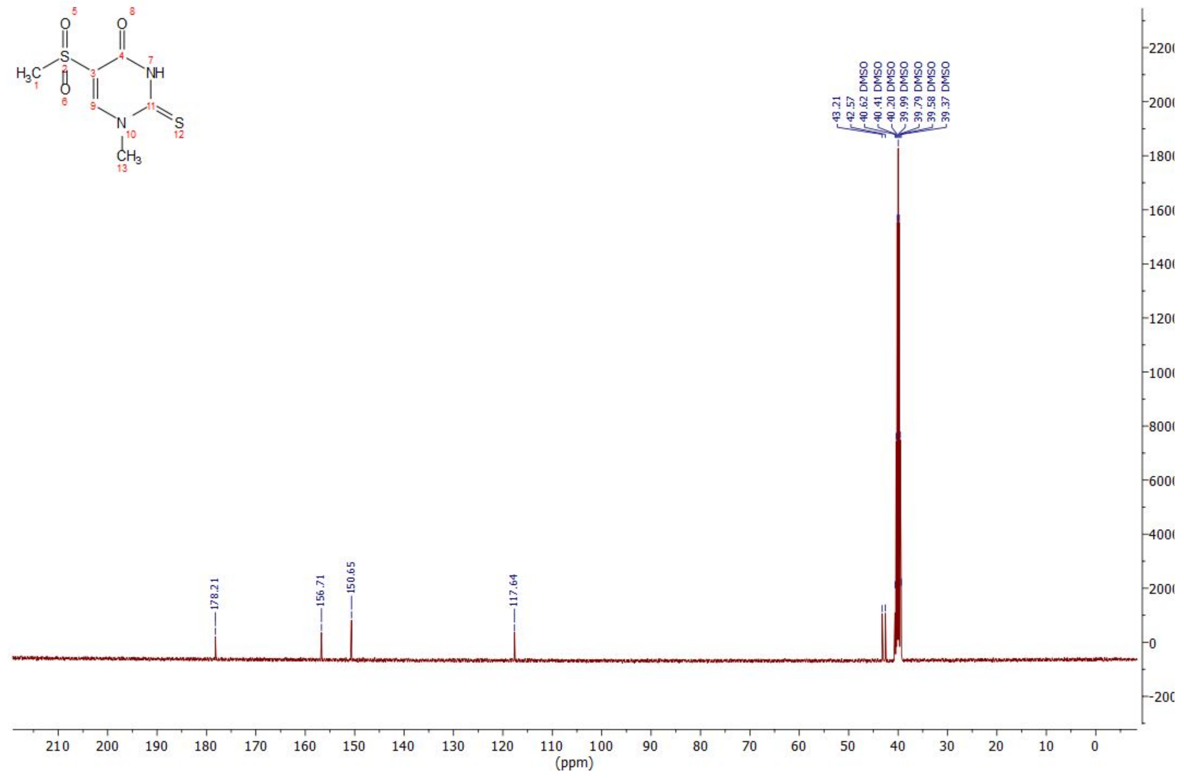

Figure S 97. *^{1}H (400 MHz) and $^{13}C\{^{1}H\}$ (101 MHz) NMR-Spectra in DMSO-d_6 of **12s**.*

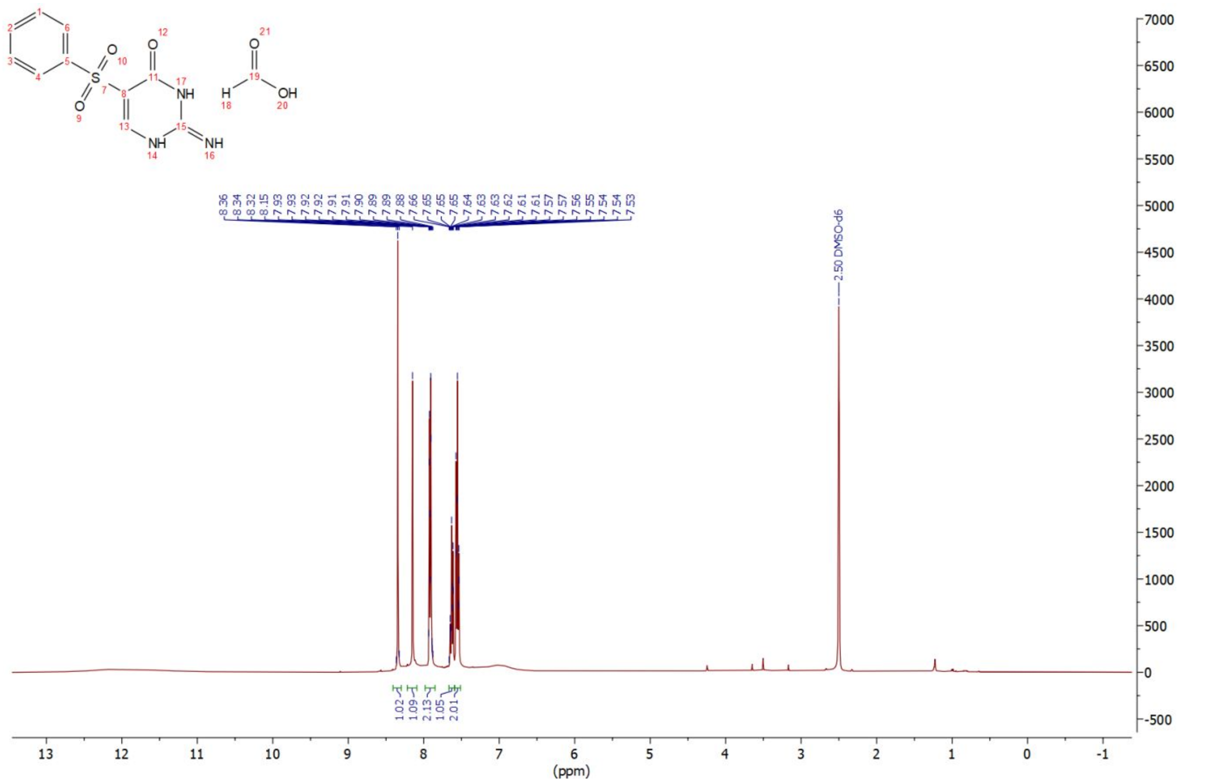

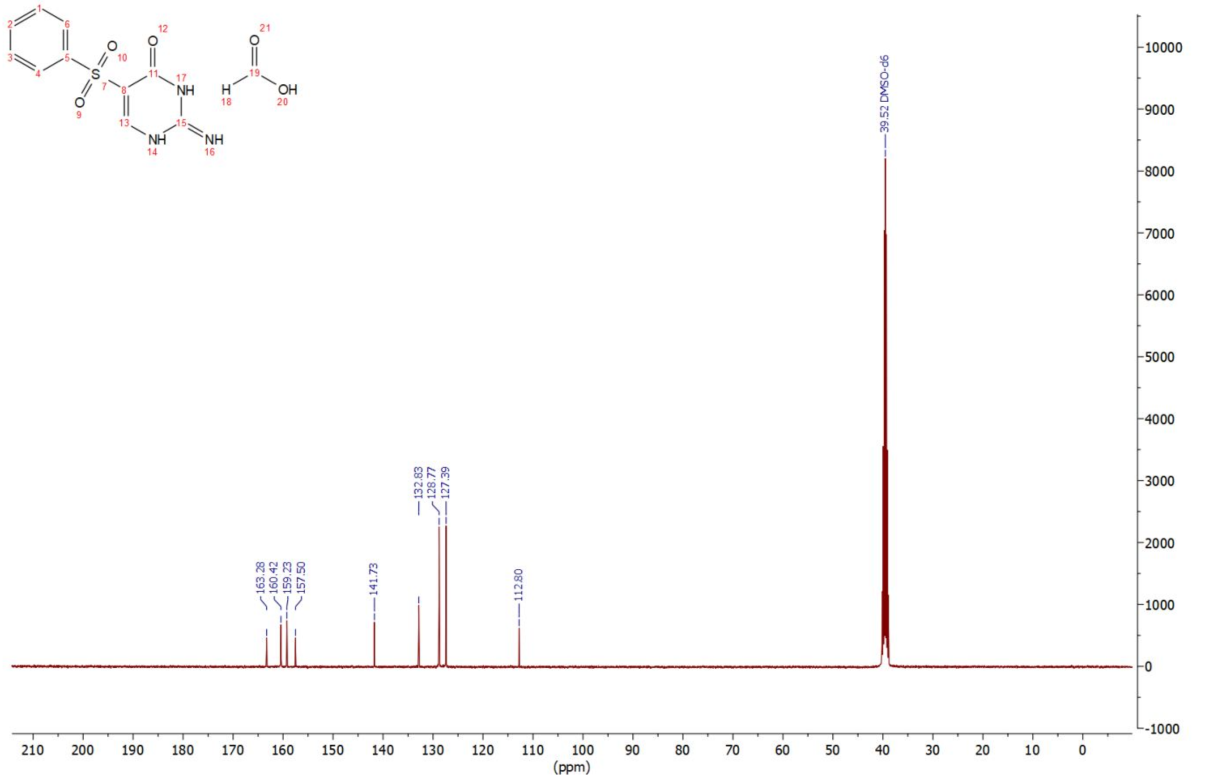

Figure S 98. *^{1}H (400 MHz) and $^{13}C\{1H\}$ (101 MHz) NMR-Spectra in DMSO-d_6 of* ***15a****.*

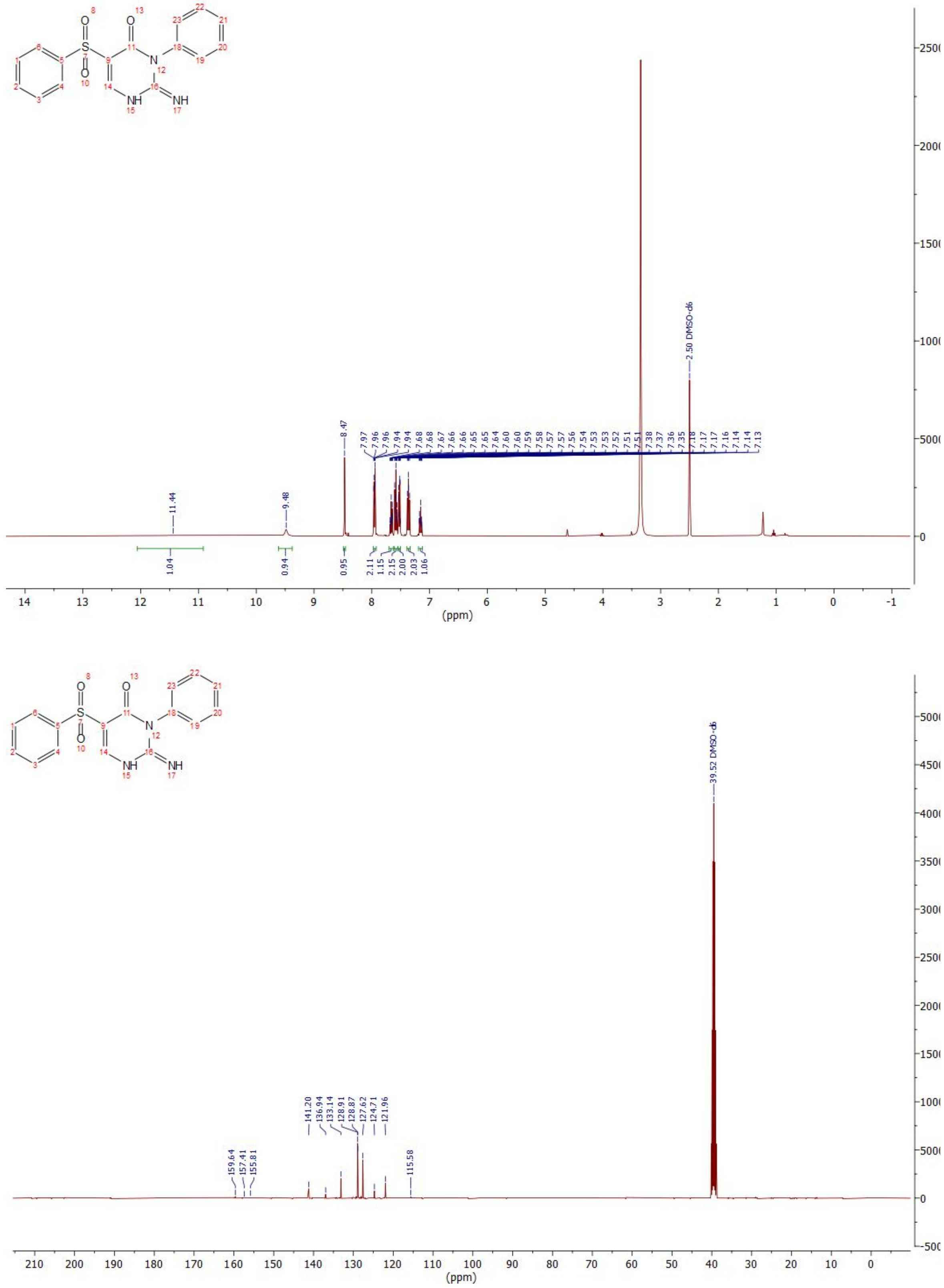

Figure S 99. *^{1}H (400 MHz) and $^{13}C\{^{1}H\}$ (101 MHz) NMR-Spectra in DMSO-d_6 of **15b**.*

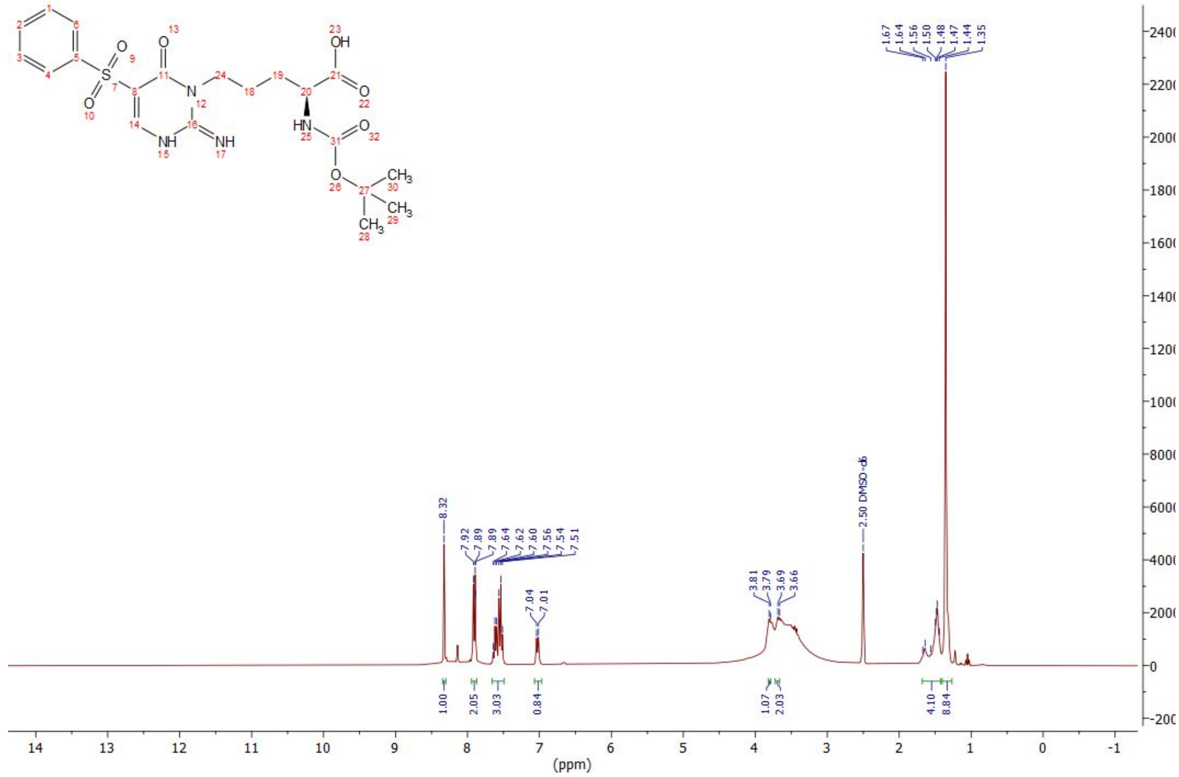

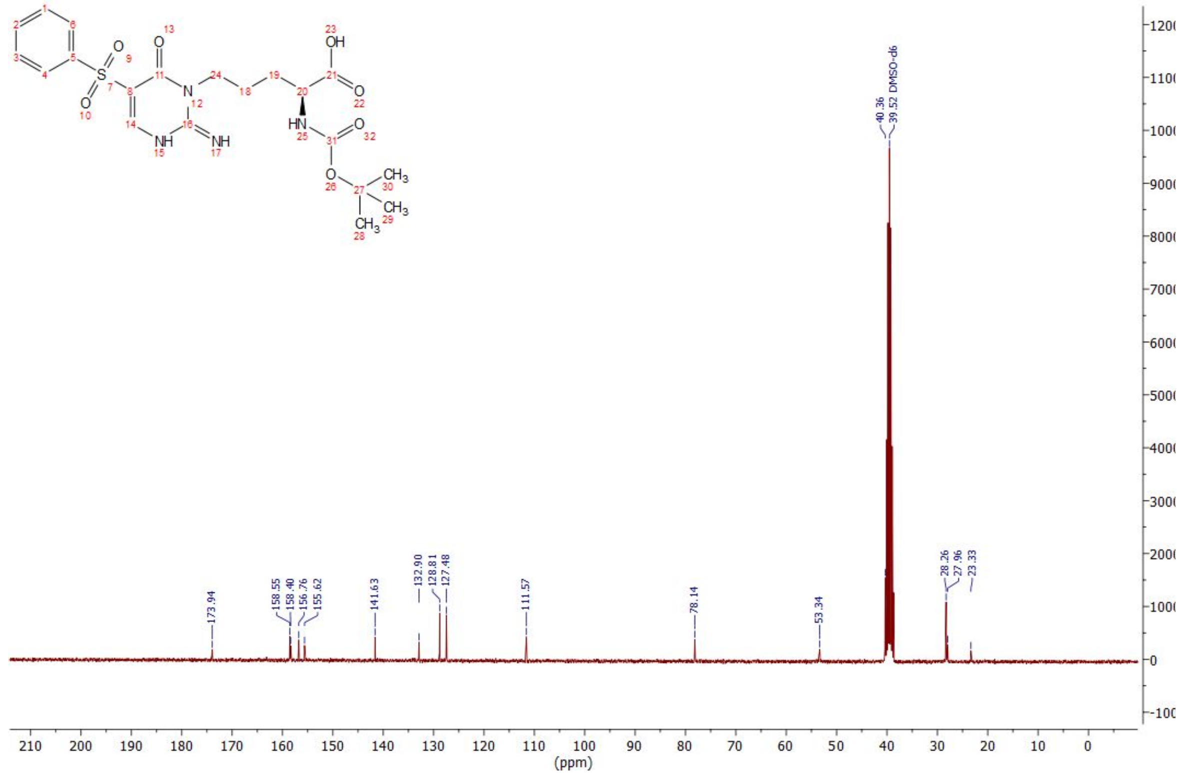

Figure S 100. *^{1}H (300 MHz) and $^{13}C\{^{1}H\}$ (76 MHz) NMR-Spectra in DMSO-d_6 of* ***15c****.*

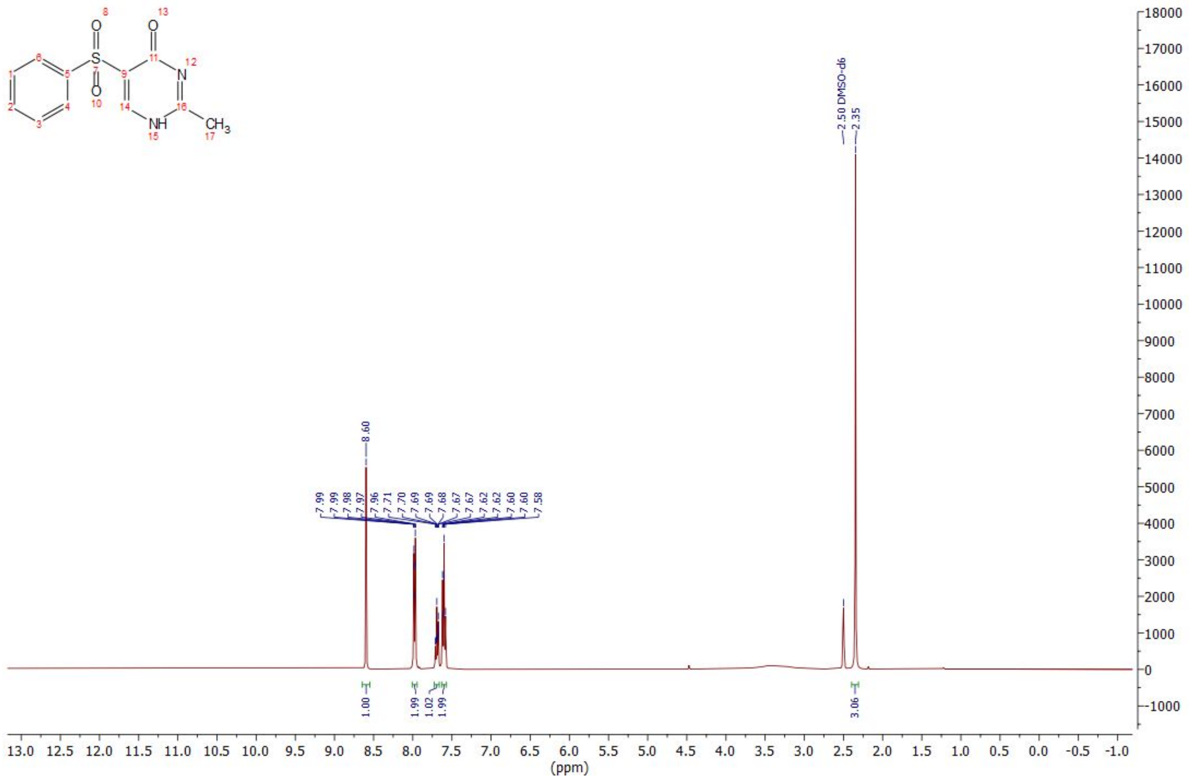

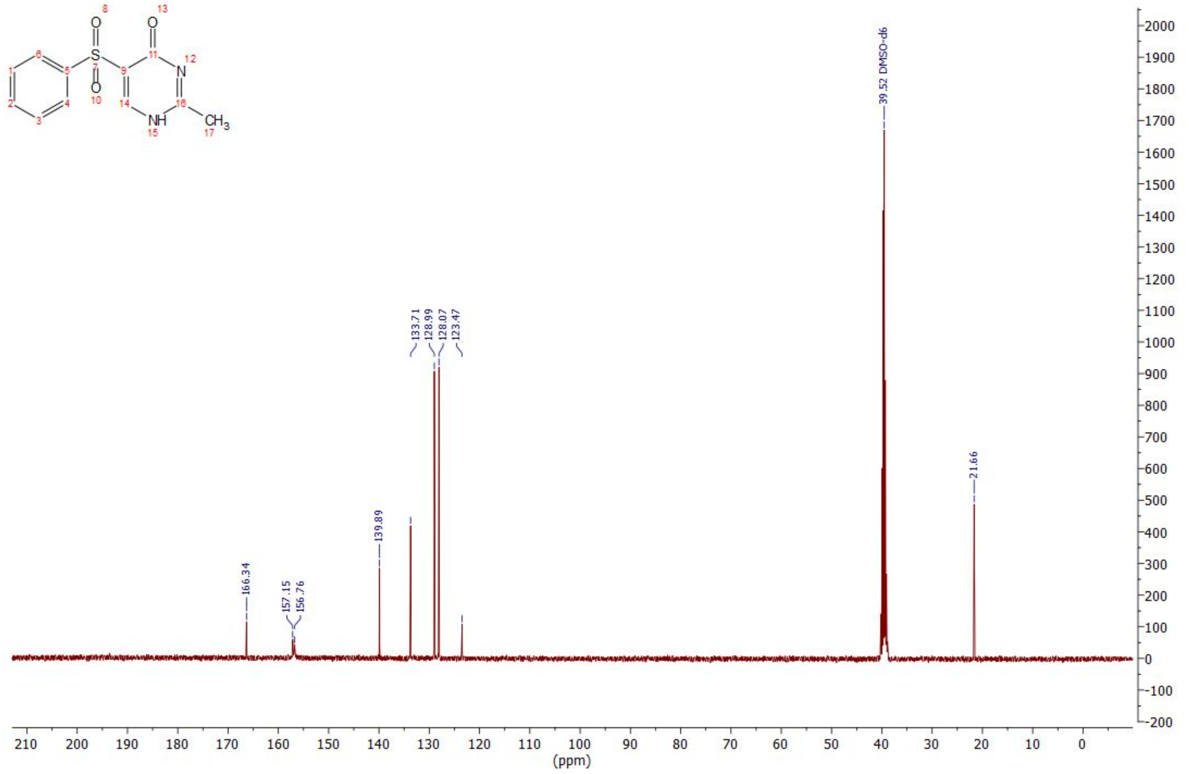

Figure S 101. *^{1}H (400 MHz) and ^{13}C{1H} (101 MHz) NMR-Spectra in DMSO-d_6 of **17**.*

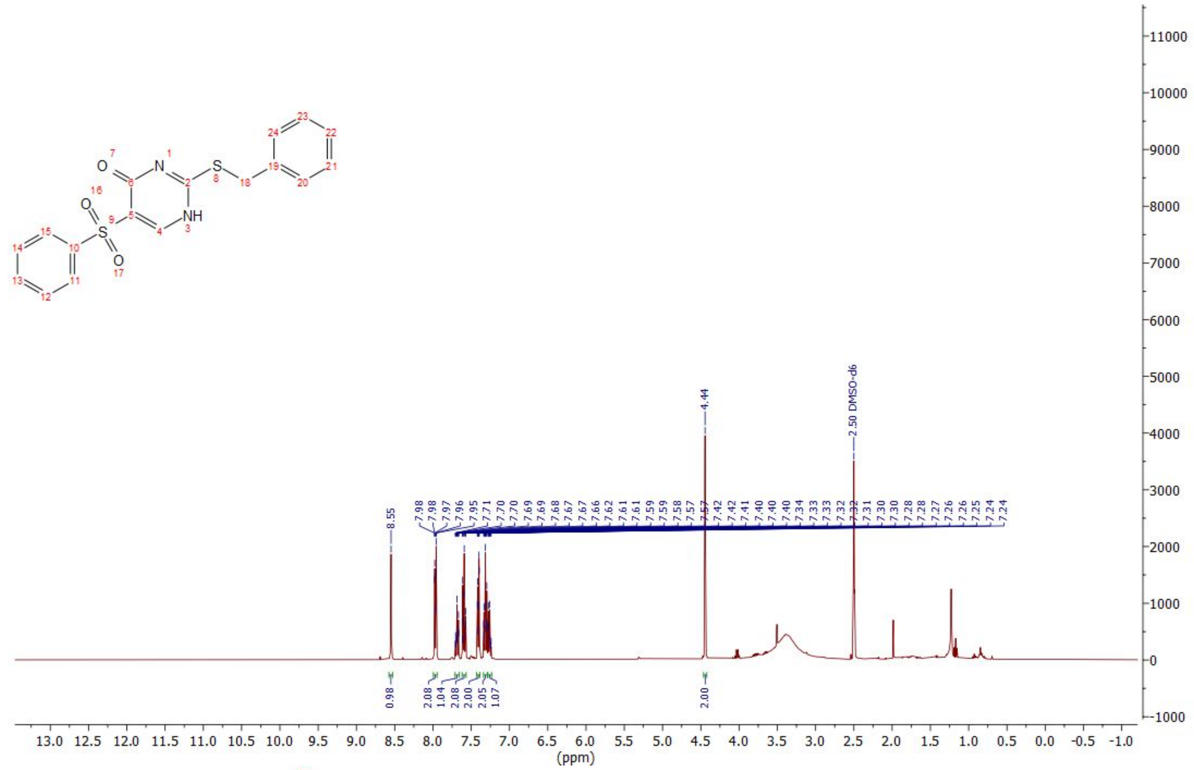

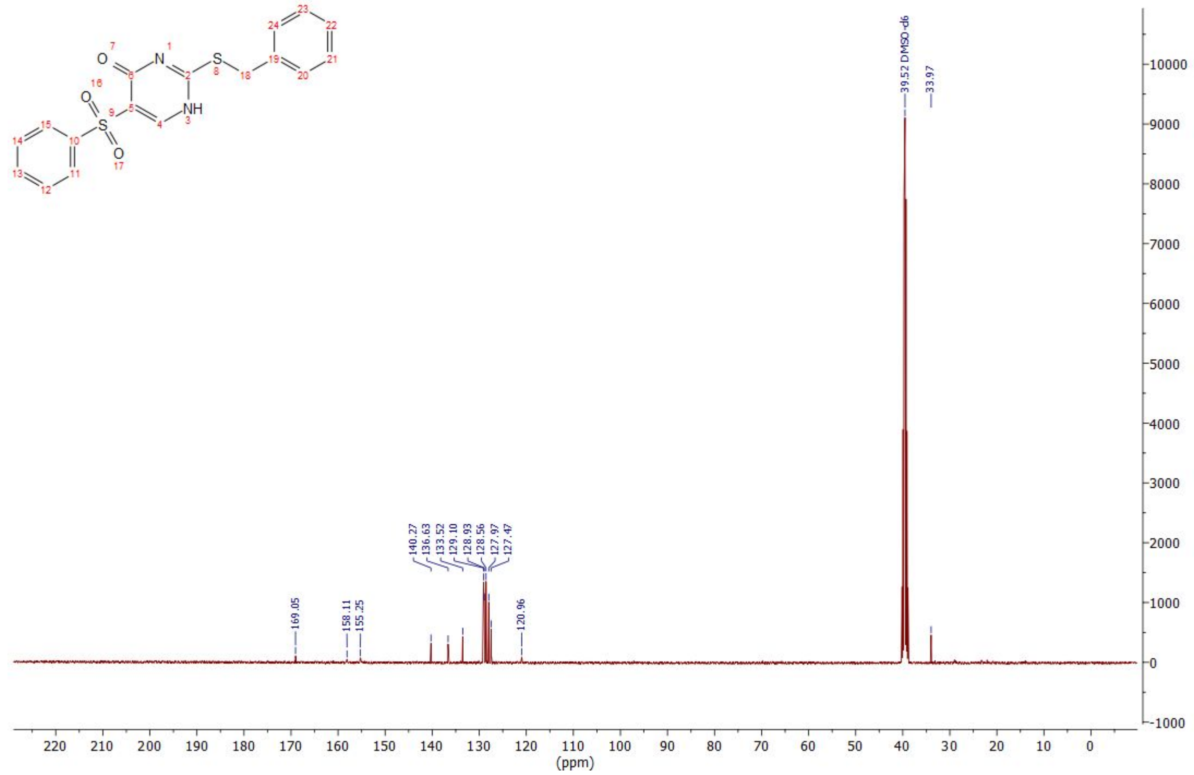

Figure S 102. *^{1}H (400 MHz) and $^{13}C\{1H\}$ (101 MHz) NMR-Spectra in DMSO-d_6 of **19**.*

4. References

(1) Harris, R. K.; Becker, E. D.; Cabral de Menezes, S. M.; Granger, P.; Hoffman, R. E.; Zilm, K. W. Further conventions for NMR shielding and chemical shifts (IUPAC Recommendations 2008). *Pure and Applied Chemistry* **2008**, *80* (1), 59–84. DOI: 10.1351/pac200880010059.

(2) Harris, R. K.; Becker, E. D.; Cabral de Menezes, S. M.; Goodfellow, R.; Granger, P. NMR nomenclature. Nuclear spin properties and conventions for chemical shifts(IUPAC Recommendations 2001). *Pure and Applied Chemistry* **2001**, *73* (11), 1795–1818. DOI: 10.1351/pac200173111795.

(3) Wang, Y.; Zhang, F.; Wang, Y.; Pan, Y. Electrochemistry Enabled Nickel-Catalyzed Selective C−S Bond Coupling Reaction. *Eur J Org Chem* **2022**, *2022* (5). DOI: 10.1002/ejoc.202101462.
